# Diisonitrile Chalkophore Structure and Function in Mycobacterial Copper Acquisition

**DOI:** 10.1021/jacs.6c09506

**Published:** 2026-10-01

**Authors:** Pooja B. Pandya, Chad E. Hatch, John A. Buglino, Yaprak Ozakman, Kate Ryan, Ruofei Faye Li, Farhan A. Chowdhury, Michael S. Glickman, Derek S. Tan

**Affiliations:** † Pharmacology Graduate Program, Weill Cornell Graduate School, 5803 Memorial Sloan Kettering Cancer Center, 1275 York Avenue, New York, New York 10065, United States; ‡ Chemical Biology Program, Sloan Kettering Institute, 132189 Memorial Sloan Kettering Cancer Center, 1275 York Avenue, New York, New York 10065, United States; § Immunology Program, Sloan Kettering Institute, Memorial Sloan Kettering Cancer Center, 1275 York Avenue, New York, New York 10065, United States; ∥ Gerstner Sloan Kettering Graduate School, Memorial Sloan Kettering Cancer Center, 1275 York Avenue, New York, New York 10065, United States; ⊥ Gerstner Sloan Kettering Graduate School, Memorial Sloan Kettering Cancer Center, Mechanistic Biology Summer Program, 1275 York Avenue, New York, New York 10065, United States; # Tri-Institutional Research Program, Memorial Sloan Kettering Cancer Center, 1275 York Avenue, New York, New York 10065, United States

## Abstract

Tuberculosis, the disease caused by *Mycobacterium tuberculosis* infection, is a leading cause of death worldwide. While first-line treatments are effective for many patients, multidrug-resistant tuberculosis poses a continued threat to global public health, and new approaches are needed to provide effective chemotherapy against drug-resistant disease. Diisonitrile lipopeptide chalkophores are a novel class of natural products that play critical roles in copper homeostasis and virulence in *M. tuberculosis* by supplying copper to the heme:Cu oxidase of the respiratory chain. However, the molecular mechanisms by which the bacteria use diisonitriles to mediate copper homeostasis are poorly understood. Herein, we investigate structure–activity relationships of synthetic diisonitriles to identify structural features required for biological function in mycobacterial copper acquisition. We have developed a modular synthetic approach that allows efficient construction of diisonitrile natural products and diverse analogues. We confirmed that a short-chain analogue of the natural products binds tightly to Cu­(I) and Cu­(II), but not Zn­(II), and estimated its Cu­(I) binding affinity. We then used these analogues to probe the impacts of diisonitrile acyl chain length, amino acid backbone, and C-terminal motif on the rescue of *M. tuberculosis* and *Mycobacterium marinum* growth in copper-restricted conditions. The observed structural preferences align with current knowledge of the corresponding natural product structures in these bacteria, but both species also exhibited surprising flexibility in accepting diverse diisonitrile analogues for this function. This work provides new insights into the structural features of diisonitrile chalkophores required for function in mycobacterial copper homeostasis, and the distinct structural preferences between species, while suggesting some structural plasticity in their cellular copper acquisition systems.

## Graphical abstract

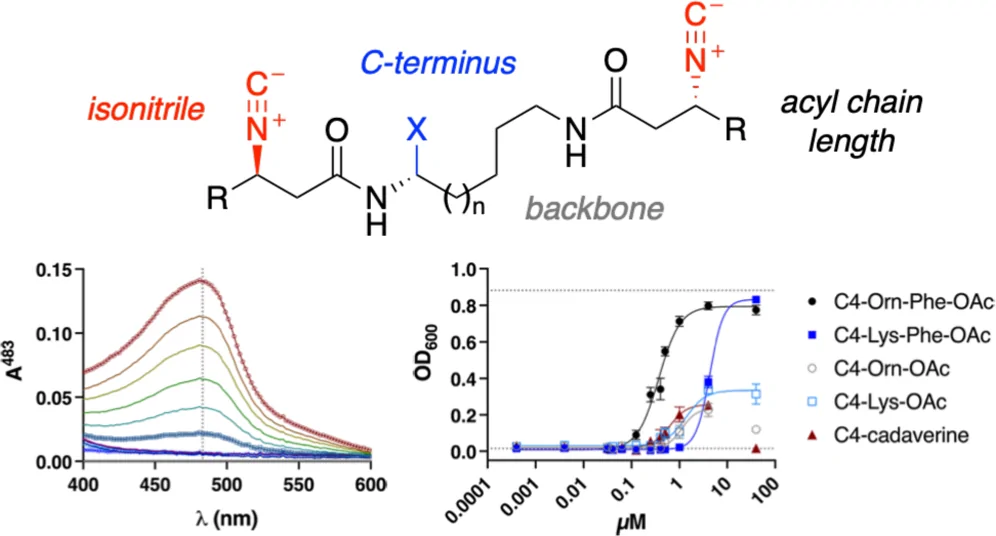

## Introduction

Tuberculosis (TB) is caused by the bacterium *Mycobacterium tuberculosis* and is the leading cause of death by infectious disease in the world.^1,2^ The World Health Organization has enacted a goal to end TB by 2035,^3^ but with the rise of drug-resistant strains,^4^ new drugs are needed to combat TB effectively. First-line treatment for TB is a six-month course of isoniazid and rifampicin and is effective against drug-sensitive strains.^5^ However, multidrug-resistant TB (MDR-TB) and extensively drug-resistant TB (XDR-TB) are resistant to first- and second-line drugs, respectively, and are difficult to treat.^3^ Currently, the best treatment for MDR-TB is a 26 week regimen of bedaquiline, pretomanid, and linezolid that work synergistically to overcome bacterial resistance.^3,4^ In addition to improving MDR-TB treatment, the effectiveness of this regimen also validates the *M. tuberculosis* respiratory chain (bedaquiline targets mycobacterial ATP synthase) as a promising target space for additional inhibitor classes that could further improve treatment of both drug-sensitive and MDR-TB. Herein, we report our investigations of diisonitrile chalkophore natural products that are biosynthesized in *M. tuberculosis* and other mycobacteria and mediate copper homeostasis. We recently showed that these molecules maintain the function of the primary respiratory oxidase in *M. tuberculosis*, thus representing a new aspect of mycobacterial metabolism to target in therapeutic discovery.^6^ We have now developed a modular synthetic route to access a variety of analogues of the *M. tuberculosis* and putative *Mycobacterium marinum* natural products and have tested the ability of these diisonitriles to rescue mycobacterial growth under copper limitation in vitro. Consistent with structural expectations based on the natural products, an ornithine–phenylalaninol backbone was preferred by *M. tuberculosis*, while a lysinol backbone was preferred by *M. marinum*. However, both species accepted a surprisingly wide range of analogues, suggesting that there is considerable tolerance of diisonitrile structural diversity in the cellular systems that mediate diisonitrile function. This work provides new insights into the structure–activity relationships (SAR) of this important copper transport system and sets the stage for future studies to identify the cellular components of this system.

During bacterial infection, host cells sequester copper and other metals as a means of “nutritional immunity” against the pathogens.^7^ Copper is essential for bacterial growth, and thus copper restriction is an effective defense mechanism in the host cell. Conversely, host macrophages also release copper in response to intracellular infection,^8^ leading to antibacterial effects such as oxidative stress and increased formation of reactive oxygen species in *M. tuberculosis*.^9^ The response of *M. tuberculosis* to high levels of copper is mediated by well-studied transcriptional repressors and metal efflux transporters,^6,10,11^ but the mechanisms that bacteria use to overcome copper limitation are poorly understood.

Genetic studies have identified a biosynthetic gene cluster in *M. tuberculosis* that encodes a nonribosomal peptide synthetase pathway.^12–14^ Disruption of this pathway in *M. tuberculosis* reduces its virulence in mice.^6^ Recent work has revealed that this pathway produces novel diisonitrile lipopeptides that bind copper tightly and are required for bacterial growth under copper limitation, identifying them as chalkophores (from “chalkos”, the Greek word for copper) (Figure 1).^15^ While some biological studies have suggested that these diisonitriles function in zinc homeostasis,^12,16–19^ chemical experiments indicate that they do not bind tightly to zinc and, rather, bind copper tightly in 2:1 complexes via their isonitrile (isocyanide) motifs.^20–23^ However, the significance of other structural features has yet to be determined. Notably, these diisonitriles are structurally and functionally distinct from the methanobactin class of chalkophores, which are ribosomally produced, post-translationally modified peptides that bind copper in methanotrophs.^13^

**Figure 1:**
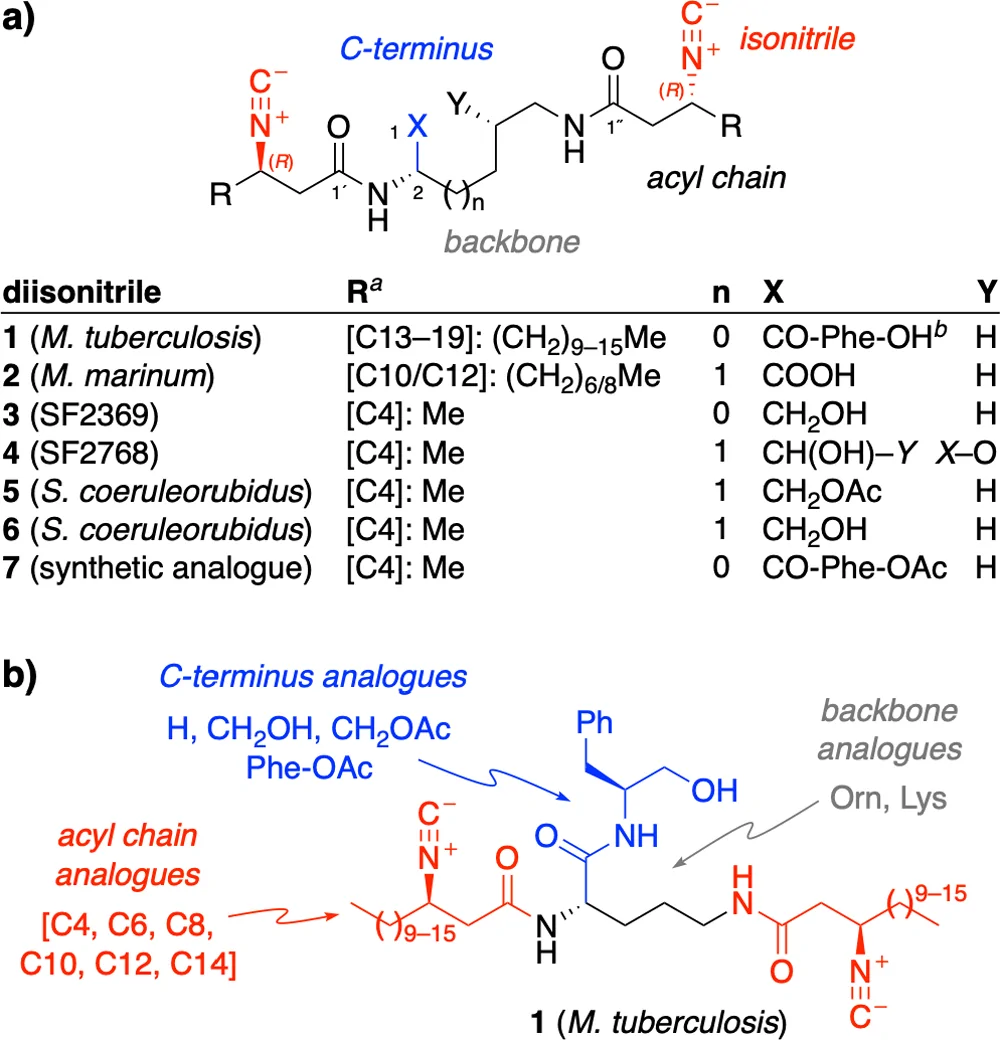
Bacterial diisonitrile lipopeptides. (a) Structures of known diisonitrile lipopeptides **1–6** identified by isolation as natural products or heterologous expression of the biosynthetic gene cluster. Diisonitrile **7** has been synthesized previously as a natural product analogue.^6^ (b) Structures of diisonitrile analogues synthesized and evaluated herein. ^a^Numbers in square brackets refer to overall acyl chain length. ^b^Phe-OH refers herein to phenylalaninol having a reduced C-terminus. Orn = ornithine (*n* = 0), Lys = lysine (*n* = 1).

The cellular machinery that mediates diisonitrile function in mycobacteria is largely unknown. By analogy to related pathways for siderophore-mediated iron uptake and methanobactin-mediated copper uptake in bacteria,^24,25^ this may include proteins that export free diisonitriles into the host environment, import diisonitrile–copper complexes back into the bacteria, release the copper from the complex, and deliver the copper to intracellular cuproproteins that require it for their functions.^18,20^ The structural features of the diisonitrile chalkophores that are required for integration with these cellular systems are also unknown. To investigate these requirements, we sought to synthesize and evaluate a series of diisonitrile natural product analogues to elucidate SAR for rescue of mycobacterial growth in copper-restricted conditions.

## Results and Discussion

### Total Synthesis of a *M. tuberculosis* Diisonitrile Natural Product and Analogues

Gokhale and co-workers have elucidated the structures of the *M. tuberculosis* diisonitrile natural products **1** by mass spectrometry (Figure 1a).^12^ They are comprised of an ornithine (Orn) backbone, C-terminal phenylalaninol (Phe-OH) residue, and β-isonitrile acyl chains ranging from 13 to 19 carbons (C13 to C19) in length. Chang and workers have synthesized the C14-Orn-Phe-OH natural product and confirmed its copper-binding activity.^22^ More recently, Aldrich and co-workers have synthesized C12 and C18 congeners and investigated their metal-binding properties.^19^ The *M. tuberculosis* diisonitriles differ from those produced in other bacteria with homologous biosynthetic gene clusters that have a lysine (Lys) backbone, lack the C-terminal Phe-OH residue, and/or have short C4 acyl chains (**3–6**).^16,20,26,27^ In particular, the *M. marinum* diisonitriles **2** are predicted to have a Lys backbone, no C-terminal residue, and medium-length (C10–C12) acyl chains, based on heterologous expression studies.^16^

Prior work from our groups has demonstrated that a synthetic diisonitrile C4-Lys-OAc (**5**) can rescue the growth of *M. tuberculosis* and *M. marinum* under copper-restricted conditions,^10^ as can C4-Orn-Phe-OAc (**7**) in *M. tuberculosis*.^6^ Notably, these synthetic diisonitriles have 4-carbon acyl chains, in contrast to the longer side chains in the *M. tuberculosis* natural products and putative *M. marinum* natural products.^20^ Further, *M. tuberculosis* growth was also rescued by C4-Lys-OAc (**5**), which has a Lys rather than an Orn backbone and lacks the C-terminal Phe-OH residue of the *M. tuberculosis* natural products **1**. Taken together, these data suggest that mycobacterial copper acquisition systems may tolerate some structural diversity in diisonitriles.

To investigate this SAR, we developed a modular synthetic approach to access diisonitriles with varying backbones, C-terminal motifs, and acyl chain lengths and stereochemistry (Figure 1b). Diisonitrile analogues were designed to have an Orn or a Lys backbone, corresponding to the *M. tuberculosis* and predicted *M. marinum* natural products, respectively. The C-terminus was designed to include the Phe-OH residue of the *M. tuberculosis* natural product, as well as the corresponding phenylalaninol acetate (Phe-OAc) and truncated acetate (OAc), alcohol (OH), or proton (H; based on cadaverine) motifs.

We have previously reported that the C4 β-isocyanoacyl chain **15a** can be synthesized from the chiral pool (Figure 2).^21^ However, access to longer side chains required development of an enantioselective synthesis. Thus, we investigated approaches to stereoselective installation of the β-isonitrile motif, targeting the *R* enantiomers based on the known stereochemistry of C4 diisonitrile chalkophores.^16,21,23^ Attempted diastereoselective Reformatsky reaction of the zinc enolate of *t*-butyl acetate with the *S*-phenylglycine imine of 1-decanal^28^ resulted in a modest yield and diastereoselectivity (16%, 4:1 dr), with unexpected formation of a β-lactam side product. Mannich reaction of methyl acetate with the (*R*)-*t*-butylsulfinimine of 1-decanal (**9d**)^29^ also provided a modest yield (21%) but high diastereoselectivity (98:2 dr). Moreover, Reformatsky reaction with the same (*R*)-*t*-butylsulfinimine **9d** afforded the desired β-(*R*)-*t*-butylsulfinamide **11d** in a 78% yield and 98:2 dr (Figure 2). Use of a freshly prepared Reformatsky reagent rather than commercially available reagent was required to achieve this high diastereoselectivity. The stereochemical configuration was confirmed by ^1^H NMR and optical rotation analysis of the corresponding hydrolyzed β-aminoester **12d** compared to the literature.^28^ While this manuscript was in preparation, Aldrich and co-workers reported a closely related approach using Mannich-type reactions of the titanium enolate of methyl acetate with (*R*)-*t*-butylsulfinimines **9**.^19^ In their earlier synthesis of the *M. tuberculosis* natural product C14-Orn-Phe-OH (C14-**1**), Chang and co-workers used a diastereoselective Michael addition to *t*-butyl 2-decenoate to provide stereoselective access to β-isonitrile acyl chains.^22^

**Figure 2:**
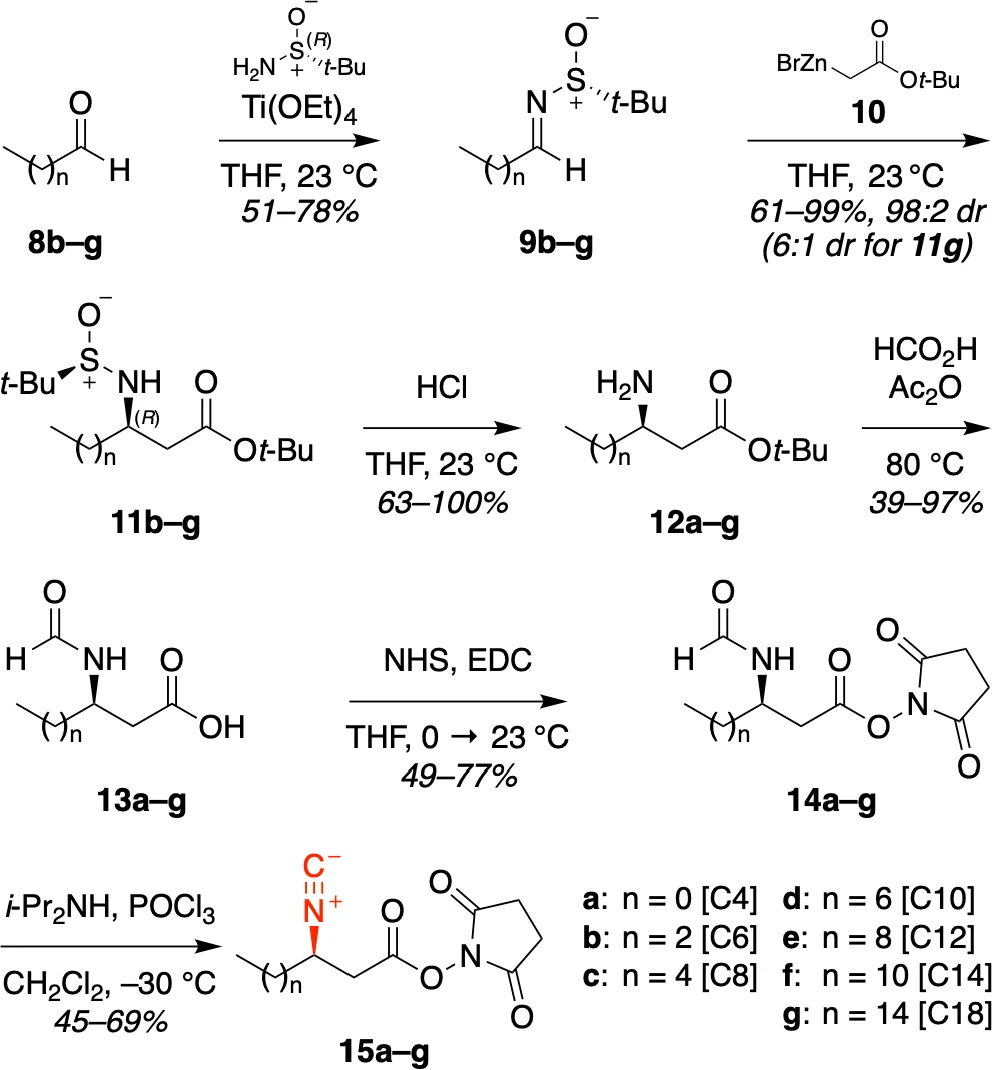
Diastereoselective synthesis of (*R*)-β-isocyanoacyl chains. EDC = 1-ethyl-3-(dimethylaminopropyl)­carbodiimide; NHS = *N*-hydroxysuccinimide; THF = tetrahydrofuran.

We then implemented this diastereoselective synthesis in a modular route to the corresponding diisonitrile analogues. The C6, C8, C10, C12, C14, and C18 β-aminoesters **12b–g** were synthesized accordingly. Formamidation and concomitant ester cleavage yielded formamides **13**,^21^ which were then converted to *N-*hydroxysuccinimide (NHS) esters **14**.^21^ Dehydration with phosphoryl chloride provided isonitriles **15** with chain lengths up to 14 carbons.^21^ Due to solubility limitations at longer chain lengths, the substrate concentration was reduced from 0.3 to 0.2 M in the dehydration of the C14 substrate **14f**, while the corresponding C18 substrate **14g** was insoluble at rt in numerous solvents evaluated.

Synthesis of the Orn-Phe-OAc backbone began with the activation of bis­(Boc)-l-Orn (**16**) to NHS ester **17**, which was coupled to Phe-OH (**18**) to furnish dipeptide **19** (Figure 3a). Acetylation of the C-terminal hydroxyl group and TFA deprotection provided the Orn-Phe-OAc core **21**. Coupling with C4 to C14 side chains **15a–f** (Figure 2) yielded diisonitrile analogues **7** and **22b–f**. Acetate deprotection of C4-Orn-Phe-OAc (**7**) and its C14 congener (**22f**) yielded the corresponding alcohols **23a** and **23f**, respectively. The latter natural product **23f** had analytical data matching that reported by Chang and co-workers (Supporting Information Figure S1) and was accessed in 8 steps (longest linear sequence).^22^

**Figure 3:**
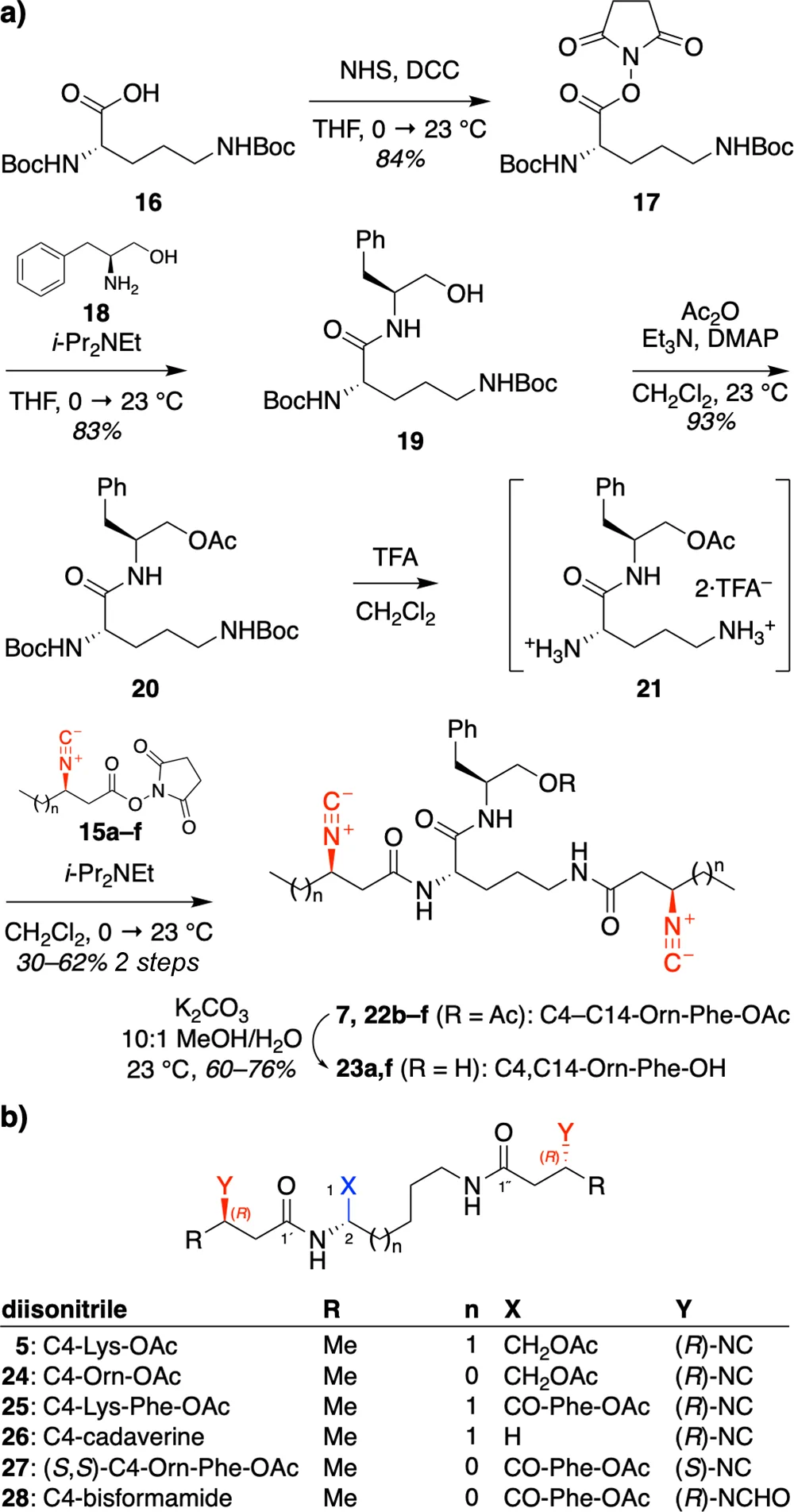
Synthesis of diisonitrile analogues. (a) Synthesis of the Orn-Phe-OH backbone and coupling of β-isocyanoacyl side chains to afford diisonitrile analogues with varied chain lengths. Boc = *t*-butoxycarbonyl; DCC = dicyclohexylcarbodiimide; DMAP = 4-(dimethylamino)pyridine; TFA = trifluoroacetic acid. (b) Structures of additional analogues synthesized with modified backbone lengths, C-terminal motifs, and β-substituents (*R*,*R* diastereomer shown). See Supporting Information for synthetic details.

To evaluate the SAR of the backbone and C-terminus, we also synthesized several additional analogues and controls (Figure 3b). The C4-Lys-OAc analogue (**5**) was synthesized as previously described,^16,21^ and the corresponding ornithine analogue C4-Orn-OAc (**24**) was synthesized analogously (Supporting Information Figure S2). We also synthesized the lysine congener of the short-chain natural product analogue, C4-Lys-Phe-OAc (**25**) (Supporting Information Figure S3), and a C-terminally truncated C4-cadaverine analogue (**26**) (Supporting Information Figure S4). The diastereomeric (*S*,*S*)-C4-Orn-Phe-OAc analogue (**27**) and hydrated (*R*,*R*)-C4-bisformamide analogue (**28**) were also synthesized as controls via analogous routes (Supporting Information Figures S5 and S6).

### Metal-Binding Properties of Diisonitrile Chalkophores

We next investigated the metal-binding properties of the *M. tuberculosis* natural product analogue C4-Orn-Phe-OAc (**7**). In titration of C4-Orn-Phe-OAc (**7**) with [Cu­(MeCN)_4_]­PF_6_ in 3.3:1 PBS-*d*/DMSO-*d*_6_, the diastereotopic H^5a/b^ protons of the diisonitrile backbone separated from two multiplets at 3.18 and 3.26 ppm to 3.05 and 3.36 ppm, respectively, and a side chain H^3^′^^ proton at 4.17 ppm merged with another peak at 4.21 ppm (Figure 4). The shifts reached maximum amplitudes by 0.6 equiv Cu­(I), consistent with the formation of a 2:1 diisonitrile-to-metal complex.^20–22^ Titration of C4-Orn-Phe-OAc (**7**) with CuSO_4_ resulted in similar shifts of the H^5a/b^ and H^3^′^^ protons, again reaching maximum amplitude at 0.6 equiv Cu­(II), albeit with line broadening due to the paramagnetic character of Cu­(II). In contrast, upon addition of ZnBr_2_, no changes to the diisonitrile peaks were observed up to 1 equiv Zn­(II). Notably, Aldrich and co-workers reported a shift in the isocyanide α-proton of C12-Orn-Phe-OH when complexed with ZnBr_2_ in THF (4 h), followed by evaporation (12 h) and NMR analysis in CDCl_3_,^19^ but we observed no such NMR shift with C4-Orn-Phe-OAc in DMSO-*d*_6_ or PBS-*d*/DMSO-*d*_6._ This effective Cu binding and lack of Zn binding under aqueous conditions is consistent with our group’s earlier study of C4-Lys-OAc (**5**)^21^ and Chang and co-workers’ recent studies of C14-Orn-Phe-OH (**23f**), C4-Orn-Phe-OH (**23a**), and C4-Orn-Phe-OAc (**7**).^22^ This is also consistent with a recent report by Zhang and co-workers that a *Mycobacterium fortuitum* diisonitrile, fortcupinile A (C12-Lys-COOMe), also binds to Cu­(I) and Cu­(II), but not Zn­(II), based on mass spectrometric and colorimetric assays.^23^

**Figure 4:**
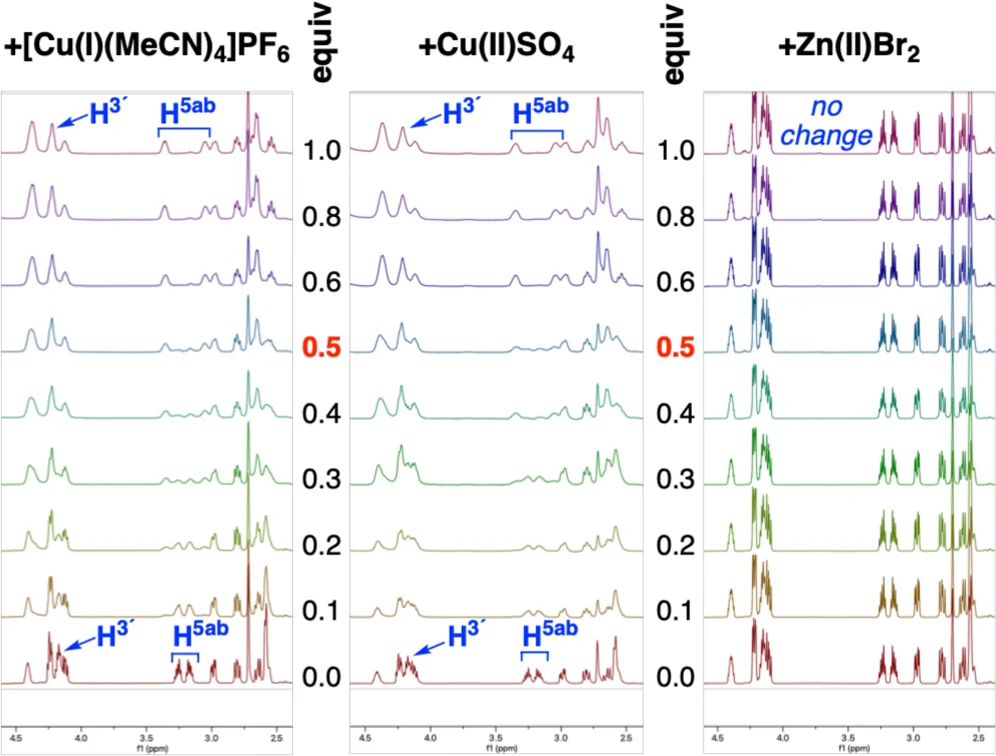
Metal binding of diisonitrile analogues. ^1^H NMR titrations of C4-Orn-Phe-OAc (**7**) with Cu­(I), Cu­(II), and Zn­(II) in 3.3:1 PBS-*d*/DMSO-*d*_6_.

He and co-workers have observed that the Cu­(II) complexes of SF2768 (**4**), C4-Lys-OAc (**5**), and C4-Lys-OH (**6**) were detected as the corresponding Cu­(I) complexes by HR-ESI-MS, suggesting possible in situ reduction.^20^ However, Chang and co-workers recently reported that ^1^H NMR titrations of C4-Lys-OAc (**5**) and C4-Lys-OH (**6**) resulted in line broadening, which they interpreted as binding to paramagnetic Cu­(II) without reduction to Cu­(I). To evaluate this question in the context of the *M. tuberculosis* diisonitrile analogue C4-Orn-Phe-OAc (**7**), we formed the Cu­(II) complex with 0.5 equiv of CuSO_4_. In LC–MS analysis, we observed a signal at 520.1 *m*/*z* as expected for the 2:1 complex with Cu­(II) [2M + Cu]^2+^, as well as another signal at 977.4 *m*/*z*, corresponding to a Cu­(I) complex [2M + Cu]^+^, consistent with the observations of He and co-workers.^20^

To investigate this further, we performed ^63^Cu-NMR experiments with (C4-Orn-Phe-OAc)_2_·Cu­(II), (C4-Orn-Phe-OAc)_2_·Cu­(I), and benzylisonitrile (BnNC)_4_·Cu­(I) complexes in 3.3:1 PBS-*d*/DMSO-*d*_6_ (Supporting Information Figure S7). Cu­(I) is diamagnetic and can be observed by ^63^Cu-NMR, while Cu­(II) is paramagnetic and generally not observed due to line broadening. No ^63^Cu-NMR signals were observed in the sample of (C4-Orn-Phe-OAc)_2_·Cu­(II), while a ^63^Cu peak was readily observed at 466.6 ppm in the sample of (C4-Orn-Phe-OAc)_2_·Cu­(I), as well as at 442.8 ppm in the sample of (BnNC)_4_·Cu­(I). These results indicate that complexation of Cu­(II) by the diisonitrile is insufficient to reduce the metal center to Cu­(I), although it is still possible that this complexation may facilitate reduction under certain conditions, such as in-source reduction during MS analysis.

Next, to assess the binding affinity of the C4-Orn-Phe-OAc (**7**) analogue to Cu­(I), we initially attempted isothermal calorimetry measurements, but the resulting thermograms had weak and highly variable signals, possibly resulting from poor solubility of the Cu­(I) complexes.^23,30,31^ Thus, we conducted a UV–vis competition experiment with BCS (bathocuproinedisulfonic acid), which forms a 2:1 complex with Cu­(I) with an overall two-step association constant of β_2_ = 10^19.8^ M^–2^.^32^ We have previously used this method to evaluate the relative Cu­(I) binding affinity of C4-Lys-OAc (**5**) and BCS.^21^ Titration of a preformed complex of 2:1 BCS·[Cu­(MeCN)_4_]­PF_6_ with C4-Orn-Phe-OAc (**7**) resulted in a linear decrease in BCS absorbance (483 nm) that reached a maximum at approximately 1 equiv of diisonitrile (relative to Cu­(I)), indicating that the diisonitrile binds to Cu­(I) much more strongly than BCS (Figure 5a,b). This stoichiometry also indicates that only the BCS_2_·Cu complex absorbs at this wavelength, while the presumed mixed complex BCS·Cu·**7** does not. Notably, it was essential to carry out the titration under an inert atmosphere (Ar) as titrations in air led to highly variable results, which we attributed to in situ oxidation to Cu­(II) species.

**Figure 5:**
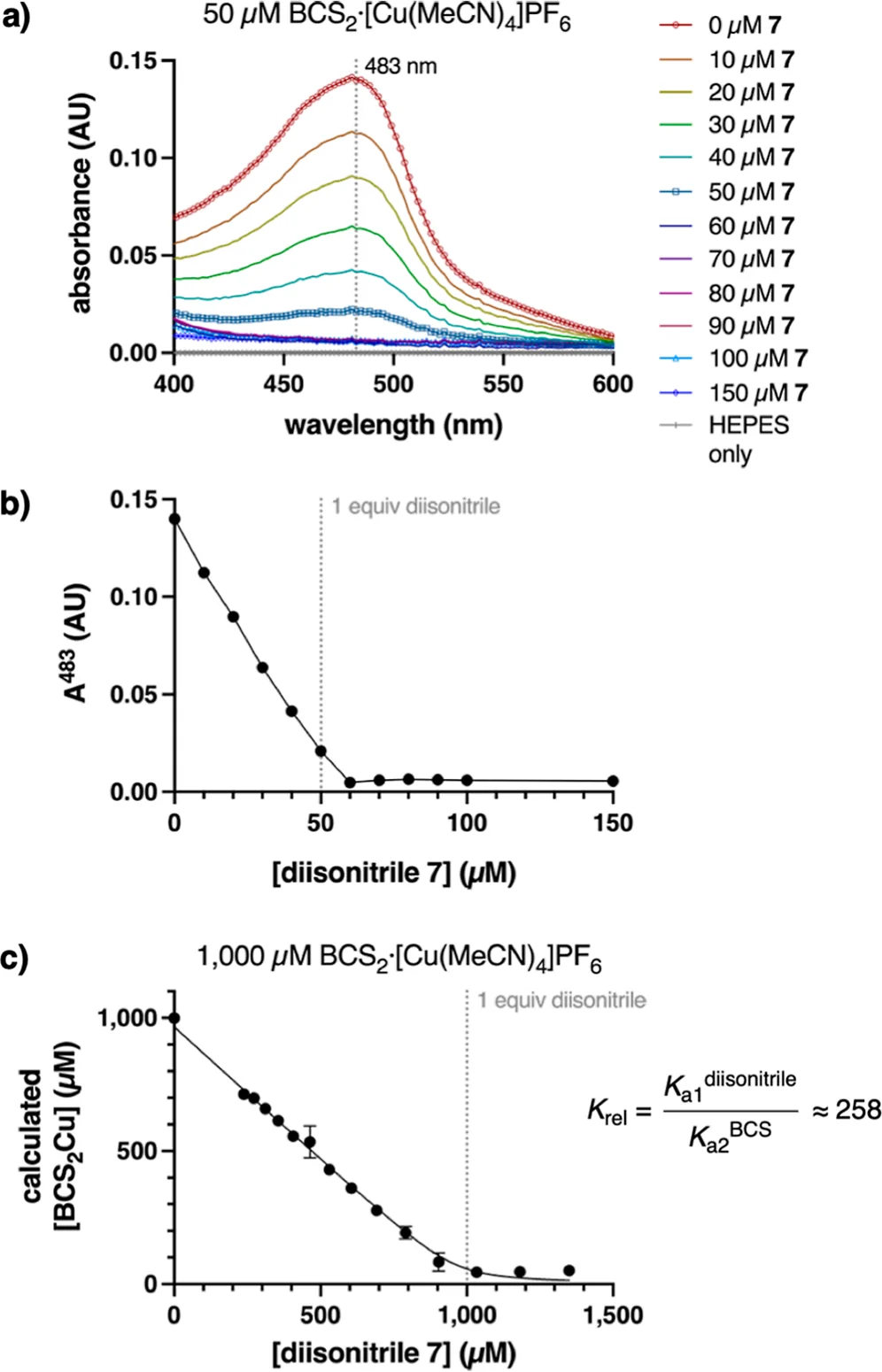
Metal binding of diisonitrile analogues. (a) UV–vis titration of the preformed BCS_2_·[Cu­(MeCN)_4_]­PF_6_ complex (50 μM) with 0 to 3 equiv of C4-Orn-Phe-OAc (**7**). Stock solutions of 1–10 mM and 15 mM **7** in DMSO were diluted in solutions containing 50 μM BCS_2_·[Cu­(MeCN)_4_]­PF_6_ in 25 mM HEPES buffer (0.1 M NaCl, pH 7.2, MeCN:H_2_O v/v = 1:10) + 1% DMSO to final concentrations of **7** between 0 and 150 μM and then analyzed on a plate reader. (b) Plot of data in panel (a) at 483 nm. (c) UV–vis titration of the preformed BCS_2_·[Cu­(MeCN)_4_]­PF_6_ complex (1000 μM) with C4-Orn-Phe-OAc (**7**). A solution of 135 mM **7** in DMSO was serially diluted by a factor of 1.14, then further diluted in 1000 μM BCS_2_·[Cu­(MeCN)_4_]­PF_6_ in 25 mM HEPES buffer (0.1 M NaCl, pH 7.2, MeCN:H_2_O v/v = 1:10) + 1% DMSO to final concentrations of **7** between 238 and 1350 μM, and then analyzed on a plate reader. [BCS_2_·Cu] calculated using Beer’s law; ε = 8344.3 M^–1^cm^–1^ calculated using an absorbance of 1 mM BCS_2_·[Cu­(MeCN)_4_]­PF_6_ (A^483^ = 2.5033) without **7**.

In our previous studies with C4-Lys-OAc (**5**), it did not appear possible to establish an equilibrium between the diisonitrile and BCS for Cu­(I) binding to calculate a *K*_a_ value, due to the much higher affinity of the diisonitrile (hockey stick-shaped titration).^21^ However, close inspection of the titration with C4-Orn-Phe-OAc (**7**) above (Figure 5b) revealed incomplete competition at 1 equiv of diisonitrile, which should enable determination of binding affinity. Thus, we carried out a second titration at a higher concentration of BCS_2_·Cu and with more data points to evaluate this competition in detail (Figure 5c).

The titration was analyzed based on an equilibrium reaction (eq 1), assuming that the A^483^ reflects the concentration of BCS_2_·Cu­(I), with no absorbance by the mixed complex BCS·Cu(I)·**7**. The derived equilibrium equation reflects the ratio (*K*_rel_) of the first association constant for the diisonitrile (*K*_a1_^D^) to the second association constant for BCS (*K*_a2_^BCS^) (eq 2). The first association constant for BCS·Cu­(II) (log *K*_a1_ = 6.1) and the overall two-step association constant for BCS_2_·Cu­(II) (log β_2_ = 11.9) are known^32^ and have been used in conjunction with the reduction potentials for free Cu­(II)/Cu­(I) (+0.153 V) and doubly bound [BCS_2_·Cu­(II)·OH_2_]^2–^/[BCS_2_·Cu­(I)]^3–^ (+0.620 V)^33^ to estimate the corresponding overall two-step association constant for BCS_2_·Cu­(I) (log β_2_ = 19.8) via the Nernst equation.^32^ However, because the reduction potential for singly bound [BCS·Cu­(II)·L_n_]/[BCS·Cu­(I)·L_n_] is not known, it is not possible to estimate the second association constant for BCS_2_·Cu­(I) (*K*_a2_^BCS^) using the analogous approach. Thus, we were only able to determine the relative binding affinity (*K*_rel_) of the diisonitrile (*K*_a1_^D^) compared to BCS (*K*_a2_^BCS^) for Cu­(I). Substitution of BCS, diisonitrile, and mixed complex concentrations with measurable variables then led to a complex equation (Supporting Information Figure S8). Fitting the titration data to the equation yielded a *K*_rel_ of 258 (*R* ^2^ = 0.992), indicating that the diisonitrile binds over 2 logs more tightly than BCS. The two BCS association constants for Cu­(II) are comparable (log *K*_a1_^BCS^ = 6.1, log *K*_a2_^BCS^ = 5.8);^32^ if one assumes that this is also the case for Cu­(I) (log *K*_a1_^BCS^ ≈ log *K*_a2_^BCS^ ≈ 0.5 * log *β*_2_ ≈ 10), then this would imply that log *K*_a1_^D^ ≈ 12.

$${BCS}_{2}\cdot Cu+D\begin{array}{l} {K_{1}}^{D}\\ \;⇌\;\\ {K_{a2}}^{BCS} \end{array}BCS\cdot Cu\cdot D+BCS$$ 1

$$K_{rel}=\frac{{K_{a1}}^{D}}{{K_{a2}}^{BCS}}=\frac{\left[BCS\cdot Cu\cdot D\right]\times \left[{BCS}_{free}\right]}{\left[{BCS}_{2}\cdot Cu\right]\times \left[D_{free}\right]}$$ 2

To date, determination of diisonitrile-binding affinities for metals has been challenging due to their high affinities compared to most other ligands and, in the case of BCS competition experiments, unknown values for the first and second association constants (*K*_a1_, *K*_a2_) with Cu­(I). As a result, affinities reported in the literature vary greatly. Chang and co-workers have estimated a single-ligand dissociation constant for C14-Orn-Phe-OH (**23f**) of only 3 mM (log *K*_a1_ ≈2.5) based on partial complexation to Cu­(I) observed by ^1^H NMR.^22^ In contrast, tighter binding constants of 75 μM (log *K*_a1_ ≈4.1) were estimated for the C4-Lys-OH (**6**) and C4-Lys-OAc (**5**) congeners. However, both of those experiments are based on single concentration data points and were also conducted in DMSO-*d*_6_. In contrast, the UV–vis experiments herein were carried out in aqueous buffer (25 mM HEPES, 0.1 M NaCl, pH 7.2, MeCN:H_2_O v/v = 1:10; 1% DMSO), conditions that are more likely to be physiologically relevant.

Aldrich and co-workers have reported apparent dissociation constants for C12-Orn-Phe-OH (**23e**) binding to Cu­(I) (*K*_d_ = 3.0 nM; equivalent to log *K*_a1_ = 8.5) or Cu­(II) (*K*_d_ = 710 nM; log *K*_a1_ = 6.1) in micelles, based on competition experiments with Phen Green SK (PGSK).^19^ These experiments were also conducted at a single concentration and assume that fluorescence of the reported 1:1 PGSK·Cu­(I) complex (*K*_d_ = 4.2 pM; log *K*_a_ = 11.4) is competed directly and completely by formation of a 1:1 diisonitrile·Cu­(I) complex, without formation of a mixed 1:1:1 PGSK·Cu·diisonitrile complex or formation of the expected final 2:1 diisonitrile_2_·Cu­(I) complex. Notably, the longer-chain C18-Orn-Phe-OH (**23g**) had ≈1 log weaker binding to both Cu­(I) (*K*_d_ = 56 nM; log *K*_a1_ = 7.3) and Cu­(II) (*K*_d_ = 2.1 μM; log *K*_a1_ = 5.7). Thus, the higher association constant that we estimated herein for C4-Orn-Phe-OAc (**7**) (log *K*_a1_^D^ ≈12) is consistent with this general trend that diisonitriles with shorter acyl chains have higher affinities for Cu­(I).

### *M. tuberculosis* Growth Rescue by Diisonitrile Analogues under Low-Copper Conditions

Diisonitriles have been implicated in protecting cellular respiration in *M. tuberculosis* and contributing to bacterial virulence.^6^ Recently, we showed that *M. tuberculosis* diisonitriles are required to maintain the function of the copper-dependent *bcc:aa*_3_ primary respiratory oxidase under copper-restricted conditions.^6^ However, the structural motifs that are responsible for interacting with this copper acquisition system remain poorly understood. To investigate this, we tested diisonitrile analogues **5, 7, 22b–f, 23a,** and **24–28** for their ability to rescue bacterial growth and *bcc*:*aa*_3_ oxidase function in copper-restricted conditions, a function that is critical for *M. tuberculosis* virulence.^34^ We have previously shown that C4-Lys-OAc (**5**) and C4-Orn-Phe-OAc (**7**) can rescue the growth in copper-restricted media of *M. tuberculosis* and *M. marinum* diisonitrile biosynthesis mutants lacking *nrp (rv0101* and *mmaA,* respectively*)*, which encodes the nonribosomal peptide synthetase that assembles the diisonitrile backbone, or *fadD10* (*rv0099* and *mmaC*, respectively), which encodes the adenylation enzyme that activates side chains for coupling to the backbone.^6,10^ In *M. tuberculosis*, this growth defect is further exacerbated by the loss of *cydAB*, which encodes CytBD, a heme-only alternative respiratory oxidase that compensates for loss of the chalkophore-dependent primary heme:Cu respiratory oxidase *bcc:aa*_3_ under copper-restricted conditions.^6^

Thus, *M. tuberculosis* Δ*nrp*Δ*cydAB* growth was assessed in the presence of the copper chelator tetrathiomolybdate (TTM), which we have previously shown phenocopies growth defects observed in Chelex-treated media in which all metals except copper have been reintroduced.^6,10^ Both C4-Orn-Phe-OAc (**7**) and C4-Orn-Phe-OH (**23a**) rescued growth completely at sub-μM concentrations (Figure 6a), indicating that both the C-terminal acetate and alcohol could be accepted; all subsequent studies were carried out with the corresponding acetate analogues. Intracellular hydration of the isonitrile motifs to the corresponding formamides, possibly catalyzed by an isonitrile hydratase, has been postulated as a mechanism for copper release from diisonitriles.^12,18,35^ Consistent with this hypothesis, the C4-bisformamide analogue **28** was ineffective at rescuing growth under these conditions.

**Figure 6:**
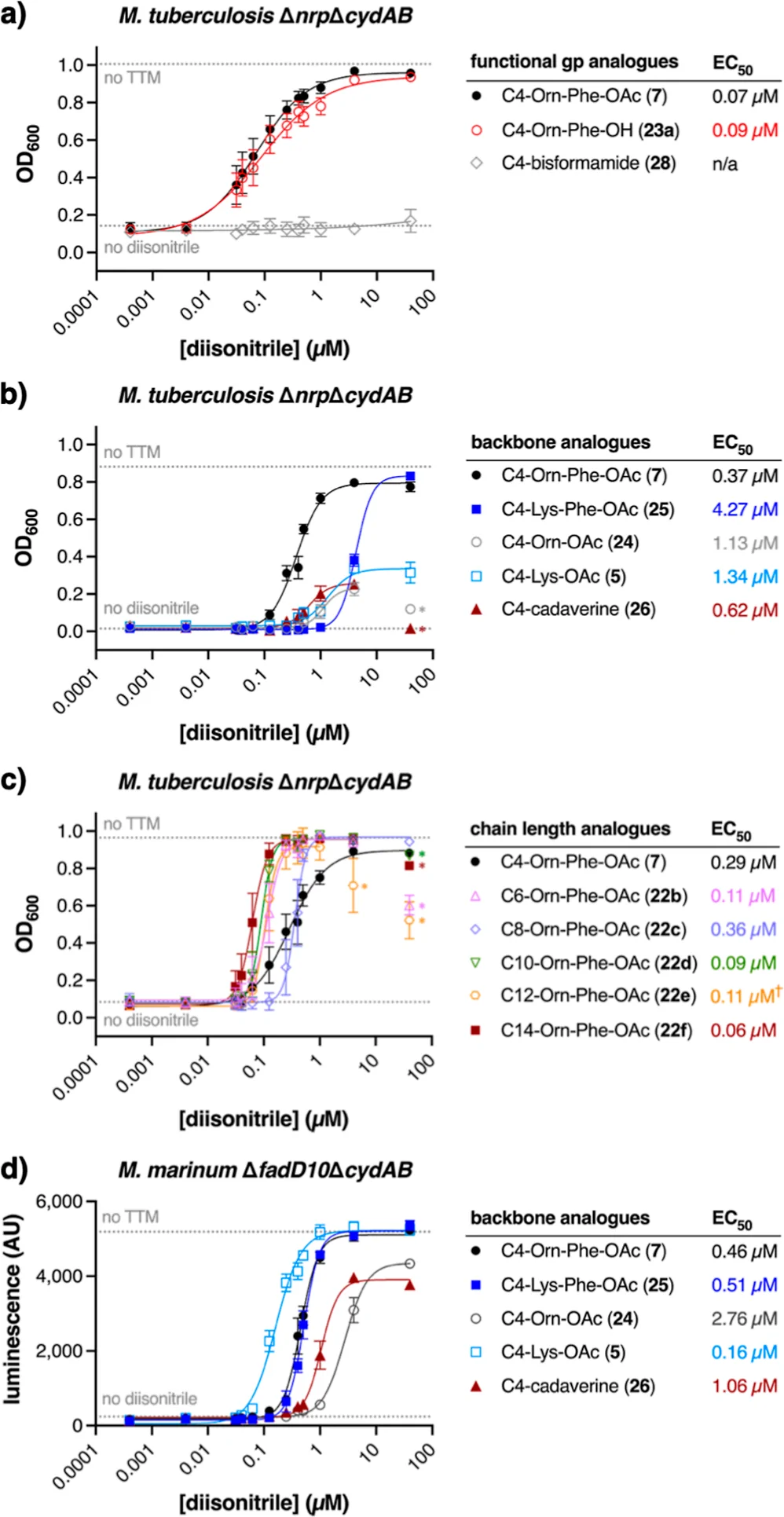
Rescue of mycobacterial mutant growth in copper-restricted media by diisonitrile analogues. SAR for rescue of *M. tuberculosis* Δ*nrp*Δ*cydAB* growth rescue in 20 μM TTM by diisonitrile analogues with (a) C-terminal and side-chain functional group modifications, (b) backbone modifications, and (c) various acyl chain lengths. *Data point excluded from curve fit due to hook effect. ^†^Data for C12 analogue **22e** from a separate experiment in which positive control **7** had a similar EC_50_; OD_600_ is range-normalized based on controls (see Supporting Information Figure S9 for original data). (d) SAR for *M. marinum* Δ*fadD10*Δ*cydAB* growth rescue in 20 μM TTM by diisonitrile analogues with backbone modifications. Assays were carried out in 7H9-ADS (albumin, dextrose, saline) media with 0.5% glycerol, 0.05% Tween-80, prepared as previously described,^10^ with growth recorded on Day 14 (panels a–c) or Day 6 (panel d). Data represent mean ± SEM of *n* = 3 independent biological experiments, each with *n* = 2 technical replicates.

To evaluate SAR of the backbone region, we tested diisonitrile analogues having Orn and Lys cores (**7**, **25**), with and without the C-terminal phenylalaninol motif found in the *M. tuberculosis* natural products (**5**, **24**), as well as a cadaverine analogue (**26**) lacking the C-terminus altogether (Figure 6b). Growth was rescued more potently with the ornithine analogue C4-Orn-Phe-OAc (**7**) compared to the corresponding lysine analogue C4-Lys-Phe-OAc (**25**), consistent with the presence of Orn in the *M. tuberculosis* natural products. In contrast, analogues lacking the C-terminal phenylalaninol motif (**5**, **24, 26**) rescued growth incompletely and exhibited a hook effect with reduced growth at higher concentrations, possibly due to cytotoxicity of these diisonitriles at these concentrations.

Next, to evaluate SAR of acyl chain lengths, we tested C4- to C14-Orn-Phe-OAc (**7**, **22b–f**) (Figure 6c, Supporting Information Figure S9). The C14 analogue (**22f**) rescued growth most effectively, while the C10 and C12 analogues (**22d**, **22e**) exhibited slightly lower activity. Meanwhile, the shorter-chain analogues (**7**, **22b,c**) trended toward less effective rescue. These data indicate that acyl chain length does impact effectiveness of growth rescue and are generally consistent with previous biochemical studies of the *M. tuberculosis* biosynthetic enzyme FadD10 suggesting chain length preferences for C12 and C14 acids,^17,36^ as well as chain lengths of C13 to C19 reported for the *M. tuberculosis* natural products.^12^ Notably, the diastereomeric (*S*,*S*)-C4-Orn-Phe-OAc analogue (**27**) also rescued growth but with ≈6-fold lower potency compared to the (*R*,*R*)-diastereomer (Supporting Information Figure S10).

### *M. marinum* Growth Rescue by Diisonitrile Analogues under Low-Copper Conditions

*M. marinum* is a slow-growing, pathogenic mycobacterium that contains a diisonitrile biosynthetic gene cluster related to that found in *M. tuberculosis*. Although the structures of the *M. marinum* natural products have not been identified definitively, biochemical analysis of the nonribosomal peptide synthetase, MmaA, indicates a preference for Lys over Orn, and heterologous expression experiments with the FadD10 homologue, MmaC, indicate that at least chain lengths of C10 and C12 are accepted.^20^ Notably, previous studies of *Streptomyces* diisonitrile chalkophore function in intracellular copper accumulation indicated species specificity of the diisonitriles.^20^ Thus, to compare SAR in *M. marinum*, we tested the same backbone and C-terminally modified diisonitrile analogues for rescue of *M. marinum* Δ*fadD10*Δ*cydAB* growth in TTM-chelated media (Figure 6d). The diisonitrile analogue C4-Lys-OAc (**5**) rescued growth completely with sub-μM potency, while C4-Orn-OAc (**24**) was 17-fold less potent and afforded incomplete rescue at the highest concentration tested (40 μM). Analogues bearing the C-terminal phenylalaninol motif (**7**, **25**) and the cadaverine analogue lacking the C-terminus (**26**) were also less effective. Taken together, these results are consistent with a preference by the *M. marinum* copper acquisition system for diisonitriles with a Lys rather than an Orn backbone and lacking the C-terminal phenylalaninol residue, in contrast to the SAR observed for *M. tuberculosis* above.

## Conclusion

In conclusion, we have synthesized a series of *M. tuberculosis* diisonitrile chalkophore analogues and evaluated their metal-binding properties and SAR for the rescue of mycobacterial growth under copper-restricted conditions that mimic the host environment during infection. We developed a modular synthetic route that features stereoselective installation of a β-isonitrile moiety on acyl chains of up to 14 carbons in length via Reformatsky reaction with a *t*-butylsulfinimine. We have confirmed that the *M. tuberculosis* diisonitrile analogue C4-Orn-Phe-OAc (**7**) binds selectively to Cu­(I) and Cu­(II) over Zn­(II) based on NMR titrations, consistent with previous reports on other diisonitrile chalkophores.^21,22^ Furthermore, while this diisonitrile binds to both Cu­(I) and Cu­(II), complexation alone does not reduce Cu­(II) to Cu­(I) in situ as shown by ^63^Cu-NMR, although it remains possible that diisonitrile binding may facilitate reduction of the metal center under certain conditions, and that this may be physiologically relevant. We have also shown that the relative binding affinity of C4-Orn-Phe-OAc (**7**) with Cu­(I) (*K*_a1_^D^) is approximately 2 logs greater than that of BCS (*K*_a2_^BCS^). This tight binding may provide insight into the physiologic source of copper that is presumably captured by these diisonitriles, based on relative binding affinities.^37^ While this source of copper is currently unknown, we suspect that diisonitriles may chelate free copper or compete for copper in abundant cuproenzymes from the host cell.

In assays for diisonitrile rescue of mycobacterial growth under copper-restricted conditions, we observed species-specific preferences for the diisonitrile backbone and C-terminal structure, with *M. tuberculosis* preferring an ornithine backbone with the C-terminal phenylalaninol extension and *M. marinum* preferring a lysine backbone without the extension, consistent with the reported and predicted structures of the diisonitrile natural products, respectively.^12,20^ Such species specificity has been demonstrated previously by He and co-workers,^20^ who have shown that intracellular copper concentrations in *Streptomyces thioluteus* can be increased by treatment with the corresponding natural product SF2768 (**4**) but not C4-Lys-OAc (**5**) and C4-Lys-OH (**6**) analogues. Notably, in *M. tuberculosis*, a C-terminal phenylalaninol motif (**7**, **25**) was required for complete growth rescue, whereas *M. marinum* did not exhibit clear trends with respect to such C-terminal modifications.

Interestingly, acyl chain length had a relatively limited impact on *M. tuberculosis* growth rescue in these assays, spanning only a 6-fold range of EC_50_ values, although it is possible that chain length may have greater impacts in vivo.^19^ Compared to (*R*,*R*)-C4-Orn-Phe-OAc (**7**), which has the presumed natural *R* side-chain configuration,^20,21^ the diastereomeric (*S*,*S)*-C4-Orn-Phe-OAc analogue (**27**) exhibited 6-fold weaker potency, consistent with the hypothesis that diisonitrile function is protein-mediated. Further, the fact that related formamides do not bind copper^35^ or rescue growth is also consistent with previous reports that metal release from diisonitriles is driven enzymatically by isonitrile hydratase-catalyzed hydration of the isonitrile motifs.^18,35^ Nonetheless, in our studies of *M. tuberculosis* growth rescue herein, all modifications to the diisonitrile backbone, C-terminus, and side chains were tolerated to varying degrees, suggesting some plasticity in the mycobacterial cellular machinery that mediates diisonitrile–Cu uptake and processing. It is possible that this may be due simply to the biochemical tolerances of the cellular machinery involved in diisonitrile uptake and processing or due to some underlying competitive or evolutionary reason in the bacteria. It is also important to note that the experiments herein do not differentiate between structural changes that impact bacterial recognition of the diisonitrile–Cu complexes and those that alter copper-binding affinity directly. Thus, it is also possible that diisonitrile–Cu complexes enter mycobacteria via a passive process, resulting in this apparent structural plasticity. Addressing these outstanding questions will be the subject of future studies, and the SAR established herein will now enable the design of diisonitrile analogues as chemical probes to identify the putative proteins and cellular machinery that are involved in bacterial copper homeostasis.

## Supplementary Material



## Notes

Supplementary figures, materials and methods, experimental protocols and analytical data, and ^1^H and ^13^C NMR spectra (PDF)
