# Supplementary material for: Diisonitrile Chalkophore Structure and Function in Mycobacterial Copper Acquisition
Source: J Am Chem Soc. 2026 Oct 1;148(40):43041–9. doi: 10.1021/jacs.6c09506 (PMC13674278; doi:10.1021/jacs.6c09506)

Diisonitrile Chalkophore Structure and Function in Mycobacterial Copper Acquisition

Pooja B. Pandya,[†,§] Chad E. Hatch,[§] John A. Buglino,[£] Yaprak Ozakman,[£] Kate Ryan,[‡,§] Ruofei Faye Li,[∞,§] Farhan Chowdhury,[§] Michael S. Glickman,[£,‡] and Derek S. Tan*,[†,§,‡,¶]

[†]*Pharmacology Program, Weill Cornell Graduate School of Medical Sciences,*
[§]*Chemical Biology Program, Sloan Kettering Institute*
[£]*Immunology Program, Sloan Kettering Institute*
[‡]*Gerstner Sloan Kettering Graduate Program, Gerstner Sloan Kettering Graduate School,*
[∞]*Mechanistic Biology Summer Program, Gerstner Sloan Kettering Graduate School,*
and [¶]*Tri-Institutional Research Program*

Memorial Sloan Kettering Cancer Center
1275 York Avenue, New York, New York 10065

Supporting Information

A. Supplementary Figures S1–S10 S2
B. Materials and Methods S9
C. Synthesis of Diisonitrile Sidechains **15b–f** S11
D. Synthesis of Diisonitriles **7, 22b–f, 23a,f** S25
E. Synthesis of C4-Orn-OAc (**24**) S33
F. Synthesis of C4-Lys-Phe-OAc (**25**) S36
G. Synthesis of C4-Cadaverine (**26**) S39
H. Synthesis of *S,S*-C4-Orn-Phe-OAc (**27**) S40
I. Synthesis of C4-bisformamide (**28**) S43
J. UV-Vis Competition Titrations of **7** with $Cu(BCS)_2$ S44
K. Mycobacterial Growth Assays S45
L. ^{1}H-NMR and ^{13}C-NMR Spectra S46

A. SUPPLEMENTARY FIGURES S1–S10

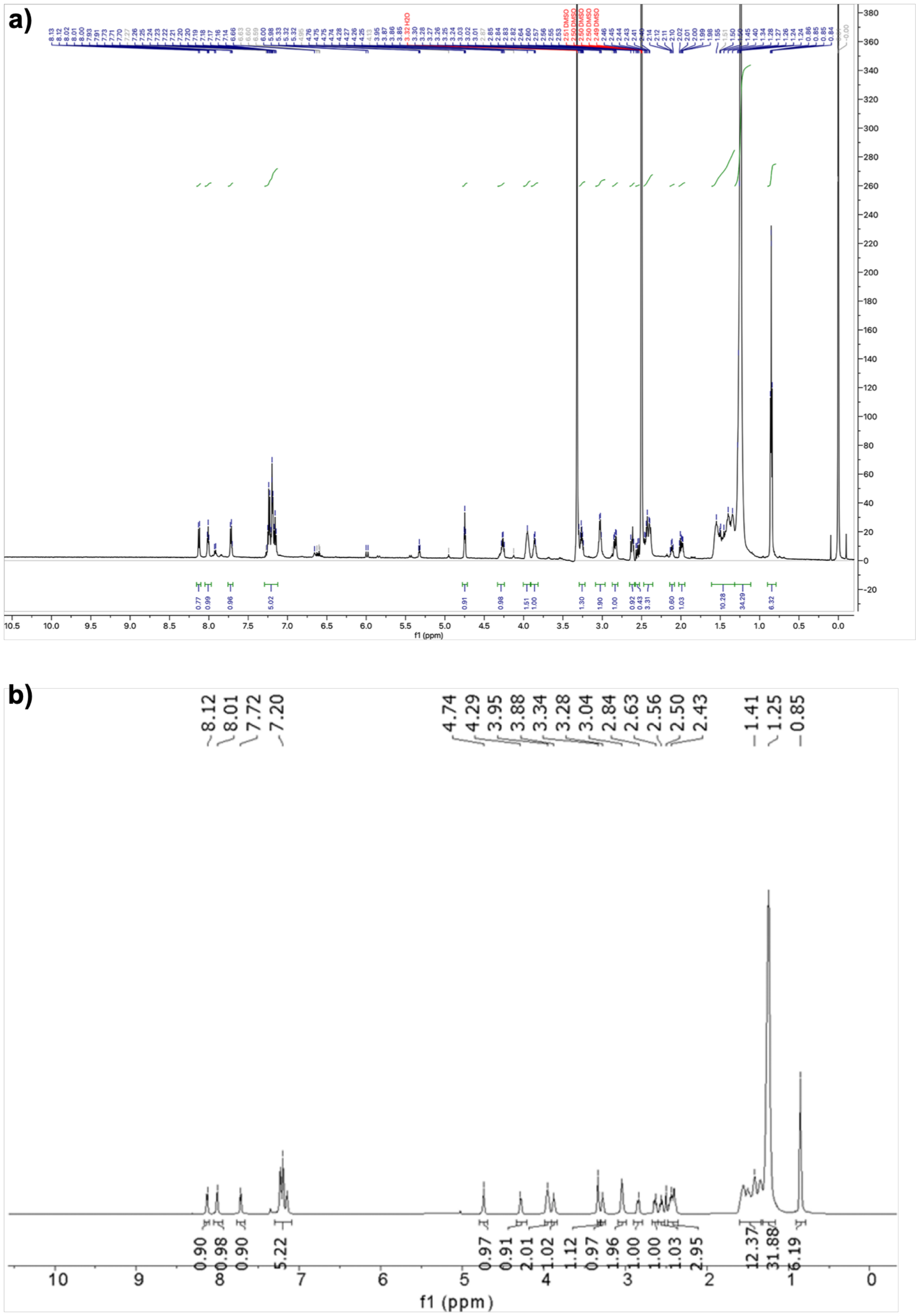

Supplementary Figure S1. Comparison of ¹H-NMR spectra for synthetic C14-Orn-Phe-OH (23f) in DMSO-*d*₆. (a) Material synthesized herein. **(b)** As reported previously by Chang and coworkers.[1]

[1] Chen, T.-Y.; Chen, J.; Ruszczycky, M. W.; Hilovsky, D.; Hostetler, T.; Liu, X.; Zhou, J.; Chang, W.-c.. *ACS Catal.* **2024**, *14*, 4975–4983.

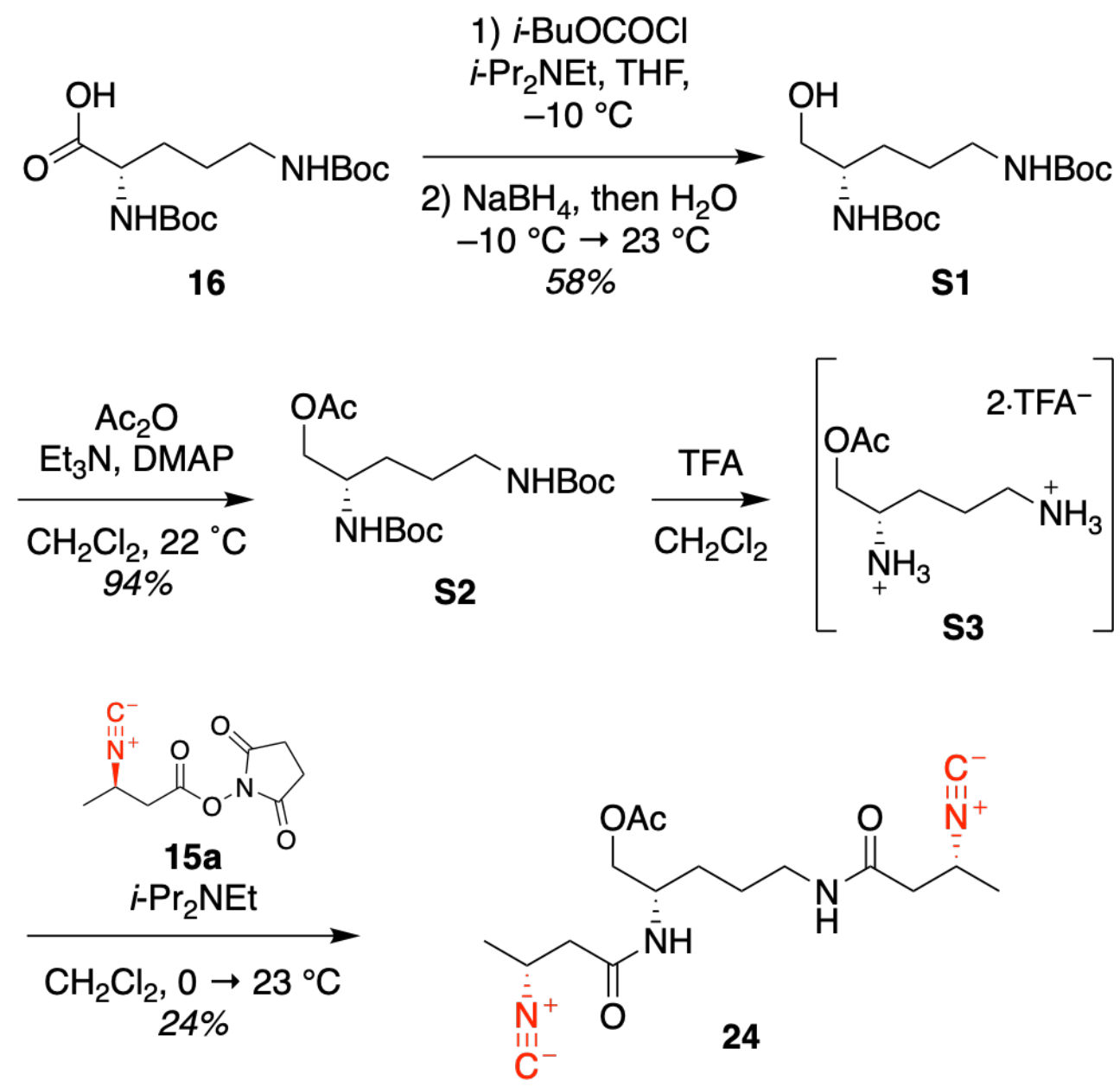

Supplementary Figure S2. Synthesis of C4-Orn-OAc (24). Boc = *t*-butoxycarbonyl; DMAP = 4-(dimethylamino)pyridine; TFA = trifluoroacetic acid; THF = tetrahydrofuran.

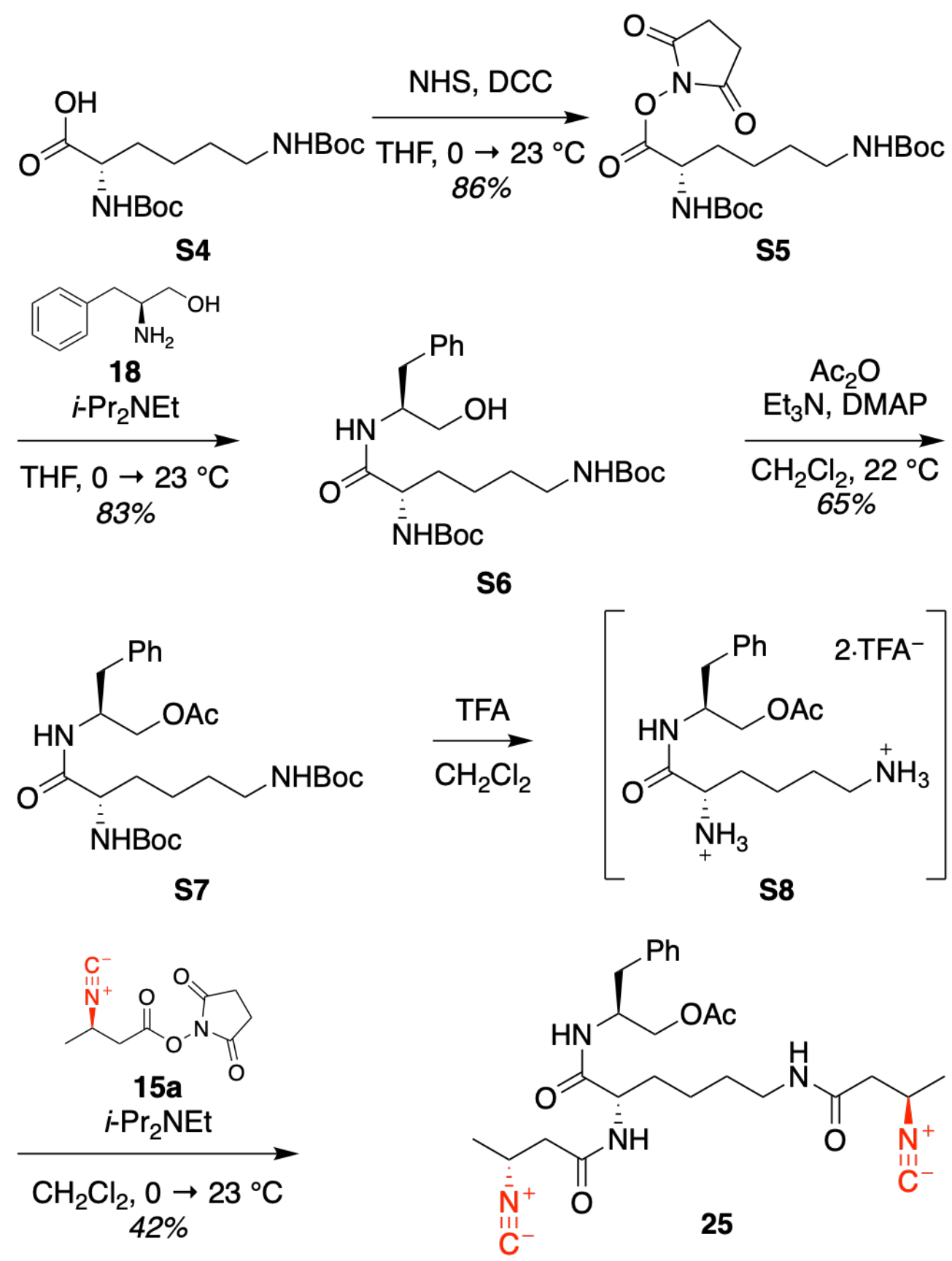

Supplementary Figure S3. Synthesis of C4-Lys-Phe-OAc (25). Boc = *t*-butyloxycarbonyl; DCC = *N,N*′-dicyclohexylcarbodiimide; DMAP = 4-(dimethylamino)pyridine; NHS = *N*-hydroxysuccinimide; TFA = trifluoroacetic acid; THF = tetrahydrofuran.

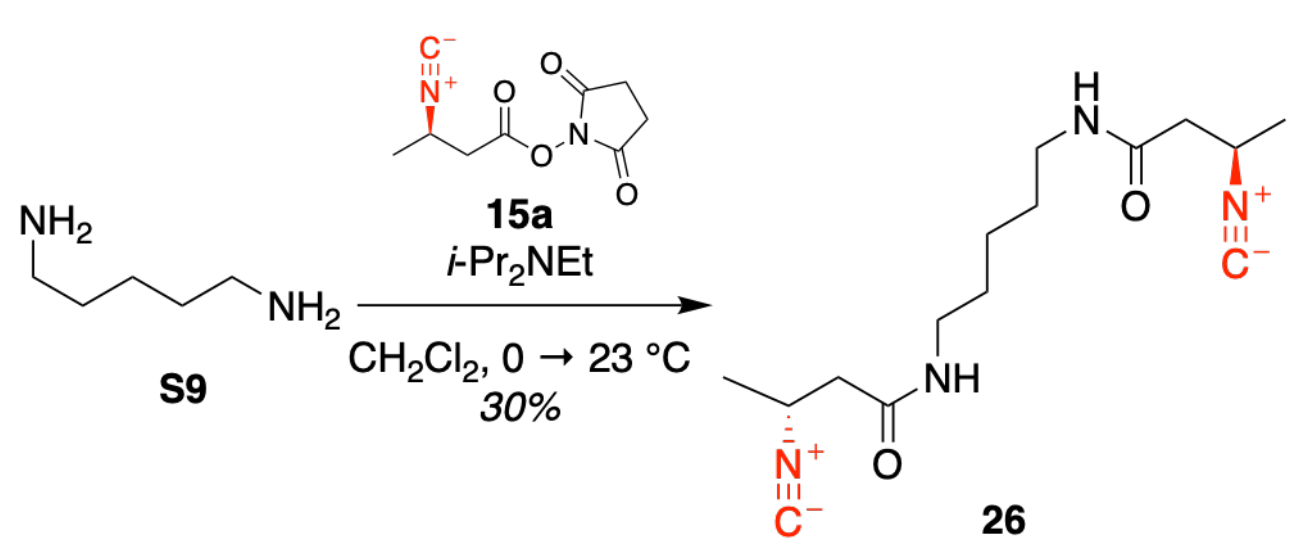

Supplementary Figure S4. Synthesis of C4-cadaverine analogue (26).

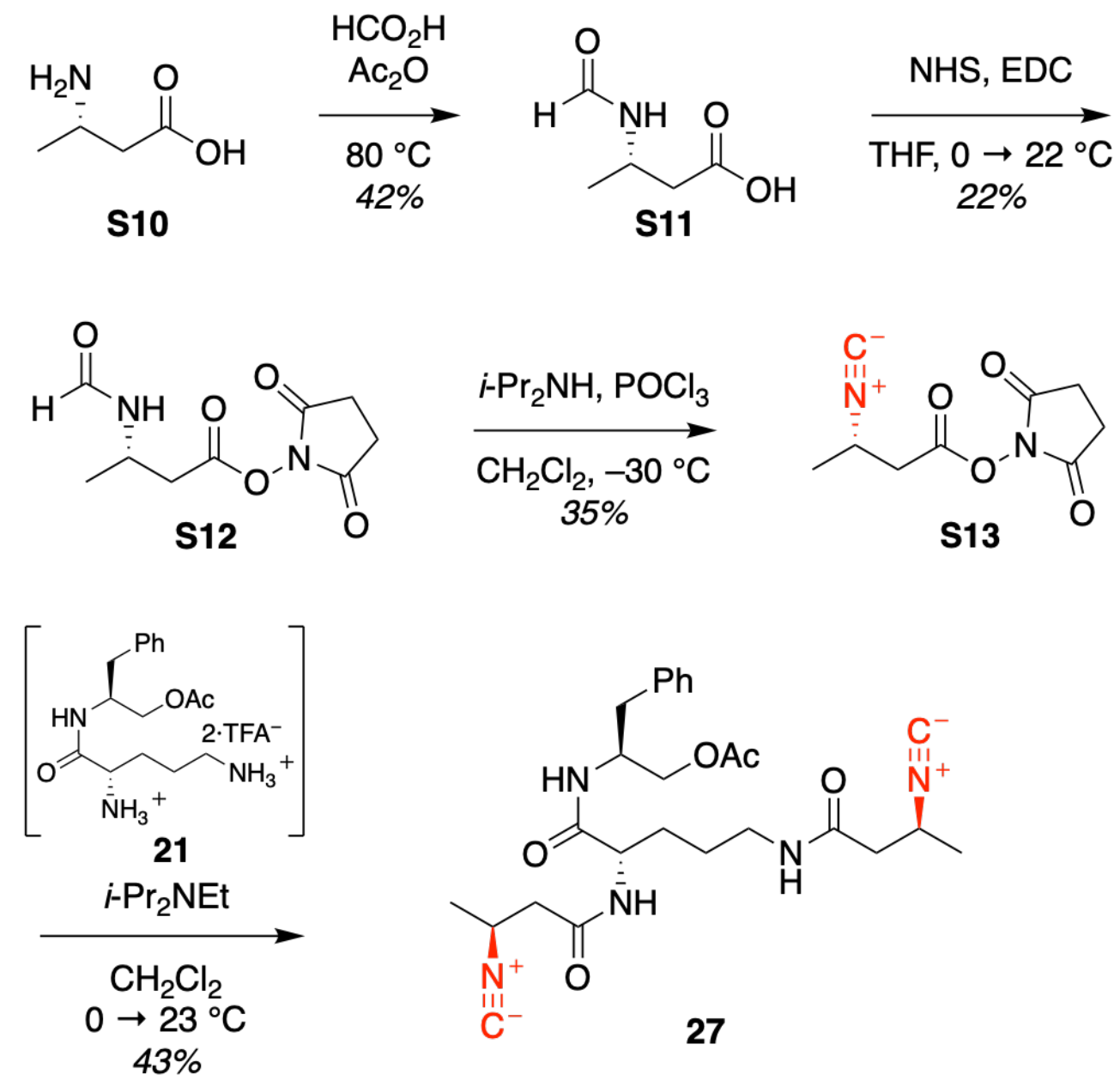

Supplementary Figure S5. Synthesis of (*S,S*)-C4-Orn-Phe-OAc (27). DCC = *N*,*N*´-dicyclohexylcarbodiimide ; NHS = *N*-hydroxysuccinimide; THF = tetrahydrofuran.

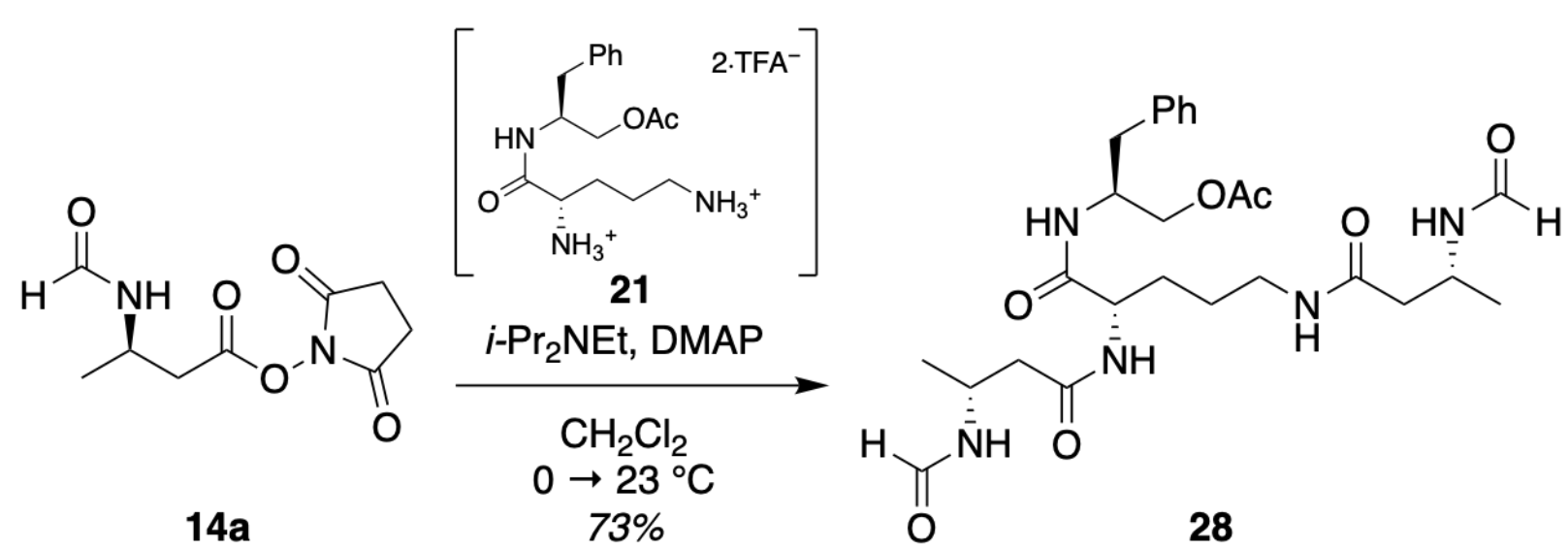

Supplementary Figure S6. Synthesis of (*R,R*)-C4-bisformamide (28). DMAP = 4-(dimethylamino)-pyridine.

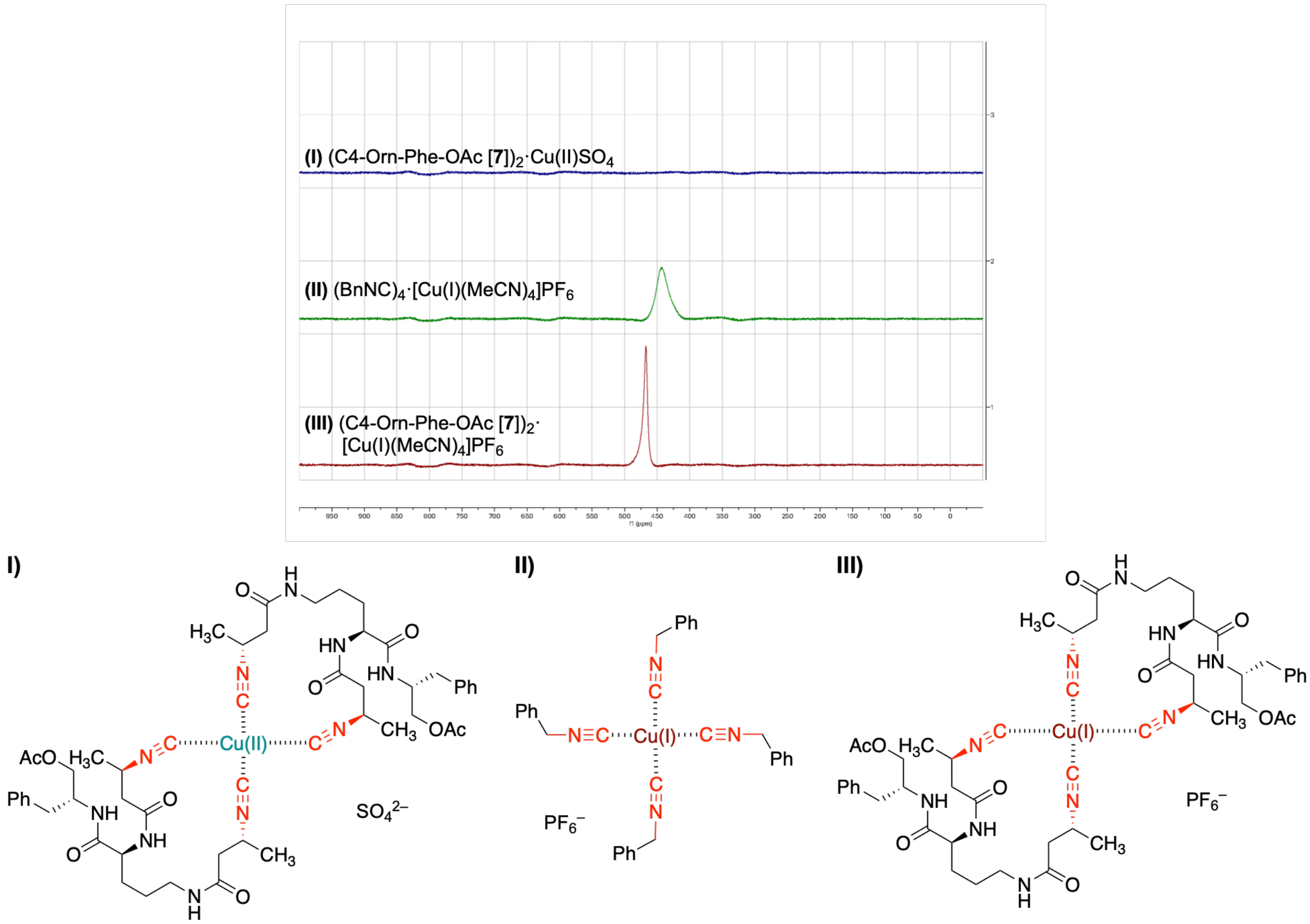

Supplementary Figure S7. ^{63}Cu-NMR analysis indicates that diisonitrile chelation alone does not cause reduction of Cu(II) to Cu(I) in CD_3CN. Cu(I) is diamagnetic and can be observed by ^{63}Cu-NMR, while Cu(II) is paramagnetic and generally not observed due to line broadening. ^{63}Cu-NMR recorded at 159.1 MHz, CD_3CN, 24.1 °C. (top) 2:1 mixture of C4-Orn-Phe-OAc (7) with Cu(II)SO_4, showing no signal for Cu(II) or Cu(I). (middle) 4:1 mixture of BnNC with [Cu(I)$(MeCN)_4$]PF_6, showing a reference signal for Cu(I). (bottom) 2:1 mixture of C4-Orn-Phe-OAc (7) with [Cu(I)$(MeCN)_4$]PF_6, showing signal for Cu(I). The Cu(I) complexes are presumed to be tetrahedral while the Cu(II) complex is presumed to be square planar; the structures shown are schematic and are not intended to imply geometry or conformation.

Equilibrium reaction:

$$BCS_2{\cdot}Cu + D \underset{K_2^{BCS}}{\overset{K_1^{D}}{\rightleftharpoons}} BCS{\cdot}Cu{\cdot}D + BCS \qquad (1)$$

Convert to equilibrium equation:

$$K_{rel} = \frac{K_{a1}^{D}}{K_{a2}^{BCS}} = \frac{[BCS{\cdot}Cu{\cdot}D] \times [BCS_{free}]}{[BCS_2{\cdot}Cu] \times [D_{free}]} \qquad (2)$$

Define concentrations for species not measured directly:

$$[BCS_{free}] = [BCS_{tot}] - [BCS{\cdot}Cu{\cdot}D] - 2\,[BCS_2{\cdot}Cu] \qquad (3)$$

$$[D_{free}] = [D_{tot}] - [BCS{\cdot}Cu{\cdot}D] \qquad (4)$$

$$[BCS{\cdot}Cu{\cdot}D] = [Cu_{tot}] - [BCS_2{\cdot}Cu] \qquad (5)$$

Substitute into equilibrium equation:

$$K_{rel} = \frac{([Cu_{tot}] - [BCS_2{\cdot}Cu]) * ([BCS_{tot}] - [Cu_{tot}] + [BCS_2{\cdot}Cu] - 2\,[BCS_2{\cdot}Cu])}{[BCS_2{\cdot}Cu] * ([D_{tot}] - [Cu_{tot}] + [BCS_2{\cdot}Cu])} \qquad (6)$$

Convert measured concentrations into graph coordinates and constants:

for $y = [BCS_2{\cdot}Cu]$; $x = [D_{tot}]$; $b = [BCS_{tot}]$; $c = [Cu_{tot}]$:

$$K_{rel} = \frac{(c - y) * (b - c + y - 2\,y)}{y * (x - c + y)} \qquad (7)$$

Convert to a quadratic equation:

$$K_{rel} * \{y^2 + (x-c)y\} = y^2 - by + bc - c^2 \qquad (8)$$

$$K_{rel}*y^2 + K_{rel}(x-c)y = y^2 - by + bc - c^2 \qquad (9)$$

$$K_{rel}*y^2 + K_{rel}(x-c)y - y^2 + by - bc + c^2 = 0 \qquad (10)$$

$$(K_{rel}-1)y^2 + \{K_{rel}(x-c)+b\}y + c^2 - bc = 0 \qquad (11)$$

Solve using quadratic formula:

$$y = \frac{-\{K_{rel}*(x-c)+b\} \pm \sqrt{\{K_{rel}*(x-c)+b\}^2 - \{4(K_{rel}-1)(c^2-bc)\}}}{2(K_{rel}-1)} \qquad (12)$$

where $b = [BCS_{tot}] = 2{,}500\ \mu M$,
$c = [Cu_{tot}]$ was determined by the curve fit as 966.5 μM,
and K_{rel} was determined by the curve fit as 257.7

Supplementary Figure S8. Diisonitrile titration equations. Derivation of curve fit simplifying the equilibrium reaction to a quadratic equation using measurable terms. $[BCS_2Cu]$ is measured by absorbance; $b = [BCS_{tot}] = 2{,}500\ \mu M$, $c = [Cu_{tot}]$ was determined by the curve fit as 966.5 μM, and K_{rel} was determined by the curve fit as 257.7.

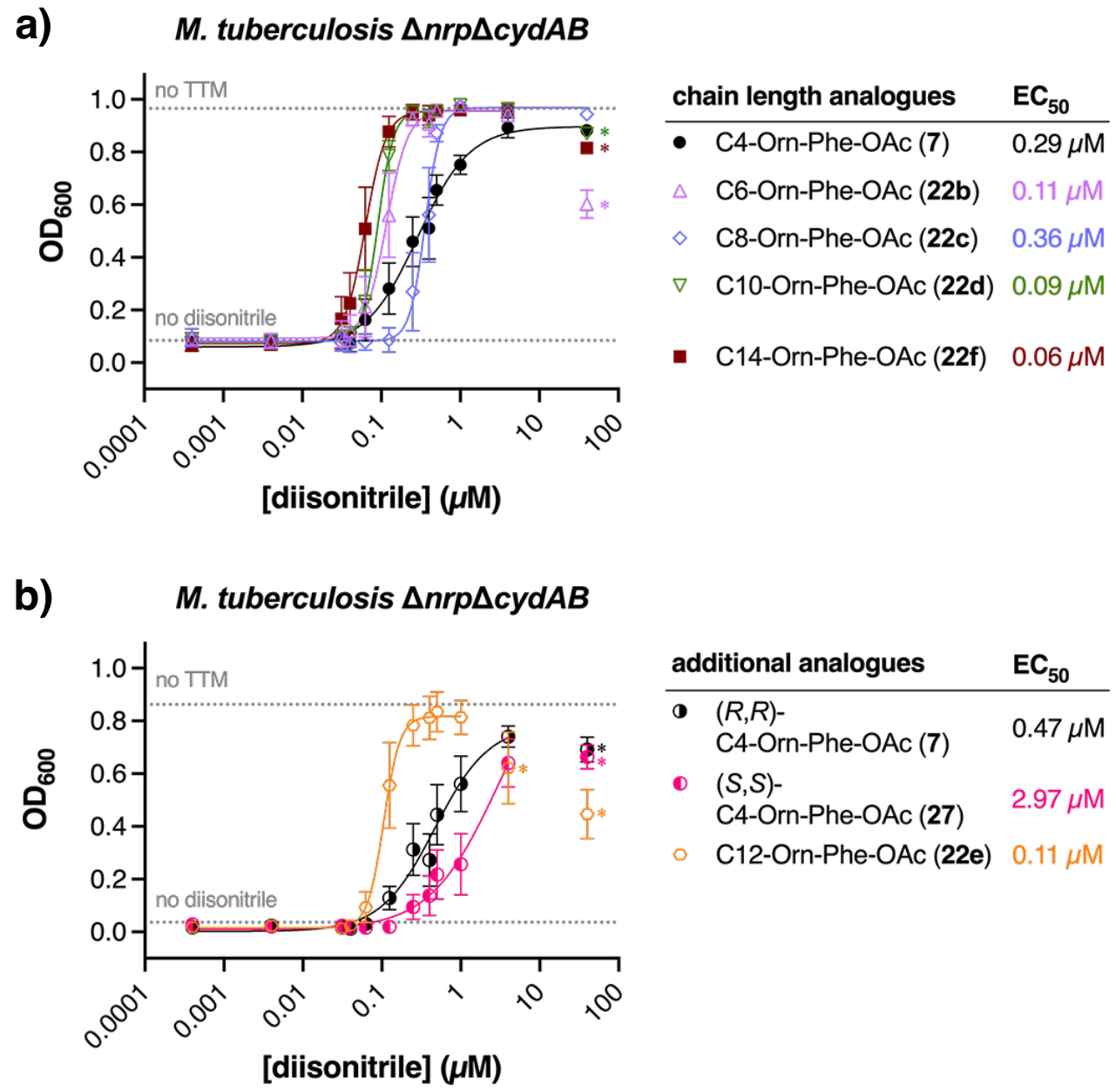

Supplementary Figure S9. Rescue of mycobacterial mutant growth in copper-restricted media by diisonitrile analogues. Original experiments for data combined in **Figure 6c** of the manuscript. The parent analogue C4-Orn-Phe-OAc (**7**) has comparable activity in both assays. Thus, data for C12-Orn-Phe-OAc (**22e**) was combined with the data for the other acyl chain lengths in **Figure 6c** after normalization of the OD_{600} range based on the no-TTM and no-diisonitrile controls.

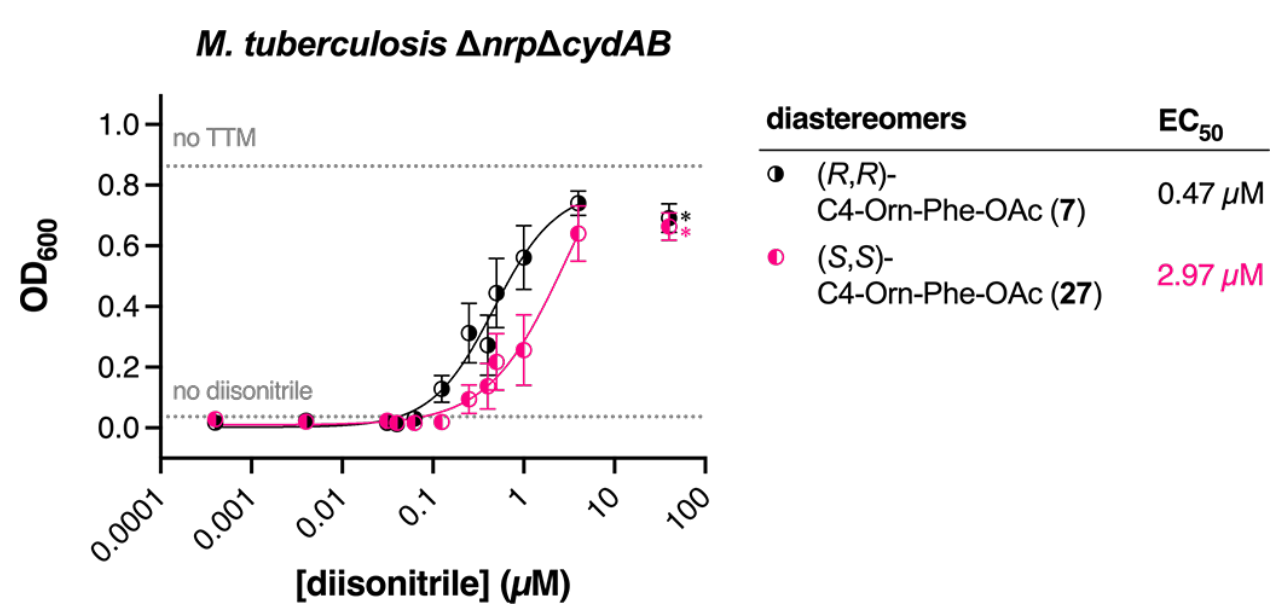

Supplementary Figure S10. Rescue of mycobacterial mutant growth in copper-restricted media by diisonitrile analogues. SAR for rescue of *M. tuberculosis ΔnrpΔcydAB* growth rescue in 20 μM TTM by diisonitrile analogues diastereomeric acyl chains. Assays were carried out in 7H9-ADS (albumin, dextrose, saline) media with 0.5% glycerol, 0.05% Tween-80, prepared as previously described,[2] with growth recorded on Day 14. Data represent mean ± SEM of n = 3 independent biological experiments, each with n = 2 technical replicates.

[2] Buglino, J. A.; Ozakman, Y.; Xu, Y.; Chowdhury, F.; Tan D. S.; Glickman M. S. *mBio* **2022**, *13*, e02513–02522

B. MATERIALS AND METHODS

Reagents

Reagents were obtained from Aldrich Chemical (www.sigma-aldrich.com), Acros Organics (www.fishersci.com), Ambeed (www.ambeed.com), or Oakwood Chemicals (www.oakwoodchemicals.com) and used without further purification. Optima or HPLC grade solvents were obtained from Fisher Scientific (www.fishersci.com), degassed with Ar, and purified on a solvent drying system as described[3] unless otherwise indicated. Diisopropylamine (*i*-Pr_2NH) was obtained from Aldrich in SureSeal bottles.

Reactions

All reactions were performed in flame-dried glassware under positive Ar pressure with magnetic stirring unless otherwise noted. Liquid reagents and solutions were transferred thru rubber septa via syringes flushed with Ar prior to use. Cold baths were generated as follows: 0 °C, wet ice/water; –10 °C, wet ice/brine; –30 °C, cryocool.

Chromatography

TLC was performed on 0.25 mm E. Merck silica gel 60 F254 plates and visualized under UV light (254 nm) or by staining with potassium permanganate ($KMnO_4$), cerium ammonium molybdenate (CAM), or *p*-anisaldehyde. Silica flash chromatography was performed on E. Merck 230–400 mesh silica gel 60. Parallel chromatography was performed on an ISCO CombiFlash OptiX 10 instrument with RediSep silica gel normal phase columns with ELSD detection and with UV detection at 254 nm.

Analytical Instrumentation

Optical rotations were recorded on a JASCO model P-1020 digital polarimeter with P-103T temperature controller. NMR spectra were recorded on a Bruker UltraShield Plus 500 MHz Avance III NMR or UltraShield Plus 600 MHz Avance III NMR with DCH CryoProbe at 24 °C in $CDCl_3$ unless otherwise indicated. Chemical shifts are expressed in ppm relative to TMS (^1H, 0 ppm) or solvent signals: $CDCl_3$ (^1H, 7.26 ppm; ^{13}C, 77.0 ppm), DMSO-d_6 (^1H, 2.50 ppm; ^{13}C, 39.52 ppm), MeOD-d_4 (^1H, 3.31 ppm; ^{13}C, 49.0 ppm); coupling constants are expressed in Hz. NMR spectra were processed using Mnova (www.mestrelab.com/software/mnova-nmr). High resolution mass spectra were obtained at the MSKCC Analytical Core Facility on a Waters Acuity Premiere XE TOF LC-MS by electrospray ionization (ESI). UV-Vis spectra were recorded on a Tecan i-control infinite M1000Pro. Samples were prepared in a Costar 96-well black, clear-bottom multiplate. Data was analyzed using GraphPad Prism 6.

Nomenclature

Atom numbers used in the text of the article and Supporting Information refer to standard amino acid and carboxylic acid numbering systems, and not to IUPAC nomenclature, which was used solely to name each compound. Compounds not cited in the paper are numbered herein from **S1**.

---

[3] Pangborn, A. B.; Giardello, M. A.; Grubbs, R. H.; Rosen, R. K.; Timmers, F. J. *Organometallics* **1996,** *15*, 1518–1520.

Abbreviations for C-terminal motifs of amino acids are as defined in Figure 3 of the manuscript, with -OH representing a reduced C-terminal hydroxymethyl group and not the C-terminal carboxylate as sometimes abbreviated. By extension, -OAc represents a C-terminal acetoxymethyl group.

C. Synthesis of Diisonitrile Sidechains 15b–f

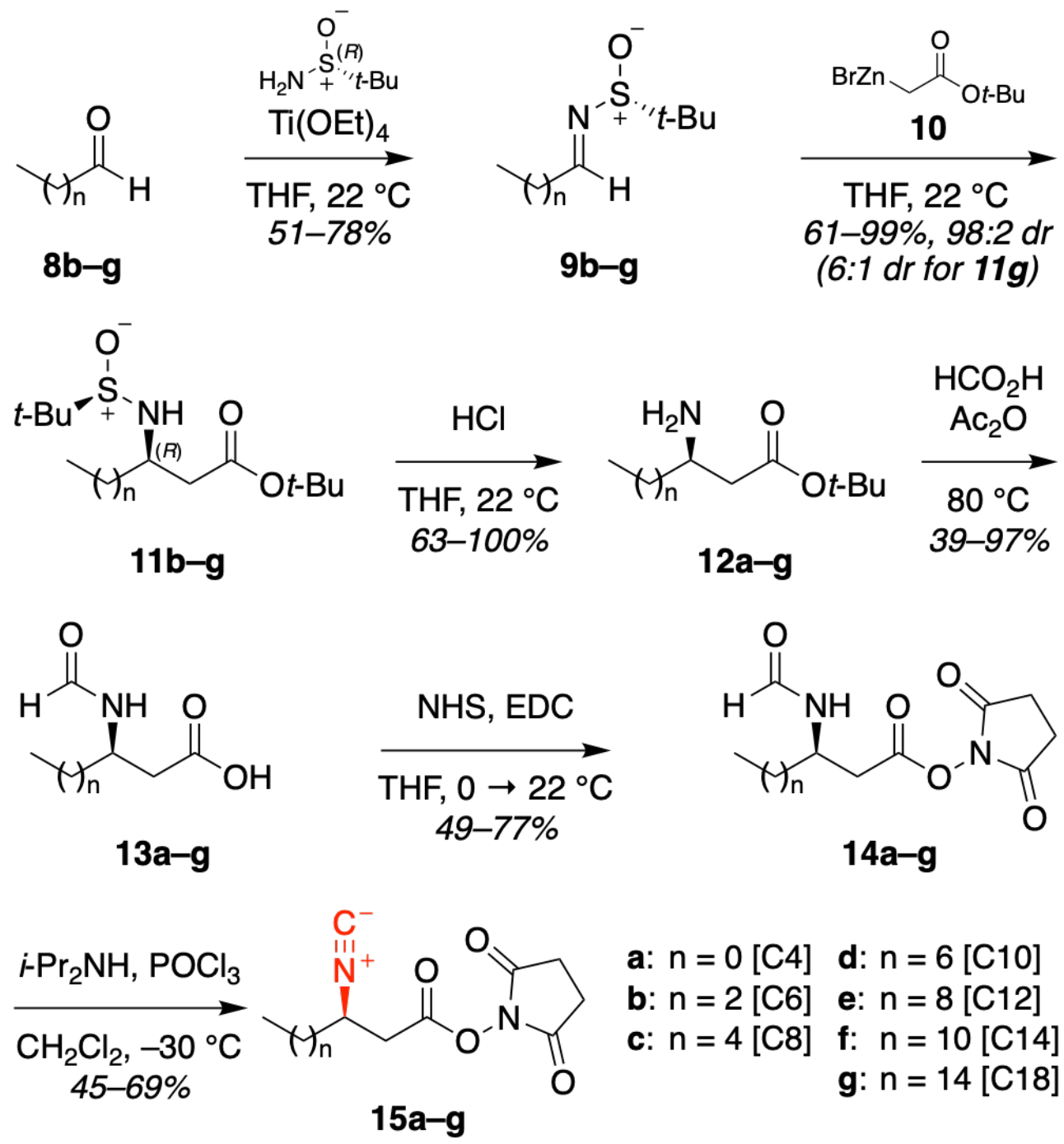

Supplementary Figure S11 ***(Figure 2 in manuscript).*** **Diastereoselective synthesis of (*R*)-β-isocyano-acyl chains.** EDC = 1-ethyl-3-(dimethylaminopropyl)carbodiimide; NHS = *N*-hydroxysuccinimide; THF = tetrahydrofuran.

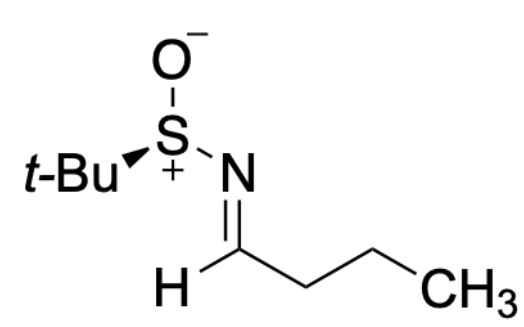

(*R,E*)-*N*-Butylidene-2-methylpropane-2-sulfinamide (9b). In a 250-mL roundbottom flask, butanal (1.25 mL, 13.86 mmol, 1 equiv), (*R*)-2-methylpropane-2-sulfinamide (1.855 g, 15.30 mmol, 1.1 equiv), and titanium(IV) ethoxide (7.3 mL, 34.81 mmol, 2.5 equiv) were dissolved in THF (30 mL, 0.46 M) and stirred for 2 h at rt. The reaction mixture was quenched with brine (20 mL) and filtered over a pad of celite that had been rinsed with EtOAc. The filtrate was extracted with EtOAc (1 x 200 mL). The combined organic extracts were washed with brine (1 x 25 mL), dried (Na_2SO_4), filtered, and concentrated by rotary evaporation to afford the crude sulfinimine **9b** as a yellow oil (2.43 g, quant.), which was used without further purification.

TLC: R_f 0.25 (10:1 hexanes/EtOAc, UV, *p*-anisaldehyde). **^{1}H-NMR** (600 MHz, $CDCl_3$): δ 8.07 (t, J = 4.7 Hz, 1H), 2.50 (td, J = 7.5, 4.9 Hz, 2H), 1.71 – 1.62 (m, 2H), 1.19 (s, 9H), 0.99 (t, J = 7.4 Hz, 3H). **^{13}C-NMR** (151 MHz, $CDCl_3$): δ 169.67, 56.50, 38.07, 22.36, 18.97, 13.82. **HRMS** (ESI) *m/z* calcd for $C_8H_{17}NOS$ ($[M+H]^+$) 176.1109; found 176.1103.

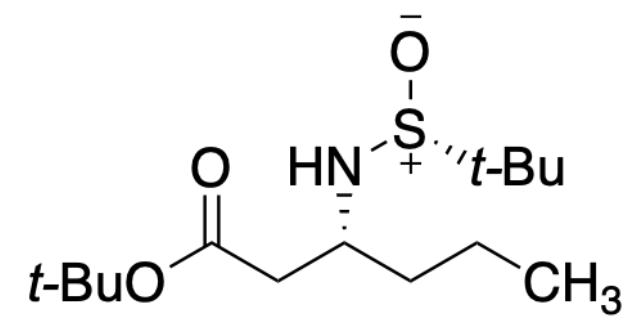

***tert*-Butyl (*R*)-3-(((*R*)-*tert*-butylsulfinyl)amino)hexanoate (11b):** In a 500-mL roundbottom flask, (*R,E*)-*N*-butylidene-2-methylpropane-2-sulfinamide (**9b**) (5.5055 g, 31.40 mmol, 1 equiv) was dissolved in THF (60 mL, 0.53 M). A solution of Reformatsky reagent (**10**) in THF (26.8478 g, 80.70 mmol, 0.5 M, 2.7 equiv) was added to the reaction mixture and stirred for 17.5 h at rt. The reaction mixture was quenched with satd aq NH_4Cl, diluted with H_2O, and extracted with EtOAc (2 x 125 mL). The combined organic extracts were washed with brine, dried (Na_2SO_4), filtered, and concentrated by rotary evaporation to afford the crude product. Purification by silica flash chromatography (30–50% EtOAc/hexanes) yielded β-sulfinamido ester **11b** as a pale yellow oil (9.0269 g, 99%).

TLC: R_f 0.24 (25% EtOAc/hexanes, CAM, *p*-anisaldehyde). $[\alpha]^{22}$: –11.0635° (*c* 0.88, $CHCl_3$). **^{1}H NMR** (600 MHz, $CDCl_3$): δ 4.12 (d, *J* = 8.0 Hz, 1H), 3.49 (tt, *J* = 10.4, 4.3 Hz, 1H), 2.64 (dd, *J* = 15.7, 5.3 Hz, 1H), 2.50 (dd, *J* = 15.8, 5.5 Hz, 1H), 1.61 – 1.50 (m, 1H), 1.44 (d, *J* = 1.2 Hz, 11H), 1.32 (tdd, *J* = 12.9, 5.1, 2.7 Hz, 1H), 1.20 (s, 9H), 0.89 (t, *J* = 7.3 Hz, 3H). **^{13}C NMR** (151 MHz, $CDCl_3$): δ 171.31, 81.13, 55.75, 53.54, 41.68, 37.47, 28.12, 22.70, 19.12, 13.78. **HRMS** (ESI) *m/z* calcd for $C_{14}H_{29}NO_3S$ ($[M+H]^+$) 292.1946; found 292.1946.

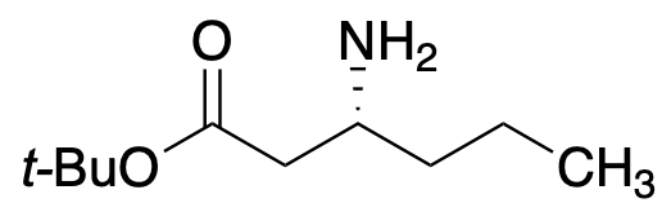

***tert*-Butyl (*3R*)-3-aminohexanoate (12b):** In a 500-mL roundbottom flask, *tert*-butyl (*R*)-3-(((*R*)-*tert*-butylsulfinyl)amino)hexanoate amine (**11b**) (3.4219 g, 11.73 mmol, 1 equiv), THF (90 mL, 0.13 M), and HCl in Et_2O (2.0 M, 60 mL, 120 mmol, 10.2 equiv) were added and stirred for 3 h at rt. The reaction mixture was concentrated by rotary evaporation to afford the crude HCl salt. The resulting HCl salt was dissolved in EtOAc, washed with satd aq $NaHCO_3$ until pH 9, and the aq phase was back-extracted with EtOAc (2 x 50 mL). The combined organic extracts were dried (Na_2SO_4), filtered, and concentrated by rotary evaporation to afford β-amino ester **12b** as a yellow oil (2.1616 g, 98%), which was used without further purification.

TLC: R_f 0.31 (100% EtOAc, ninhydrin).

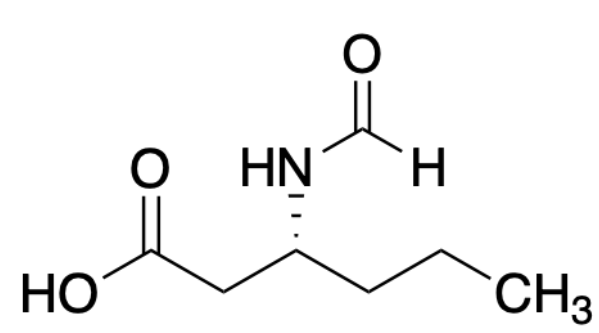

(*3R*)-3-Formamidohexanoic acid (13b): In a 250-mL roundbottom flask containing *tert*-butyl (*3R*)-3-aminohexanoate (**12b**) (3.53 g, 18.84 mmol, 1 equiv), formic acid (40 mL, 1.060 mol, 56 equiv, 3:2 ratio of formic acid to acetic anhydride), and acetic anhydride (27 mL, 285.6 mmol, 15 equiv) were heated together at 80 °C for 20 h. The reaction mixture was concentrated by rotary evaporation to afford the crude product. Purification by silica flash chromatography (50–75% EtOAc/hexanes + 1% AcOH) yielded β-formamido acid (**13b**) as a white powder (1.5064 g, 50%).

TLC: R_f 0.47 (80% EtOAc/hexanes + 1% AcOH, CAM, $KMnO_4$). **^{1}H NMR** (500 MHz, MeOD): δ 8.02 (d, *J* = 5.9 Hz, 1H), 4.31 – 4.22 (m, 1H), 2.48 (d, *J* = 6.5 Hz, 2H), 1.59 – 1.45 (m, 2H), 1.45 – 1.38 (m, 1H), 1.38 – 1.30 (m, 1H), 0.94 (t, *J* = 7.2 Hz, 3H). **^{13}C NMR** (126 MHz, MeOD): δ 174.67, 167.07, 163.25, 51.07, 49.15, 46.27, 41.26, 40.23, 38.18, 37.46, 20.17, 20.16, 14.07, 13.92. **HRMS** (ESI) *m/z* calcd for $C_7H_{12}NO_3$ ($[M+H]^+$) 158.0817; found 158.0820.

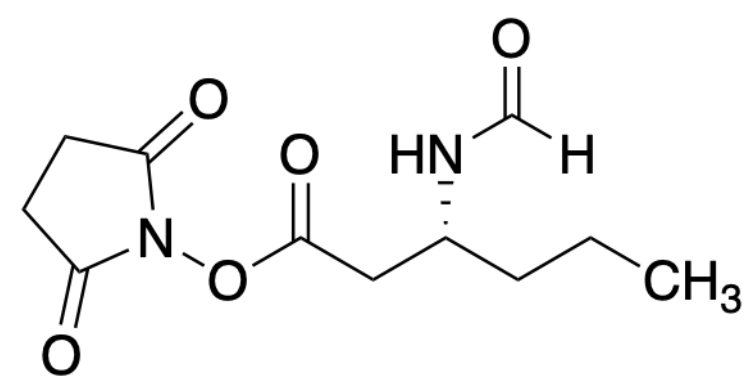

2,5-Dioxopyrrolidin-1-yl (*3R*)-3-formamidohexanoate (14b): In a 25-mL roundbottom flask, (*3R*)-3-formamidohexanoic acid (**13b**) (0.1481 g, 0.9303 mmol, 1 equiv) and EDC (0.5534 g, 2.886 mmol, 3.1 equiv) were suspended in THF (5.8 mL, 0.16 M) and cooled to 0 °C for 10 min. *N*-hydroxysuccinimide (0.3613 g, 3.139 mmol, 3.4 equiv) was added to the stirring solution in one portion. The mixture was stirred at 0 °C for 30 min, warmed to 22 °C, and stirred for 17.5 h. The mixture was filtered through a pad of celite and washed with Et_2O (20 mL). The combined filtrates were concentrated by rotary evaporation to afford the crude product. Purification by silica flash chromatography (75% EtOAc/hexanes) yielded β-formamido NHS ester **14b** as a white powder (0.0966 g, 41%).

TLC: R_f 0.26 (75% EtOAc/hexanes, CAM, $KMnO_4$). **^{1}H NMR** (500 MHz, $CDCl_3$): δ 8.15 (d, *J* = 1.6 Hz, 1H), 6.19 (d, *J* = 9.2 Hz, 1H), 4.41 (tt, *J* = 11.6, 4.4 Hz, 1H), 2.91 (dd, *J* = 15.1, 5.4 Hz, 1H), 2.82 (d, *J* = 6.5 Hz, 4H), 2.81 – 2.72 (m, 3H), 1.60 (ddt, *J* = 11.6, 8.8, 6.4 Hz, 2H), 1.48 – 1.28 (m, 2H), 0.92 (t, *J* = 7.3 Hz, 3H). **^{13}C NMR** (126 MHz, $CDCl_3$): δ 169.64, 169.03, 166.80, 166.20, 165.93, 164.40, 160.91, 151.50, 76.89, 49.13, 44.29, 37.66, 37.17, 36.99, 35.61, 35.20, 31.29, 25.43, 25.41, 25.31, 25.26, 19.14, 18.82, 13.44, 13.30. **HRMS** (ESI) *m/z* calcd for $C_{11}H_{17}N_2O_5$ ($[M+H]^+$) 257.1137; found 257.1135.

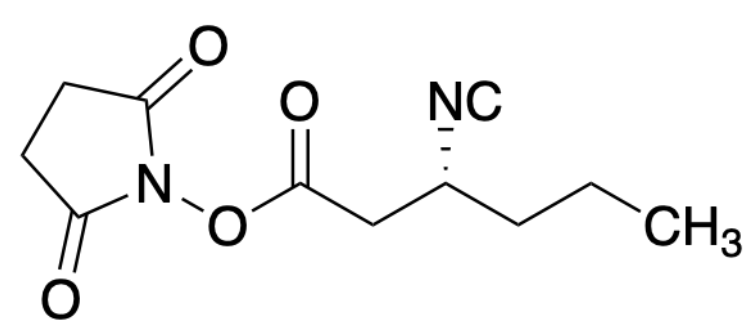

2,5-Dioxopyrrolidin-1-yl (*3R*)-3-isocyanohexanoate (15b): In a 5-mL roundbottom flask, 2,5-dioxopyrrolidin-1-yl (*3R*)-3-formamidohexanoate (**14b**) (0.200 g, 0.7804 mmol, 1 equiv) and diisopropylamine (0.300 mL, 2.125 mmol, 2.7 equiv) were dissolved in CH_2Cl_2 (2.6 mL, 0.3 M total) and cooled to –30 °C for 15 min. $POCl_3$ (0.090 mL, 0.9637 mmol, 1.2 equiv) was added to the solution and the mixture was stirred at –30 °C for 3.5 h. The reaction mixture was quenched with satd aq $NaHCO_3$ and extracted with CH_2Cl_2 (2 x 50 mL). The combined organic extracts were washed with brine, dried (Na_2SO_4), filtered, and concentrated by rotary evaporation to afford the crude product. Purification by silica flash chromatography (40% EtOAc/hexanes) yielded β-isocyano NHS ester (**15b**) as a white powder (0.1047 g, 56%).

TLC: R_f 0.26 (40% EtOAc/hexanes, UV, $KMnO_4$). **^{1}H NMR** (600 MHz, $CDCl_3$): δ 4.07 – 4.01 (m, 1H), 3.06 (dd, *J* = 16.4, 7.0 Hz, 1H), 2.90 (dd, *J* = 16.4, 6.5 Hz, 1H), 2.85 (s, 4H), 1.71 (ddt, *J* = 16.8, 13.9, 9.3 Hz, 2H), 1.65 – 1.56 (m, 1H), 1.50 (tdd, *J* = 13.6, 7.2, 4.1 Hz, 1H), 0.98 (t, *J* = 7.3 Hz, 3H). **^{13}C NMR** (151 MHz, $CDCl_3$): δ 168.61, 164.80, 158.09, 50.32, 50.28, 50.24, 36.99,

36.34, 25.57, 18.74, 13.19. **HRMS** (ESI) *m/z* calcd for $C_{11}H_{17}N_2O_5$ ($[M+H+H_2O]^+$) 257.1137; found 257.1134.

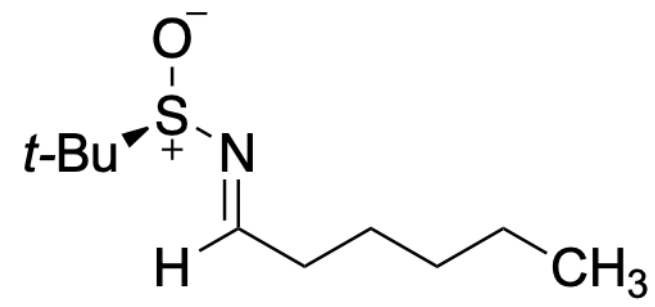

***(R,E)*-*N*-Hexylidene-2-methylpropane-2-sulfinamide (9c):** In a 250-mL roundbottom flask, hexanal (6 mL, 48.76 mmol, 1 equiv), (*R*)-2-methylpropane-2-sulfinamide (16.5 g, 53.63 mmol, 1.1 equiv), and titanium (IV) ethoxide (25 mL, 119.2 mmol, 2.4 equiv) were dissolved in THF (100 mL, 0.49 M) and stirred for 2 h at rt. The reaction mixture was quenched with brine (50 mL) and filtered over a pad of celite that had been rinsed with EtOAc. The filtrate was extracted with EtOAc (2 x 100 mL). The combined organic extracts were washed with brine (1 x 25 mL), dried (Na_2SO_4), filtered, and concentrated by rotary evaporation. Purification by silica flash chromatography (10% EtOAc/hexanes) yielded sulfinimine **9c** as a yellow oil (7.43 g, 75%).

TLC: R_f 0.23 (10% EtOAc/hexanes, UV, *p*-anisaldehyde). **^{1}H-NMR** (600 MHz, $CDCl_3$): δ 8.06 (t, *J* = 4.8 Hz, 1H), 2.53 – 2.48 (m, 2H), 1.65 – 1.59 (m, 2H), 1.37 – 1.31 (m, 4H), 1.19 (s, 9H), 0.89 (t, *J* = 7.2 Hz, 3H). **^{13}C-NMR** (151 MHz, $CDCl_3$): δ 169.82, 56.50, 36.09, 31.39, 25.19, 22.40, 22.35, 13.92. **HRMS** (ESI) *m/z* calcd for $C_{10}H_{21}NOS$ ($[M+H]^+$) 204.1422; found 204.1423.

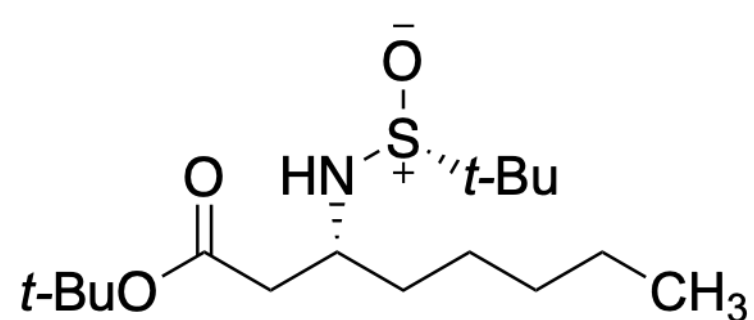

***tert*-Butyl (*R*)-3-(((*R*)-*tert*-butylsulfinyl)amino)octanoate (11c):** In a 200-mL roundbottom flask, (*R,E*)-*N*-hexylidene-2-methylpropane-2-sulfinamide (**9c**) (1.500 g, 9.301 mmol, 1 equiv) was dissolved in THF (19 mL, 0.49 M, 2.6 equiv). A solution of Reformatsky reagent (**10**) in THF (6.3045 g, 24.20 mmol, 0.5 M) was added to the reaction mixture and stirred for 17.5 h at rt. The reaction mixture was quenched with satd aq NH_4Cl, diluted with H_2O, and extracted with EtOAc (2 x 75 mL). The combined organic extracts were washed with brine, dried (Na_2SO_4), filtered, and concentrated by rotary evaporation to afford the crude product. Purification by silica flash chromatography (30–50% EtOAc/hexanes) yielded β-sulfinamido ester **11c** as a pale yellow oil (1.890 g, 73%).

TLC: R_f 0.25 (25% EtOAc/hexanes, CAM, *p*-anisaldehyde). **^{1}H NMR** (600 MHz, $CDCl_3$): δ 4.06 (dd, *J* = 9.3, 7.5 Hz, 1H), 3.43 (dtt, *J* = 10.8, 7.6, 3.9 Hz, 1H), 2.59 (dd, *J* = 15.7, 5.3 Hz, 1H), 2.45 (dd, *J* = 15.7, 5.4 Hz, 1H), 1.55 – 1.47 (m, 1H), 1.47 – 1.41 (m, 1H), 1.39 (d, *J* = 1.1 Hz, 9H), 1.33 (dt, *J* = 16.1, 5.7 Hz, 1H), 1.30 – 1.17 (m, 5H), 1.17 – 1.11 (m, 9H), 0.85 – 0.77 (m, 3H). **^{13}C NMR** (151 MHz, $CDCl_3$): δ 171.31, 81.14, 55.74, 53.76, 41.69, 35.26, 31.46, 28.13, 25.52, 22.71, 22.62, 22.52, 13.99. **HRMS** (ESI) *m/z* calcd for $C_{16}H_{33}NO_3S$ ($[M+H+Na]^+$) 342.21; found 342.21.

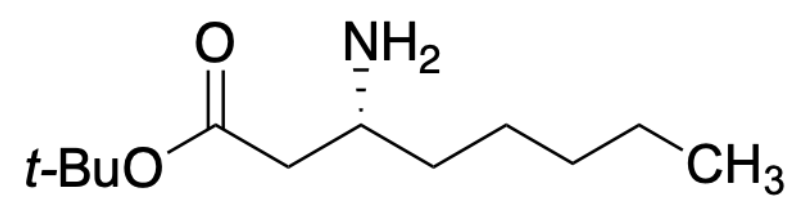

***tert*-Butyl (*3R*)-3-aminooctanoate (12c):** In a 500-mL roundbottom flask, *tert*-butyl (*R*)-3-(((*R*)-*tert*-butylsulfinyl)amino)octanoate amine (**11c**) (3.761 g, 11.76 mmol, 1 equiv), THF (90 mL, 0.13 M), and HCl in Et_2O (2.0 M, 60 mL, 120 mmol, 10 equiv) were added and stirred for 3 h at rt. The reaction mixture was concentrated by rotary evaporation to afford the crude HCl salt. The resulting HCl salt was dissolved in EtOAc, washed with satd aq $NaHCO_3$ until pH 9, and the aq phase was back-extracted with EtOAc (2 x 50 mL). The combined organic extracts were dried (Na_2SO_4), filtered, and concentrated by rotary evaporation to afford β-amino ester **12c** as a yellow oil (2.5 g, quant.), which was used without further purification.

TLC: R_f 0.30 (80% EtOAc + 1% triethylamine, ninhydrin).

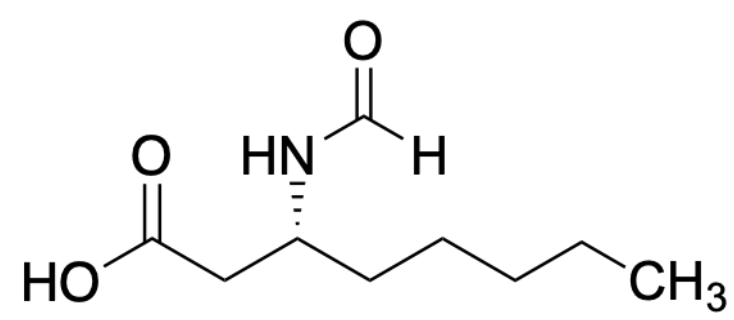

(*3R*)-3-Formamidooctanoic acid: In a 250-mL roundbottom flask containing *tert*-butyl (*3R*)-3-aminooctanoate (**12c**) (2.3445 g, 10.88 mmol, 1 equiv), formic acid (20 mL, 530.1 mmol, 49 equiv, 3:2 ratio of formic acid to acetic anhydride), and acetic anhydride (14 mL, 148.1 mmol, 14 equiv) were heated together at 80°C for 16 h. The reaction mixture was concentrated by rotary evaporation to afford the crude product. Purification by recrystallization in ~6:1 hexanes:EtOAc yielded β-formamido acid (**13c**) as a white powder (0.8159 g, 40%).

TLC: R_f 0.40 (80% EtOAc/hexanes + 1% AcOH, CAM, $KMnO_4$). **^{1}H NMR** (600 MHz, MeOD): δ 7.95 (d, J = 9.0 Hz, 1H), 4.22 – 4.15 (m, 1H), 2.41 (d, J = 6.6 Hz, 2H), 1.92 (s, 1H), 1.51 (ddq, J = 18.6, 9.0, 4.6 Hz, 1H), 1.47 – 1.37 (m, 1H), 1.37 – 1.19 (m, 6H), 0.84 (t, J = 6.7 Hz, 3H). **^{13}C NMR** (151 MHz, MeOD): δ 173.82, 173.27, 173.14, 165.69, 161.87, 161.86, 49.96, 49.95, 48.04, 47.89, 47.75, 47.61, 47.47, 47.33, 47.18, 45.12, 45.11, 39.88, 38.83, 34.62, 33.84, 31.23, 31.13, 25.33, 25.29, 22.19, 19.36, 19.35, 12.94. **HRMS** (ESI) *m/z* calcd for $C_9H_{16}NO_3$ ([M+H]$^+$) 186.1136; found 186.1130.

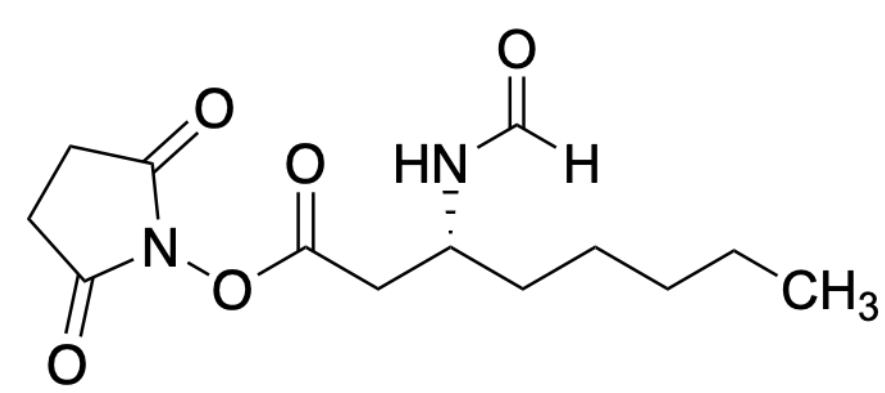

2,5-Dioxopyrrolidin-1-yl (*3R*)-3-formamidooctanoate (14c): In a 100-mL roundbottom flask, (*3R*)-3-formamidooctanoic acid (**13c**) (0.6749 g, 3.604 mmol, 1 equiv) and EDC (2.3413 g, 12.21 mmol, 3.4 equiv) were suspended in THF (36 mL, 0.1 M) and cooled to 0 °C for 10 min. *N*-hydroxysuccinimide (1.6299 g, 14.15 mmol, 3.9 equiv) was added to the stirring solution in one portion. The mixture was stirred at 0 °C for 30 min, warmed to 22 °C, and stirred for 14 h. The mixture was filtered through a pad of celite and washed with Et_2O. The combined filtrates were concentrated by rotary evaporation to afford the crude product. Purification by silica flash chromatography (75% EtOAc/hexanes) yielded β-formamido NHS ester **14c** as a white powder (0.6999 g, 68%).

TLC: R_f 0.29 (75% EtOAc/hexanes, CAM, $KMnO_4$). **^{1}H NMR** (600 MHz, $CDCl_3$): δ 8.15 (s, 1H), 6.15 (d, *J* = 9.1 Hz, 1H), 4.40 (tq, *J* = 7.5, 3.8 Hz, 1H), 2.92 (dd, *J* = 15.1, 5.4 Hz, 1H), 2.84 (q, *J* = 3.6 Hz, 4H), 2.80 (dq, *J* = 11.7, 5.7 Hz, 2H), 1.61 (p, *J* = 7.9 Hz, 2H), 1.39 – 1.33 (m, 2H), 1.28 (tdd, *J* = 16.3, 8.4, 3.2 Hz, 5H), 0.86 (t, *J* = 6.7 Hz, 3H). *NHS impurity at 2.7 ppm.* **^{13}C NMR** (151 MHz, $CDCl_3$): δ 172.09, 169.73, 169.14, 166.35, 166.07, 164.54, 161.05, 49.62, 44.75, 37.86, 37.38, 35.78, 35.12, 33.26, 31.53, 31.29, 31.16, 25.77, 25.60, 25.58, 25.49, 25.47, 25.42, 22.44, 22.42, 13.96, 13.94. **HRMS** (ESI) *m/z* calcd for $C_{13}H_{20}N_2O_5$ ($[M+H+Na]^+$) 307.1276; found 307.1270.

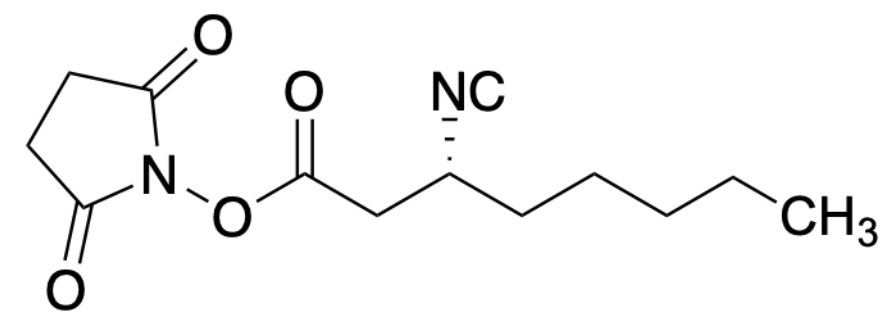

2,5-Dioxopyrrolidin-1-yl (*3R*)-3-isocyanooctanoate (15c): In a 5-mL roundbottom flask, 2,5-dioxopyrrolidin-1-yl (*3R*)-3-formamidooctanoate (**14c**) (0.0593 g, 0.2085 mmol, 1 equiv) and diisopropylamine (0.118 mL, 0.836 mmol, 4 equiv) were dissolved in CH_2Cl_2 (0.7 mL, 0.3 M total) and cooled to –30 °C for 15 min. $POCl_3$ (0.035 mL, 0.3753 mmol, 1.8 equiv) was added to the solution and the mixture was stirred at –30 °C for 1.5 h. The reaction mixture was quenched with satd aq $NaHCO_3$ and extracted with CH_2Cl_2 (1 x 60 mL). The combined organic extracts were washed with brine, dried (Na_2SO_4), filtered, and concentrated by rotary evaporation to afford the crude product. Purification by silica flash chromatography (40% EtOAc/hexanes) yielded β-isocyano NHS ester (**15c**) as a white powder (0.0249 g, 45%).

TLC: R_f 0.32 (40% EtOAc/hexanes, UV, $KMnO_4$). **^{1}H NMR** (600 MHz, $CDCl_3$): δ 4.02 (p, *J* = 6.8 Hz, 1H), 3.06 (dd, *J* = 16.4, 7.0 Hz, 1H), 2.90 (dd, *J* = 16.4, 6.5 Hz, 1H), 2.85 (s, 4H), 1.72 (q, *J* = 7.5 Hz, 2H), 1.62 – 1.53 (m, 1H), 1.50 – 1.41 (m, 1H), 1.32 (pdd, *J* = 10.0, 5.1, 2.6 Hz, 4H), 0.90 (t, *J* = 6.8 Hz, 3H). **^{13}C NMR** (151 MHz, $CDCl_3$): δ 167.61, 163.79, 157.00, 59.38, 49.57, 49.52, 49.49, 35.96, 33.31, 29.81, 28.68, 24.55, 24.06, 21.33, 20.04, 13.18, 12.89. **HRMS** (ESI) *m/z* calcd for $C_{13}H_{21}N_2O_5$ ($[M+H+H_2O]^+$) 285.1450; found 285.1449.

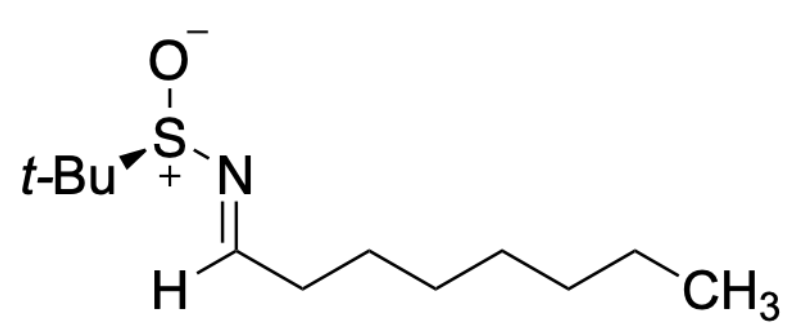

(*R*,*E*)-2-Methyl-*N*-octylidenepropane-2-sulfinamide (9d): In a 50-mL roundbottom flask, octanal (0.625 mL, 4.001 mmol, 1 equiv), (*R*)-2-methylpropane-2-sulfinamide (0.5334 g, 4.401 mmol, 1.1 equiv), and titanium (IV) ethoxide (2 mL, 9.539 mmol, 2.4 equiv) were dissolved in THF (8 mL, 0.5 M) and stirred for 18.5 h at 22 °C. The reaction mixture was quenched with brine (12 mL) and filtered over a pad of celite that had been rinsed with EtOAc. The filtrate was extracted with EtOAc (2 x 100 mL). The combined organic extracts were washed with brine (1 x 25 mL), dried (Na_2SO_4), filtered, and concentrated by rotary evaporation. Purification by silica flash chromatography (10% EtOAc/hexanes) yielded sulfinimine **9d** as a colorless oil (0.7248 g, 78%).

TLC: R_f 0.37 (15% EtOAc/hexanes, UV, *p*-anisaldehyde). $[\alpha]_D^{25}$: –221.53° (*c* 0.7, $CHCl_3$). **^{1}H-NMR** (500 MHz, $CDCl_3$): δ 8.06 (t, *J* = 4.8 Hz, 1H), 2.53 – 2.47 (m, 2H), 1.66 – 1.58 (m, 2H), 1.38 – 1.21 (m, 8H), 1.19 (s, 9H), 0.87 (t, *J* = 6.9 Hz, 3H). **^{13}C-NMR** (126 MHz, $CDCl_3$): δ 169.83,

77.24, 56.50, 36.14, 31.69, 29.21, 29.02, 25.53, 22.60, 22.36, 14.07. **HRMS** (ESI) *m/z* calcd for $C_{12}H_{25}NOS$ ($[M+H]^+$) 232.1736; found 232.1735.

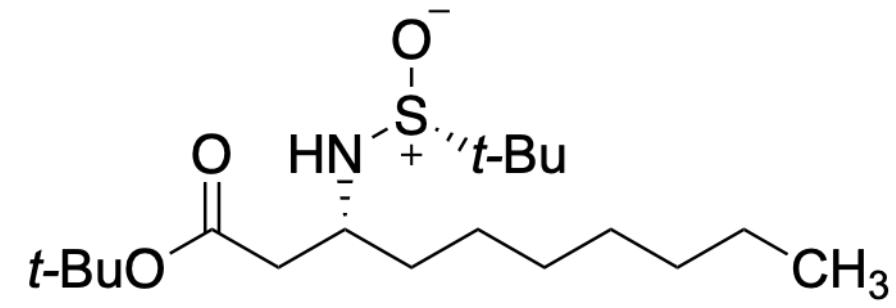

***tert*-Butyl (*R*)-3-(((*R*)-*tert*-butylsulfinyl)amino)decanoate (11d):** In a 200-mL roundbottom flask, (*R,E*)-*N*-octylidene-2-methylpropane-2-sulfinamide (**9d**) (8.2698 g, 35.73 mmol, 1 equiv) was dissolved in THF (68 mL, 0.53 M). A solution of Reformatsky reagent (**10**) in THF (24.74 g, 95 mmol, 0.5 M, 2.7 equiv) was added to the reaction mixture and stirred for 18 h at rt. The reaction mixture was quenched with satd aq NH_4Cl, diluted with H_2O, and extracted with EtOAc (2 x 125 mL). The combined organic extracts were washed with brine, dried (Na_2SO_4), filtered, and concentrated by rotary evaporation to afford the crude product. Purification by silica flash chromatography (25% EtOAc/hexanes) yielded β-sulfinamido ester **11d** as a colorless oil (9.7393 g, 78%).

TLC: R_f 0.22 (25% EtOAc/hexanes, CAM, *p*-anisaldehyde). **^{1}H NMR** (600 MHz, $CDCl_3$): δ 4.12 (t, *J* = 7.2 Hz, 1H), 3.50 (tq, *J* = 7.9, 5.5 Hz, 1H), 2.65 (dd, *J* = 15.6, 5.3 Hz, 1H), 2.51 (dd, *J* = 15.7, 5.5 Hz, 1H), 1.59 (s, 1H), 1.59 – 1.54 (m, 1H), 1.54 – 1.48 (m, 1H), 1.45 (s, 9H), 1.37 (ddd, *J* = 16.8, 9.2, 4.2 Hz, 1H), 1.34 – 1.23 (m, 10H), 1.22 (s, 9H), 0.87 (t, *J* = 7.0 Hz, 3H). **^{13}C NMR** (151 MHz, $CDCl_3$): δ 171.33, 81.16, 55.76, 53.77, 41.70, 35.32, 31.78, 29.26, 29.19, 28.14, 28.05, 25.87, 22.73, 22.63, 22.45, 14.21, 14.10. **HRMS** (ESI) *m/z* calcd for $C_{18}H_{37}NO_3S$ ($[M+H]^+$+Na) 370.2392; found 370.2379.

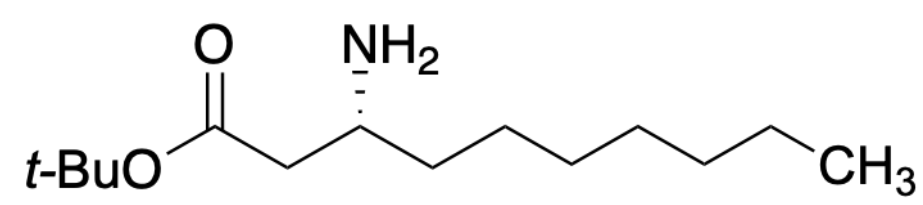

***tert*-Butyl (*3R*)-3-aminodecanoate (12d):** In a 500-mL roundbottom flask, *tert*-butyl (*R*)-3-(((*R*)-*tert*-butylsulfinyl)amino)decanoate amine (**11d**) (9.2336 g, 25.56 mmol, 1 equiv), THF (204 mL, 0.13 M), and HCl in Et_2O (2.0 M, 133 mL, 266 mmol, 10 equiv) were added and stirred for 3.5 h at rt. The reaction mixture was concentrated by rotary evaporation to afford the crude HCl salt. The resulting HCl salt was dissolved in EtOAc, washed with satd aq $NaHCO_3$ until pH 9, and the aq phase was back-extracted with EtOAc (2 x 50 mL). The combined organic extracts were dried (Na_2SO_4), filtered, and concentrated by rotary evaporation to afford β-amino ester **12d** as a yellow oil (6.4 g, quant.), which was used without further purification.

TLC: R_f 0.30 (80% EtOAc + 1% triethylamine, ninhydrin). $[\alpha]^{22}$: 2.3338° (*c* 0.88, $CHCl_3$).

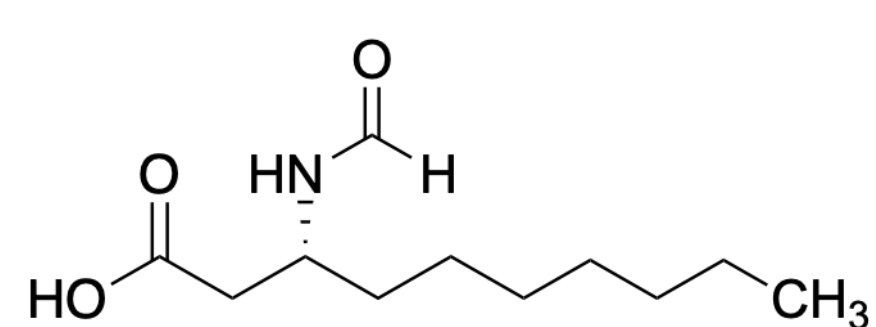

(*3R*)-3-Formamidodecanoic acid (13d): In a 15-mL roundbottom flask containing *tert*-butyl (*3R*)-3-aminodecanoate (**12d**) (0.6026 g, 2.475 mmol, 1 equiv), formic acid (5.2 mL, 137.8 mmol, 56 equiv, 3:2 ratio of formic acid to acetic anhydride), and acetic anhydride (3.5 mL, 37.02 mmol, 15 equiv) were heated together at 80 °C for 19 h. The reaction mixture was concentrated by rotary

evaporation to afford the crude product. Purification by silica flash chromatography (50–75% EtOAc/hexanes + 1% AcOH) yielded β-formamido acid (**13d**) as a white powder (0.1893 g, 36%).

TLC: R_f 0.40 (80% EtOAc/hexanes + 1% AcOH, CAM, $KMnO_4$). **^{1}H NMR** (600 MHz, MeOD): δ 7.98 – 7.94 (m, 1H), 4.20 (dq, *J* = 9.2, 6.4 Hz, 1H), 2.42 (d, *J* = 6.5 Hz, 2H), 1.52 (ddd, *J* = 14.4, 8.7, 4.9 Hz, 1H), 1.48 – 1.39 (m, 1H), 1.36 – 1.20 (m, 10H), 0.84 (t, *J* = 7.0, 1.9 Hz, 3H). **^{13}C NMR** (151 MHz, MeOD): δ 173.83, 173.30, 173.17, 165.69, 161.86, 60.14, 49.96, 45.11, 39.90, 38.84, 34.66, 33.87, 31.54, 31.53, 28.96, 28.90, 28.86, 25.66, 25.61, 22.29, 19.47, 19.35, 13.07, 13.02. **HRMS** (ESI) *m/z* calcd for $C_{11}H_{20}NO_3$ ($[M+H]^+$) 214.1443; found 214.1451.

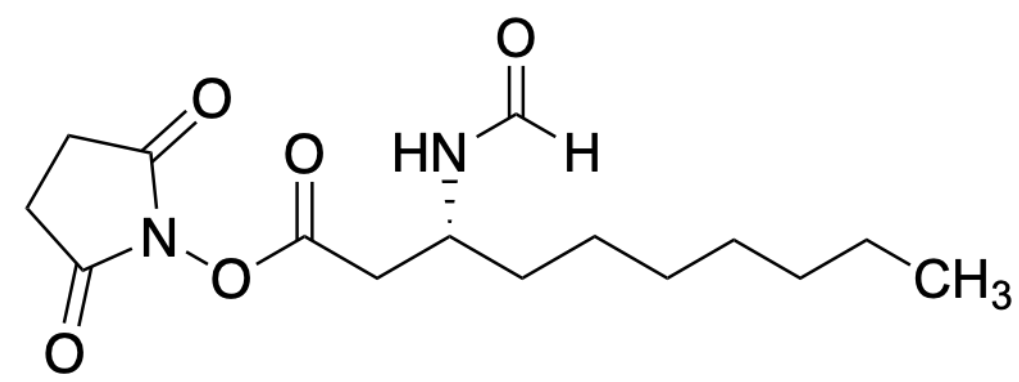

2,5-Dioxopyrrolidin-1-yl (*3R*)-3-formamidodecanoate (14d): In a 25-mL roundbottom flask, (*3R*)-3-formamidodecanoic acid (**13d**) (0.1893 g, 0.8788 mmol, 1 equiv) and EDC (0.5299 g, 2.764 mmol, 3.1 equiv) were suspended in THF (5.5 mL, 0.16 M) and cooled to 0 °C for 10 min. *N*-hydroxysuccinimide (0.3490 g, 3.032 mmol, 3.5 equiv) was added to the stirring solution in one portion. The mixture was stirred at 0 °C for 30 min, warmed to 22 °C, and stirred for 18 h. The mixture was filtered through a pad of celite and washed with Et_2O. The combined filtrates were concentrated by rotary evaporation to afford the crude product. Purification by silica flash chromatography (75% EtOAc/hexanes) yielded β-formamido NHS ester **14d** as a white powder (0.2123 g, 77%).

TLC: R_f 0.33 (75% EtOAc/hexanes, CAM, $KMnO_4$). **^{1}H NMR** (600 MHz, $CDCl_3$): δ 8.18 (s, 1H), 6.02 (d, *J* = 9.2 Hz, 1H), 4.42 (tq, *J* = 7.4, 3.8 Hz, 1H), 2.94 (dd, *J* = 15.0, 5.4 Hz, 1H), 2.85 (s, 4H), 2.80 (dd, *J* = 14.9, 4.5 Hz, 1H), 1.63 (q, *J* = 7.4 Hz, 2H), 1.36 (dt, *J* = 13.5, 8.0 Hz, 2H), 1.27 (qd, *J* = 17.2, 9.3 Hz, 8H), 0.87 (t, *J* = 6.9 Hz, 3H). **^{13}C NMR** (151 MHz, $CDCl_3$): δ 168.97, 166.34, 165.98, 164.01, 160.84, 49.38, 44.72, 38.05, 35.78, 35.15, 33.27, 31.72, 31.68, 29.10, 29.09, 29.05, 28.98, 26.16, 25.87, 25.60, 25.57, 25.46, 25.41, 22.61, 22.60, 14.08, 14.06, 1.03. **HRMS** (ESI) *m/z* calcd for $C_{16}H_{28}N_2O_6$ ($[M+Na+MeOH]^+$) 367.1845; found 367.1832.

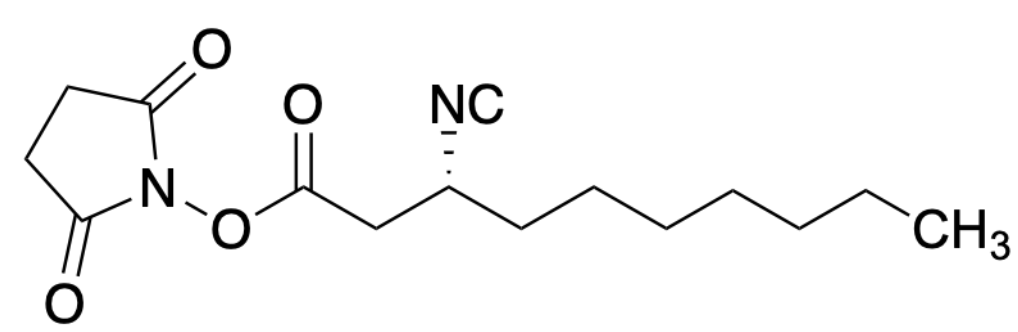

2,5-Dioxopyrrolidin-1-yl (*3R*)-3-isocyanodecanoate (15d): In a 5-mL roundbottom flask, 2,5-dioxopyrrolidin-1-yl (*3R*)-3-formamidodecanoate (**14d**) (0.0770 g, 0.2465 mmol, 1 equiv) and diisopropylamine (0.0940 mL, 0.6655 mmol, 2.7 equiv) were dissolved in CH_2Cl_2 (0.82 mL, 0.3 M total) and cooled to –30 °C for 15 min. $POCl_3$ (0.028 mL, 0.2995 mmol, 1.2 equiv) was added to the solution and the mixture was stirred at –30 °C for 3 h. The reaction mixture was quenched with satd aq $NaHCO_3$ and extracted with CH_2Cl_2 (1 x 60 mL). The combined organic extracts were washed with brine, dried (Na_2SO_4), filtered, and concentrated by rotary evaporation to afford the crude product. Purification by silica flash chromatography (40% EtOAc/hexanes) yielded β-isocyano NHS ester (**15d**) as a white powder (0.0449 g, 62%).

TLC: R_f 0.31 (40% EtOAc/hexanes, UV, $KMnO_4$). **^{1}H NMR** (600 MHz, $CDCl_3$): δ 4.03 (p, *J* = 6.8 Hz, 1H), 3.06 (dd, *J* = 16.4, 7.0 Hz, 1H), 2.91 (dd, *J* = 16.4, 6.6 Hz, 1H), 2.86 (s, 4H), 1.72 (q, *J* = 7.6 Hz, 2H), 1.62 – 1.56 (m, 1H), 1.51 – 1.41 (m, 1H), 1.40 – 1.34 (m, 1H), 1.34 – 1.23 (m, 8H), 0.89 (t, *J* = 6.9 Hz, 3H). **^{13}C NMR** (151 MHz, $CDCl_3$): δ 168.57, 164.79, 158.09, 50.54, 37.00, 34.40, 31.66, 28.97, 28.67, 25.57, 25.42, 22.59, 14.07, 1.03. **HRMS** (ESI) *m/z* calcd for $C_{15}H_{25}N_2O_5$ ($[M+H+H_2O]^+$) 313.1763; found 313.1765.

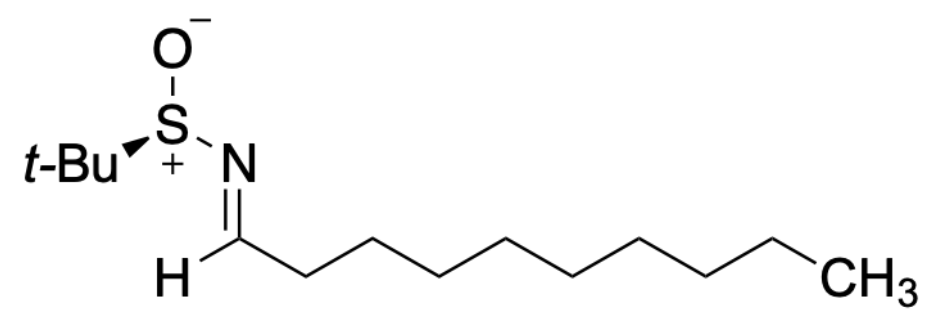

(*R*,*E*)-*N*-Dodecylidene-2-methylpropane-2-sulfinamide (9e): In a 500-mL roundbottom flask, decanal (16 mL, 84.98 mmol, 1 equiv), (*R*)-2-methylpropane-2-sulfinamide (11.3263 g, 93.39 mmol, 1.1 equiv), and titanium (IV) ethoxide (45 mL, 214.6 mmol, 2.5 equiv) were dissolved in THF (170 mL, 0.5 M) and stirred for 18.5 at rt. Sodium sulfate (16 g) was added to the mixture at 16 h to drive the reaction to completion. The reaction mixture was filtered over a pad of celite that had been rinsed with EtOAc, quenched with brine (75 mL), and filtered a second time over a pad of celite that had been rinsed with EtOAc. The filtrate was extracted with EtOAc (2 x 250 mL). The combined organic extracts were washed with brine (1 x 50 mL), dried (Na_2SO_4), filtered, and concentrated by rotary evaporation. Purification by silica flash chromatography (10% EtOAc/hexanes) yielded sulfinimine **9e** as a colorless oil (12.9751 g, 59%).

TLC: R_f 0.56 (15% EtOAc/hexanes, UV, *p*-anisaldehyde). **^{1}H-NMR** (600 MHz, $CDCl_3$): δ 8.06 (t, *J* = 4.8 Hz, 1H), 2.53 – 2.49 (m, 2H), 1.65 – 1.58 (m, 2H), 1.38 – 1.22 (m, 12H), 1.19 (s, 9H), 0.88 (t, *J* = 6.9 Hz, 3H). **^{13}C-NMR** (151 MHz, $CDCl_3$): δ 169.84, 56.51, 36.14, 31.86, 29.45, 29.36, 29.26, 29.25, 25.53, 22.67, 22.36, 14.11. **HRMS** (ESI) *m/z* calcd for $C_{14}H_{29}NOS$ ($[M+H]^+$) 260.2048; found 260.2053.

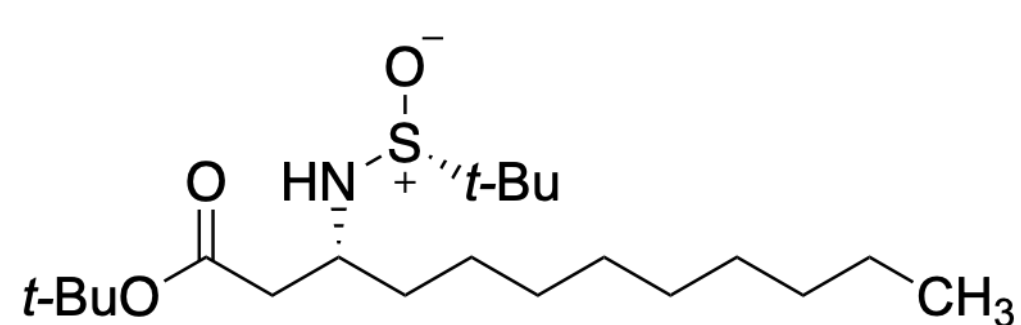

***tert*-Butyl (*R*)-3-(((*R*)-*tert*-butylsulfinyl)amino)dodecanoate (11e):** In a 500-mL roundbottom flask, (*R,E*)-*N*-decylidene-2-methylpropane-2-sulfinamide (**9e**) (10.1643 g, 39.15 mmol, 1 equiv) was dissolved in THF (74 mL, 0.53 M). A solution of Reformatsky reagent (**10**) in THF (27.3458 g, 104.9 mmol, 0.5 M, 2.7 equiv) was added to the reaction mixture and stirred for 18 h at rt. The reaction mixture was quenched with satd aq NH_4Cl, diluted with H_2O, and extracted with EtOAc (2 x 125 mL). The combined organic extracts were washed with brine, dried (Na_2SO_4), filtered, and concentrated by rotary evaporation to afford the crude product. Purification by silica flash chromatography (15% EtOAc/hexanes) yielded β-sulfinamido ester **11e** as a colorless oil (13.941 g, 95%).

TLC: R_f 0.40 (30% EtOAc/hexanes, CAM, *p*-anisaldehyde). **^{1}H NMR** (600 MHz, $CDCl_3$): δ 4.10 (d, *J* = 8.0 Hz, 1H), 3.45 (dtt, *J* = 10.8, 7.8, 3.9 Hz, 1H), 2.61 (dd, *J* = 15.7, 5.3 Hz, 1H), 2.47 (dd, *J* = 15.7, 5.4 Hz, 1H), 1.57 – 1.49 (m, 1H), 1.49 – 1.43 (m, 1H), 1.41 (s, 9H), 1.38 – 1.32 (m, 1H), 1.32 – 1.19 (m, 13H), 1.17 (s, 9H), 0.83 (t, *J* = 7.0 Hz, 3H). **^{13}C NMR** (151 MHz, $CDCl_3$): δ

171.27, 81.08, 55.70, 53.73, 41.64, 35.27, 31.85, 29.47, 29.24, 28.09, 25.82, 22.68, 22.63, 22.59, 14.08. **HRMS** (ESI) *m/z* calcd for $C_{20}H_{41}NO_3S$ ([M+H]$^+$) 376.2885; found 376.2886.

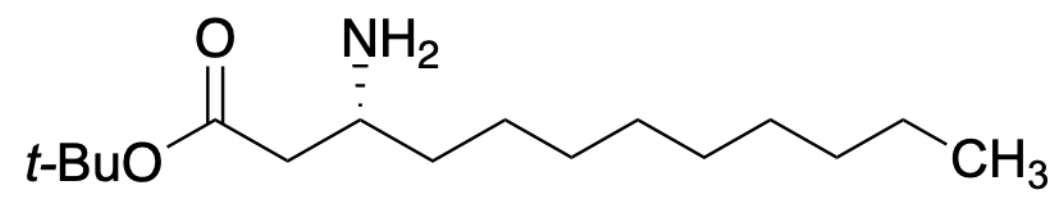

***tert*-Butyl (*3R*)-3-aminododecanoate (12e):** In a 500-mL roundbottom flask, *tert*-butyl (*R*)-3-(((*R*)-*tert*-butylsulfinyl)amino)dodecanoate amine (**11e**) (13.6467 g, 36.31 mmol, 1 equiv), THF (280 mL, 0.13 M), and HCl in Et_2O (2.0 M, 180 mL, 360 mmol, 10 equiv) were added and stirred for 6 h at rt. The reaction mixture was concentrated by rotary evaporation to afford the crude HCl salt. The resulting HCl salt was dissolved in EtOAc, washed with satd aq $NaHCO_3$ until pH 9, and the aq phase was back-extracted with EtOAc (2 x 50 mL). The combined organic extracts were dried (Na_2SO_4), filtered, and concentrated by rotary evaporation to afford β-amino ester **12e** as a yellow oil (6.2175 g, 63%), which was used without further purification.

TLC: R_f 0.19 (100% EtOAc, ninhydrin). **HRMS** (ESI) *m/z* calcd for $C_{17}H_{35}NO_3S$ ([M+H]$^+$+Na) 356.2235; found 356.2232.

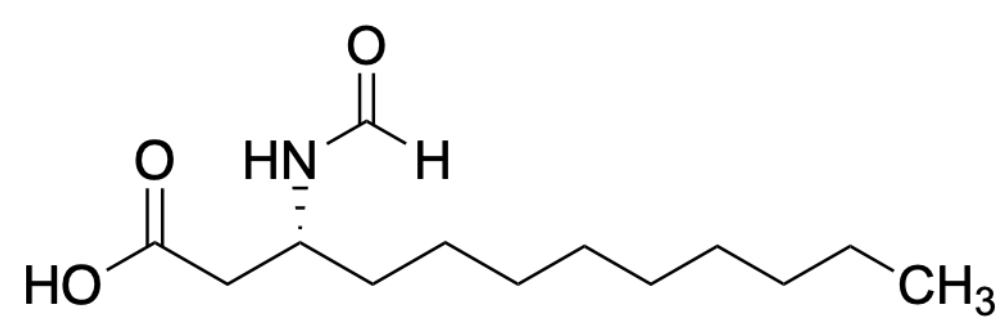

(*3R*)-3-Formamidododecanoic acid (13e): In a 50-mL roundbottom flask containing *tert*-butyl (*3R*)-3-aminododecanoate (**12d**) (0.7521 g, 2.770 mmol, 1 equiv), formic acid (6 mL, 159 mmol, 57 equiv, 3:2 ratio of formic acid to acetic anhydride), and acetic anhydride (4 mL, 42.31 mmol, 15 equiv) were heated together at 80 °C for 19 h. The reaction mixture was concentrated by rotary evaporation to afford the crude product. Purification by silica flash chromatography (50–75% EtOAc/hexanes + 1% AcOH) yielded β-formamido acid (**13e**) as a white powder (0.2334 g, 33%).

TLC: R_f 0.40 (75% EtOAc/hexanes + 1% AcOH, CAM, $KMnO_4$). **^{1}H NMR** (500 MHz, MeOD): δ 8.02 (d, *J* = 7.6 Hz, 1H), 4.26 (dt, *J* = 11.9, 5.8 Hz, 1H), 2.48 (d, *J* = 6.5 Hz, 2H), 1.59 (ddd, *J* = 14.4, 8.6, 4.9 Hz, 1H), 1.50 (dtd, *J* = 13.6, 8.6, 3.2 Hz, 1H), 1.31 (d, *J* = 6.7 Hz, 15H), 0.90 (t, *J* = 6.8 Hz, 3H). **^{13}C NMR** (126 MHz, MeOD): δ 161.87, 48.09, 45.20, 33.93, 31.68, 29.26, 29.05, 29.03, 28.93, 25.64, 22.35, 13.05. **HRMS** (ESI) *m/z* calcd for $C_{13}H_{26}NO_3$ ([M+H]$^+$) 244.1913; found 244.1909.

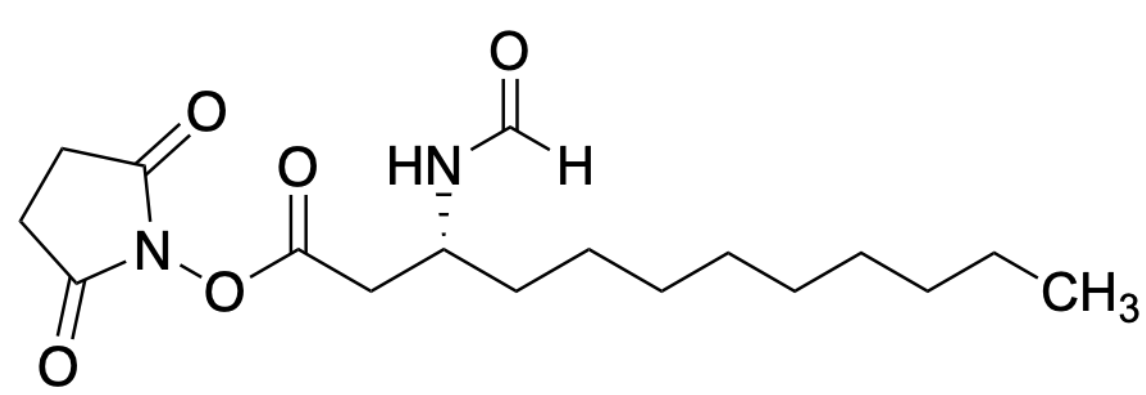

2,5-Dioxopyrrolidin-1-yl (*3R*)-3-formamidododecanoate (14e): In a 10-mL roundbottom flask, (*3R*)-3-formamidododecanoic acid (**13e**) (0.070 g, 0.2876 mmol, 1 equiv) and EDC (0.1821 g, 0.9499 mmol, 3.3 equiv) were suspended in THF (3 mL, 0.16 M) and cooled to 0 °C for 10 min. *N*-hydroxysuccinimide (0.1310 g, 1.138 mmol, 4 equiv) was added to the stirring solution in one portion. The mixture was stirred at 0 °C for 30 min, warmed to 22 °C, and stirred for 18 h. The mixture was filtered through a pad of celite and washed with Et_2O. The combined filtrates were

concentrated by rotary evaporation to afford the crude product. Purification by silica flash chromatography (75% EtOAc/hexanes) yielded β-formamido NHS ester **14e** as a white powder (0.0481 g, 49%).

TLC: R_f 0.42 (75% EtOAc/hexanes, CAM, $KMnO_4$). **^{1}H NMR** (500 MHz, $CDCl_3$): δ 8.11 (d, *J* = 1.6 Hz, 1H), 5.99 (d, *J* = 9.2 Hz, 1H), 4.35 (dtt, *J* = 9.5, 7.4, 5.0 Hz, 1H), 2.87 (dd, *J* = 15.0, 5.4 Hz, 1H), 2.79 (s, 4H), 2.74 (dd, *J* = 15.1, 4.7 Hz, 1H), 1.56 (q, *J* = 7.5 Hz, 2H), 1.29 (tt, *J* = 11.7, 6.1 Hz, 2H), 1.25 – 1.13 (m, 12H), 0.81 (t, *J* = 7.0 Hz, 3H). **^{13}C NMR** (126 MHz, $CDCl_3$): δ 166.34, 160.85, 77.24, 49.39, 44.74, 38.04, 35.78, 35.18, 33.30, 31.86, 31.84, 30.33, 29.48, 29.45, 29.43, 29.40, 29.26, 29.15, 29.03, 26.16, 25.86, 25.60, 25.58, 22.66, 14.10. **HRMS** (ESI) *m/z* calcd for $C_{17}H_{28}N_2O_5$ ($[M+H]^+$) 340.2080; found 340.2076.

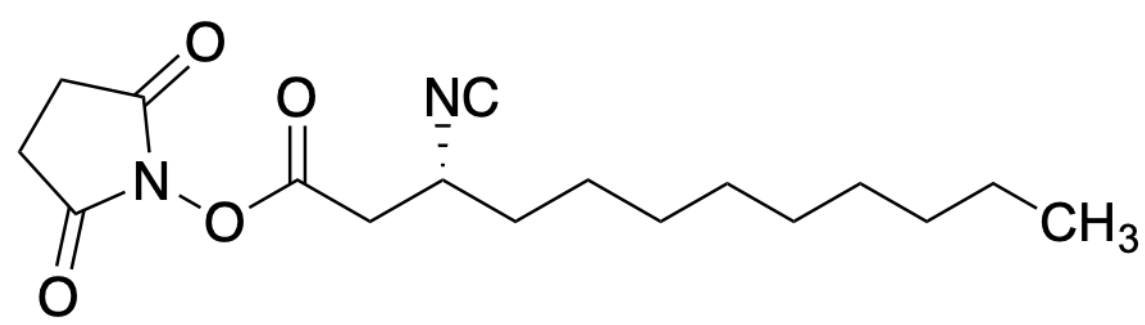

2,5-Dioxopyrrolidin-1-yl (*3R*)-3-isocyanododecanoate (15e): In a 5-mL roundbottom flask, 2,5-dioxopyrrolidin-1-yl (*3R*)-3-formamidododecanoate (**14e**) (0.0863 g, 0.2535 mmol, 1 equiv) and diisopropylamine (0.0970 mL, 0.6882 mmol, 2.7 equiv) were dissolved in CH_2Cl_2 (0.85 mL, 0.3 M total) and cooled to –30 °C for 15 min. $POCl_3$ (0.029 mL, 0.3101 mmol, 1.2 equiv) was added to the solution and the mixture was stirred at –30 °C for 4 h. The reaction mixture was quenched with satd aq $NaHCO_3$ and extracted with CH_2Cl_2 (1 x 60 mL). The combined organic extracts were washed with brine, dried (Na_2SO_4), filtered, and concentrated by rotary evaporation to afford the crude product. Purification by silica flash chromatography (40% EtOAc/hexanes) yielded β-isocyano NHS ester(**15e**) as a white powder (0.0437 g, 53%).

TLC: R_f 0.44 (40% EtOAc/hexanes, UV, $KMnO_4$). **^{1}H NMR** (600 MHz, $CDCl_3$): δ 4.03 (p, *J* = 6.8 Hz, 1H), 3.06 (dd, *J* = 16.4, 7.0 Hz, 1H), 2.90 (dd, *J* = 16.4, 6.5 Hz, 1H), 2.85 (s, 4H), 1.72 (q, *J* = 7.4 Hz, 2H), 1.62 – 1.52 (m, 1H), 1.46 (tq, *J* = 13.2, 7.6 Hz, 1H), 1.40 – 1.34 (m, 1H), 1.34 – 1.20 (m, 12H), 0.88 (t, *J* = 6.9 Hz, 3H). **^{13}C NMR** (151 MHz, $CDCl_3$): δ 168.58, 164.80, 158.06, 60.41, 50.55, 37.00, 34.40, 31.85, 29.71, 29.43, 29.31, 29.24, 28.71, 25.57, 25.43, 22.67, 21.07, 14.21, 14.12. **HRMS** (ESI) *m/z* calcd for $C_{17}H_{29}N_2O_5$ ($[M+H+H_2O]^+$) 341.2076; found 341.2075.

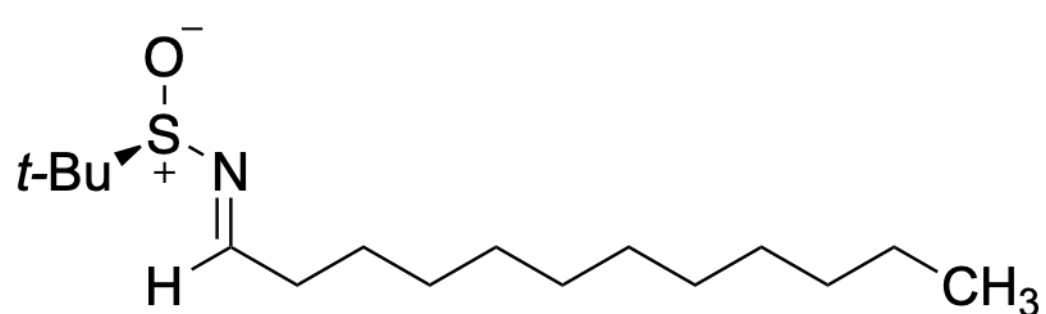

(*R*,*E*)-*N*-Dodecylidene-2-methylpropane-2-sulfinamide (9f): In a 500-mL roundbottom flask, dodecanal (19 mL, 85.55 mmol, 1 equiv), (*R*)-2-methylpropane-2-sulfinamide (11.4036 g, 94.05 mmol, 1.1 equiv), and titanium (IV) ethoxide (45 mL, 214.6 mmol, 2.5 equiv) were dissolved in THF (171 mL, 0.5 M) and stirred for 18 at rt. Sodium sulfate (13 g) was added to the mixture at 16 h to drive the reaction to completion. The reaction mixture was filtered over a pad of celite that had been rinsed with EtOAc, quenched with brine (75 mL), and filtered a second time over a pad of celite that had been rinsed with EtOAc. The filtrate was extracted with EtOAc (2 x 250 mL). The combined organic extracts were washed with brine (1 x 50 mL), dried (Na_2SO_4), filtered, and concentrated by rotary evaporation. Purification by silica flash chromatography (10% EtOAc/hexanes) yielded sulfinimine **9f** as a colorless oil (13.0759 g, 53%).

TLC: R_f 0.56 (15% EtOAc/hexanes, UV, *p*-anisaldehyde). $[\alpha]^{22}$: –177.3935° (*c* 1.09, $CHCl_3$). **^{1}H-NMR** (600 MHz, $CDCl_3$): δ 8.06 (t, *J* = 4.8 Hz, 1H), 2.53 – 2.49 (m, 2H), 1.65 – 1.58 (m, 2H), 1.38 – 1.22 (m, 12H), 1.19 (s, 9H), 0.88 (t, *J* = 6.9 Hz, 3H). **^{13}C-NMR** (151 MHz, $CDCl_3$): δ 169.84, 56.51, 36.14, 31.86, 29.45, 29.36, 29.26, 29.25, 25.53, 22.67, 22.36, 14.11. **HRMS** (ESI) *m/z* calcd for $C_{16}H_{33}NOS$ ($[M+H]^+$) 288.2361; found 288.2362.

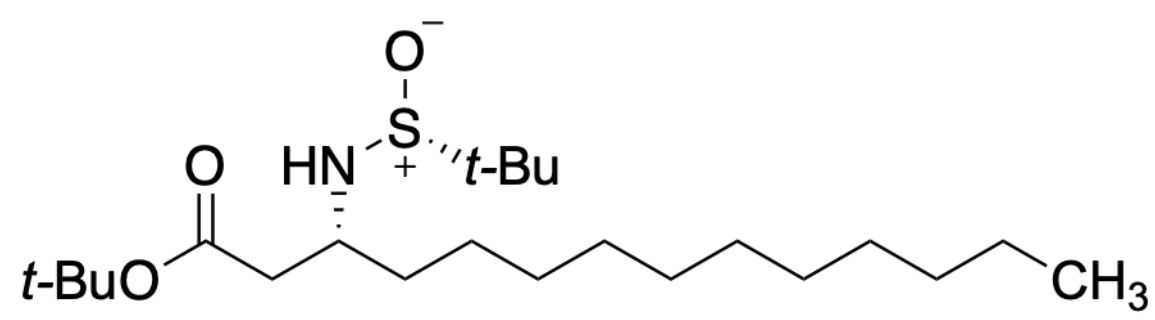

***tert*-Butyl (*R*)-3-(((*R*)-*tert*-butylsulfinyl)amino)tetradecanoate (11f):** In a 300-mL roundbottom flask, (*R,E*)-*N*-dodecylidene-2-methylpropane-2-sulfinamide (**9f**) (6.56 g, 22.81mmol, 1 equiv) was dissolved in THF (54 mL, 0.42 M). A solution of Reformatsky reagent (**10**) in THF (16.01 g, 61.50 mmol, 0.5 M, 2.7 equiv) was added to the reaction mixture and stirred for 4 h at rt. The reaction mixture was quenched with satd aq NH_4Cl, diluted with H_2O, and extracted with EtOAc (2 x 125 mL). The combined organic extracts were washed with brine, dried (Na_2SO_4), filtered, and concentrated by rotary evaporation to afford the crude product. Purification by silica flash chromatography (15% EtOAc/hexanes) yielded β-sulfinamido ester **11f** as a colorless oil (5.238 g, 57%).

TLC: R_f 0.33 (30% EtOAc/hexanes, CAM, *p*-anisaldehyde). $[\alpha]^{23}$: –23.4201° (*c* 0.88=9, $CHCl_3$). **^{1}H NMR** (600 MHz, $CDCl_3$): δ 4.12 (d, *J* = 7.8 Hz, 1H), 3.50 (tq, *J* = 7.9, 5.4 Hz, 1H), 2.66 (dd, *J* = 15.7, 5.3 Hz, 1H), 2.51 (dd, *J* = 15.7, 5.4 Hz, 1H), 1.57 (dtd, *J* = 15.8, 8.0, 4.9 Hz, 2H), 1.50 (dq, *J* = 8.7, 4.1 Hz, 1H), 1.45 (s, 9H), 1.42 – 1.36 (m, 1H), 1.25 (s, 17H), 1.22 (s, 9H), 0.88 (t, *J* = 7.0 Hz, 3H). **^{13}C NMR** (151 MHz, $CDCl_3$): δ 170.30, 80.12, 54.72, 52.73, 40.66, 34.29, 30.89, 28.60, 28.54, 28.50, 28.32, 28.27, 27.11, 24.84, 21.70, 21.67, 13.10. **HRMS** (ESI) *m/z* calcd for $C_{22}H_{45}NO_3S$ ($[M+H]^+$) 404.3198; found 404.3199.

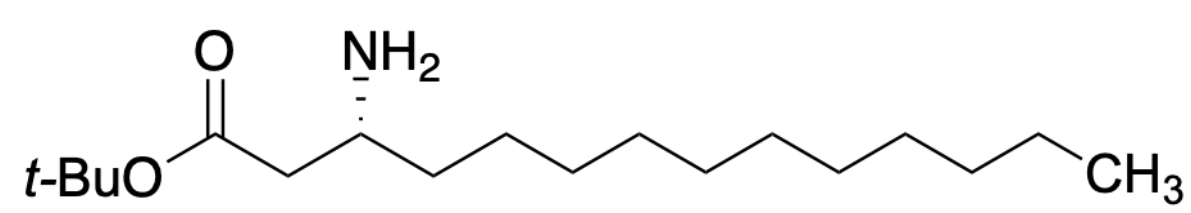

***tert*-Butyl (*3R*)-3-aminotetradecanoate (12f):** In a 250-mL roundbottom flask, *tert*-butyl (*R*)-3-(((*R*)-*tert*-butylsulfinyl)amino)tetradecanoate amine (**11f**) (5.2094 g, 12.90 mmol, 1 equiv), THF (100 mL, 0.13 M), and HCl in Et_2O (2.0 M, 65 mL, 130 mmol, 10 equiv) were added and stirred for 4.5 h at rt. The reaction mixture was concentrated by rotary evaporation to afford the crude HCl salt. The resulting HCl salt was dissolved in EtOAc, washed with satd aq $NaHCO_3$ until pH 9, and the aq phase was back-extracted with EtOAc (2 x 50 mL). The combined organic extracts were dried (Na_2SO_4), filtered, and concentrated by rotary evaporation to afford β-amino ester **12f** as a yellow oil (3.6854 g, 95%), which was used without further purification.

TLC: R_f 0.31 (100% EtOAc, ninhydrin). $[\alpha]^{22}$: 3.0067° (*c* 0.90, $CHCl_3$). **HRMS** (ESI) *m/z* calcd for $C_{18}H_{37}NO_2S$ ($[M+H]^+$) 300.2903; found 300.2899.

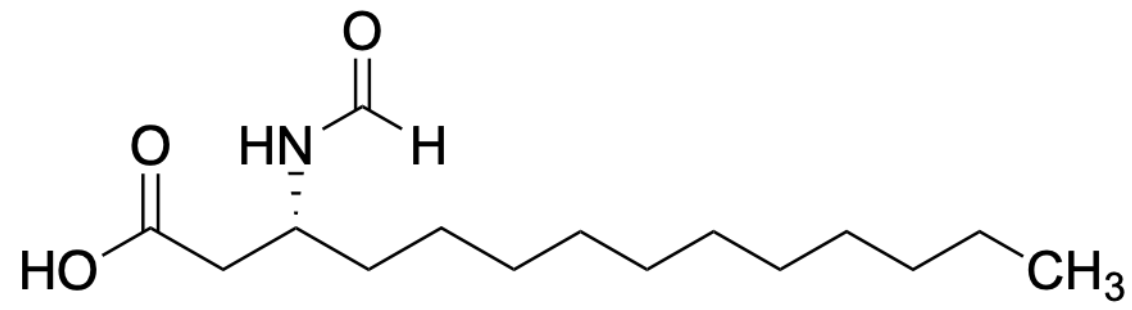

(*3R*)-3-Formamidotetradecanoic acid (13f): In a 50-mL roundbottom flask containing *tert*-butyl (*3R*)-3-aminotetradecanoate (**12e**) (3.6698 g, 12.25 mmol, 1 equiv), formic acid (22.2 mL, 588.3 mmol, 48 equiv, 3:2 ratio of formic acid to acetic anhydride), and acetic anhydride (14.8 mL, 156.5 mmol, 13 equiv) were heated together at 80 °C for 20.5 h. The reaction mixture was concentrated by rotary evaporation to afford the crude product. Purification by silica flash chromatography (50–75% EtOAc/hexanes + 1% AcOH) yielded β-formamido acid (**13f**) as a white powder (1.3412 g, 40%).

TLC: R_f 0.43 (75% EtOAc/hexanes + 1% AcOH, CAM, $KMnO_4$). **^{1}H NMR** (600 MHz, MeOD): δ 8.03 (d, *J* = 0.8 Hz, 1H), 4.26 (ddt, *J* = 8.7, 6.8, 3.5 Hz, 1H), 2.48 (d, *J* = 6.6 Hz, 2H), 1.58 (ddd, *J* = 14.4, 8.6, 4.9 Hz, 1H), 1.55 – 1.44 (m, 1H), 1.44 – 1.24 (m, 19H), 0.90 (t, *J* = 7.0 Hz, 3H). **^{13}C NMR** (151 MHz, MeOD): δ 173.28, 173.16, 165.66, 161.83, 56.07, 49.95, 48.16, 45.85, 45.10, 39.92, 39.02, 38.85, 34.66, 33.90, 33.87, 31.69, 31.67, 29.43, 29.40, 29.38, 29.36, 29.35, 29.34, 29.28, 29.27, 29.23, 29.10, 29.07, 28.99, 28.90, 25.77, 25.65, 25.60, 22.35, 22.33, 13.04, 13.03. **HRMS** (ESI) *m/z* calcd for $C_{19}H_{33}N_6O_5$ ($[M+H]^+$) 270.2077; found 270.2069.

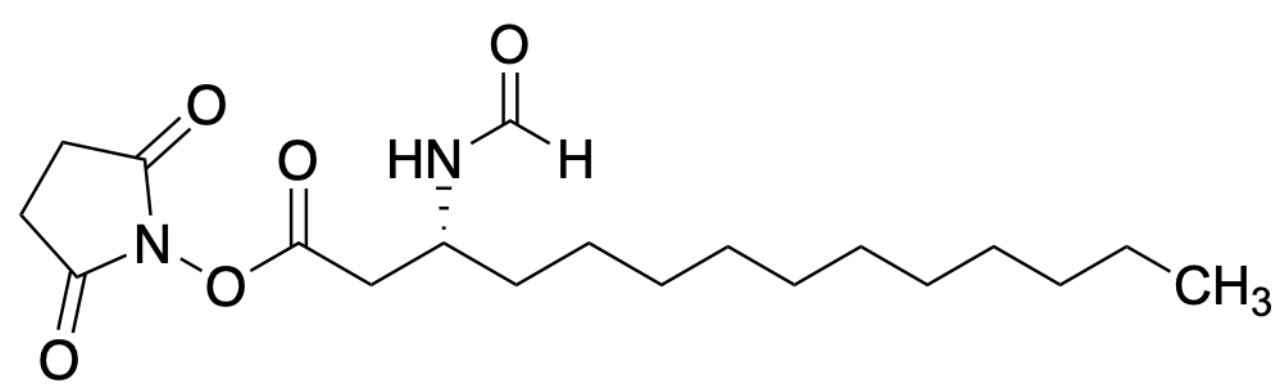

2,5-Dioxopyrrolidin-1-yl (*3R*)-3-formamidotetradecanoate (14f): In a 10-mL roundbottom flask, (*3R*)-3-formamidotetradecanoic acid (**13f**) (0.1276 g, 0.6529 mmol, 1 equiv) and EDC (0.2724 g, 1.420 mmol, 3 equiv) were suspended in THF (3 mL, 0.16 M) and cooled to 0 °C for 10 min. *N*-hydroxysuccinimide (0.1753 g, 1.523 mmol, 3.2 equiv) was added to the stirring solution in one portion. The mixture was stirred at 0 °C for 30 min, warmed to 22 °C, and stirred for 19 h. The mixture was filtered through a pad of celite and washed with Et_2O. The combined filtrates were concentrated by rotary evaporation to afford the crude product. Purification by silica flash chromatography (75% EtOAc/hexanes) yielded β-formamido NHS ester **14f** as a white powder (0.1004 g, 58%).

TLC: R_f 0.33 (75% EtOAc/hexanes, CAM, $KMnO_4$). **^{1}H NMR** (600 MHz, $CDCl_3$): δ 8.18 (d, *J* = 1.6 Hz, 1H), 6.01 (d, *J* = 9.2 Hz, 1H), 4.42 (dtt, *J* = 9.5, 7.4, 5.0 Hz, 1H), 2.94 (dd, *J* = 15.0, 5.3 Hz, 1H), 2.86 (p, *J* = 5.1 Hz, 4H), 2.80 (dd, *J* = 15.0, 4.8 Hz, 1H), 1.63 (q, *J* = 7.5 Hz, 2H), 1.40 – 1.33 (m, 2H), 1.26 (d, *J* = 10.0 Hz, 17H), 0.88 (t, *J* = 7.0 Hz, 3H). **^{13}C NMR** (151 MHz, $CDCl_3$): δ 166.34, 165.96, 163.80, 160.80, 49.30, 44.71, 38.10, 35.78, 35.15, 33.27, 31.92, 29.62, 29.60, 29.53, 29.50, 29.44, 29.40, 29.35, 29.33, 29.15, 29.03, 26.18, 25.89, 25.60, 25.57, 22.70, 14.13. **HRMS** (ESI) *m/z* calcd for $C_{19}H_{32}N_2O_5$ ($[M+H+Na]^+$) 391.2206; found 391.2209.

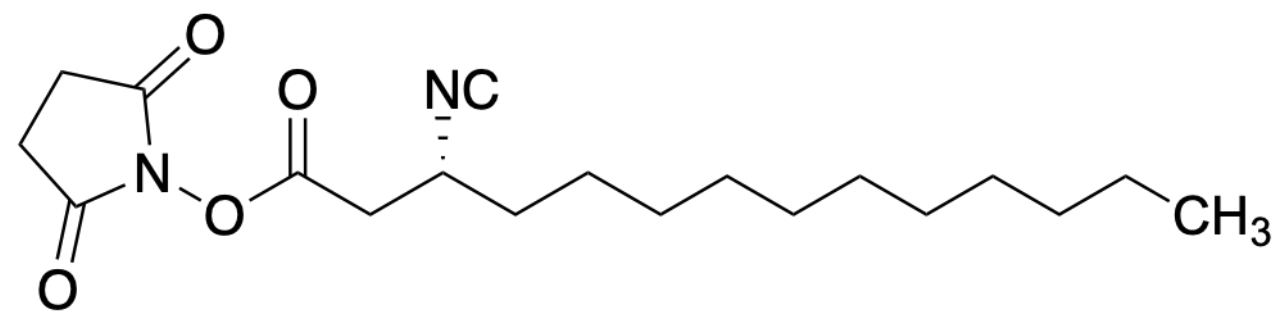

2,5-Dioxopyrrolidin-1-yl (*3R*)-3-isocyanotetradecanoate (15f): In a 5-mL roundbottom flask, 2,5-dioxopyrrolidin-1-yl (*3R*)-3-formamidotetradecanoate (**14f**) (0.0986 g, 0.2675 mmol, 1 equiv) and diisopropylamine (0.151 mL, 1.069 mmol, 4 equiv) were dissolved in CH_2Cl_2 (1.4 mL, 0.2 M total) and cooled to –30 °C for 15 min. $POCl_3$ (0.045 mL, 0.4815 mmol, 1.8 equiv) was added to the solution and the mixture was stirred at –30 °C for 4 h. The reaction mixture was quenched with satd aq $NaHCO_3$ and extracted with CH_2Cl_2 (1 x 60 mL). The combined organic extracts were washed with brine, dried (Na_2SO_4), filtered, and concentrated by rotary evaporation to afford the crude product. Purification by silica flash chromatography (40% EtOAc/hexanes) yielded β-isocyano NHS ester(**15f**) as a white powder (0.0645 g, 69%).

TLC: R_f 0.48 (40% EtOAc/hexanes, UV, $KMnO_4$). **^{1}H NMR** (600 MHz, $CDCl_3$): δ 4.03 (p, *J* = 6.8 Hz, 1H), 3.06 (dd, *J* = 16.4, 7.0 Hz, 1H), 2.90 (dd, *J* = 16.4, 6.6 Hz, 1H), 2.88 – 2.81 (m, 4H), 1.76 – 1.69 (m, 2H), 1.57 (s, 1H), 1.56 – 1.53 (m, 1H), 1.45 (ddd, *J* = 16.4, 9.7, 4.2 Hz, 1H), 1.38 – 1.28 (m, 6H), 1.26 (s, 11H), 0.88 (t, *J* = 7.0 Hz, 3H). **^{13}C NMR** (151 MHz, $CDCl_3$): δ 167.53, 163.76, 157.05, 49.52, 35.97, 33.38, 30.88, 28.57, 28.56, 28.45, 28.31, 28.29, 27.69, 24.54, 24.40, 21.67, 13.10. **HRMS** (ESI) *m/z* calcd for $C_{19}H_{30}N_2O_4$ ($[M+H+Na]^+$) 373.2105; found 373.2103.

D. SYNTHESIS OF DIISONITRILES 7, 22b–f, 23a,f

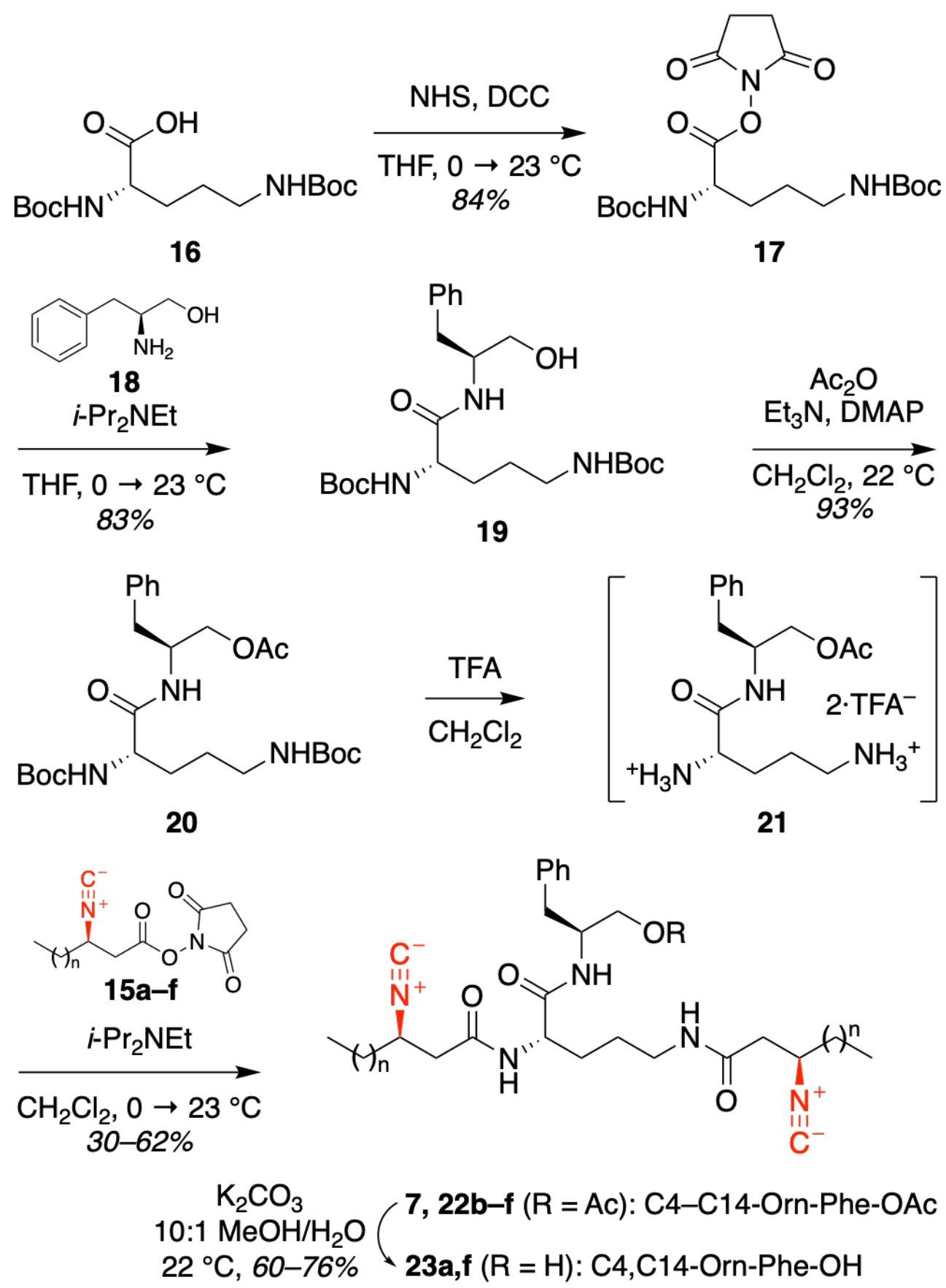

Supplementary Figure S12 ***(Figure 3a in manuscript).*** **Synthesis of Orn-Phe-OH backbone and coupling of β-isocyanoacyl sidechains to afford diisonitrile analogues with varied chain lengths.** Boc = *t*-butoxycarbonyl; DCC = dicyclohexylcarbodiimide; DMAP = 4-(dimethylamino)pyridine; TFA = trifluoroacetic acid.

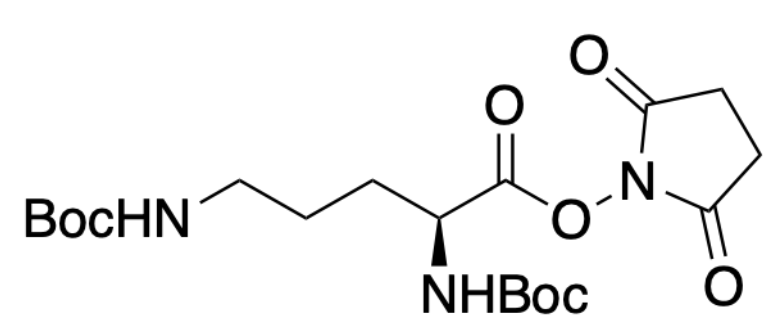

***N*^{2},*N*^{5}-Bis(*tert*-butoxycarbonyl)-L-ornithine *N*-hydroxysuccinimide ester (17, Boc-Orn[Boc]-NHS).** In a 50-mL roundbottom flask, *N*^{2},*N*^{5}-bis(*tert*-butoxycarbonyl)-L-ornithine (**1**) (2.0099 g, 6.04 mmol, 1.0 equiv) and dicyclohexylcarbodiimide (1.2932 g, 6.26 mmol, 1.03 equiv) were suspended in 20 mL anhyd THF and cooled to 0 °C for 10 min. *N*-Hydroxysuccinimide (699 mg, 6.07 mmol, 1.005 equiv) was added to the stirring solution in one portion. The mixture was stirred at 0 °C for 30 min, warmed to 22 °C, and stirred for 23 h. The mixture was filtered through a pad of celite and the solid white residue was washed with Et_2O (40 mL). The combined filtrates were concentrated by rotary evaporation to afford the crude product as an off-white solid. Purification by silica flash chromatography (50% EtOAc in hexanes) yielded NHS ester **2** (2.1874 g, 5.09 mmol, 84%) as a white solid.

TLC: R_f 0.43 (50% EtOAc in hexanes). **^{1}H-NMR** (600 MHz): δ 5.22 (d, *J* = 8.7 Hz, 1H), 4.77 (s, 1H), 4.64 (q, *J* = 7.2 Hz, 1H), 3.14 (q, *J* = 6.6 Hz, 2H), 2.81 (s, 4H), 1.99 – 1.90 (m, 1H), 1.86 – 1.78 (m, 1H), 1.63 (br s, 2H), 1.40 (m, 18H). **^{13}C-NMR** (150 MHz): δ 168.8, 168.4, 156.1, 155.0, 80.6, 79.2, 51.8, 39.9, 29.9, 28.5, 28.3, 25.6. **HRMS** (ESI) *m/z* calcd for $C_{19}H_{31}N_3O_8Na$ ($[M+Na]^+$) 452.2009; found 452.2000.

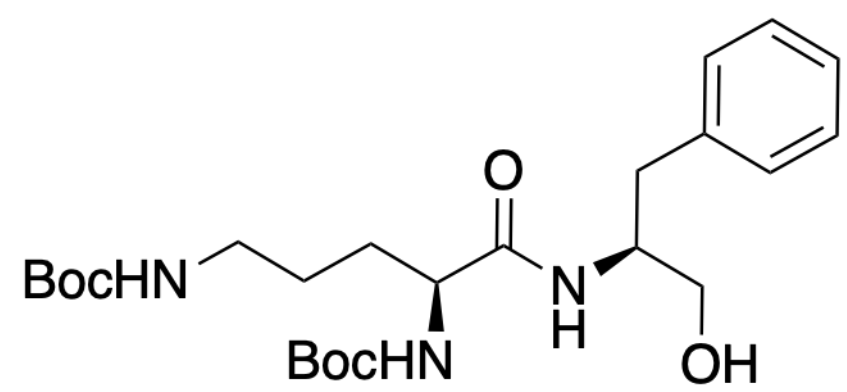

***N*^{2},*N*^{5}-Bis(*tert*-butoxycarbonyl)-L-ornithyl-L-phenylalaninol (19, Boc-Orn[Boc]-Phe-OH).** In a 25-mL roundbottom flask, L-phenylalaninol (148 mg, 0.978 mmol, 1.0 equiv) and DIPEA (405 µL, 2.94 mmol, 3.0 equiv) were dissolved in 10 mL anhyd THF and cooled to 0 °C for 10 min. *N*^{2},*N*^{5}-Bis(*tert*-butoxycarbonyl)-L-ornithine *N*-hydroxysuccinimide ester (**17**) (495 mg, 1.15 mmol, 1.18 equiv) was added to the stirring solution in one portion. The mixture was stirred at 0 °C for 5 min, warmed to 22 °C, and stirred for 17 h. The mixture was diluted with H_2O and extracted with CH_2Cl_2 (3 x 20 mL). The combined organic extracts were washed with brine, dried (Na_2SO_4), filtered, and concentrated by rotary evaporation to afford the crude product. Purification by silica flash chromatography (50% EtOAc in hexanes) yielded protected dipeptide alcohol **19** (378 mg, 0.81 mmol, 83%) as a white solid.

TLC: R_f 0.25 (50% EtOAc in hexanes). **^{1}H-NMR** (600 MHz): δ 7.30 – 7.25 (m, 2H), 7.24 – 7.19 (m, 3H), 6.59 (d, *J* = 8.2 Hz, 1H), 5.24 (d, *J* = 7.6 Hz, 1H), 4.80 – 4.74 (m, 1H), 4.20 – 4.08 (m, 2H), 3.66 (dd, *J* = 11.3, 3.5 Hz, 1H), 3.54 (dd, *J* = 11.5, 4.8 Hz, 1H), 3.21 (s, 2H), 3.01 (dt, *J* = 13.8, 6.7 Hz, 1H), 2.87 (td, *J* = 12.2, 7.5 Hz, 2H), 1.80 – 1.71 (m, 1H), 1.62 – 1.46 (m, 2H), 1.42 (s, 18H). **^{13}C-NMR** (150 MHz): δ 172.3, 156.6, 155.9, 137.9, 129.4, 128.7, 126.7, 80.2, 79.6, 63.6, 54.0, 53.1, 39.8, 37.1, 30.1, 28.6, 28.4, 26.2. **HRMS** (ESI) *m/z* calcd for $C_{24}H_{39}N_3O_6Na$ ($[M+Na]^+$) 488.2737; found 488.2749.

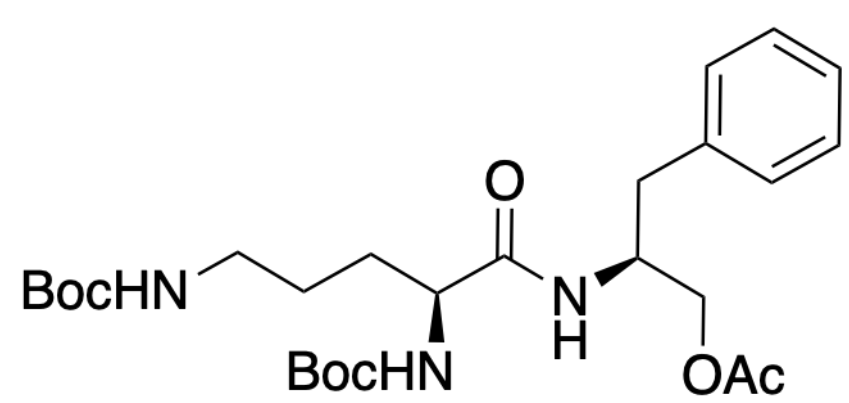

***N*^{2},*N*^{5}-Bis(tert-butoxycarbonyl)-L-ornithyl-L-phenylalaninol acetate (20, Boc-Orn[Boc]-Phe-OAc).** In a 10-mL roundbottom flask, *N*^{2},*N*^{5}-bis(*tert*-butoxycarbonyl)-L-ornithyl-L-phenylalaninol (**19**) (324 mg, 0.696 mmol, 1.0 equiv), 4-(dimethylamino)pyridine (18 mg, 0.15 mmol, 0.21 equiv), and triethylamine (194 µL, 1.39 mmol, 2.0 equiv) were dissolved in 5 mL anhyd CH_2Cl_2 at rt. Acetic anhydride (91 µL, 0.962 mmol, 1.38 equiv) was added to the stirring solution and the mixture was stirred at 22 °C for 21 h. The reaction was quenched by addition of satd aq $NaHCO_3$ and extracted with CH_2Cl_2 (3 x 20 mL). The combined organic extracts were washed with brine, dried (Na_2SO_4), filtered, and concentrated by rotary evaporation to afford the crude product. Purification by silica flash chromatography (50% EtOAc in hexanes) yielded protected dipeptide acetate **20** (325 mg, 0.64 mmol, 93%) as a white solid.

TLC: R_f 0.50 (50% EtOAc in hexanes). **^{1}H-NMR** (600 MHz): δ 7.29 (t, *J* = 7.5 Hz, 2H), 7.24 – 7.17 (m, 3H), 6.50 (s, 1H), 5.08 (s, 1H), 4.67 (s, 1H), 4.40 (s, 1H), 4.12 (mf, 1H), 4.09 – 3.99 (m, 2H), 3.23 (s, 1H), 3.04 (dq, *J* = 12.5, 6.1 Hz, 1H), 2.84 (qd, *J* = 13.8, 7.1 Hz, 2H), 2.07 (s, 3H), 1.79 – 1.69 (m, 1H), 1.58 – 1.51 (m, 1H), 1.43 (m, 19H). **^{13}C-NMR** (150 MHz): δ 171.8, 171.3, 171.1, 156.5, 155.8, 137.1, 129.3, 128.8, 126.9, 80.1, 79.4, 64.8, 60.5, 53.6, 53.6, 49.7, 39.6, 37.6, 30.1, 28.6, 28.4, 26.4, 20.9, 14.3. **HRMS** (ESI) *m/z* calcd for $C_{26}H_{42}N_3O_7$ ([M+H]$^+$) 508.3023; found 508.3023.

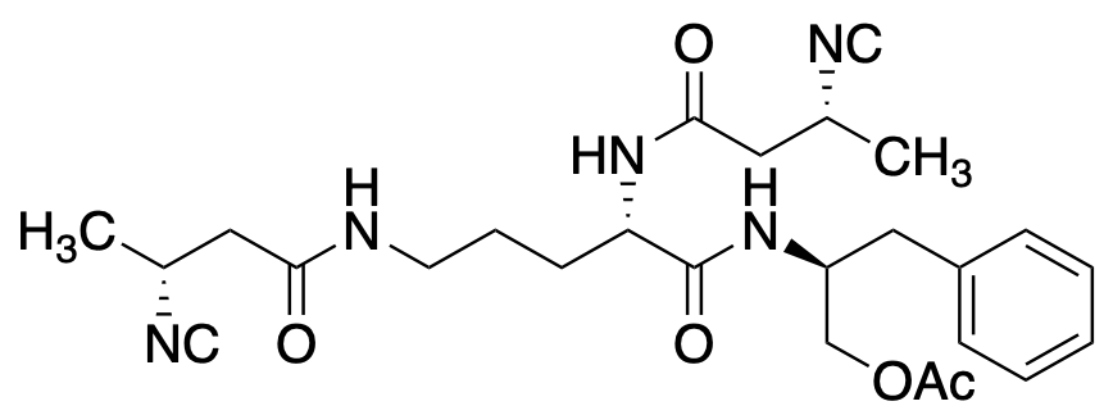

***N*^{2},*N*^{5}-Bis((*R*)-3-isocyanobutanoyl)-L-ornithyl-L-phenylalaninol acetate (7, C4-Orn-Phe-OAc).** In a 20-mL glass vial, *N*^{2},*N*^{5}-bis(*tert*-butoxycarbonyl)-L-ornithyl-L-phenylalaninol acetate (**20**) (350 mg, 0.689 mmol, 1.0 equiv) was dissolved in 8 mL anhyd CH_2Cl_2 and cooled to 0 °C. Trifluoroacetic acid (2 mL, 25.6 mmol, 37.1 equiv) was added and the mixture was stirred at 0 °C for 5 min, warmed to 22 °C, and stirred for 1 h. The mixture was concentrated by rotary evaporation to afford crude bis(trifluoroacetate) salt **21**, which was used without further purification.

In a 20-mL roundbottom flask, the crude bis(trifluoroacetate) salt **21** (369 mg, 0.689 mmol, 1.0 equiv) and DIPEA (1.08 mL, 6.20 mmol, 9 equiv) were dissolved in 5 mL anhyd CH_2Cl_2 and cooled to 0 °C. 2,5-Dioxopyrrolidin-1-yl (*R*)-3-isocyanobutanoate (**15a**), prepared as previously described,[4] (367 mg, 1.75 mmol, 2.53 equiv) was added to the stirring solution and the mixture was stirred at 0 °C for 5 min, warmed to 22 °C, and stirred for 17 h. The mixture was diluted with H_2O and extracted with CH_2Cl_2 (3x20 mL). The combined organic extracts were washed with brine, dried (Na_2SO_4), filtered, and concentrated by rotary evaporation to afford the crude product. Purification by silica flash chromatography (100% EtOAc) yielded diisonitrile **7** (212 mg, 0.43 mmol, 62%) as a colorless opaque solid.

TLC: R_f 0.18 (100% EtOAc). **^{1}H-NMR** (600 MHz, DMSO-d_6): δ 8.15 (d, *J* = 8.2 Hz, 1H), 8.02 (t, *J* = 5.7 Hz, 1H), 7.99 (d, *J* = 8.4 Hz, 1H), 7.27 (m, 2H), 7.20 (m, 3H), 4.25 (q, *J* = 7.6 Hz, 1H), 4.16 (q, *J* = 6.8 Hz, 1H), 4.06 (m, 2H), 4.01 (dd, *J* = 11.1, 4.7 Hz, 1H), 3.84 (dd, *J* = 11.1, 7.1 Hz, 1H), 3.03 (dh, *J* = 20.0, 6.7 Hz, 2H), 2.77 (dd, *J* = 13.9, 6.2 Hz, 1H), 2.72 (dd, *J* = 13.8, 7.8 Hz, 1H), 2.56 (dd, *J* = 14.6, 8.2 Hz, 1H), 2.49 – 2.35 (m, 3H), 1.99 (s, 3H), 1.56 (m, 1H), 1.50 – 1.33 (m, 1H), 1.30 (t, *J* = 7.1 Hz, 6H). **^{13}C-NMR** (150 MHz, DMSO-d_6): δ 171.1, 170.3, 168.0, 167.9, 155.0, 154.9, 154.9, 137.9, 129.1, 128.2, 126.2, 64.6, 52.2, 48.9, 46.99, 46.95, 46.92, 42.2, 42.0, 38.2, 36.6, 29.9, 25.5, 20.99, 20.95, 20.6. **HRMS** (ESI) *m/z* calcd for $C_{26}H_{35}N_5O_5Na$ ([M+Na]$^+$) 520.2536; found 520.2536.

[4] Xu, Y.; Tan, D. S. *Org. Lett.* **2019**, *21*, 8731-8735.

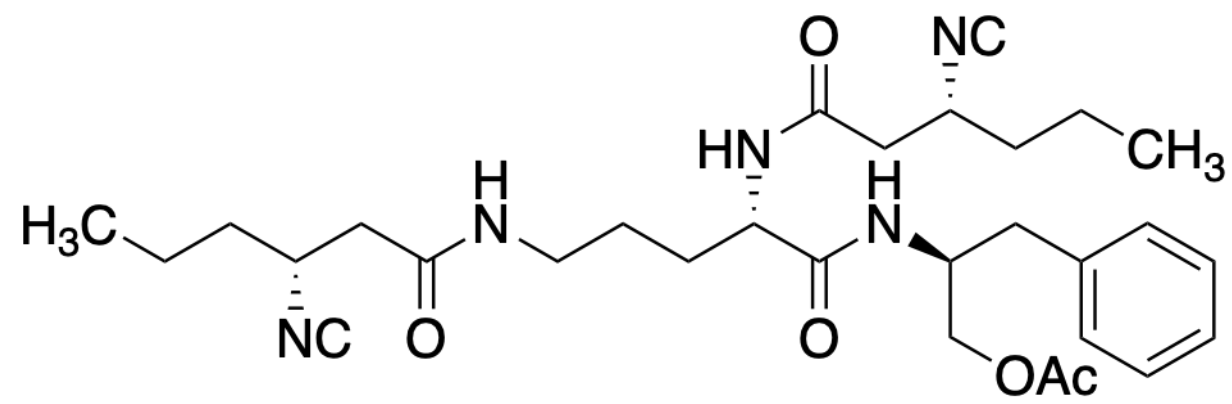

(*2S*)-2-[(*2S*)-2,5-Bis[(*3R*)-3-isocyanohexanamido]pentanamido]-3-phenylpropyl acetate (22b, C6-Orn-Phe-OAc): In a 5-mL roundbottom flask, 2,5-dioxopyrrolidin-1-yl (*R*)-3-isocyanohexanoate (**15b**) (0.0252 g, 0.1057 mmol, 2.6 equiv) and DIPEA (0.045 mL, 0.2582 mmol, 6 equiv) were dissolved in anhyd CH_2Cl_2 (0.125 M, 0.330 mL, 3:1 CH_2Cl_2/MeOH) and cooled to 0 °C for 10 min. The crude bis(trifluoroacetate) salt **21** (0.0221 g, 0.04127 mmol, 1 equiv) was dissolved in anhyd MeOH (0.375 M, 0.110 mL, 3:1 CH_2Cl_2/MeOH) and added to the stirring solution. The mixture was stirred at 0 °C for 5 min, warmed to 22 °C, and stirred for 5 h. The mixture was diluted with H_2O and extracted with CH_2Cl_2 (1 x 30 mL). The combined organic extracts were washed with brine, dried (Na_2SO_4), filtered, and concentrated by rotary evaporation to afford the crude product. Purification by silica flash chromatography (80% EtOAc/hexanes) yielded diisonitrile **22b** as a white powder (0.0097 g, 42%).

TLC: R_f 0.44 (100% EtOAc/hexanes, UV, $KMnO_4$). **^{1}H NMR** (600 MHz, $CDCl_3$): δ 7.28 (t, J = 7.4 Hz, 2H), 7.22 (d, J = 7.2 Hz, 1H), 7.18 (d, J = 7.4 Hz, 2H), 6.78 (d, J = 8.2 Hz, 1H), 6.55 (d, J = 8.5 Hz, 1H), 6.34 (dd, J = 7.8, 4.5 Hz, 1H), 4.53 (td, J = 8.7, 3.5 Hz, 1H), 4.41 – 4.34 (m, 1H), 4.06 (dd, J = 13.3, 5.2 Hz, 3H), 3.64 (dtd, J = 12.1, 8.3, 3.4 Hz, 1H), 3.08 (dq, J = 14.3, 4.8 Hz, 1H), 2.87 (dd, J = 13.9, 6.9 Hz, 1H), 2.81 (dd, J = 13.9, 7.5 Hz, 1H), 2.46 (dd, J = 14.2, 9.9 Hz, 1H), 2.43 – 2.37 (m, 2H), 2.08 (s, 2H), 1.86 (ddd, J = 16.6, 6.7, 3.7 Hz, 1H), 1.66 (q, J = 11.2 Hz, 4H), 1.62 – 1.55 (m, 4H), 1.54 – 1.41 (m, 3H), 1.25 (s, 5H), 0.98 (td, J = 7.1, 3.2 Hz, 5H), 0.88 (t, J = 6.8 Hz, 1H). **^{13}C NMR** (151 MHz, $CDCl_3$): δ 171.22, 170.96, 169.12, 168.76, 155.75, 155.29, 136.91, 129.25, 128.74, 128.62, 128.55, 126.76, 64.70, 52.58, 52.39, 52.34, 50.04, 42.77, 42.57, 38.45, 37.40, 37.29, 36.72, 36.67, 31.94, 30.37, 29.71, 29.67, 29.51, 29.37, 25.70, 22.71, 20.97, 18.98, 18.93, 14.14, 13.35, 13.32, 1.03. **HRMS** (ESI) *m/z* calcd for $C_{30}H_{43}N_5O_5$ ($[M+H+Na]^+$) 576.3162; found 576.3155.

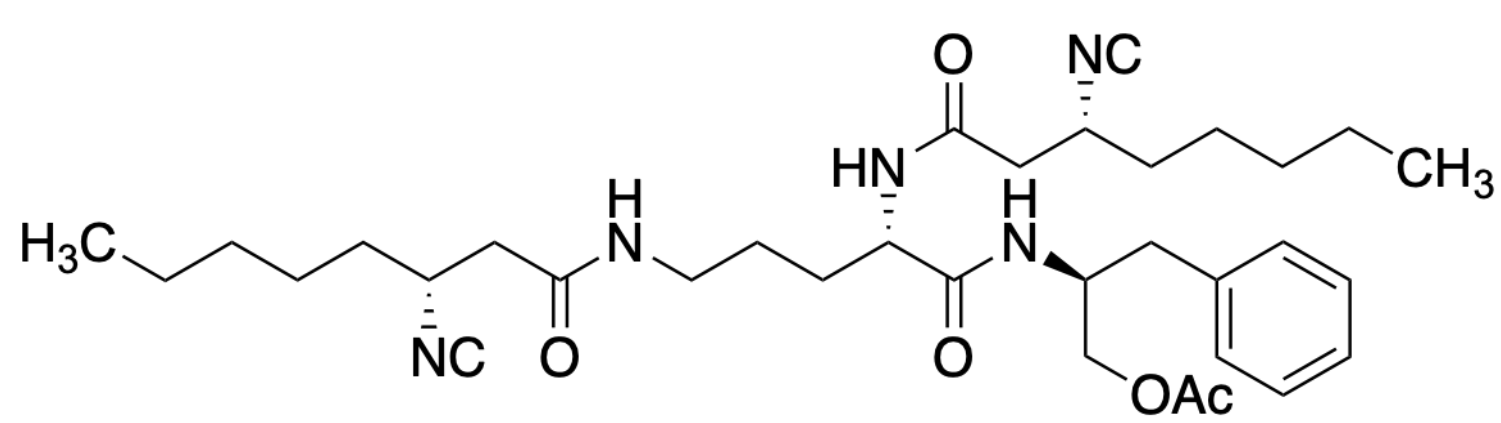

(*2S*)-2-[(*2S*)-2,5-Bis[(*3R*)-3-isocyanooctanamido]pentanamido]-3-phenylpropyl acetate (22c): In a 5-mL roundbottom flask, 2,5-dioxopyrrolidin-1-yl (*R*)-3-isocyanooctanoate (**15c**) (0.0221 g, 0.08299 mmol, 2.5 equiv) and DIPEA (0.035 mL, 0.2008 mmol, 6 equiv) were dissolved in anhyd CH_2Cl_2 (0.12 M, 0.270 mL, 3:1 CH_2Cl_2/MeOH) and cooled to 0 °C for 10 min. The crude bis(trifluoroacetate) salt **21** (0.0176 g, 0.03287 mmol, 1 equiv) was dissolved in anhyd MeOH (0.365 M , 0.90 mL, 3:1 CH_2Cl_2/MeOH) and added to the stirring solution. The mixture was stirred at 0 °C for 5 min, warmed to 22 °C, and stirred for 4 h. The mixture was diluted with H_2O and extracted with CH_2Cl_2 (1 x 50 mL). The combined organic extracts were washed with brine, dried (Na_2SO_4), filtered, and concentrated by rotary evaporation to afford the crude product. Purification by silica flash chromatography (70% EtOAc/hexanes) yielded diisonitrile **22c** as a white powder (0.0061 g, 30%).

TLC: R_f 0.22 (70% EtOAc/hexanes, UV, $KMnO_4$). **^{1}H NMR** (600 MHz, $CDCl_3$): δ 7.29 (t, *J* = 7.5 Hz, 1H), 7.21 – 7.16 (m, 1H), 4.08 (d, *J* = 5.2 Hz, 1H), 4.03 (dq, *J* = 9.0, 4.8 Hz, 1H), 2.46 (dd, *J* = 14.1, 9.9 Hz, 1H), 2.40 (dt, *J* = 13.5, 6.4 Hz, 1H), 2.08 (s, 1H), 1.71 – 1.57 (m, 4H), 1.53 (tq, *J* = 9.2, 4.4 Hz, 2H), 1.44 (td, *J* = 9.8, 4.4 Hz, 1H), 1.34 (dp, *J* = 12.2, 3.8 Hz, 4H), 1.25 (s, 20H), 0.94 – 0.85 (m, 6H), 0.85 – 0.78 (m, 2H), 0.75 – 0.68 (m, 1H). **^{13}C NMR** (151 MHz, $CDCl_3$): δ 171.57, 171.32, 169.48, 169.12, 156.06, 155.59, 137.26, 129.60, 128.97, 127.11, 65.04, 53.21, 52.97, 52.68, 50.42, 43.14, 42.95, 38.79, 37.75, 35.06, 35.00, 34.05, 32.29, 31.35, 31.32, 30.74, 30.52, 30.40, 30.09, 30.06, 30.02, 29.72, 27.06, 26.05, 25.71, 25.65, 23.54, 23.06, 22.79, 22.77, 21.31, 14.55, 14.48, 14.29, 1.38. **HRMS** (ESI) *m/z* calcd for $C_{34}H_{51}N_5O_5$ ($[M+H+Na]^+$) 632.3790; found 632.3788.

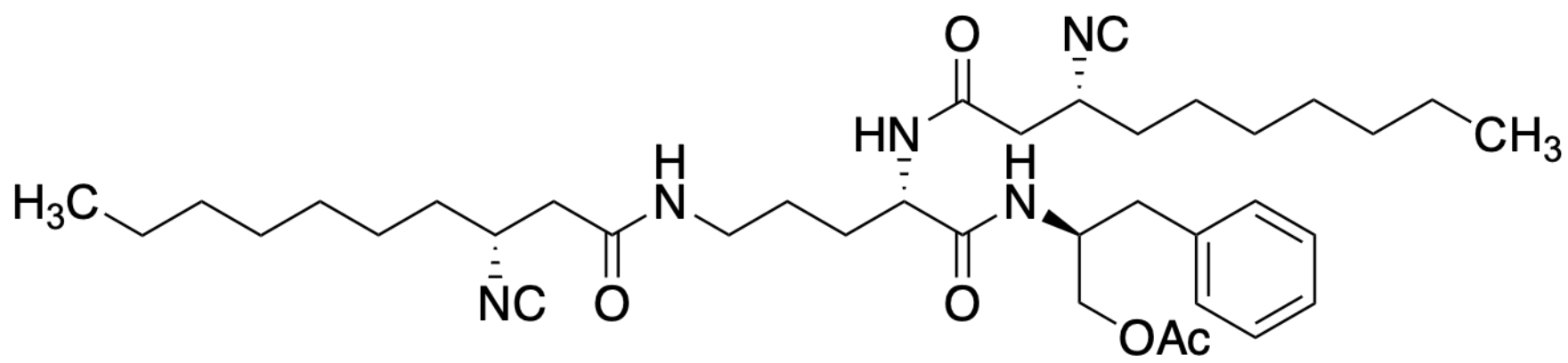

(*2S*)-2-[(*2S*)-2,5-Bis[(*3R*)-3-isocyanodecanamido]pentanamido]-3-phenylpropyl acetate (22d): In a 5-mL roundbottom flask, 2,5-dioxopyrrolidin-1-yl (*R*)-3-isocyanodecanoate (**15d**) (0.0245 g, 0.0822 mmol, 2.7 equiv) and DIPEA (0.032 mL, 0.1836 mmol, 6 equiv) were dissolved in anhyd CH_2Cl_2 (0.13 M, 0.240 mL, 3:1 CH_2Cl_2/MeOH) and cooled to 0 °C for 10 min. The crude bis(trifluoroacetate) salt **21** (0.0167 g, 0.0312 mmol, 1 equiv) was dissolved in anhyd MeOH (0.39 M , 0.080 mL, 3:1 CH_2Cl_2/MeOH) and added to the stirring solution. The mixture was stirred at 0 °C for 5 min, warmed to 22 °C, and stirred for 5.5 h. The mixture was diluted with H_2O and extracted with CH_2Cl_2 (1 x 30 mL). The combined organic extracts were washed with brine, dried (Na_2SO_4), filtered, and concentrated by rotary evaporation to afford the crude product. Purification by silica flash chromatography (70% EtOAc/hexanes) yielded diisonitrile **22d** as a white powder (0.0077 g, 37%).

TLC: R_f 0.29 (70% EtOAc/hexanes, UV, $KMnO_4$). **^{1}H NMR** (600 MHz, $CDCl_3$): δ 7.28 (t, *J* = 7.4 Hz, 2H), 7.22 (t, *J* = 7.3 Hz, 1H), 7.19 (d, *J* = 7.5 Hz, 2H), 6.76 (d, *J* = 8.2 Hz, 1H), 6.52 (d, *J* = 8.5 Hz, 1H), 6.32 (dd, *J* = 7.8, 4.4 Hz, 1H), 4.52 (td, *J* = 8.7, 3.4 Hz, 1H), 4.41 – 4.34 (m, 1H), 4.10 – 4.05 (m, 2H), 4.03 (dq, *J* = 9.0, 4.7 Hz, 1H), 3.65 (dtd, *J* = 12.6, 8.7, 3.2 Hz, 1H), 3.08 (dq, *J* = 14.5, 4.9 Hz, 1H), 2.87 (dd, *J* = 13.9, 6.8 Hz, 1H), 2.84 – 2.79 (m, 1H), 2.46 (dd, *J* = 14.2, 9.9 Hz, 1H), 2.40 (q, *J* = 5.4 Hz, 2H), 2.34 (d, *J* = 8.7 Hz, 4H), 2.08 (s, 2H), 1.88 (dd, *J* = 10.3, 5.8 Hz, 1H), 1.71 – 1.57 (m, 7H), 1.52 (qt, *J* = 9.1, 4.4 Hz, 3H), 1.48 – 1.39 (m, 2H), 1.38 – 1.18 (m, 18H), 0.89 (t, *J* = 6.7 Hz, 6H). **^{13}C NMR** (151 MHz, $CDCl_3$): δ 170.19, 169.92, 168.10, 167.73, 154.67, 154.19, 135.88, 128.22, 127.59, 125.73, 63.67, 51.84, 51.60, 51.31, 49.03, 41.77, 41.58, 37.41, 36.37, 33.74, 33.68, 30.68, 30.67, 29.36, 28.68, 28.04, 28.02, 27.81, 27.77, 24.69, 24.67, 24.62, 21.67, 21.58, 21.58, 19.94, 13.05. **HRMS** (ESI) *m/z* calcd for $C_{38}H_{59}N_5O_5$ ($[M+H]^+$) 688.4414; found 688.4417.

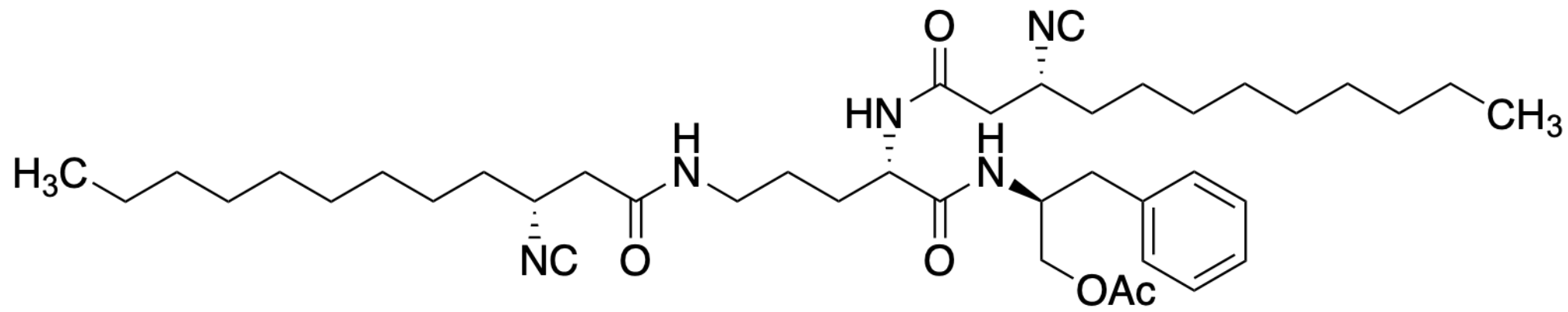

(2*S*)-2-[(2*S*)-2,5-Bis[(3*R*)-3-isocyanododecanamido]pentanamido]-3-phenylpropyl acetate (22e): In a 5-mL roundbottom flask, 2,5-dioxopyrrolidin-1-yl (*R*)-3-isocyanododecanoate (**15e**) (0.0102 g, 0.0032 mmol, 2.5 equiv) and DIPEA (0.013 mL, 0.0746 mmol, 6 equiv) were dissolved in anhyd CH_2Cl_2 (0.04 M, 0.300 mL, 3:1 CH_2Cl_2/MeOH) and cooled to 0 °C for 10 min. The crude bis(trifluoroacetate) salt **21** (0.0067 g, 0.01251 mmol, 1 equiv) was dissolved in anhyd MeOH (0.125 M , 0.100 mL, 3:1 CH_2Cl_2/MeOH) and added to the stirring solution. The mixture was stirred at 0 °C for 5 min, warmed to 22 °C, and stirred for 6.5 h. The mixture was diluted with H_2O and extracted with CH_2Cl_2 (1 x 30 mL). The combined organic extracts were washed with brine, dried (Na_2SO_4), filtered, and concentrated by rotary evaporation to afford the crude product. Purification by silica flash chromatography (70% EtOAc/hexanes) yielded diisonitrile **22e** as a white powder (0.0033 g, 37%).

TLC: R_f 0.21 (70% EtOAc/hexanes, UV, $KMnO_4$). **^{1}H NMR** (500 MHz, $CDCl_3$): δ 7.29 (d, *J* = 7.4 Hz, 1H), 7.24 – 7.20 (m, 1H), 7.20 – 7.15 (m, 2H), 6.75 (d, *J* = 8.2 Hz, 1H), 6.53 (d, *J* = 8.5 Hz, 1H), 6.32 (dd, *J* = 7.7, 4.5 Hz, 1H), 4.52 (td, *J* = 8.6, 3.5 Hz, 1H), 4.43 – 4.33 (m, 1H), 4.07 (d, *J* = 5.2 Hz, 2H), 4.04 (q, *J* = 7.6 Hz, 2H), 3.64 (dtd, *J* = 12.0, 8.4, 3.4 Hz, 1H), 3.08 (dq, *J* = 14.2, 4.9 Hz, 1H), 2.91 – 2.77 (m, 2H), 2.46 (dd, *J* = 14.1, 9.9 Hz, 1H), 2.43 – 2.36 (m, 2H), 2.08 (s, 3H), 1.91 – 1.82 (m, 1H), 1.72 – 1.57 (m, 6H), 1.52 (dt, *J* = 9.4, 4.6 Hz, 3H), 1.47 – 1.39 (m, 2H), 1.38 – 1.19 (m, 25H), 0.88 (t, *J* = 6.8 Hz, 6H). **^{13}C NMR** (126 MHz, $CDCl_3$): δ 171.20, 170.95, 169.12, 168.75, 136.92, 129.25, 128.62, 126.76, 64.70, 52.35, 50.06, 42.79, 42.60, 38.45, 37.42, 34.79, 34.73, 31.86, 30.39, 29.48, 29.42, 29.40, 29.27, 28.89, 28.86, 25.72, 25.66, 22.67, 20.96, 14.11. **HRMS** (ESI) *m/z* calcd for $C_{42}H_{67}N_5O_5$ ($[M+H]^+$) 744.504; found 744.5028.

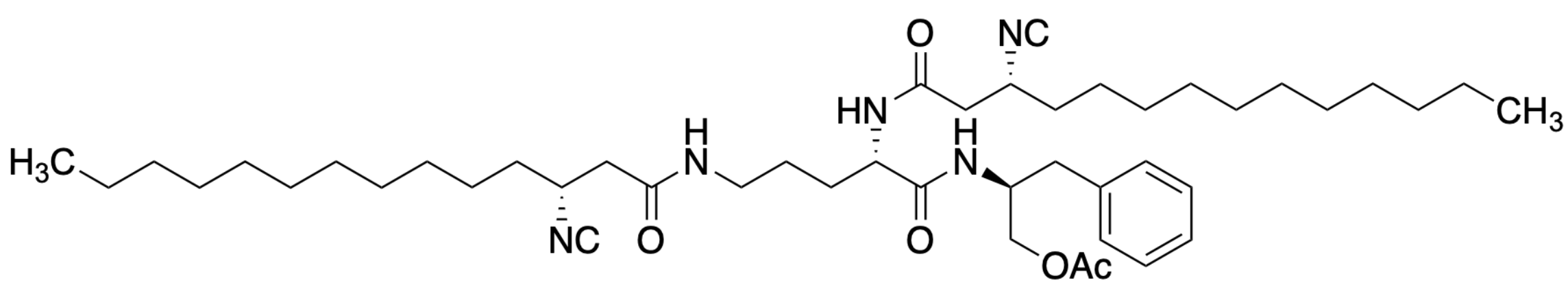

(2*S*)-2-[(2*S*)-2,5-Bis[(3*R*)-3-isocyanotetradecanamido]pentanamido]-3-phenylpropyl acetate (22f): In a 5-mL roundbottom flask, 2,5-dioxopyrrolidin-1-yl (*R*)-3-isocyanotetradecanoate (**15f**) (0.0375 g, 0.1070 mmol, 2.7 equiv) and DIPEA (0.040 mL, 0.2295 mmol, 6 equiv) were dissolved in anhyd CH_2Cl_2 (0.13 M, 0.300 mL, 3:1 CH_2Cl_2/MeOH) and cooled to 0 °C for 10 min. The crude bis(trifluoroacetate) salt **21** (0.0210 g, 0.0392 mmol, 1 equiv) was dissolved in anhyd MeOH (0.39 M , 0.100 mL, 3:1 CH_2Cl_2/MeOH) and added to the stirring solution. The mixture was stirred at 0 °C for 5 min, warmed to 22 °C, and stirred for 4.5 h. The mixture was diluted with H_2O and extracted with CH_2Cl_2 (1 x 30 mL). The combined organic extracts were washed with brine, dried (Na_2SO_4), filtered, and concentrated by rotary evaporation to afford the crude product. Purification by silica flash chromatography (70% EtOAc/hexanes) yielded diisonitrile **22f** as a white powder (0.0094 g, 31%).

TLC: R_f 0.18 (70% EtOAc/hexanes, UV, $KMnO_4$). **^{1}H NMR** (600 MHz, $CDCl_3$): δ 7.28 (dd, *J* = 8.1, 6.9 Hz, 2H), 7.24 – 7.20 (m, 1H), 7.19 (tt, *J* = 5.9, 1.3 Hz, 2H), 6.76 (d, *J* = 8.2 Hz, 1H), 4.52

(td, J = 8.6, 3.4 Hz, 1H), 4.38 (dtd, J = 12.6, 7.5, 5.3 Hz, 1H), 4.07 (d, J = 5.2 Hz, 2H), 4.03 (ddt, J = 13.6, 9.1, 4.8 Hz, 2H), 3.64 (dq, J = 13.8, 5.0 Hz, 1H), 3.11 – 3.03 (m, 1H), 2.87 (dd, J = 13.8, 6.9 Hz, 1H), 2.80 (dd, J = 13.8, 7.5 Hz, 1H), 2.46 (dd, J = 14.2, 9.9 Hz, 1H), 2.43 – 2.37 (m, 2H), 2.08 (s, 3H), 1.87 (ddd, J = 16.5, 8.4, 5.3 Hz, 1H), 1.65 (tdd, J = 14.9, 7.7, 4.6 Hz, 7H), 1.52 (tq, J = 10.5, 5.1 Hz, 3H), 1.46 – 1.38 (m, 2H), 1.28 (d, J = 21.6 Hz, 34H), 0.88 (t, J = 7.0 Hz, 6H). **^{13}C NMR** (151 MHz, $CDCl_3$): δ 170.19, 169.93, 169.85, 168.10, 168.01, 167.74, 154.59, 154.10, 135.89, 128.22, 127.59, 125.73, 63.67, 63.65, 51.85, 51.61, 51.32, 51.28, 49.03, 48.93, 41.75, 41.71, 41.57, 37.42, 37.29, 36.38, 36.35, 33.75, 33.69, 30.89, 29.36, 28.68, 28.64, 28.60, 28.59, 28.51, 28.49, 28.40, 28.38, 28.32, 27.87, 27.83, 24.70, 24.68, 24.66, 24.64, 21.67, 19.94, 13.10. **HRMS** (ESI) *m/z* calcd for $C_{46}H_{75}N_5O_5$ ($[M+H]^+$) 800.5666; found 800.5692.

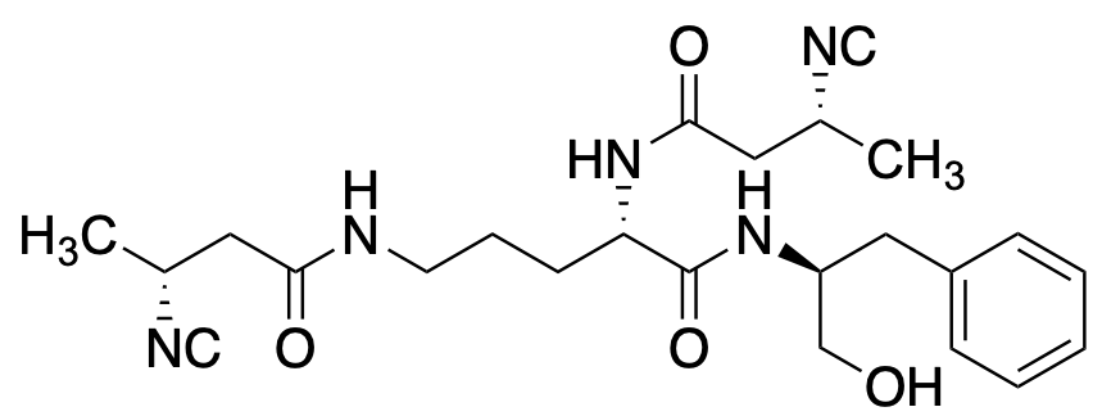

(3*R*,3'*R*)-*N*,*N*'-((*S*)-5-(((*S*)-1-Hydroxy-3-phenylpropan-2-yl)amino)-5-oxopentane-1,4-diyl)bis(3-isocyanobutanamide) (23a, C4-Orn-Phe-OH): In a 5-mL roundbottom flask, N^2,N^5-Bis((*R*)-3-isocyanobutanoyl)-L-ornithyl-L-phenylalaninol acetate (**7**) (0.101 g, 0.202 mmol, 1 equiv) was dissolved in a 10:1 mixture of anhyd MeOH (0.1 M, 2.5 mL) and H_2O (1 M, 0.250 mL). Potassium carbonate (0.339 g, 2.45 mmol, 12 equiv) was added to the solution. The reaction mixture was stirred at 22 °C for 6h. The mixture was filtered through a pad of celite and washed with MeOH (25 mL), then concentrated by rotary evaporation to afford the crude product. Purification by silica flash chromatography (5% MeOH/EtOAc) yielded diisonitrile **23a** as a white powder (0.054 g, 60%).

TLC: R_f 0.48 (2.5% MeOH/EtOAc, UV, $KMnO_4$). **^{1}H NMR** (600 MHz, MeOD): δ 7.28 – 7.21 (m, 4H), 7.21 – 7.14 (m, 1H), 4.37 (dd, J = 8.7, 5.4 Hz, 1H), 4.20 – 4.11 (m, 2H), 4.09 (dq, J = 7.6, 5.6 Hz, 1H), 3.55 – 3.47 (m, 2H), 3.30 – 3.24 (m, 1H), 3.14 (dt, J = 13.4, 6.6 Hz, 1H), 2.91 (dd, J = 13.8, 6.3 Hz, 1H), 2.76 (dd, J = 13.8, 8.0 Hz, 1H), 2.62 (dd, J = 14.6, 8.8 Hz, 1H), 2.54

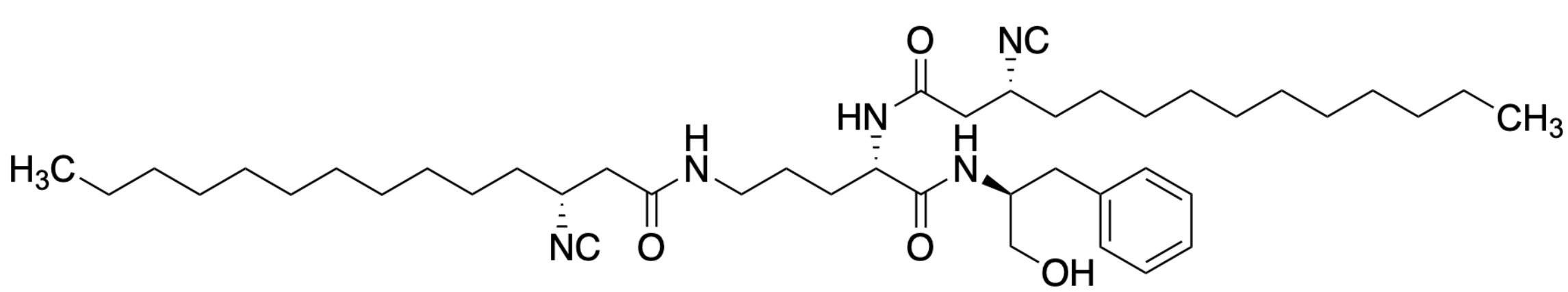

(dd, J = 14.5, 8.5 Hz, 1H), 2.48 (dtd, J = 14.4, 7.0, 3.9 Hz, 2H), 1.76 (ddt, J = 14.6, 9.5, 6.4 Hz, 1H), 1.67 – 1.49 (m, 3H), 1.40 (ddt, J = 6.7, 4.3, 2.0 Hz, 6H). **^{13}C NMR** (151 MHz, MeOD): δ 172.22, 169.93, 169.78, 154.20, 154.16, 154.13, 154.08, 154.04, 154.01, 138.28, 128.99, 127.95, 125.91, 62.51, 52.90, 52.82, 42.73, 42.49, 38.34, 36.48, 29.20, 25.39, 20.35, 20.32, -1.45. **HRMS** (ESI) *m/z* calcd for $C_{24}H_{34}N_5O_4$ ($[M+H]^+$) 456.2611; found 456.2610.

(3*R*,3'*R*)-*N*,*N*'-((*S*)-5-(((*S*)-1-Hydroxy-3-phenylpropan-2-yl)amino)-5-oxopentane-1,4-diyl)bis(3-isocyanotetradecanamide) (23f, C14-Orn-Phe-OH): In a 5-mL roundbottom flask, (2*S*)-2-[(2*S*)-2,5-Bis[(3*R*)-3-isocyanotetradecanamido]pentanamido]-3-phenylpropyl acetate (**22f**) (0.009 g, 0.0116 mmol, 1 equiv) was dissolved in a 10:1 mixture of anhyd MeOH (0.01 M, 1 mL)

and H_2O (0.1 M, 0.1 mL). Potassium carbonate (0.0192 g, 0.1386 mmol, 12 equiv) was added to the solution. The reaction mixture was stirred at 22 °C for 4 h. The mixture was filtered through a pad of celite and washed with MeOH (10 mL), then concentrated by rotary evaporation to afford the crude product. Purification by silica flash chromatography (5% MeOH/EtOAc) yielded diisonitrile **23f** as a white powder (0.0065 g, 76%).

TLC: R_f 0.51 (100% EtOAc, UV, $KMnO_4$). **^{1}H NMR** (600 MHz, DMSO-d_6): δ 8.13 (d, *J* = 8.3 Hz, 1H), 8.02 (t, *J* = 5.8 Hz, 1H), 7.73 (d, *J* = 8.3 Hz, 1H), 7.30 – 7.12 (m, 5H), 4.76 (t, *J* = 5.4 Hz, 1H), 4.27 (q, *J* = 7.7 Hz, 1H), 3.96 (s, 1H), 3.87 (d, *J* = 6.0 Hz, 1H), 3.27 (dt, *J* = 11.0, 5.9 Hz, 1H), 3.04 (d, *J* = 6.5 Hz, 2H), 2.85 (dd, *J* = 13.7, 5.7 Hz, 1H), 2.67 – 2.58 (m, 1H), 2.48 – 2.45 (m, 1H), 2.42 (d, *J* = 14.5 Hz, 2H), 2.01 (dt, *J* = 11.7, 6.7 Hz, 1H), 1.62 – 1.49 (m, 3H), 1.44 (d, *J* = 33.0 Hz, 4H), 1.35 (s, 3H), 1.25 (s, 36H), 0.86 (t, 6H). **^{13}C NMR** (151 MHz, DMSO-d_6): δ 170.32, 167.45, 138.35, 129.03, 128.50, 127.43, 125.25, 61.53, 51.70, 33.05, 30.68, 28.40, 28.32, 28.30, 28.22, 28.10, 27.67, 27.64, 24.89, 24.41, 21.49, 13.35. **HRMS** (ESI) *m/z* calcd for $C_{44}H_{74}N_5O_4$ ($[M+H]^+$) 736.5741; found 736.5736.

E. SYNTHESIS OF C4-ORN-OAC (24)

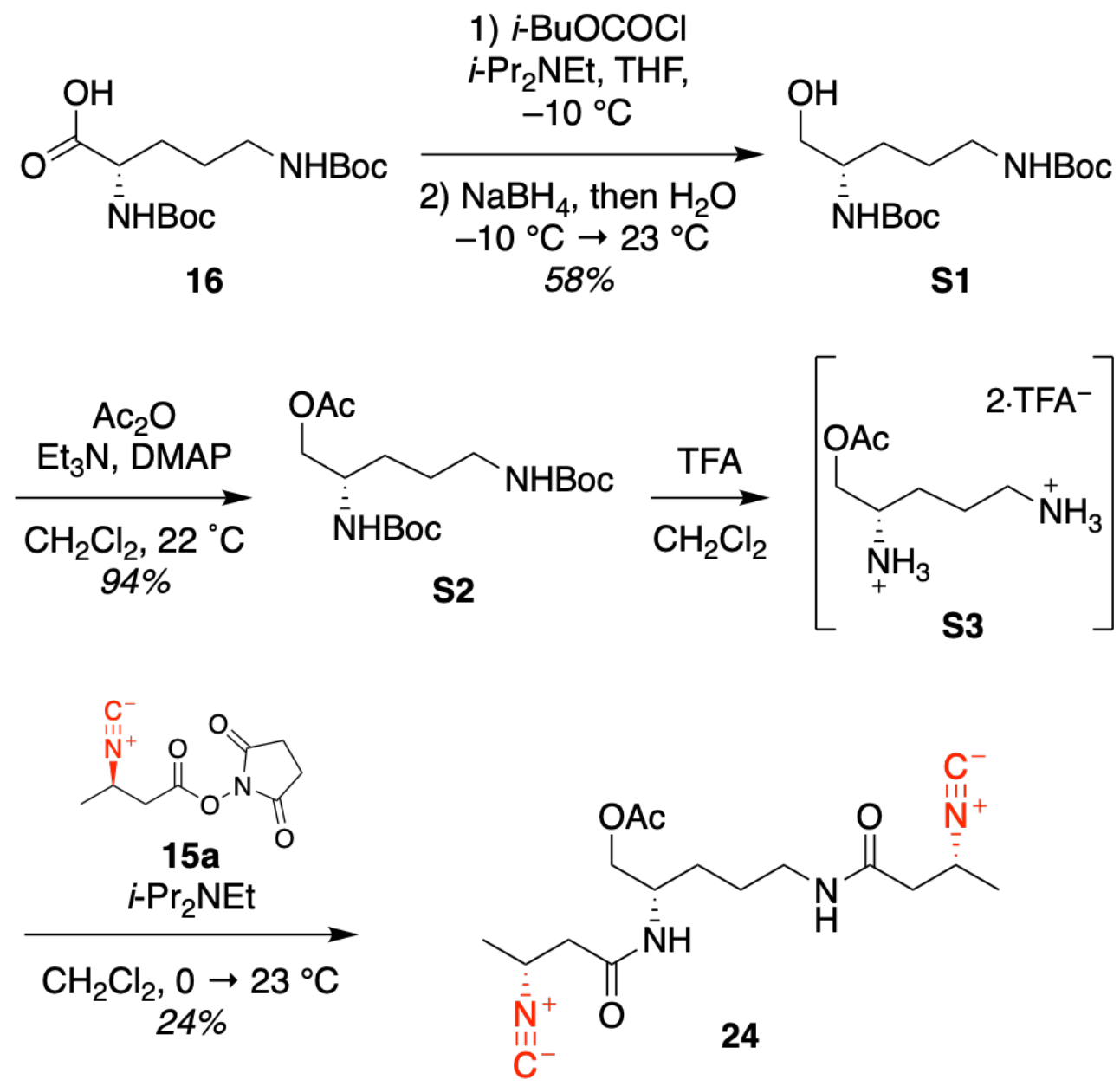

Supplementary Figure S2. Synthesis of C4-Orn-OAc (24). Boc = *t*-butoxycarbonyl; DMAP = 4-(dimethylamino)pyridine; TFA = trifluoroacetic acid; THF = tetrahydrofuran.

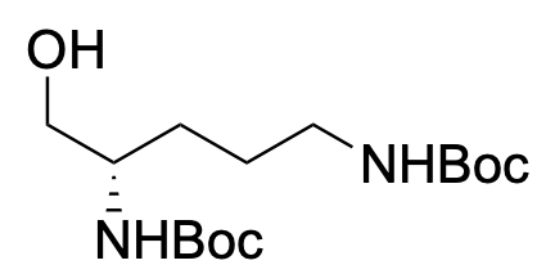

Di-*tert*-butyl (5-hydroxypentane-1,4-diyl)(*S*)-dicarbamate (S1, Boc-Orn[Boc]-OH). In a 250-mL roundbottom flask, (*S*)-2,5-bis((*tert*-butoxycarbonyl)amino)pentanoic acid (**16**) (10.563 g, 31.76 mmol, 1 equiv) and DIPEA (6.9 mL, 39.6 mmol, 1.25 equiv), were dissolved in anhyd THF (100 mL, 0.3 M) and cooled to –10 °C. Isobutyl chloroformate (5.2 mL, 40.08 mmol, 1.26 equiv) was added to the stirring solution. The mixture was stirred at –10 °C for 2.5 h. The mixture was warmed to 0 °C then filtered through a pad of celite and washed with THF (100 mL). The filtrate was cooled to –10 °C for 5 min and $NaBH_4$ (2.1702 g, 57.41 mmol, 1.8 equiv) was added to the stirring solution. H_2O (3 mL, 10.59 mmol, 0.25 equiv) was added dropwise and the mixture was cooled at –10 °C for 10 min, warmed to 23 °C, and stirred for 18.5 h. The reaction was diluted with H_2O and extracted with EtOAc (3 x 75 mL). The combined organic extracts were washed with 1 N HCl and satd aq $NaHCO_3$, dried (Na_2SO_4), filtered, and concentrated by rotary evaporation to afford the crude product. Purification by silica flash chromatography (50–100% EtOAc/hexanes) yielded alcohol **S1** (5.8487 g, 18.36 mmol, 58%) as a white solid.

TLC: *Rf* 0.68 (100% EtOAc). **^{1}H-NMR** (600 MHz, $CDCl_3$) δ 4.73 (s, 1H), 4.63 (s, 1H), 3.72 – 3.58 (m, 2H), 3.56 (dt, *J* = 9.7, 4.2 Hz, 1H), 3.14 (dq, *J* = 13.2, 7.2 Hz, 2H), 2.47 (s, 1H), 1.55 (dqd, *J* = 15.7, 9.9, 4.7 Hz, 3H), 1.44 (s, 19H). **^{13}C-NMR** (151 MHz, $CDCl_3$) δ 156.41, 156.13, 79.65, 79.26, 65.65, 52.49, 40.30, 28.63, 28.42, 28.38, 26.79. **HRMS** (ESI) *m/z* calcd for $C_{15}H_{30}N_2O_5$ ($[M+Na]^+$) 341.2052; found 341.2051.

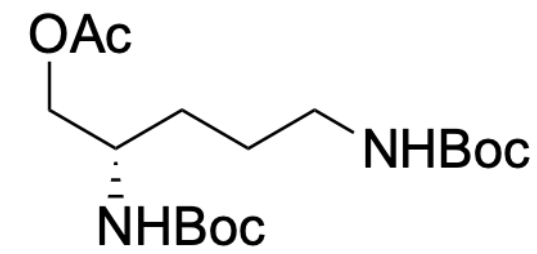

(*S*)-2,5-Bis((*tert*-butoxycarbonyl)amino)pentyl acetate (S2, Boc-Orn[Boc]-OAc). In a 25-mL roundbottom flask, Boc-Orn[Boc]-OH (**S1**) (1.1641 g, 3.655 mmol, 1.0 equiv), 4-(dimethylamino)pyridine (95 mg, 0.7776 mmol, 0.21 equiv), and triethylamine (1.02 mL, 7.317 mmol, 2.0 equiv) were dissolved in anhyd CH_2Cl_2 (10 mL, 0.3 M). Acetic anhydride (0.518 mL, 5.479 mmol, 1.5 equiv) was added to the solution and the mixture was stirred at 22 °C for 19 h. The reaction was quenched by addition of satd aq $NaHCO_3$ and extracted with CH_2Cl_2 (3 x 20 mL). The combined organic extracts were washed with brine, dried (Na_2SO_4), filtered, and concentrated by rotary evaporation to afford the crude product. Purification by silica flash chromatography (25–50% EtOAc/hexanes) yielded acetate **S2** (1.2313 g, 3.42 mmol, 94%) as a white solid.

TLC: R_f 0.64 (50% EtOAc/hexanes, $KMnO_4$). **^{1}H NMR** (500 MHz, $CDCl_3$): δ 4.58 (d, *J* = 9.8 Hz, 2H), 4.04 (qd, *J* = 11.3, 4.8 Hz, 2H), 3.88 – 3.78 (m, 1H), 3.13 (q, *J* = 6.5 Hz, 2H), 2.06 (s, 3H), 1.62 – 1.48 (m, 3H), 1.43 (d, *J* = 1.9 Hz, 20H). **^{13}C NMR** (126 MHz, $CDCl_3$): δ 171.09, 156.12, 155.61, 79.69, 66.39, 49.53, 40.37, 29.31, 28.54, 28.49, 26.79, 20.97. **HRMS** (ESI) *m/z* calcd for $C_{17}H_{32}N_2O_6Na$ ($[M+H+Na]^+$) 383.2158; found 383.2153.

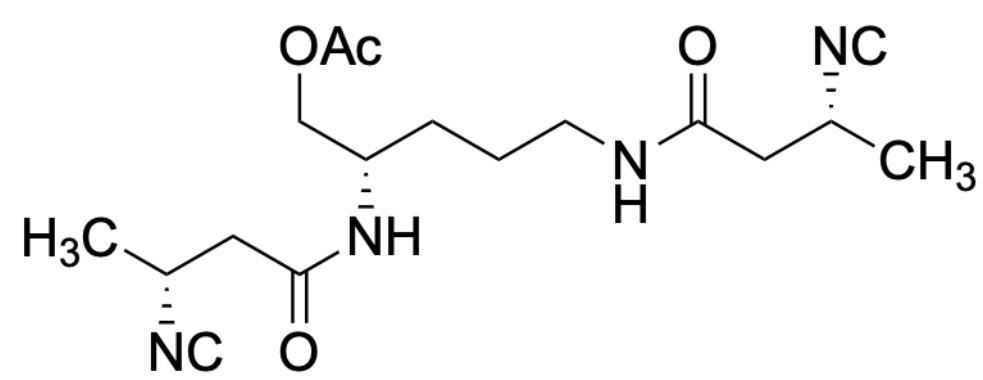

(*S*)-2,5-Bis((*R*)-3-isocyanobutanamido)pentyl acetate (24, C4-Orn-OAc). In a 50-mL roundbottom flask, acetate **S2** (3.579 g, 9.929 mmol, 1.0 equiv) was dissolved in anhyd CH_2Cl_2 (12 mL, 0.8 M) and cooled to 0 °C. Trifluoroacetic acid (12 mL, 153.6 mmol, 15 equiv) was added and the mixture was stirred at 0 °C for 5 min, warmed to 22 °C, and stirred for 20 h. The mixture was concentrated by rotary evaporation to afford the crude bis(trifluoroacetate) salt **S3**, which was used without further purification.

In a 20-mL roundbottom flask, the crude bis(trifluoroacetate) salt **S3** (290 mg, 0.7469 mmol, 1.0 equiv) and DIPEA (784 µL, 4.5 mmol, 6 equiv) were dissolved in anhyd THF (10 mL, 0.3 M) and cooled to 0 °C. 2,5-Dioxopyrrolidin-1-yl (*R*)-3-isocyanobutanoate (**15a**), prepared as previously described,[4] (401 mg, 1.91 mmol, 2.5 equiv) was added to the solution and the mixture was stirred at 0 °C for 5 min, warmed to 22 °C, and stirred for 24 h. The mixture was diluted with H_2O and extracted with CH_2Cl_2 (3 x 20 mL). The combined organic extracts were washed with brine, dried (Na_2SO_4), filtered, and concentrated by rotary evaporation to afford the crude product. Purification by silica flash chromatography (100% EtOAc) yielded diisonitrile **24** (62 mg, 0.18 mmol, 24%) as a clear yellow solid.

TLC: R_f 0.19 (100% EtOAc). **^{1}H NMR** (600 MHz, MeOD): δ 4.19 – 4.12 (m, 4H), 4.00 – 3.95 (m, 1H), 3.28 (dt, *J* = 13.0, 6.5 Hz, 1H), 3.20 (dt, *J* = 13.1, 6.5 Hz, 1H), 2.56 (dddt, *J* = 21.5, 14.4, 8.6, 1.3 Hz, 2H), 2.51 (dd, *J* = 5.7, 2.9 Hz, 0H), 2.48 (ddt, *J* = 8.4, 5.9, 2.3 Hz, 1H), 2.04 (s, 3H), 1.65 (tdd, *J* = 9.6, 4.8, 2.3 Hz, 2H), 1.60 – 1.56 (m, 1H), 1.53 – 1.47 (m, 1H), 1.42 (tt, *J* = 6.4, 2.2 Hz, 6H). **^{13}C NMR** (151 MHz, MeOD): δ 171.23, 169.88, 154.28, 65.60, 53.40, 48.16, 48.06,

42.80, 42.75, 38.60, 27.93, 25.50, 20.33, 20.29, 19.32. **HRMS** (ESI) *m/z* calcd for $C_{17}H_{27}N_4O_4$ ($[M+H]^+$) 351.2032; found 351.2031.

F. Synthesis of C4-Lys-Phe-OAc (25)

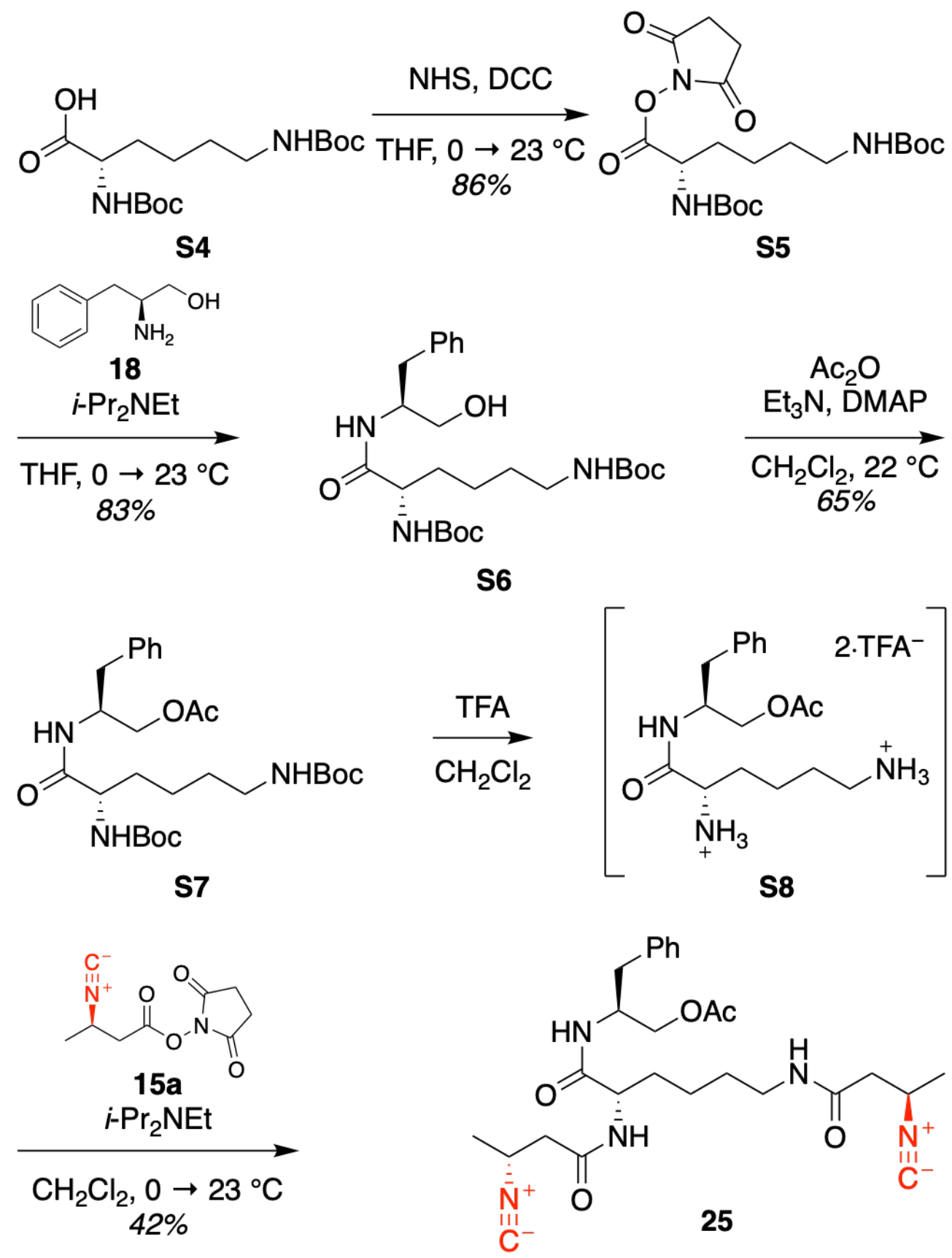

Supplementary Figure S3. Synthesis of C4-Lys-Phe-OAc (25). Boc = *t*-butyloxycarbonyl; DCC = *N,N'*-dicyclohexylcarbodiimide; DMAP = 4-(dimethylamino)pyridine; NHS = N-hydroxysuccinimide; TFA = trifluoroacetic acid; THF = tetrahydrofuran.

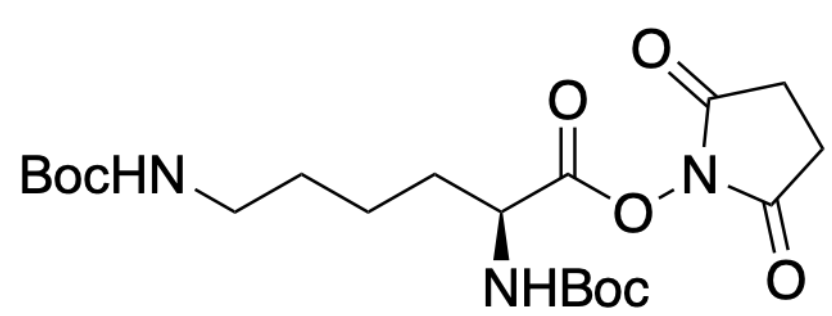

***N*^{2},*N*^{6}-Bis(*tert*-butoxycarbonyl)-L-lysine *N*-hydroxysuccinimide ester (S5, Boc-Lys[Boc]-NHS).** In a 250-mL roundbottom flask, *N*^{2},*N*^{6}-bis(*tert*-butoxycarbonyl)-L-lysine (**S4**) (10.03 g, 28.95 mmol, 1.0 equiv) and dicyclohexylcarbodiimide (6.08 g, 29.46 mmol, 1.02 equiv) were suspended in 100 mL anhyd THF and cooled to 0 °C for 10 min. *N*-Hydroxysuccinimide (3.49 g, 30.32 mmol, 1.05 equiv) was added to the stirring solution in one portion. The mixture was stirred at 0 °C for 30 min, warmed to 22 °C, and stirred for 22.33 h. The mixture was filtered through a pad of celite and the solid white residue was washed with Et_2O (40 mL). The combined filtrates were concentrated by rotary evaporation to afford the crude product as an off-white solid. Purification by silica flash chromatography (50% EtOAc in hexanes) yielded NHS ester **S5** (11.0986 g, 25.03 mmol, 86%) as a white solid.

TLC: R_f 0.56 (50% EtOAc in hexanes). **^{1}H-NMR** (500 MHz, $CDCl_3$) δ 5.18 (d, *J* = 8.0 Hz, 1H), 4.75 (d, *J* = 40.7 Hz, 1H), 4.65 (s, 1H), 3.12 (dd, *J* = 12.0, 6.3 Hz, 2H), 2.83 (s, 3H), 2.01 – 1.90 (m, 1H), 1.90 – 1.79 (m, 1H), 1.76 – 1.66 (m, 1H), 1.44 (s, 23H). **^{13}C-NMR** (126 MHz, $CDCl_3$)

δ 168.63, 168.47, 156.21, 79.19, 60.42, 51.80, 39.78, 32.04, 29.40, 28.44, 28.36, 28.29, 25.59, 24.86, 21.92, 14.20. **HRMS** (ESI) *m/z* calcd for $C_{20}H_{33}N_3O_8Na$ ($[M+Na]^+$) 466.2165; found 466.2169.

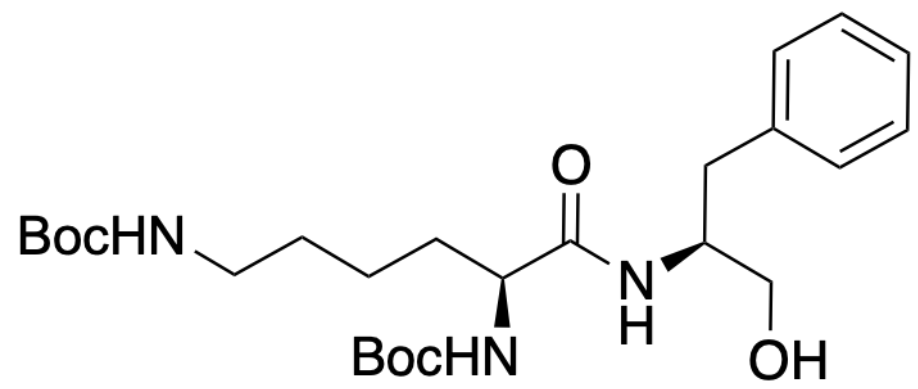

***N*^{2},*N*^{6}-Bis(*tert*-butoxycarbonyl)-L-lysyl-L-phenylalaninol (S6, Boc-Lys[Boc]-Phe-OH).** In a 100-mL roundbottom flask, L-phenylalaninol (2.5248 g, 16.69 mmol, 1.0 equiv) and DIPEA (8.6 mL, 49.37 mmol, 3.0 equiv) were dissolved in 30 mL anhyd THF and cooled to 0 °C for 10 min. *N*^{2},*N*^{6}-Bis(*tert*-butoxycarbonyl)-L-lysine *N*-hydroxysuccinimide ester (**S5**) (8.7483 g, 19.72 mmol, 1.18 equiv) was added to the stirring solution in one portion. The mixture was stirred at 0 °C for 5 min, warmed to 22 °C, and stirred for 26 h. The mixture was diluted with H_2O and extracted with CH_2Cl_2 (3 x 50 mL). The combined organic extracts were washed with brine, dried (Na_2SO_4), filtered, and concentrated by rotary evaporation to afford the crude product. Purification by silica flash chromatography (100% EtOAc) yielded protected dipeptide alcohol **S6** (6.6355 g, 13.8 mmol, 83%) as a white solid.

TLC: R_f 0.76 (100% EtOAc). **^{1}H-NMR** (600 MHz, $CDCl_3$): δ 7.32 (t, *J* = 7.5 Hz, 2H), 7.24 (d, *J* = 4.6 Hz, 2H), 6.53 (s, 1H), 5.40 (s, 1H), 4.79 (s, 1H), 4.22 (qt, *J* = 7.9, 4.2 Hz, 1H), 4.04 (s, 1H), 3.71 (d, *J* = 11.1 Hz, 1H), 3.57 (dd, *J* = 11.7, 5.0 Hz, 1H), 3.39 – 3.26 (m, 1H), 3.11 (qt, *J* = 13.8, 6.7 Hz, 2H), 2.90 (q, *J* = 6.8 Hz, 2H), 1.79 (h, *J* = 6.2 Hz, 1H), 1.70 – 1.59 (m, 1H), 1.47 (s, 21H), 1.32 (tq, *J* = 14.1, 8.5 Hz, 2H). **^{13}C-NMR** (151 MHz, $CDCl_3$) δ 172.23, 156.47, 156.00, 137.80, 129.26, 128.52, 126.52, 80.17, 79.32, 63.34, 54.91, 52.67, 39.65, 36.97, 33.93, 31.66, 29.63, 28.47, 28.33, 24.95, 22.29. **HRMS** (ESI) *m/z* calcd for $C_{25}H_{41}N_3O_6Na$ ($[M+Na]^+$) 502.2893; found 502.2899.

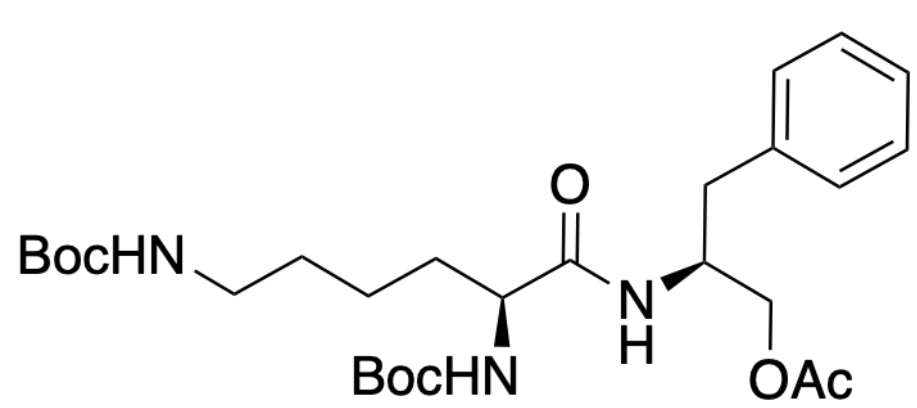

***N*^{2},*N*^{6}-Bis(tert-butoxycarbonyl)-L-lysyl-L-phenylalaninol acetate (S7, Boc-Lys[Boc]-Phe-OAc).** In a 250-mL roundbottom flask, *N*^{2},*N*^{6}-bis(*tert*-butoxycarbonyl)-L-lysyl-L-phenylalaninol (**S6**) (6.6355 g, 13.8 mmol, 1.0 equiv), 4-(dimethylamino)pyridine (481 mg, 3.937 mmol, 0.28 equiv), and triethylamine (3.85 mL, 27.66 mmol, 2.0 equiv) were dissolved in 100 mL anhyd CH_2Cl_2. Acetic anhydride (1.96 mL, 20.74 mmol, 1.5 equiv) was added to the solution and the mixture was stirred at 22 °C for 18.75 h. The reaction was quenched by addition of satd aq $NaHCO_3$ and extracted with CH_2Cl_2 (3 x 70 mL). The combined organic extracts were washed with brine, dried (Na_2SO_4), filtered, and concentrated by rotary evaporation to afford the crude product. Purification by silica flash chromatography (50% EtOAc/hexanes) yielded protected dipeptide acetate **S7** (4.6729 g, 9.0 mmol, 65%) as a white solid.

TLC: R_f 0.50 (50% EtOAc in hexanes). **^{1}H-NMR** (500 MHz, $CDCl_3$) δ 7.29 (t, *J* = 7.4 Hz, 2H), 7.22 (t, *J* = 7.2 Hz, 1H), 7.20 – 7.16 (m, 2H), 6.39 – 6.29 (m, 1H), 5.04 (s, 1H), 4.60 (s, 1H), 4.40 (s, 1H), 4.05 (d, *J* = 4.7 Hz, 2H), 3.98 (s, 1H), 3.07 (dt, *J* = 13.6, 6.8 Hz, 2H), 2.83 (qd, *J* = 13.7,

7.2 Hz, 2H), 2.08 (s, 3H), 1.75 (s, 1H), 1.56 (s, 1H), 1.44 (s, 21H), 1.36 – 1.21 (m, 3H). **^{13}C-NMR** (126 MHz, $CDCl_3$) δ 171.70, 170.96, 156.18, 136.96, 129.21, 128.64, 126.79, 77.24, 64.65, 49.51, 39.78, 37.54, 31.66, 29.65, 28.46, 28.32, 22.43, 21.06, 20.82, 14.21. **HRMS** (ESI) *m/z* calcd for $C_{27}H_{44}N_3O_7$ ([M+H]$^+$) 522.3179; found 522.3183.

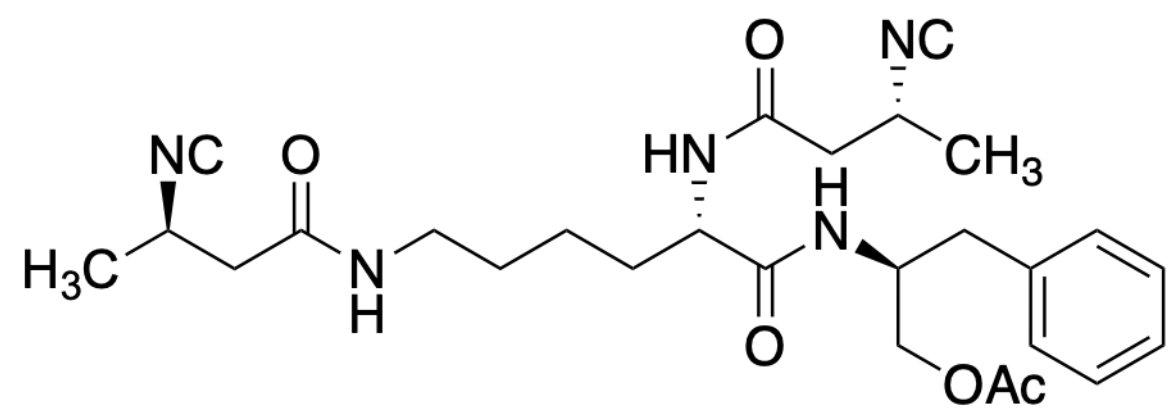

***N*^{2},*N*^{6}-Bis((*R*)-3-isocyanobutanoyl)-L-lysyl-L-phenylalaninol acetate (25, C4-Lys-Phe-OAc).** In a 100-mL roundbottom flask, *N*^{2},*N*^{6}-bis(*tert*-butoxycarbonyl)-L-lysyl-L-phenylalaninol acetate (**S7**) (4.6729 g, 14.44 mmol, 1.0 equiv) was dissolved in 18 mL anhyd CH_2Cl_2 and cooled to 0 °C. Trifluoroacetic acid (16.9 mL, 216.6 mmol, 15 equiv) was added and the mixture was stirred at 0 °C for 5 min, warmed to 22 °C, and stirred for 5 h. The mixture was concentrated by rotary evaporation to afford the crude bis(trifluoroacetate) salt **S8**, which was used without further purification.

In a 20-mL roundbottom flask, the crude bis(trifluoroacetate) salt **S8** (434 mg, 0.789 mmol, 1.0 equiv) and DIPEA (784 µL, 4.5 mmol, 5.7 equiv) were dissolved in 10 mL anhyd CH_2Cl_2 and cooled to 0 °C. 2,5-Dioxopyrrolidin-1-yl (*R*)-3-isocyanobutanoate (**15a**), prepared as previously described,[4] (399 mg, 1.89 mmol, 2.40 equiv) was added to the solution and the mixture was stirred at 0 °C for 5 min, warmed to 22 °C, and stirred for 18.25 h. The mixture was diluted with H_2O and extracted with CH_2Cl_2 (3 x 20 mL). The combined organic extracts were washed with brine, dried (Na_2SO_4), filtered, and concentrated by rotary evaporation to afford the crude product. Purification by silica flash chromatography (100% EtOAc) yielded diisonitrile **25** (170 mg, 0.33 mmol, 42%) as a colorless opaque solid.

TLC: R_f 0.18 (100% EtOAc). **^{1}H NMR** (600 MHz, MeOD): δ 8.01 – 7.94 (m, 1H), 7.25 (ddd, *J* = 8.0, 6.9, 1.2 Hz, 2H), 7.21 (ddd, *J* = 8.1, 3.4, 1.5 Hz, 2H), 7.19 – 7.15 (m, 1H), 4.36 – 4.29 (m, 2H), 4.27 (dd, *J* = 8.9, 5.4 Hz, 1H), 4.16 – 4.08 (m, 2H), 4.02 – 3.92 (m, 1H), 3.24 – 3.17 (m, 1H), 3.17 – 3.07 (m, 1H), 2.88 – 2.75 (m, 2H), 2.62 – 2.55 (m, 1H), 2.55 – 2.47 (m, 1H), 2.46 (td, *J* = 5.7, 3.4 Hz, 1H), 2.44 – 2.26 (m, 2H), 2.02 (s, 3H), 1.71 – 1.62 (m, 1H), 1.62 – 1.52 (m, 1H), 1.52 – 1.42 (m, 2H), 1.38 (dq, *J* = 6.3, 1.9 Hz, 3H), 1.35 (d, *J* = 6.6 Hz, 1H), 1.33 – 1.25 (m, 2H), 1.23 (dd, *J* = 6.7, 3.0 Hz, 1H), 1.20 – 1.11 (m, 2H). **^{13}C NMR** (151 MHz, MeOD): δ 174.14, 172.72, 172.53, 171.52, 171.24, 169.79, 169.76, 161.59, 161.59, 161.57, 161.55, 154.22, 137.60, 137.54, 137.53, 128.94, 128.12, 126.22, 126.20, 64.71, 64.67, 54.36, 53.39, 53.37, 53.36, 49.79, 48.50, 42.74, 42.52, 42.05, 42.04, 41.92, 41.85, 41.77, 38.72, 38.63, 38.61, 36.63, 36.61, 31.56, 31.55, 31.44, 28.59, 28.55, 24.91, 22.79, 22.76, 22.74, 20.42, 20.36, 19.46, 19.21, 19.10, 19.08, 17.43, 16.67. **HRMS** (ESI) *m/z* calcd for $C_{27}H_{42}N_5O_7$ ([M+2H_2O+H]$^+$) 548.3084; found 548.3079.

G. SYNTHESIS OF C4-CADAVERINE (26)

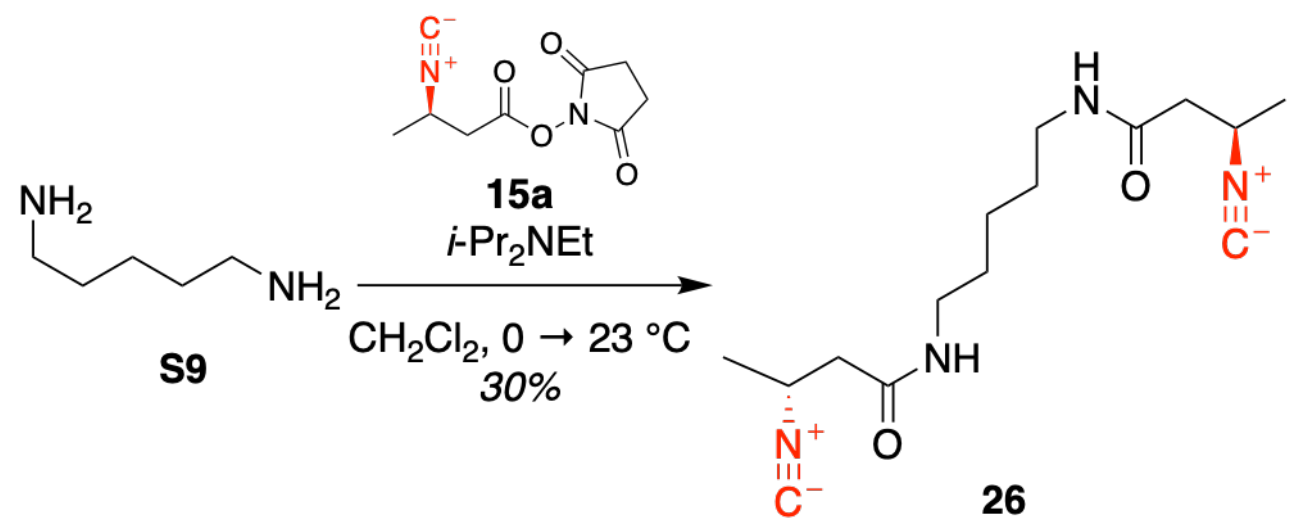

Supplementary Figure S4. Synthesis of C4-cadaverine analogue (26).

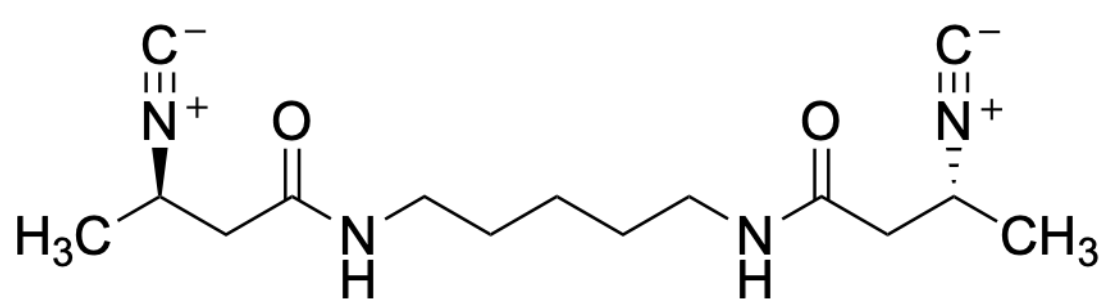

(*3R,3'R*)-*N,N'*-(Hexane-1,6-diyl)bis(3-isocyanobutanamide) (26, C4-cadaverine). In a 25-mL roundbottom flask, pentane-1,5-diamine (**S9**) (88 µL, 0.7518 mmol, 1.0 equiv) was dissolved in anhyd THF (10 mL, 0.08 M) and stirred at 0 °C. 2,5-Dioxopyrrolidin-1-yl (*R*)-3-isocyanobutanoate (**15a**), prepared as previously described,[4] (402 mg, 1.91 mmol, 2 equiv) was added to the solution and the mixture was cooled to 0 °C for 5 min, warmed to 22 °C, and stirred for 23.5 h. The mixture was diluted with H_2O and extracted with CH_2Cl_2 (3 x 10 mL). The combined organic extracts were washed with brine, dried (Na_2SO_4), filtered, and concentrated by rotary evaporation to afford the crude product. Purification by silica flash chromatography (100% EtOAc) yielded diisonitrile **26** (89 mg, 0.30 mmol, 30%).

TLC: R_f 0.30 (100% EtOAc). **^{1}H NMR** (600 MHz, MeOD): δ 8.16 (d, *J* = 6.2 Hz, 0H), 4.18 (dttd, *J* = 8.5, 6.8, 5.0, 1.6 Hz, 2H), 3.27 (dt, *J* = 12.1, 7.0 Hz, 2H), 3.20 (dt, *J* = 13.5, 6.9 Hz, 2H), 2.57 (ddt, *J* = 14.5, 8.6, 1.3 Hz, 2H), 2.49 (ddt, *J* = 14.5, 5.8, 3.0 Hz, 2H), 1.56 (h, *J* = 6.9 Hz, 4H), 1.52 – 1.36 (m, 9H). **^{13}C NMR** (151 MHz, MeOD): δ 169.84, 169.76, 154.26, 154.23, 154.19, 54.44, 42.80, 42.75, 39.02, 38.89, 28.62, 28.60, 24.91, 23.83, 20.36. **HRMS** (ESI) *m/z* calcd for $C_{15}H_{24}N_4O_2Na$ ($[M+Na]^+$) 315.1797; found 315.1808.

H. SYNTHESIS OF *S,S*-C4-ORN-PHE-OAC (27)

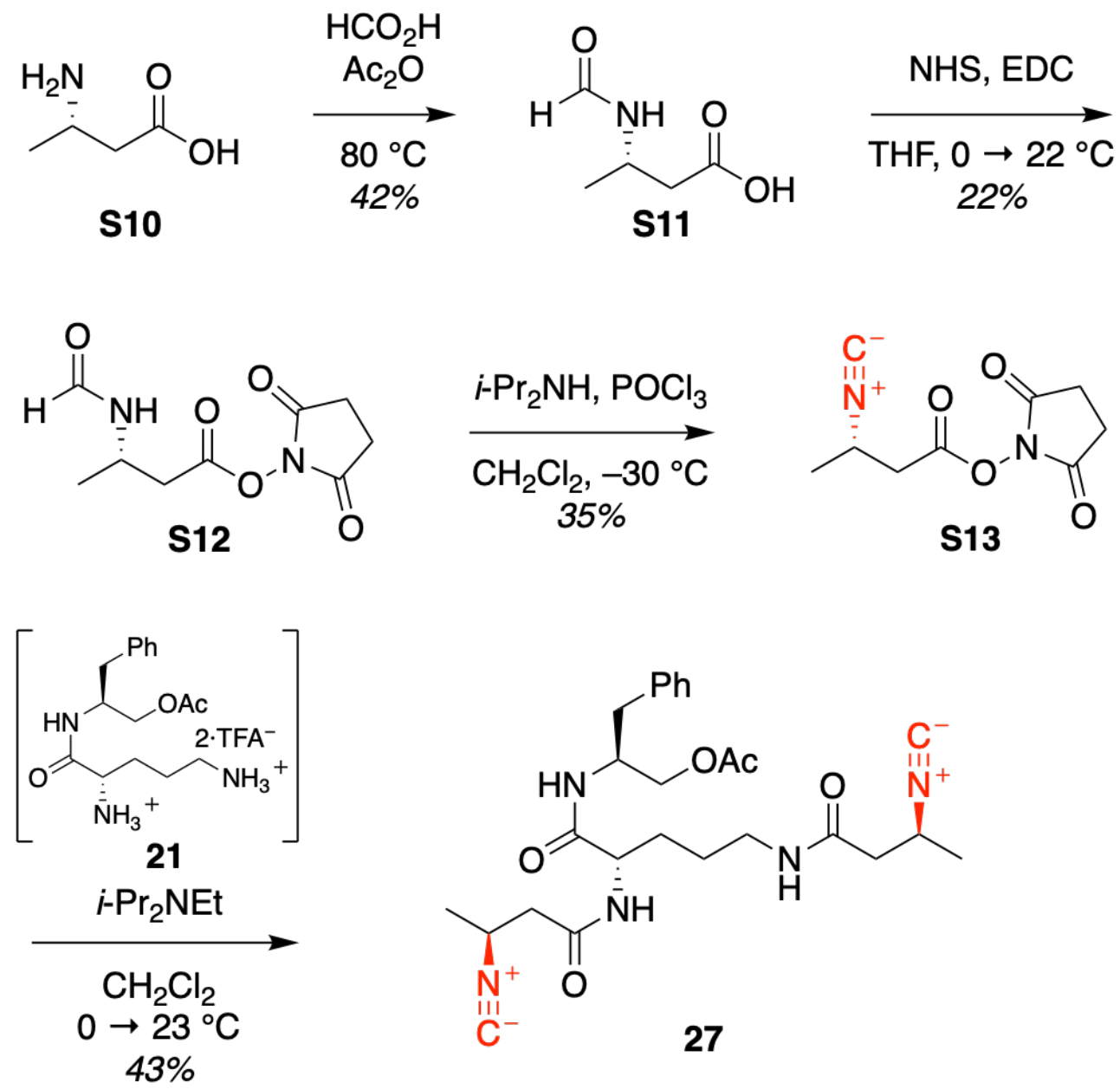

Supplementary Figure S5. Synthesis of (*S,S*)-C4-Orn-Phe-OAc (27). DCC = 1-ethyl-3-(dimethylamino-propyl)carbodiimide; NHS = N-hydroxysuccinimide; THF = tetrahydrofuran.

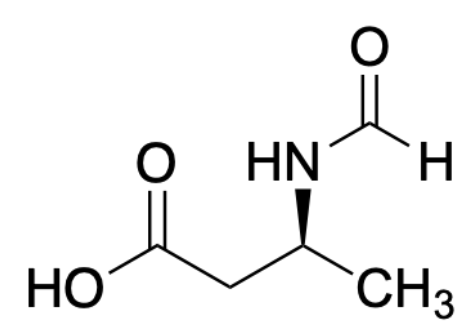

(*S*)-3-Formamidobutanoic acid (S11): In a 250-mL roundbottom flask containing (*S*)-3-aminobutanoic acid (**S10**) (2.4585 g, 23.83 mmol, 1 equiv), formic acid (15 mL, 397.6 mol, 17 equiv), and acetic anhydride (10 mL, 23.83 mmol, 4.4 equiv) were heated together at 80 °C for 19 h. The reaction mixture was concentrated by rotary evaporation to afford the crude product. Purification by recrystallization in ~6:1 hexanes:EtOAc yielded β-formamido acid **S11** as a white powder (1.3258 g, 10.11 mmol, 43%).

TLC: R_f 0.11 (75% EtOAc/hexanes, CAM, $KMnO_4$). **^{1}H NMR** (600 MHz, $CDCl_3$): δ 8.12 (s, 1H), 6.26 (s, 1H), 4.51 – 4.43 (m, 1H), 2.61 (dd, *J* = 12.3, 5.4 Hz, 2H), 1.36 (d, *J* = 6.8 Hz, 1H), 1.30 (d, *J* = 6.8 Hz, 3H). **^{13}C NMR** (151 MHz, $CDCl_3$): δ 175.38, 175.28, 174.20, 165.10, 160.89, 45.62, 42.12, 41.47, 40.81, 40.69, 39.92, 39.56, 23.37, 21.60, 20.08, 19.99, 14.14, 1.03. **HRMS** (ESI) *m/z* calcd for $C_5H_9NO_3Na$ ($[M+H+Na]^+$) 154.0480; found 154.0476.

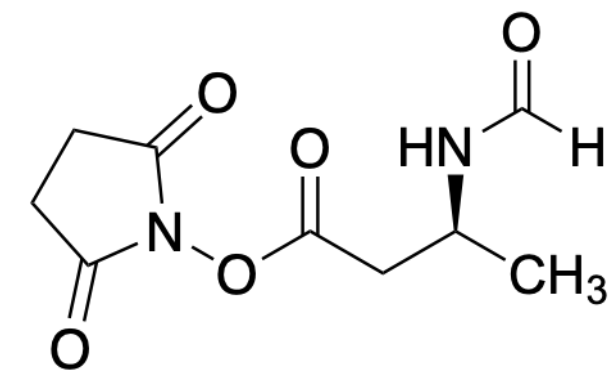

2,5-Dioxopyrrolidin-1-yl (*S*)-3-formamidobutanoate (S12): In a 50-mL roundbottom flask, (*S*)-3-formamidobutanoic acid (**S11**) (1.003 g, 7.648 mmol, 1 equiv) and dicyclohexylcarbodiimide (1.5995 g, 7.749 mmol, 1.013 equiv) were suspended in THF (16.6 mL, 0.46 M) and cooled to 0 °C for 10 min. *N*-hydroxysuccinimide (0.8937 g, 7.765 mmol, 1.015 equiv) was added to the stirring solution in one portion. The mixture was stirred at 0 °C for 30 min, warmed to 22 °C, and stirred for 16 h. The mixture was filtered through a pad of celite and washed with Et_2O (20 mL). The combined filtrates were concentrated by rotary evaporation to afford the crude product. Purification by silica flash chromatography (80% EtOAc/hexanes) yielded β-formamido NHS ester **S12** as a white powder (0.3894 g, 22%).

TLC: R_f 0.18 (80% EtOAc/hexanes, UV, $KMnO_4$). **^{1}H NMR** (500 MHz, $CDCl_3$): δ 8.13 (s, 1H), 6.10 (s, 1H), 4.61 – 4.52 (m, 1H), 2.94 (dd, *J* = 15.0, 5.7 Hz, 1H), 2.85 (s, 4H), 2.80 (dd, *J* = 15.0, 4.6 Hz, 1H), 1.35 (d, *J* = 6.9 Hz, 3H). **^{13}C NMR** (126 MHz, $CDCl_3$): δ 166.21, 160.62, 60.41, 45.10, 40.65, 39.36, 37.13, 25.61, 25.58, 21.74, 21.06, 19.40, 14.20. **HRMS** (ESI) *m/z* calcd for $C_9H_{12}N_2O_5Na$ ($[M+Na]^+$) 251.0644; found 251.0642.

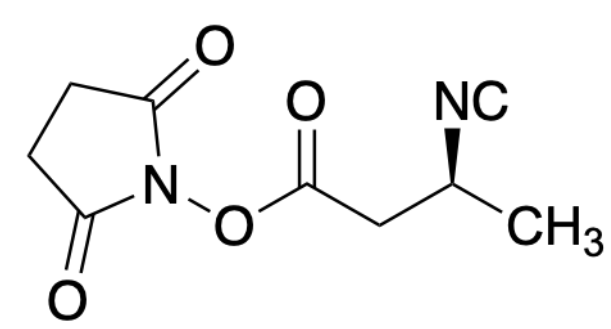

2,5-Dioxopyrrolidin-1-yl (*S*)-3-isocyanobutanoate (S13): In a 5-mL roundbottom flask, 2,5-dioxopyrrolidin-1-yl (*S*)-3-formamidobutanoate (**S12**) (0.1782 g, 0.7808 mmol, 1 equiv) and diisopropylamine (0.300 mL, 2.125 mmol, 2.7 equiv) were dissolved in CH_2Cl_2 (1.3 mL, 0.6 M total) and cooled to –30 °C for 15 min. $POCl_3$ (0.090 mL, 0.9653 mmol, 1.2 equiv) was added to the solution and the mixture was stirred at –30 °C for 2 h. The reaction mixture was quenched with satd aq $NaHCO_3$ and extracted with CH_2Cl_2 (2 x 40 mL). The combined organic extracts were washed with brine, dried (Na_2SO_4), filtered, and concentrated by rotary evaporation to afford the crude product. Purification by silica flash chromatography (50% EtOAc/hexanes) yielded β-isocyano NHS ester **S13** as a white powder (0.0567 g, 0.2697 mmol, 35%).

TLC: R_f 0.28 (50% EtOAc/hexanes, $KMnO_4$). **^{1}H NMR** (500 MHz, $CDCl_3$): δ 4.16 (h, *J* = 6.8 Hz, 1H), 3.10 (dd, *J* = 16.3, 6.4 Hz, 1H), 2.90 (dd, 1H), 2.86 (s, 4H), 1.55 (d, *J* = 2.1 Hz, 3H **^{13}C NMR** (126 MHz, $CDCl_3$): δ 168.57, 164.59, 45.87, 38.32, 25.58, 21.26. **HRMS** (ESI) *m/z* calcd for $C_9H_{10}N_2O_4Na$ ($[M+Na]^+$) 233.0538; found 233.0532.

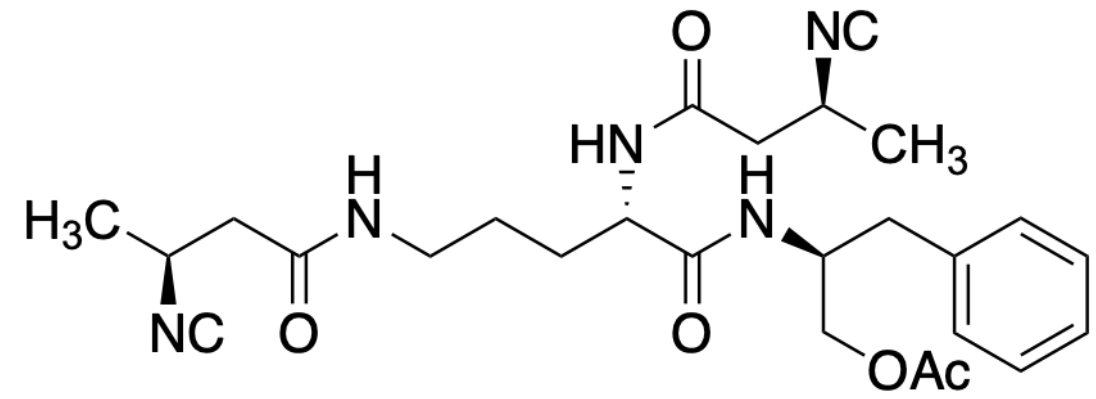

***N*[2],*N*[5]-Bis((*S*)-3-isocyanobutanoyl)-L-ornithyl-L-phenylalaninol acetate (27, (*S,S*)-C4-Orn-Phe-OAc).** In a 15-mL roundbottom flask, the crude bis(trifluoroacetate) salt **21** (55.6 mg, 0.1038 mmol, 1.0 equiv) and DIPEA (0.110 mL, 0.6314 mmol, 6 equiv) were dissolved in 0.9 mL anhyd CH_2Cl_2 and cooled to 0 °C. 2,5-Dioxopyrrolidin-1-yl (*S*)-3-isocyanobutanoate (**S13**) (56.7 mg, 0.2697 mmol, 2.6 equiv) was added to the stirring solution and the mixture was stirred at 0 °C for 5 min, warmed to 22 °C, and stirred for 2 h. The mixture was diluted with H_2O and extracted with CH_2Cl_2 (3x20 mL). The combined organic extracts were washed with brine, dried (Na_2SO_4), filtered, and concentrated by rotary evaporation to afford the crude product. Purification by silica flash chromatography (100% EtOAc) yielded diisonitrile **27** (22.4 mg, 0.04501 mmol, 43%) as a white solid.

TLC: R_f 0.20 (100% EtOAc). **^{1}H NMR** (500 MHz, $CDCl_3$): δ 7.29 (t, *J* = 7.4 Hz, 2H), 7.23 (d, *J* = 7.2 Hz, 1H), 7.21 – 7.15 (m, 2H), 7.01 (s, 1H), 6.86 (d, *J* = 8.6 Hz, 1H), 6.60 (d, *J* = 7.9 Hz, 1H), 4.54 (dq, *J* = 9.2, 4.9 Hz, 1H), 4.41 – 4.32 (m, 1H), 4.22 (q, *J* = 7.0 Hz, 1H), 4.16 (q, *J* = 6.8 Hz, 1H), 4.08 (dd, *J* = 11.4, 4.0 Hz, 1H), 3.97 (dd, *J* = 11.3, 5.7 Hz, 1H), 3.51 (q, *J* = 10.4 Hz, 1H), 3.24 – 3.15 (m, 1H), 2.82 (tt, *J* = 13.8, 7.2 Hz, 2H), 2.62 (dd, *J* = 14.9, 7.2 Hz, 1H), 2.52 – 2.41 (m, 3H), 2.08 (s, 3H), 1.80 – 1.67 (m, 2H), 1.66 – 1.60 (m, 1H), 1.51 (dt, *J* = 9.1, 4.6 Hz, 1H), 1.45 (dd, *J* = 6.8, 2.6 Hz, 6H). **^{13}C-NMR** (151 MHz, $CDCl_3$): δ 170.98, 170.86, 168.97, 168.91, 156.20, 154.05, 137.13, 129.25, 128.56, 126.69, 64.53, 52.98, 49.96, 48.42, 46.91, 46.87, 46.83, 44.47, 43.24, 39.81, 37.24, 31.41, 29.71, 24.09, 21.41, 21.09, 20.91, 1.02. **HRMS** (ESI) *m/z* calcd for $C_{26}H_{35}N_5O_5$ ($[M+H]^+$) 498.2716; found 498.2723.

I. SYNTHESIS OF C4-BISFORMAMIDE (28)

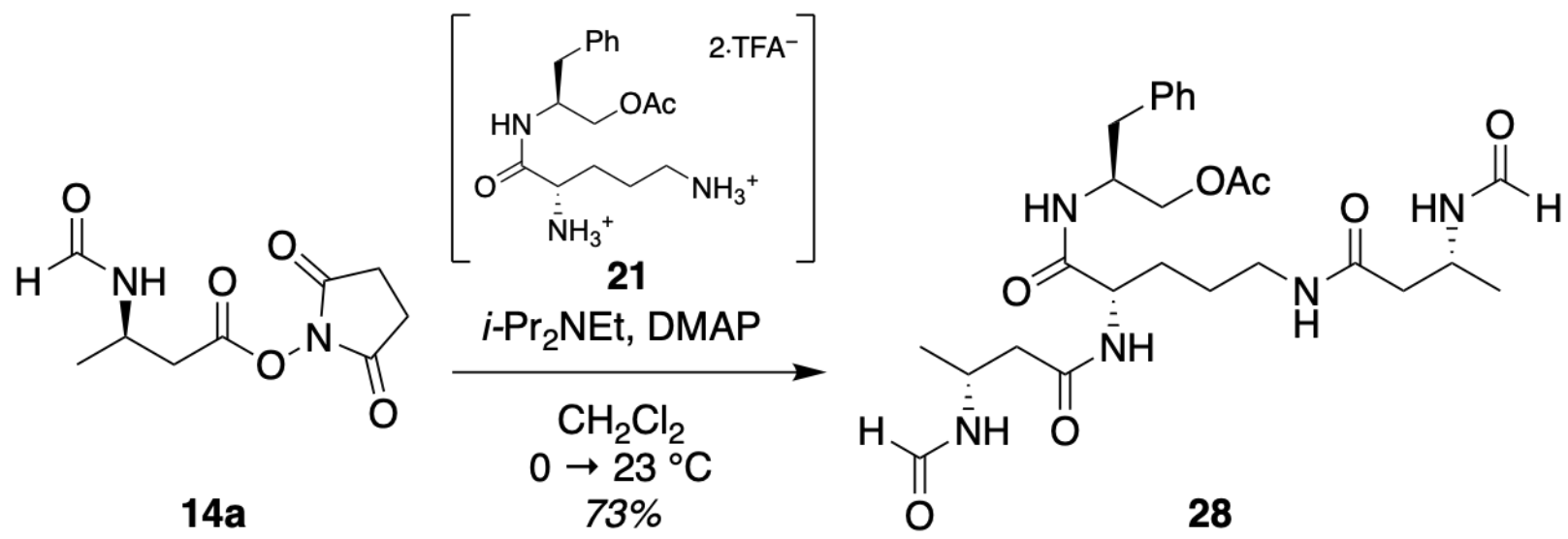

Supplementary Figure S6. Synthesis of (*R,R*)-C4-bisformamide (28).

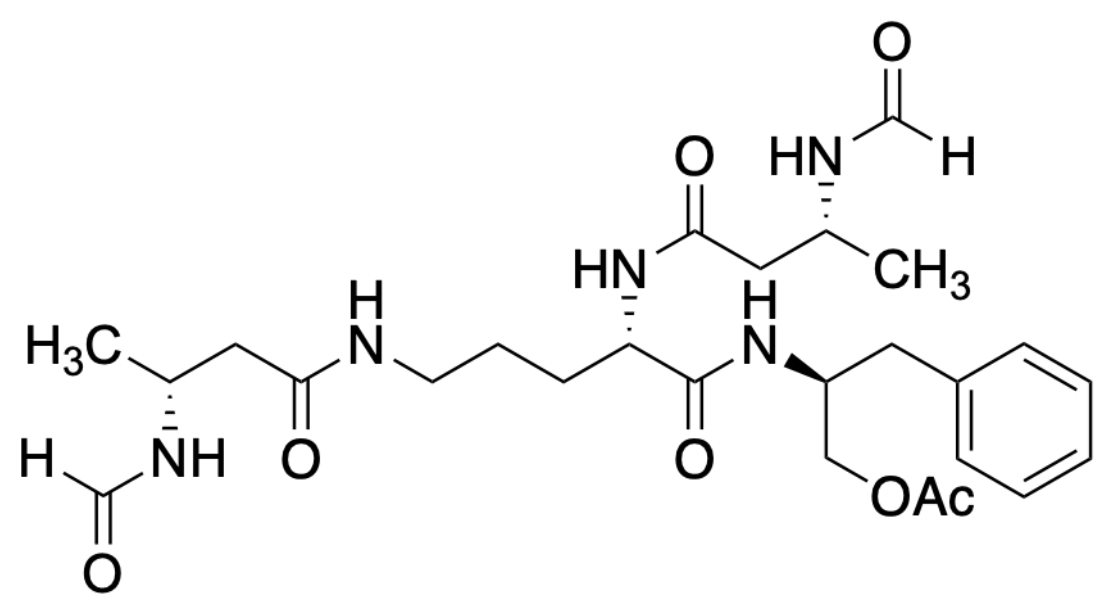

(*2S*)-2-{[(*2S*)-2,5-Bis{[(*3R*)-3-(formylamino)butanoyl]amino}\pentanoyl]amino}-3-phenyl-propyl acetate (28, C4-bisformamide). In a 5-mL roundbottom flask, the crude bis(trifluoroacetate) salt **21** (28.4 mg, 0.053 mmol, 1.0 equiv) and DIPEA (0.150 mL, 0.858 mmol, 16 equiv) were dissolved in 1 mL anhyd THF and cooled to 0 °C. 2,5-Dioxopyrrolidin-1-yl (*R*)-3-formamidobutanoate (**14a**), prepared as previously described,[4] (33.9 mg, 0.148 mmol, 2.8 equiv) and DMAP (3.8 mg, 0.0318 mmol, 0.6 equiv) were dissolved in 1 mL anhyd. THF and added to the stirring solution. The mixture was stirred at 0 °C for 5 min, warmed to 22 °C, and stirred for 11 h. The reaction mixture was filtered over a pad of celite that had been rinsed with Et_2O. The solid filtrate was triturated with Et_2O (3 x 10 mL) and concentrated by rotary evaporation to afford C4-bisformamide **28** as a white solid (0.0205 g, 0.0384 mmol, 73%), which was used without further purification.

TLC: R_f 0.29 (5% MeOH/CH_2Cl_2). **^{1}H-NMR** (600 MHz, DMSO-d_6): δ 7.95 (q, J = 9.1 Hz, 4H), 7.91 (t, J = 2.7 Hz, 2H), 7.87 (t, J = 5.7 Hz, 1H), 7.26 (t, J = 7.5 Hz, 2H), 7.19 (dd, J = 15.8, 7.5 Hz, 3H), 4.21 (h, J = 6.5 Hz, 1H), 4.14 (dp, J = 14.3, 7.0 Hz, 3H), 4.00 (dd, J = 11.0, 4.8 Hz, 1H), 3.87 – 3.78 (m, 1H), 2.99 (q, J = 6.9 Hz, 2H), 2.75 (qd, J = 13.9, 7.2 Hz, 2H), 2.29 (dp, J = 21.4, 6.7 Hz, 2H), 2.23 – 2.17 (m, 1H), 2.12 (dd, J = 13.9, 8.1 Hz, 1H), 1.98 (s, 3H), 1.52 (dq, J = 11.6, 6.4 Hz, 1H), 1.41 (dq, J = 12.5, 5.9 Hz, 1H), 1.39 – 1.27 (m, 2H), 1.24 (s, 1H), 1.09 (d, J = 6.6 Hz, 1H), 1.03 (t, J = 6.6 Hz, 5H). **^{13}C NMR** (151 MHz, DMSO-d_6): δ 170.78, 170.77, 169.64, 169.00, 168.99, 168.92, 168.90, 162.97, 162.92, 159.53, 159.50, 137.37, 137.35, 128.46, 128.44, 127.59, 125.58, 64.04, 51.61, 51.58, 51.44, 48.24, 44.58, 44.43, 42.94, 42.69, 41.34, 41.16, 40.47, 40.37, 37.52, 37.45, 35.94, 35.91, 29.16, 28.40, 25.01, 24.98, 24.86, 21.48, 21.14, 21.11, 19.98, 19.48, 19.38, 13.35. **HRMS** (ESI) *m/z* calcd for $C_{26}H_{40}N_6O_7$ ($[M+H]^+$) 534.2928; found 534.2902.

J. UV-VIS COMPETITION TITRATIONS OF 7 WITH $Cu(BCS)_2$

Preparation of HEPES buffer and $Cu(BCS)_2$ stock solution

A solution of 25 mM HEPES buffer (0.1 M NaCl, pH 7.2, MeCN:H2O v/v=1:10) was prepared by diluting commercial 1.0 M HEPES buffer (SigmaAldrich) with Milli-Q water and degassed MeCN. The diluted HEPES buffer was then degassed and deoxygenated using Ar. An aliquot of 60 µL of 10 mM stock solution of bathocuproinedisulfonic acid disodium salt (BCS) in degassed 25 mM HEPES buffer and 12 µL of 20 mM stock solution of $[Cu(MeCN)_4]PF_6$ in degassed MeCN was mixed and diluted using 25 mM HEPES buffer to the final volume of 6 mL of 50 µM $Cu(BCS)_2$.

Competitive titration experiments of $Cu(BCS)_2$ with diisonitrile 7

Stock solutions of 1 mM, 2 mM, 3 mM, 4 mM, 5 mM, 6 mM, 7 mM, 8 mM, 9 mM, 10 mM, and 15 mM diisonitrile **7** were prepared in 100 µL degassed, anhydrous DMSO each. Aliquots of 4.5 µL of each stock solution of diisonitrile **7** were added to aliquots of 445.5 µL of the $Cu(BCS)_2$ solution. Samples of 100 µL of each final solution were analyzed by UV-Vis.

Competitive titration serial dilution experiments of $Cu(BCS)_2$ with diisonitrile 7

A solution of 200 µL 135 mM diisonitrile **7** in DMSO was serially diluted by a factor of 1.14 over 14 wells in a 96-well plate coated in Ar. An aliquot of 3,000 µL of 10 mM stock solution of Bathocuproinedisulfonic acid disodium salt (BCS) in degassed 25 mM HEPES buffer and 600 µL of 20 mM stock solution of $[Cu(MeCN)_4]PF_6$ in degassed MeCN was mixed and diluted using 25 mM HEPES buffer to the final volume of 12 mL of 1,000 µM $Cu(BCS)_2$. 4 µL of each dilution of diisonitrile **7** was added to 396 µL 1000 µM $Cu(BCS)_2$ in a separate 96-well plate. Samples of 100 µL of each final solution were analyzed by UV-Vis in a Costar 96-well black, clear-bottom multiplate.

Absorbance was measured from 351 nm to 551 nm on a UV plate reader. The extinction coefficient of $Cu(BCS)_2$ without diisonitrile **7** (ε = 8,344.3 $M^{-1}cm^{-1}$) was calculated using the Beer–Lambert law (λmax = 483 nm, $[BCS_2{\cdot}Cu]$ = 1 mM, b = 0.3 cm).* Each absorbance from the competition titration was then converted to concentration of Cu(BCS)2 using the calculated extinction coefficient (λmax = 483 nm, ε = 8,344.3 $M^{-1}cm^{-1}$, b = 0.3 cm). The data at 483 nm were plotted on GraphPad Prism. We confirmed separately that the Cu(I) complex with diisonitrile **7** has no absorbance at 483 nm.

*Using the Costar 96-well black, clear-bottom multiplate for the UV-Vis measurements, raw absorbance was recorded and the pathlength of the 100 µL solution was 0.3 cm. see: http://sites.psu.edu/msproteomics/wpcontent/uploads/sites/7129/2014/05/User-Manual-SpectraMax-M3-M4-M5-M5e-.pdf

K. MYCOBACTERIAL GROWTH ASSAYS

Growth assays were carried out essentially as previously described.[2] Stock solutions of TTM (50 mM) and diisonitriles (25 mM) were dissolved in DMSO. The indicated strains were pre-grown in 7H9-ADS (albumin, dextrose, saline) media until reaching an OD_{600} value of ~1.0. Cells were then collected by centrifugation (3700 × *g*, 10 min) and washed twice with Chelex-demetallated phosphate buffered saline + 0.05% Tween-80 (PBS Tween-80). Growth assays in 96-well plates were initiated at a calculated OD_{600} of 0.005 by the addition of 0.02 mL of washed culture at an OD_{600} 0.05 to 0.18 mL of 7H9-ADS media, which had been Chelex-demetallated and reconstituted with defined concentrations of Cu(II), Zn(II), Fe(III), Mg(II), and Ca(II), as previously described.[2] Prior to inoculating, each well contained 22 μM TTM and the indicated serial dilution of chalkophore analogue at 1.1x final assay concentration. Growth at 37 °C was assayed via daily OD_{600} measurements in a 96-well plate reader. Reported data is the average and SEM of n = 3 independent biological experiments with n = 2 technical replicates in each.

L. ^{1}H-NMR AND ^{13}C-NMR SPECTRA

1. SYNTHESIS OF DIISONITRILE SIDECHAINS 15b–f **S47–S71**

a. Synthesis of C6 Sidechain **15b** (**9b–15b**) S47–S51
b. Synthesis of C8 Sidechain **15c** (**9c–15c**) S52–S56
c. Synthesis of C10 Sidechain **15d** (**9d–15d**) S57–S61
d. Synthesis of C12 Sidechain **15e** (**9e–15e**) S62–S66
e. Synthesis of C14 Sidechain **15f** (**9f–15f**) S67–S71

2. **SYNTHESIS OF DIISONITRILES 7, 22b-f, 23a,f** **S72–S82**

a. Synthesis of C4-Orn-Phe-OAc, **7 (17, 19, 20, 7)** S72–S75
b. Synthesis of C6-Orn-Phe-OAc, **22b** S76
c. Synthesis of C8-Orn-Phe-OAc, **22c** S77
d. Synthesis of C10-Orn-Phe-OAc, **22d** S78
e. Synthesis of C12-Orn-Phe-OAc, **22e** S79
f. Synthesis of C14-Orn-Phe-OAc, **22f** S80
g. Synthesis of C4-Orn-Phe-OH, **23a** S81
h. Synthesis of C14-Orn-Phe-OH, **23f** S82

3. **SYNTHESIS OF DIISONITRILE ANALOGUES 24-28** **S83–S95**

a. Synthesis of C4-Orn-OAc, **24 (S1, S2, 24)** S83–S85
b. Synthesis of C4-Lys-Phe-OAc, **25 (S5-S7, 25)** S86–S89
c. Synthesis of C4-cadaverine, **26** S90
d. Synthesis of *S,S*-C4-Orn-Phe-OAc, **27 (S11-S13, 27)** S91–S94
e. Synthesis of C4-bisformamide, **28** S95

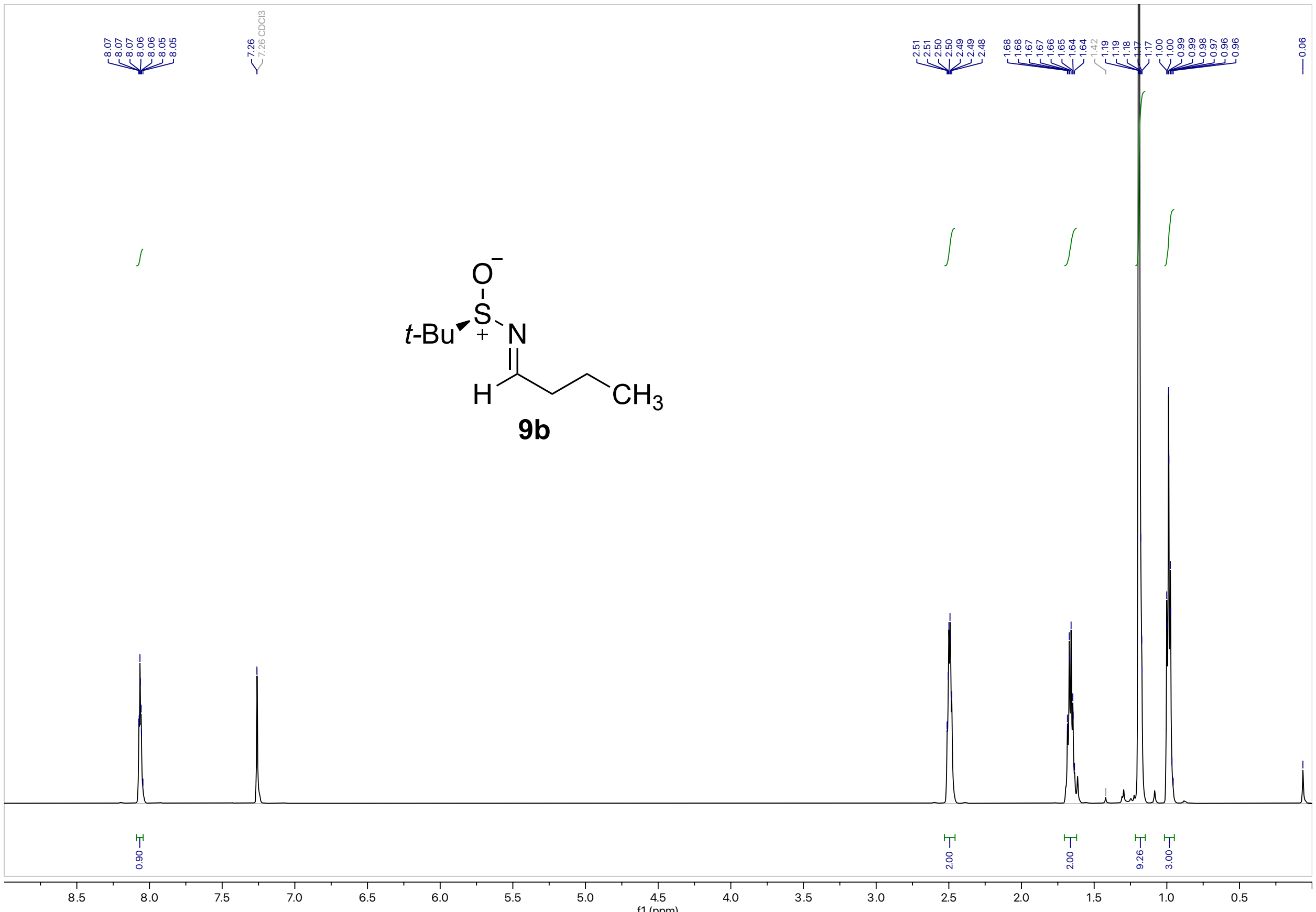
t-Bu
9b
0.90
2.00
2.00
9.26
3.00
f1 (ppm)

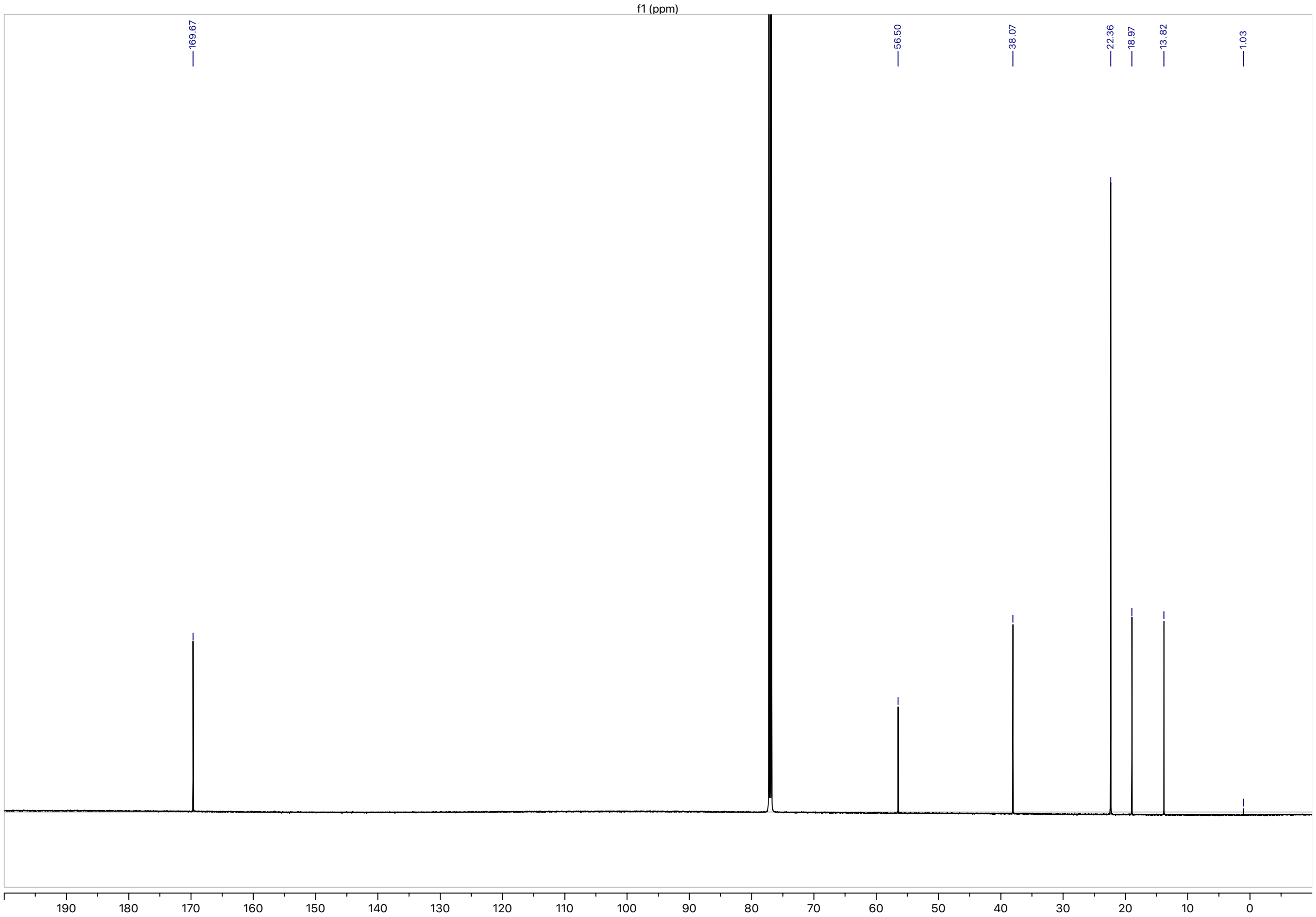
169.67
56.50
38.07
22.36
18.97
13.82
1.03
f1 (ppm)

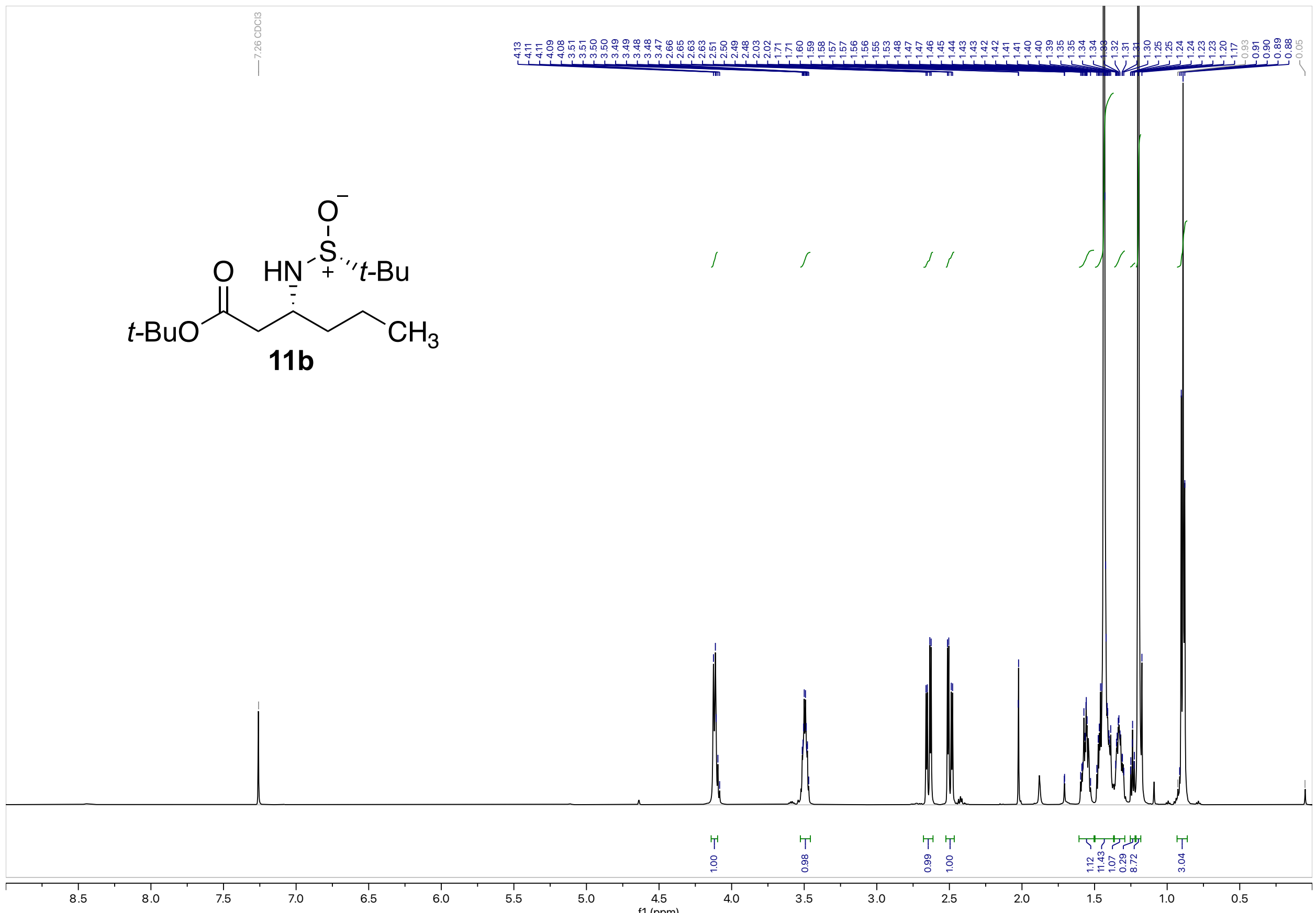
O
HN
S
+
t-Bu
t-BuO
CH3
11b
f1 (ppm)

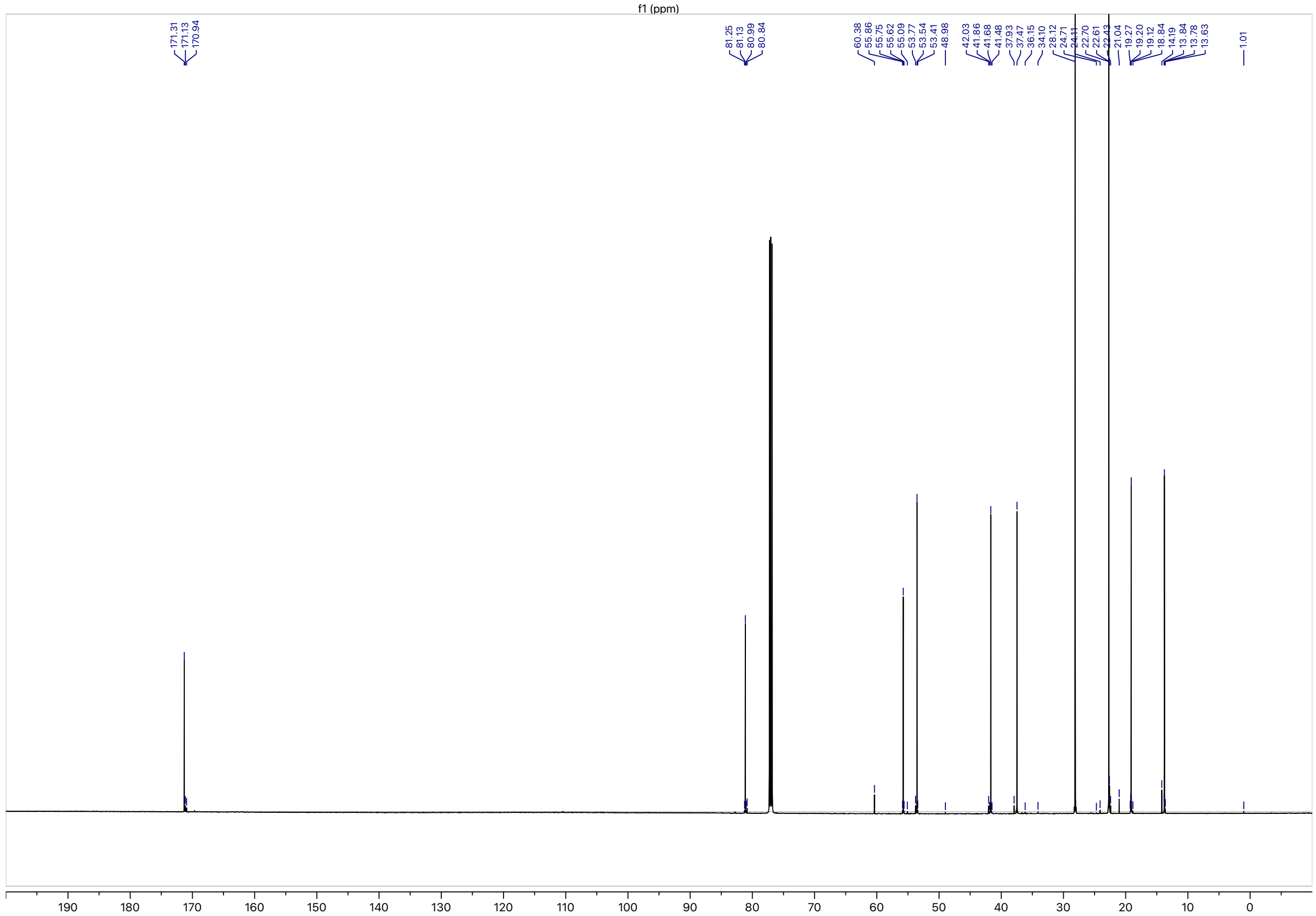
f1 (ppm)

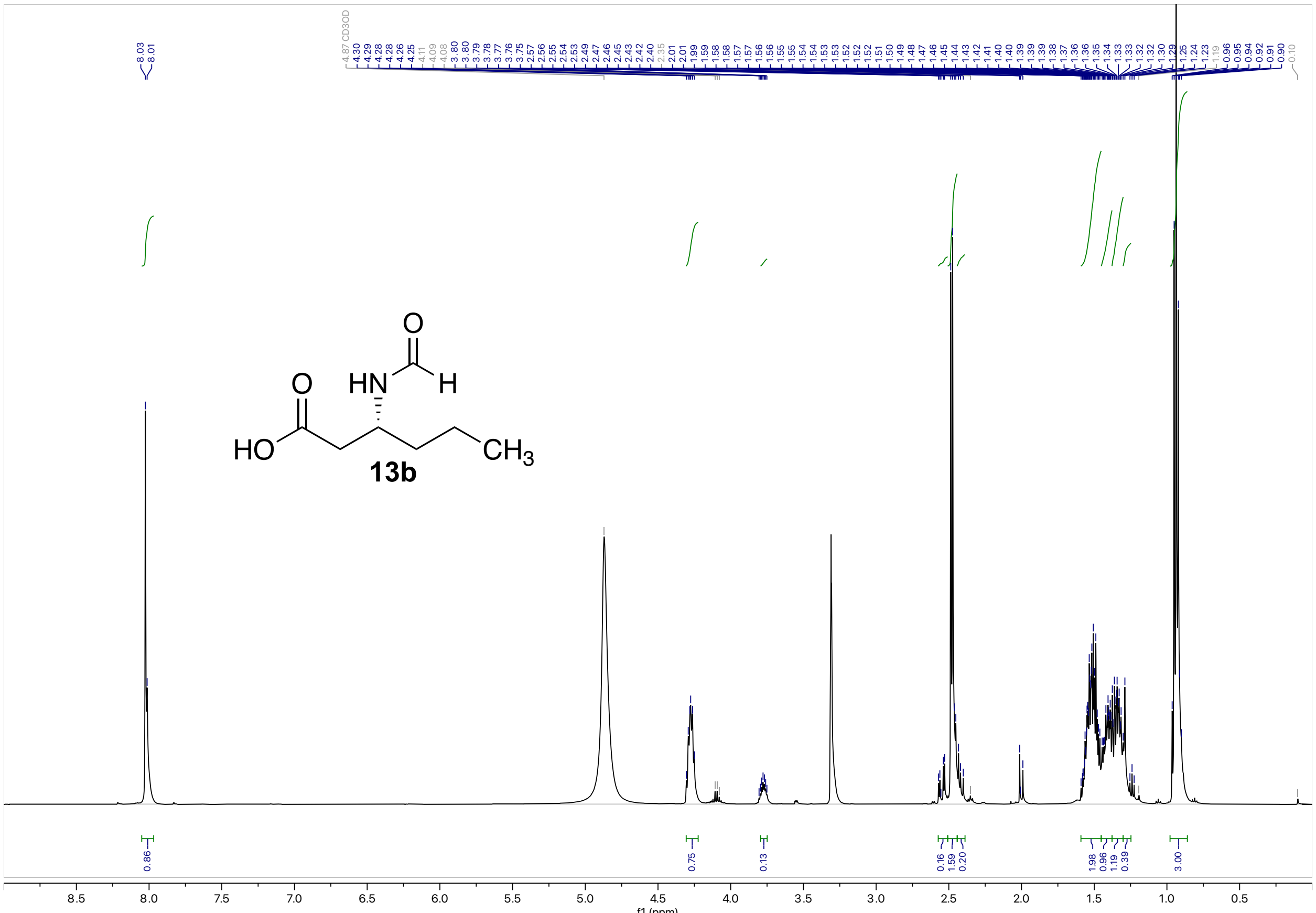
8.03
8.01
13b
HO
HN
O
H
CH3
0.86
0.75
0.13
0.16
1.59
0.20
1.98
0.96
1.19
0.39
3.00
8.5
8.0
7.5
7.0
6.5
6.0
5.5
5.0
4.5
4.0
3.5
3.0
2.5
2.0
1.5
1.0
0.5
f1 (ppm)

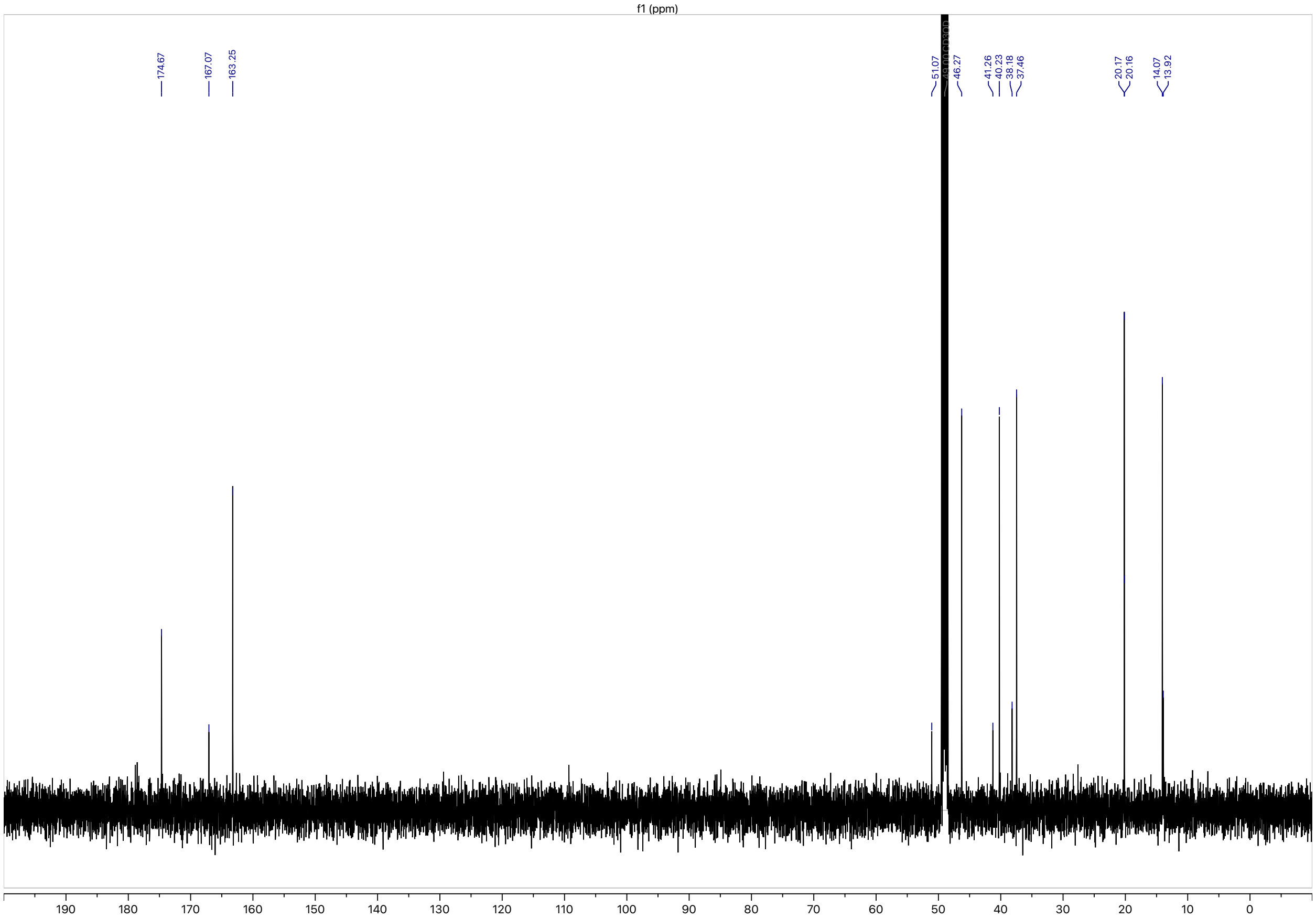
174.67
167.07
163.25
51.07
46.27
41.26
40.23
38.18
37.46
20.17
20.16
14.07
13.92
190
180
170
160
150
140
130
120
110
100
90
80
70
60
50
40
30
20
10
0
f1 (ppm)

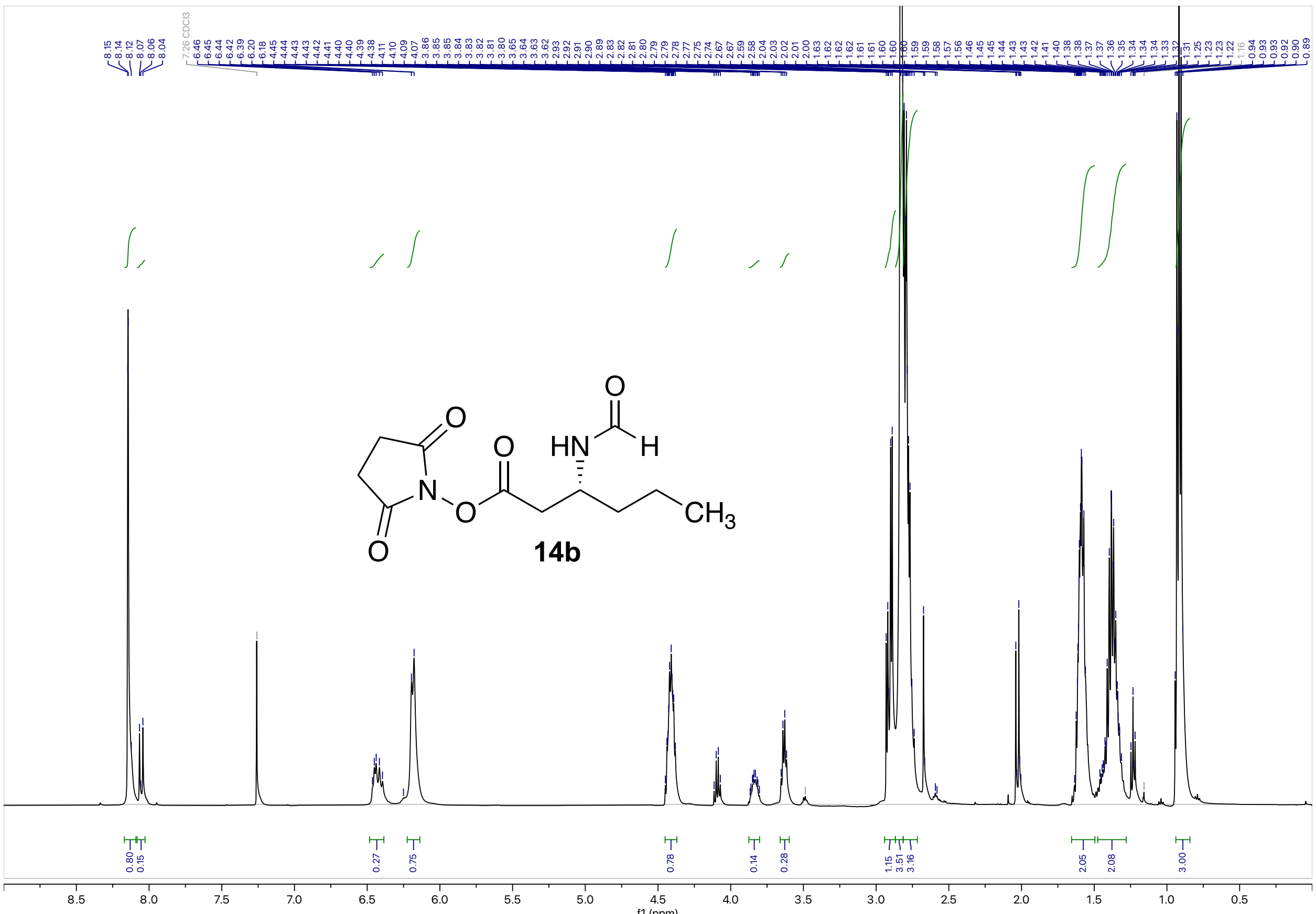
HN
O
H
O
O
N
O
O
CH3
14b
f1 (ppm)

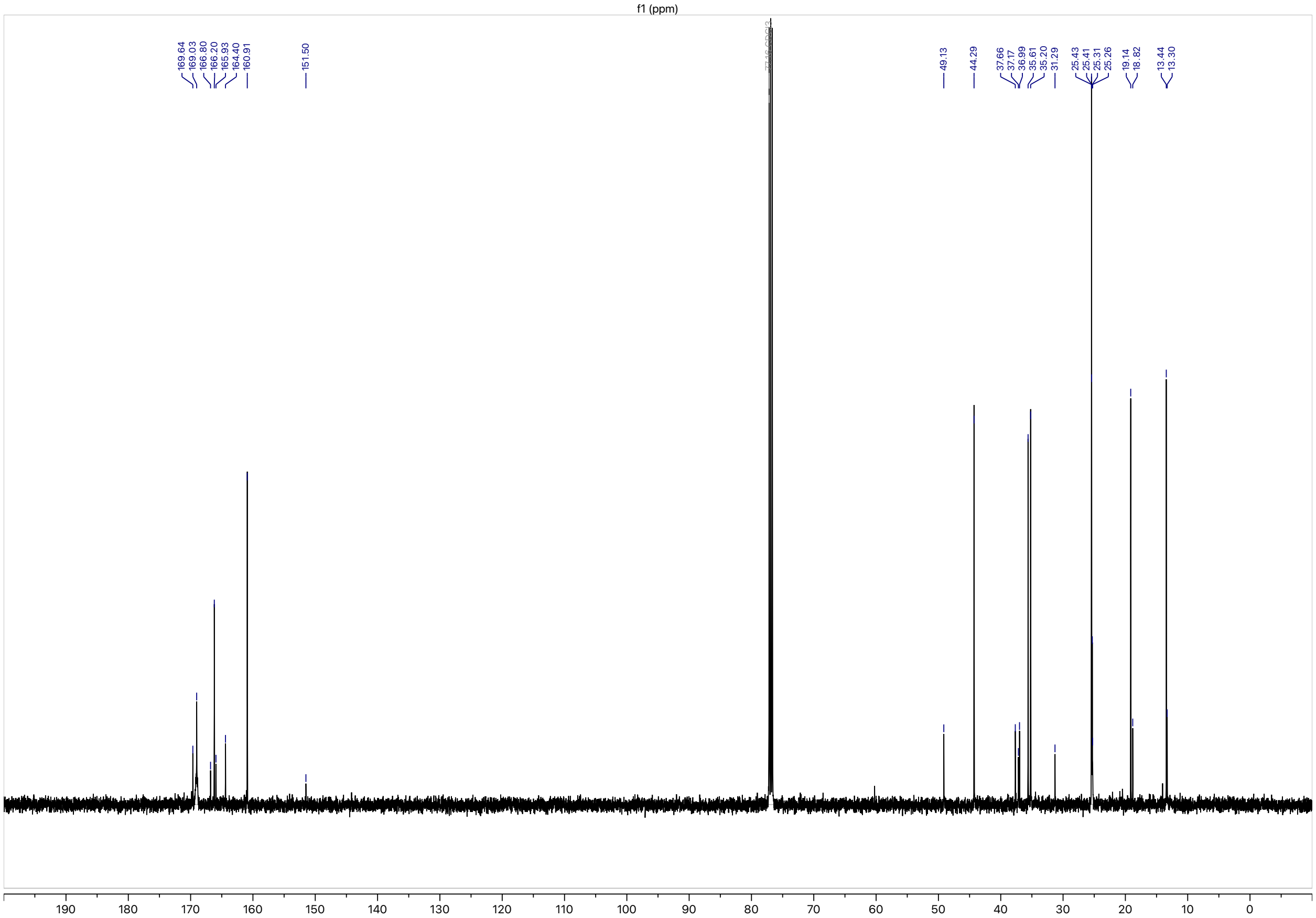
169.64
169.03
166.80
166.20
165.93
164.40
160.91
151.50
49.13
44.29
37.66
37.17
36.99
35.61
35.20
31.29
25.43
25.41
25.31
25.26
19.14
18.82
13.44
13.30
f1 (ppm)

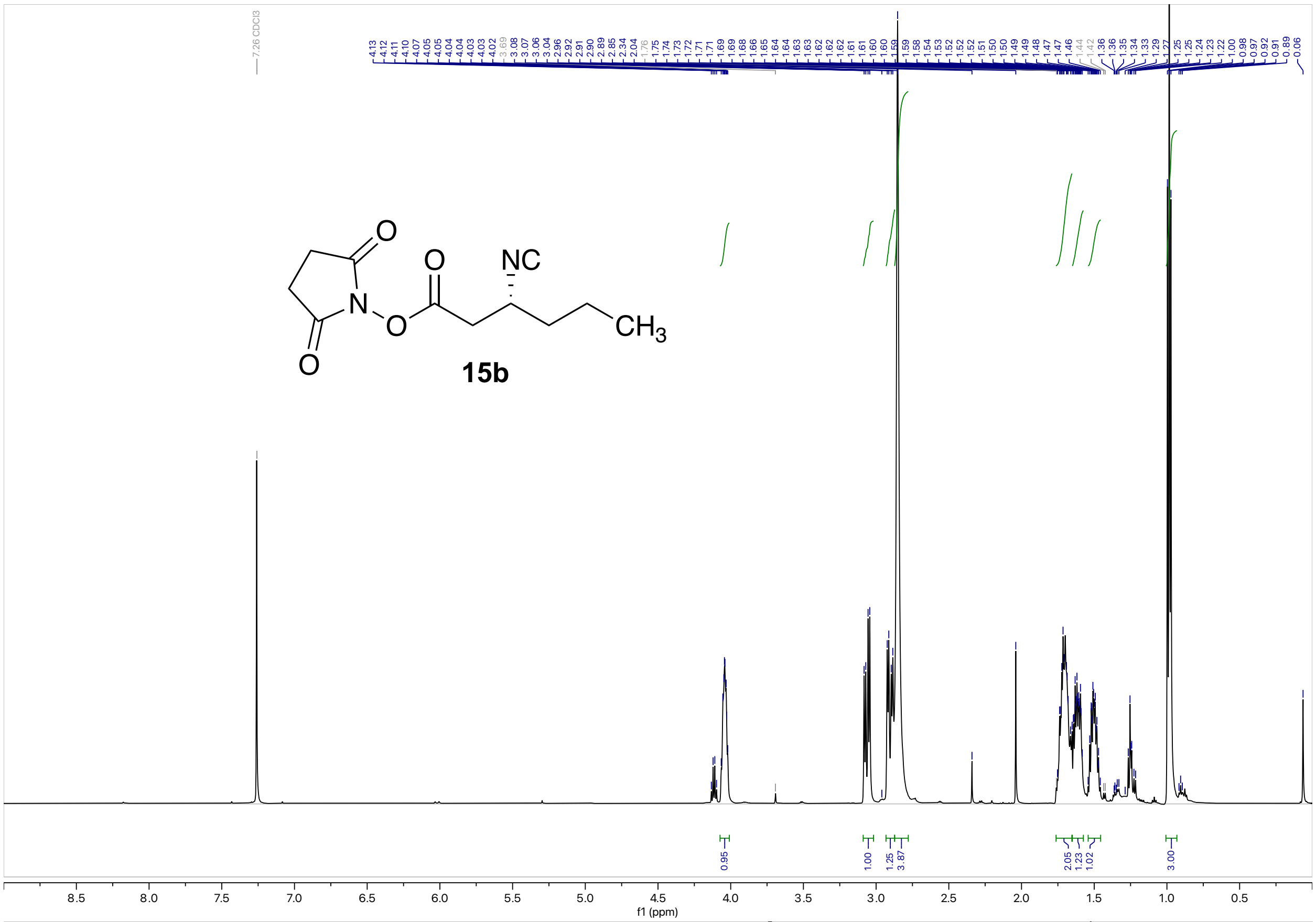
7.26 CDCl3
NC
O
O
N
O
O
O
CH3
15b
0.95
1.00
1.25
3.87
2.05
1.23
1.02
3.00
f1 (ppm)

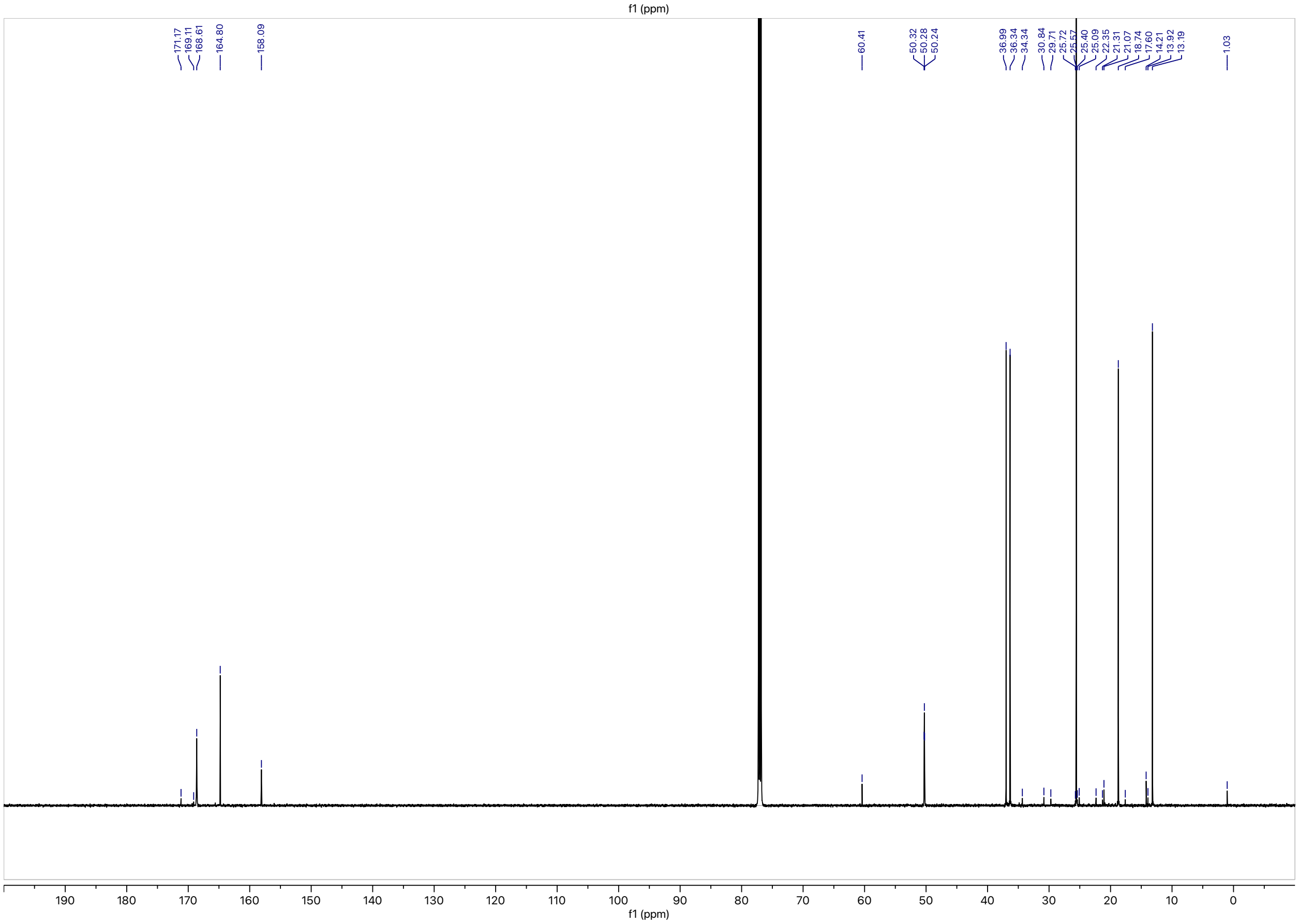
171.17
169.11
168.61
164.80
158.09
60.41
50.32
50.28
50.24
36.99
36.34
34.34
30.84
29.71
25.72
25.57
25.40
25.09
22.35
21.31
21.07
18.74
17.60
14.21
13.92
13.19
1.03
f1 (ppm)

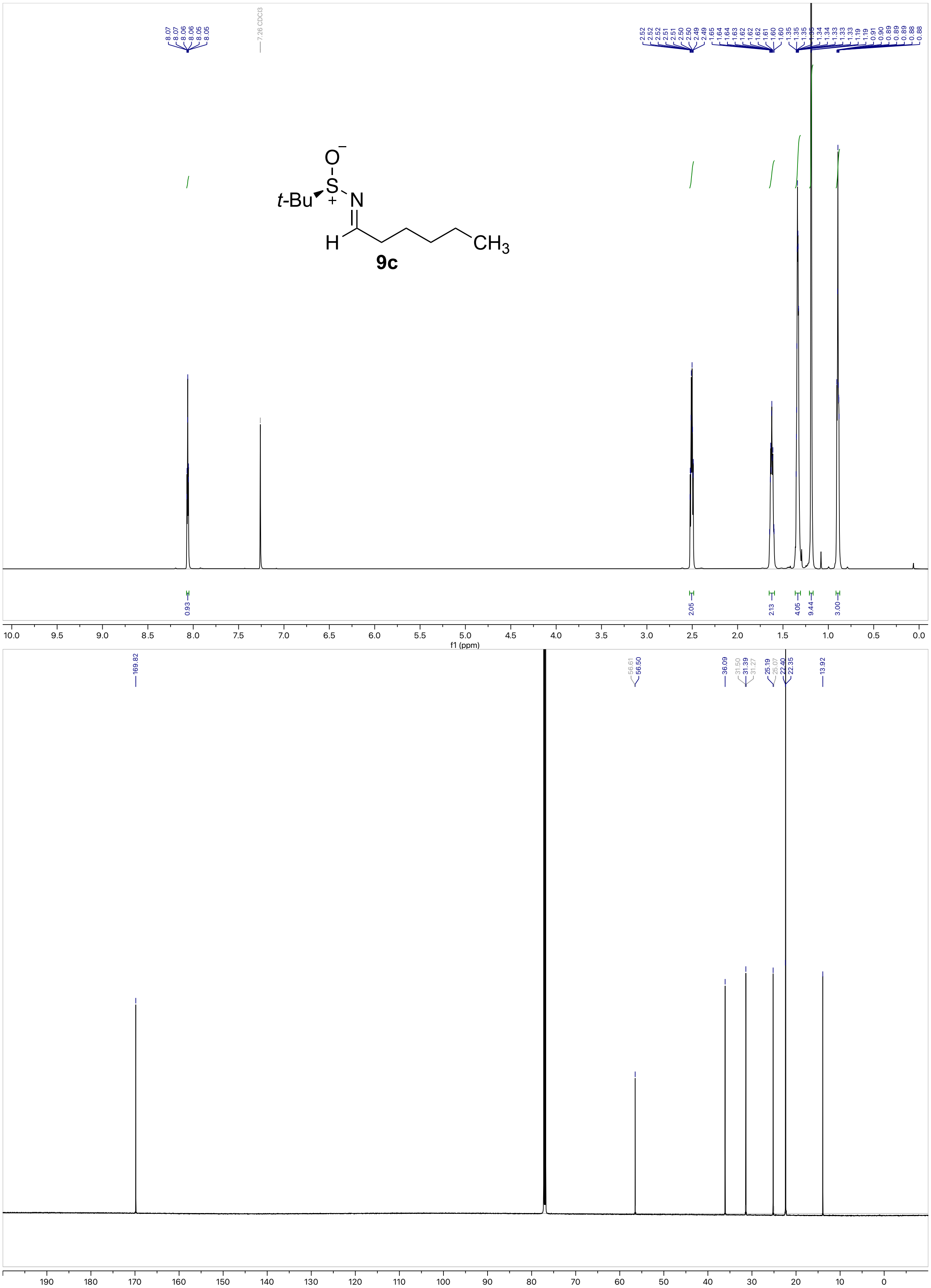
8.07
8.07
8.06
8.06
8.05
8.05
7.26 CDCl3
2.52
2.52
2.51
2.51
2.50
2.50
2.49
2.49
1.65
1.64
1.64
1.63
1.62
1.62
1.61
1.60
1.60
1.35
1.35
1.35
1.34
1.34
1.33
1.33
1.19
1.19
0.91
0.90
0.89
0.89
0.88
0.88
O
S
N
t-Bu
+
H
CH3
9c
0.93
2.05
2.13
4.05
9.44
3.00
10.0
9.5
9.0
8.5
8.0
7.5
7.0
6.5
6.0
5.5
5.0
4.5
4.0
3.5
3.0
2.5
2.0
1.5
1.0
0.5
0.0
f1 (ppm)
169.82
56.61
56.50
36.09
31.50
31.39
31.27
25.19
25.07
22.40
22.35
13.92
190
180
170
160
150
140
130
120
110
100
90
80
70
60
50
40
30
20
10
0
f1 (ppm)

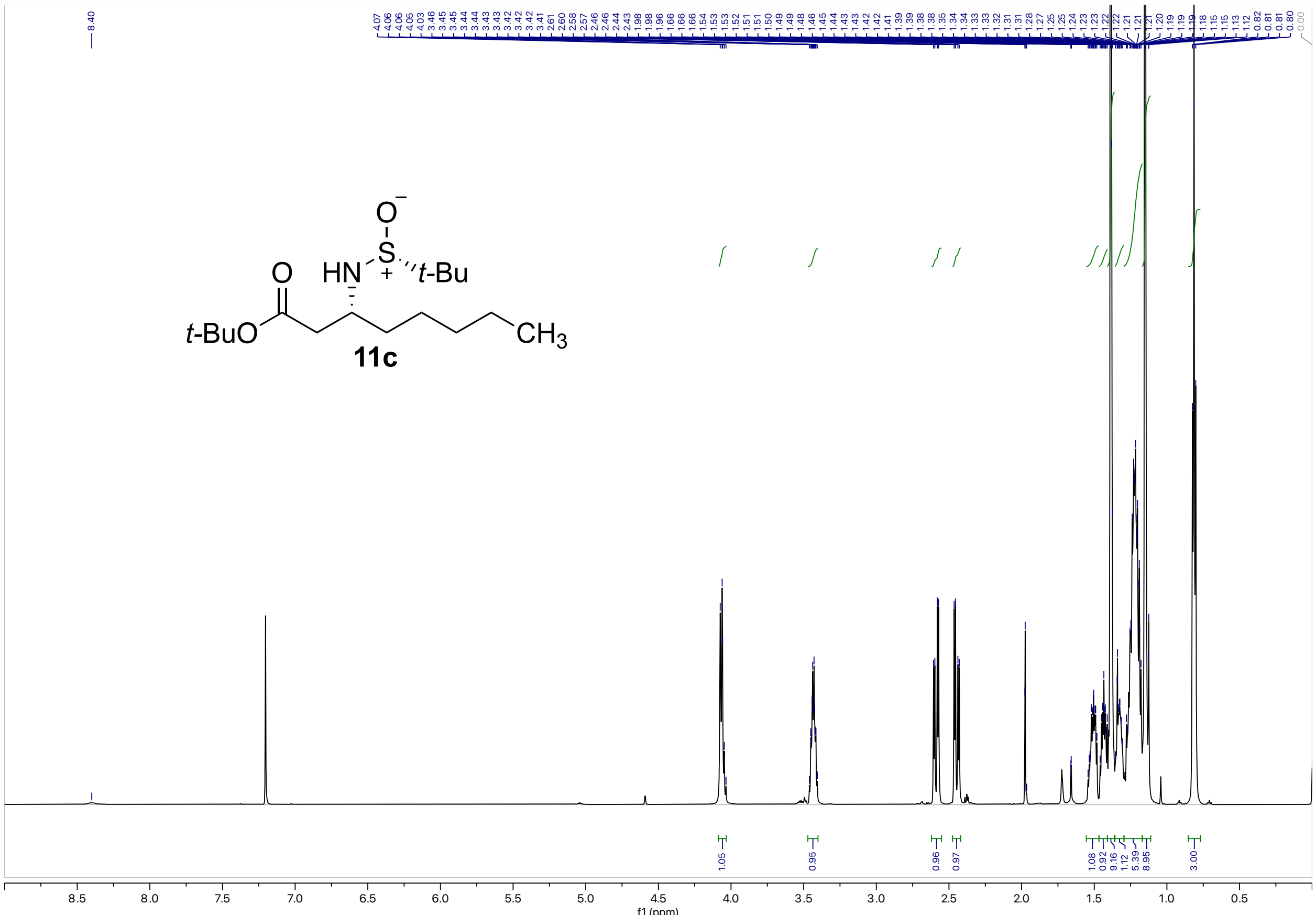

O
HN
S
+
t-Bu
t-BuO
CH3
11c
f1 (ppm)

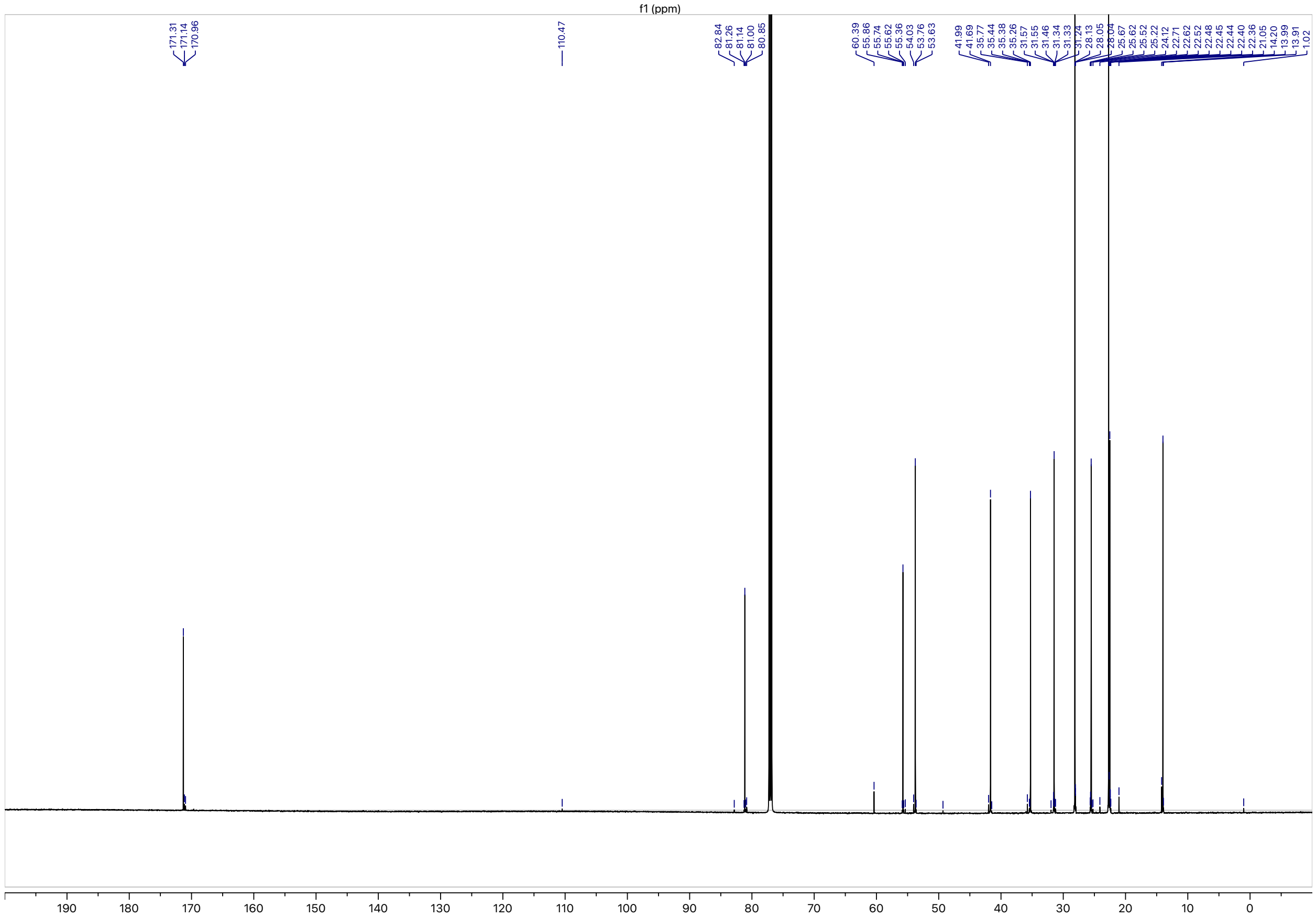

f1 (ppm)

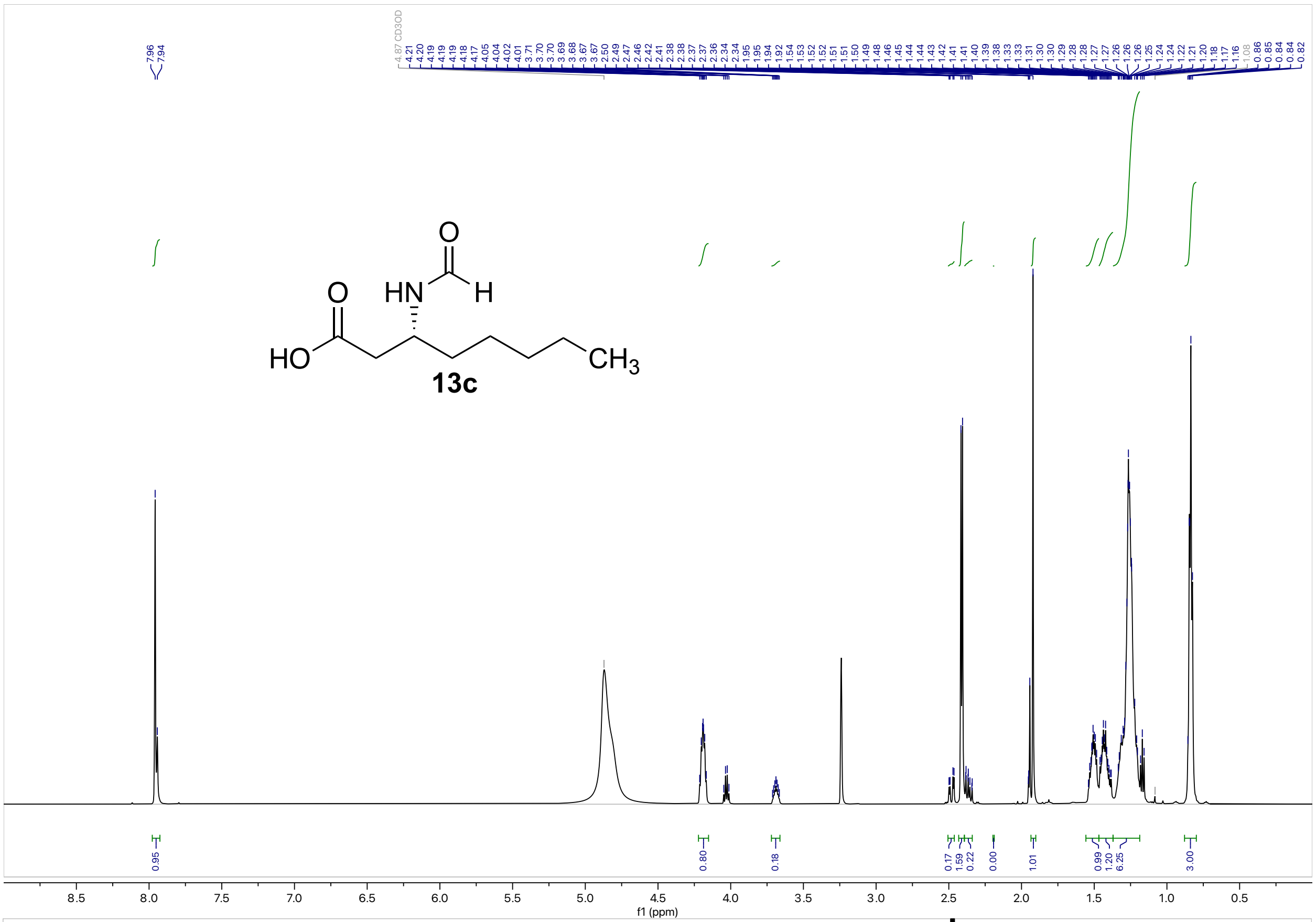
O
HN
H
O
HO
CH3
13c
f1 (ppm)

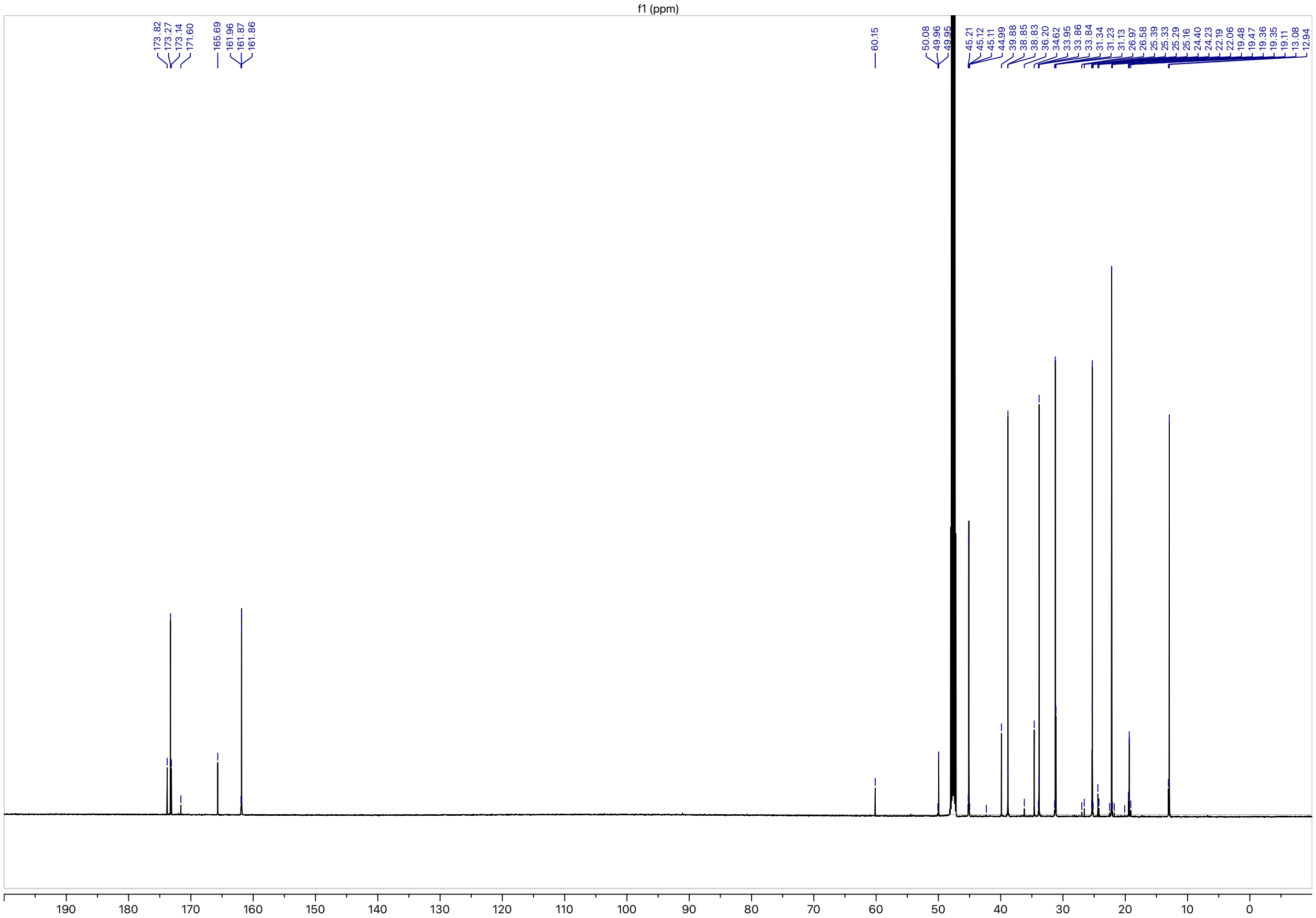
f1 (ppm)

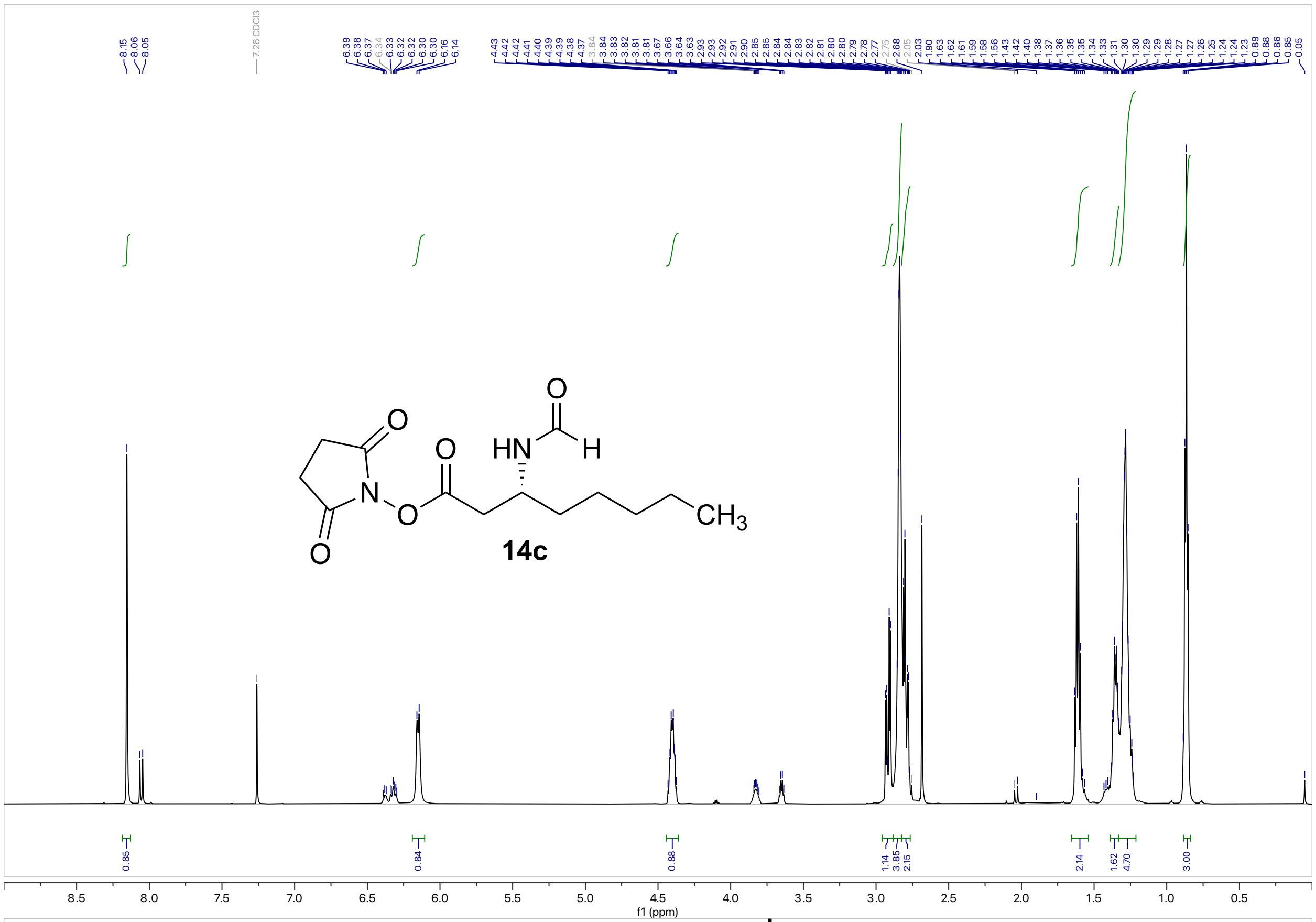
7.26 CDCl3
14c
0.85
0.84
0.88
1.14
3.85
2.15
2.14
1.62
4.70
3.00
8.5
8.0
7.5
7.0
6.5
6.0
5.5
5.0
4.5
4.0
3.5
3.0
2.5
2.0
1.5
1.0
0.5
f1 (ppm)

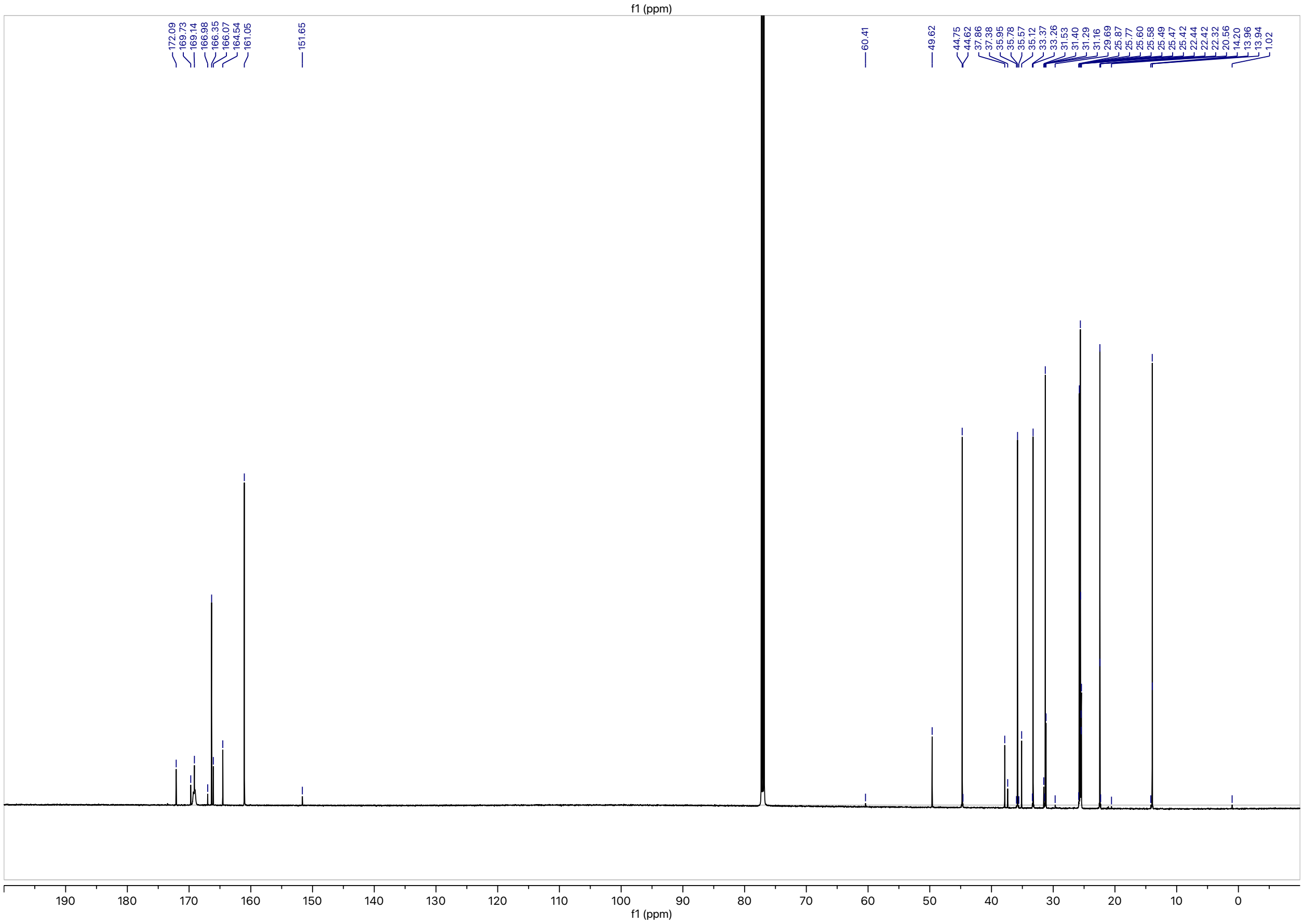
190
180
170
160
150
140
130
120
110
100
90
80
70
60
50
40
30
20
10
0
f1 (ppm)

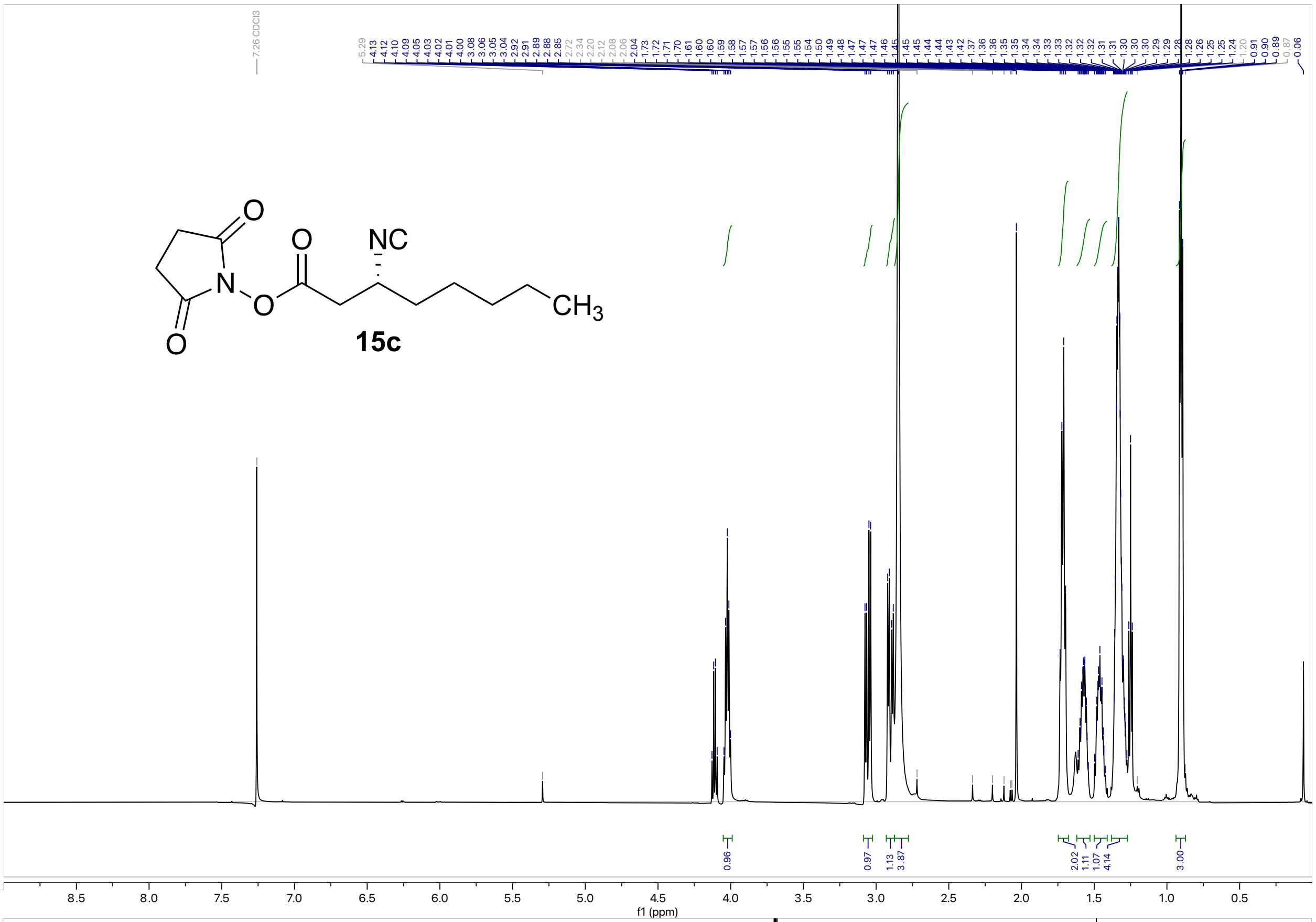
7.26 CDCl3
O
O
NC
N
O
CH3
15c
0.96
0.97
1.13
3.87
2.02
1.11
1.07
4.14
3.00
f1 (ppm)

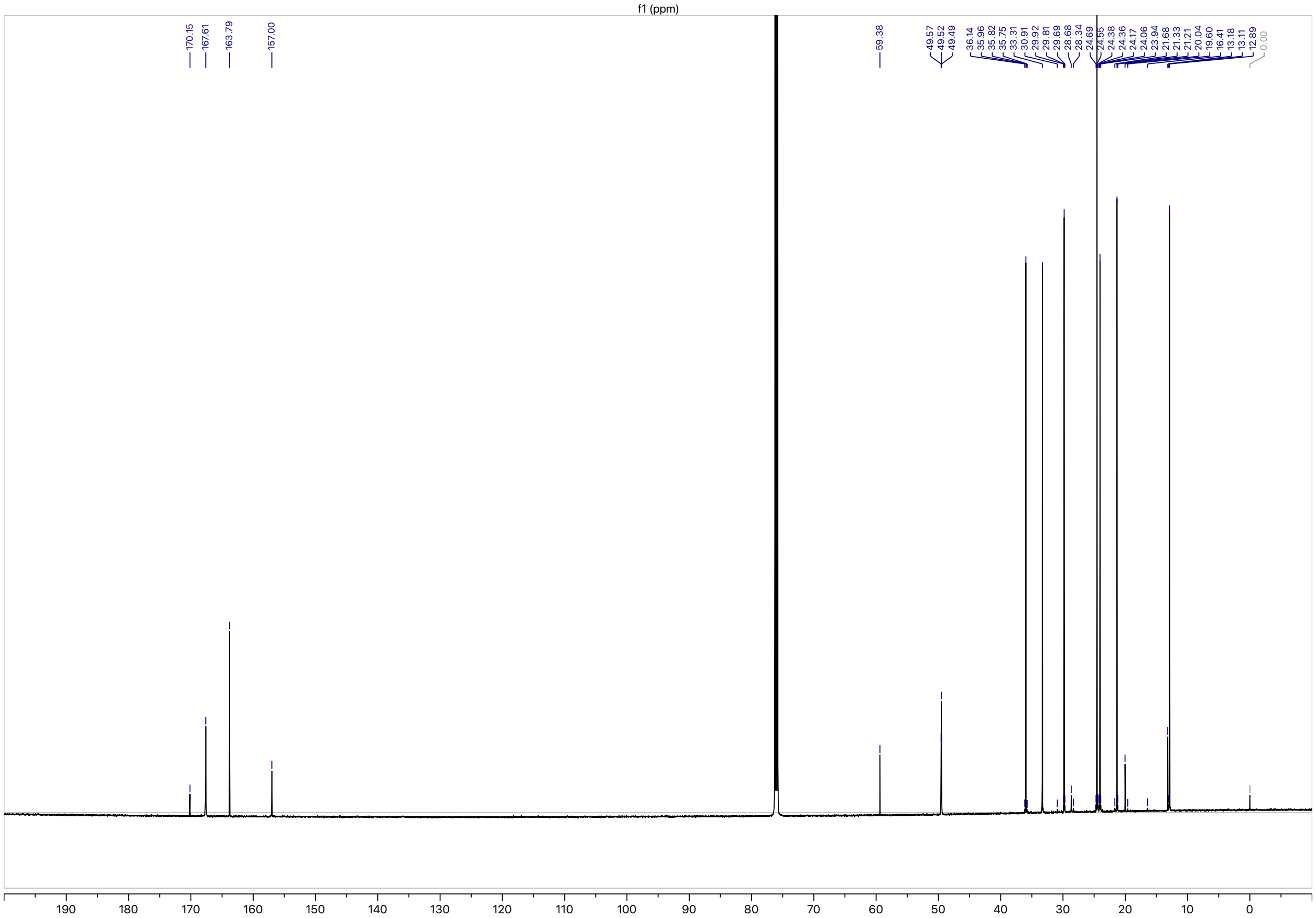
170.15
167.61
163.79
157.00
59.38
f1 (ppm)

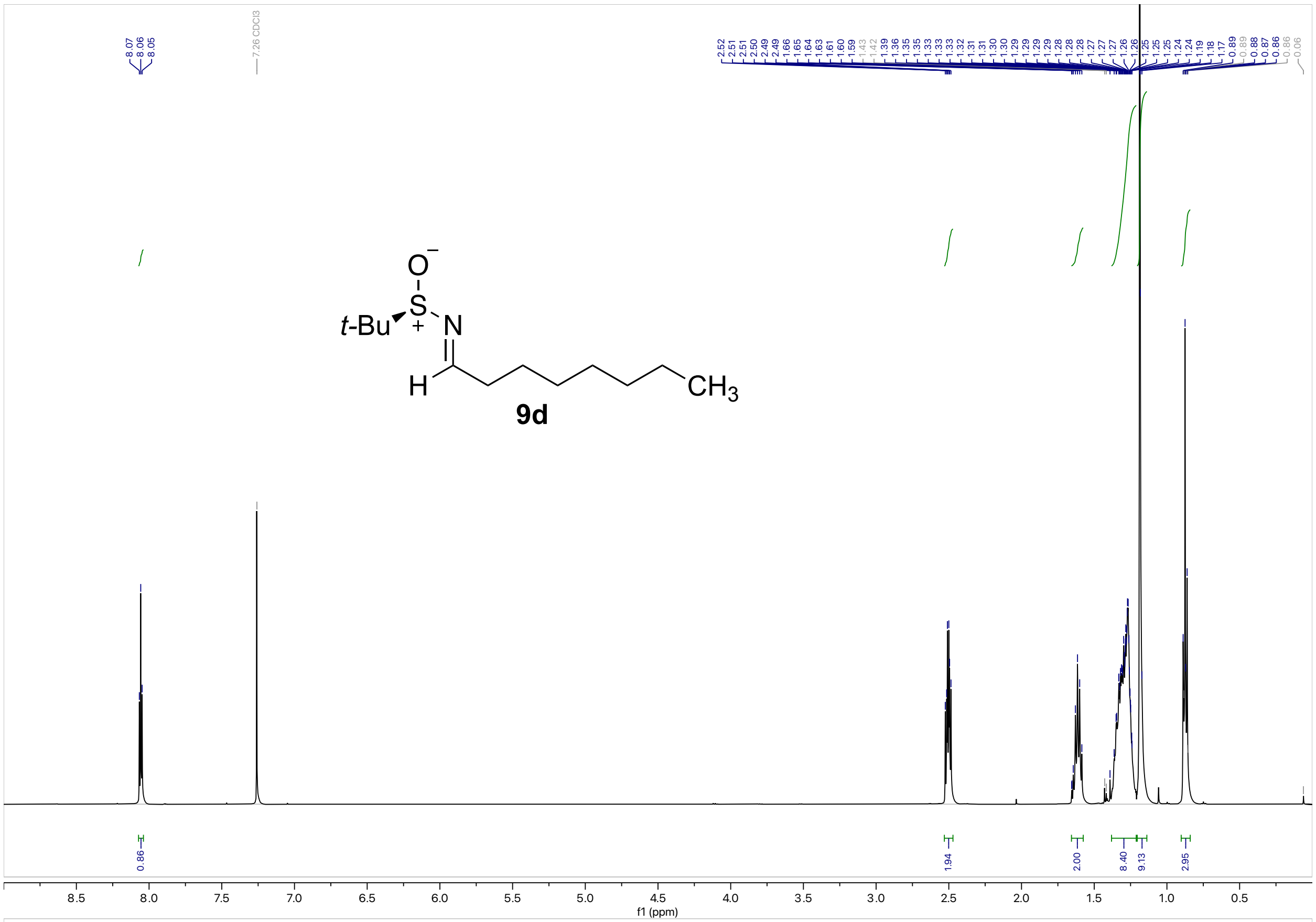
t-Bu
S
O
N
H
CH3
9d
f1 (ppm)

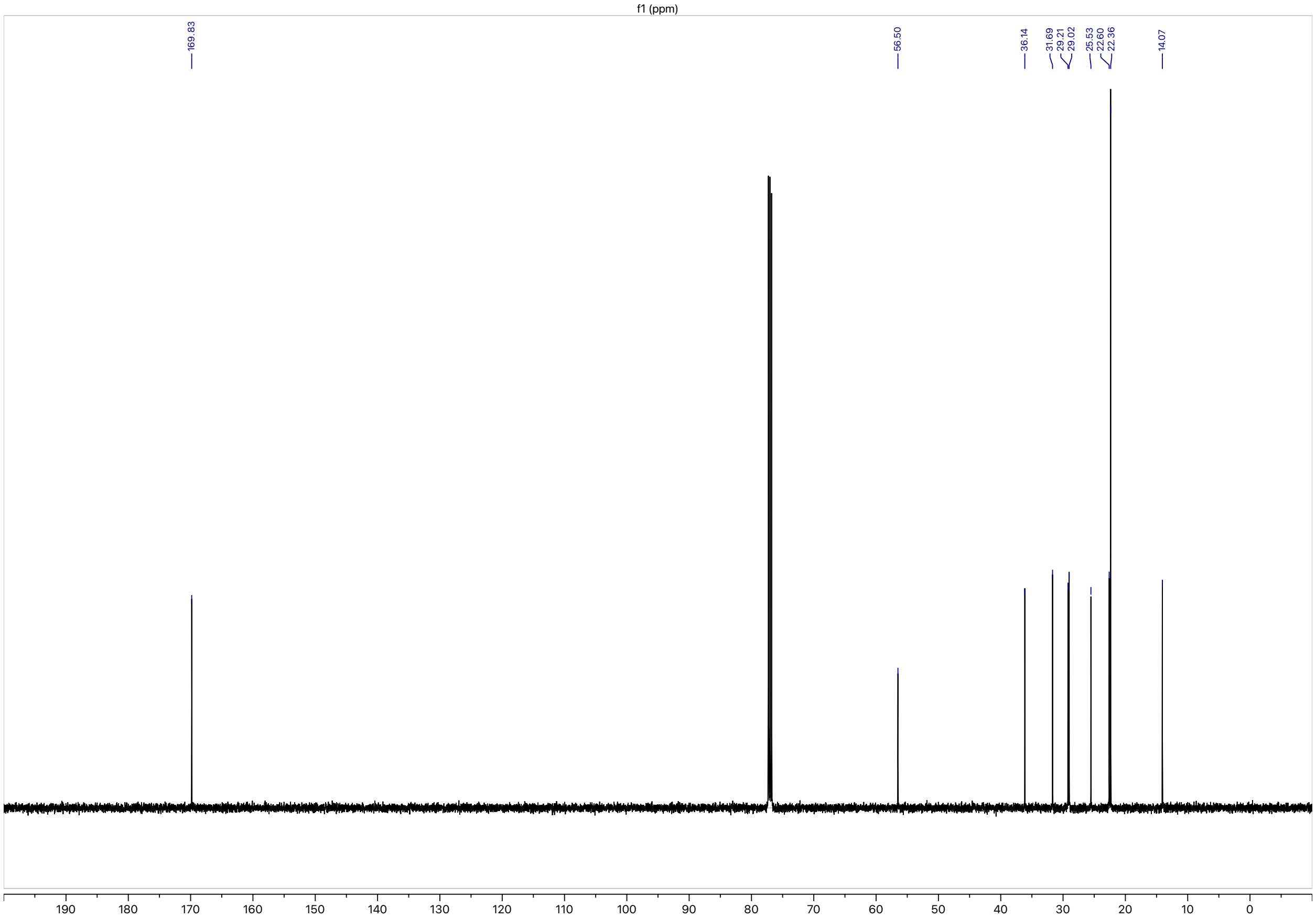
169.83
56.50
36.14
31.69
29.21
29.02
25.53
22.60
22.36
14.07
f1 (ppm)

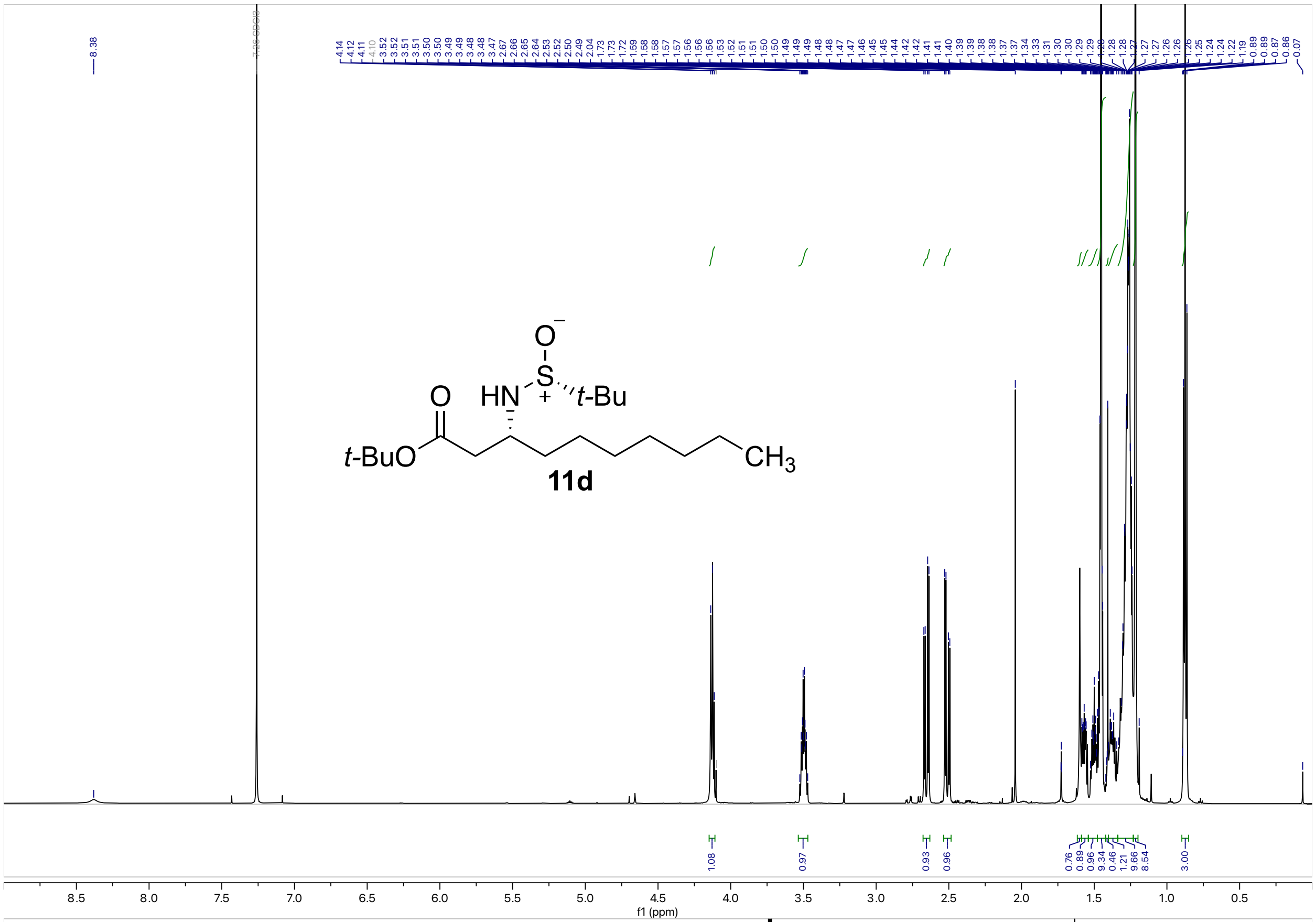
O
HN
S
t-Bu
O
t-BuO
CH3
11d
f1 (ppm)

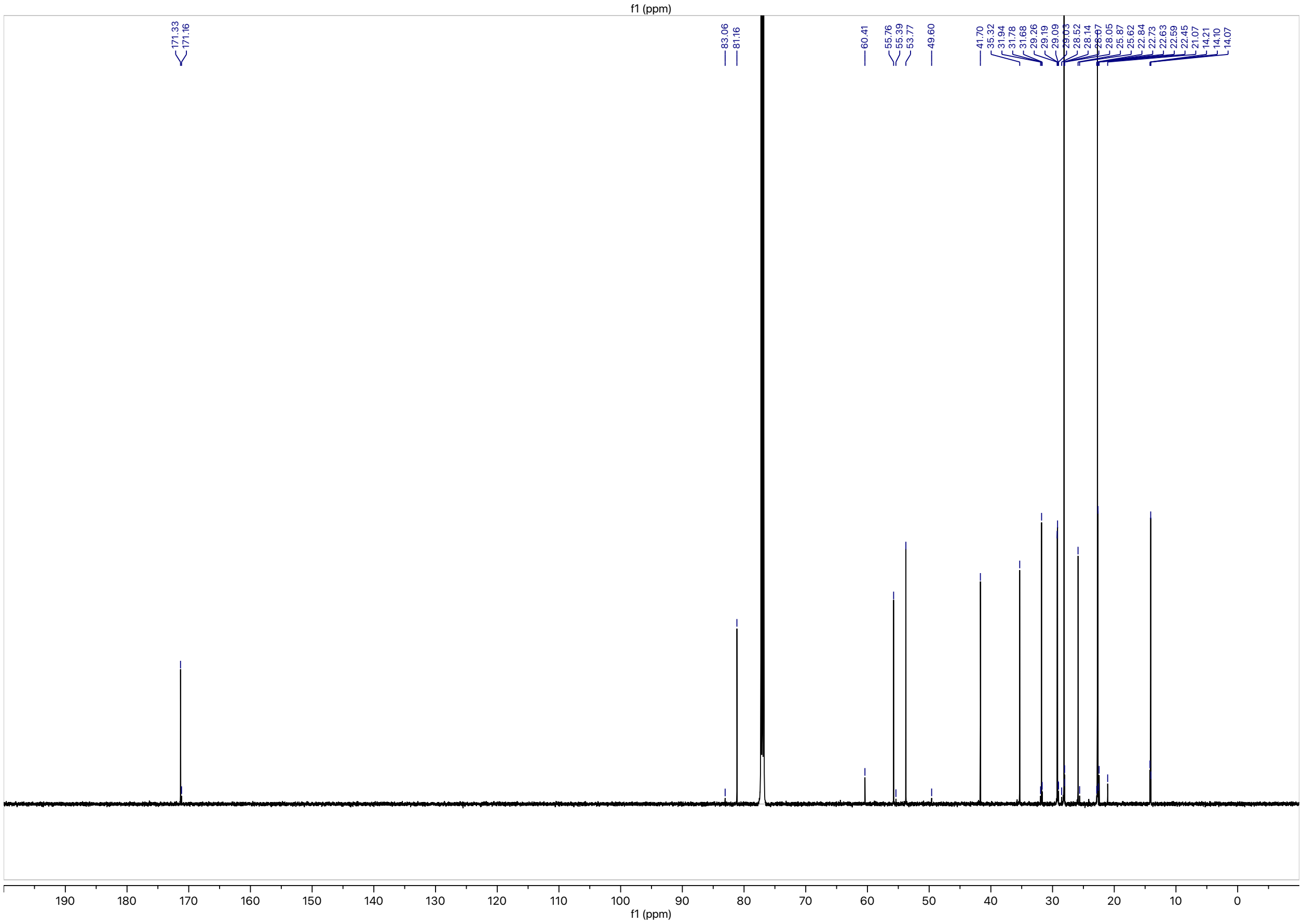
f1 (ppm)

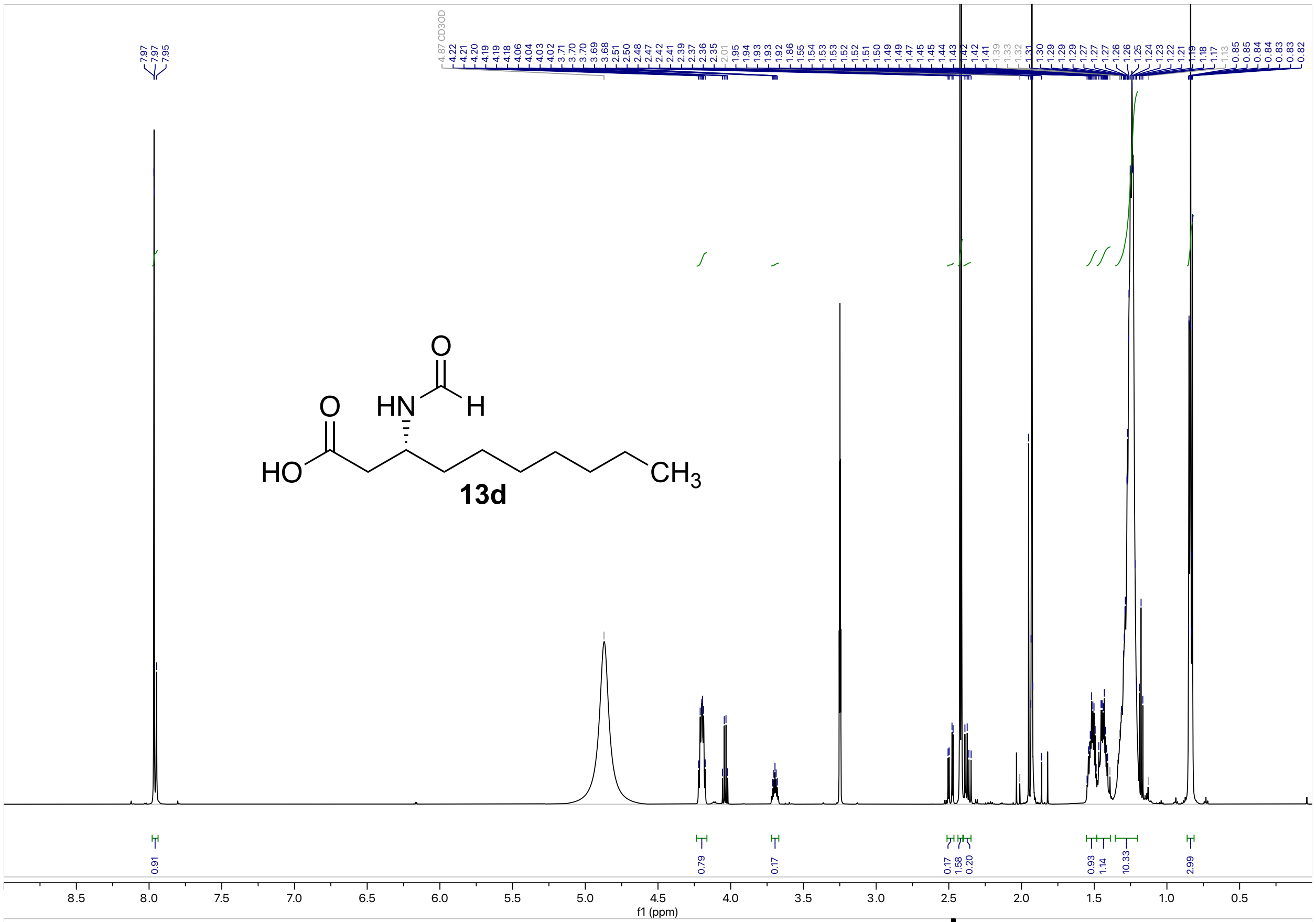
HO
O
HN
O
H
CH3
13d
f1 (ppm)

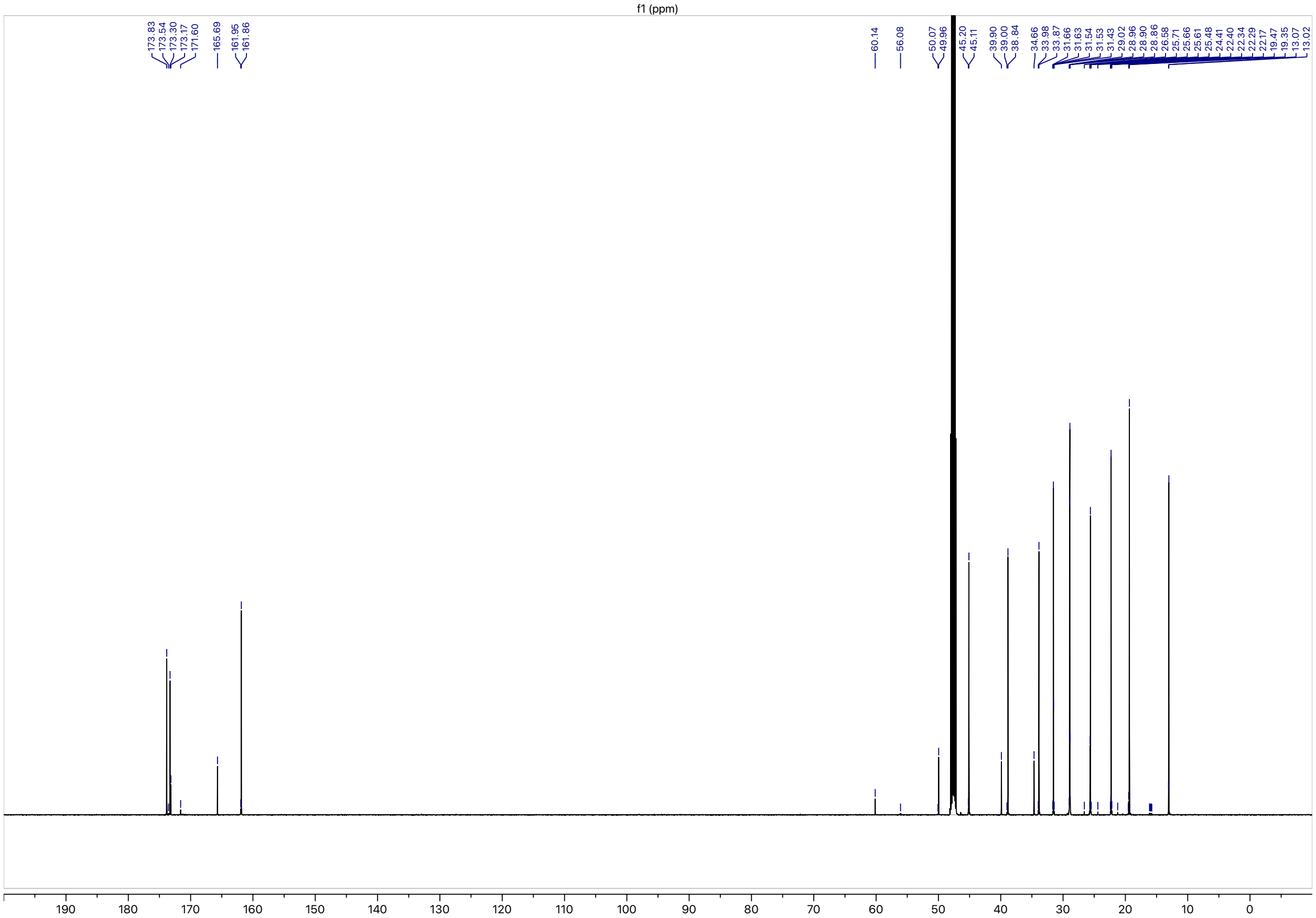
f1 (ppm)

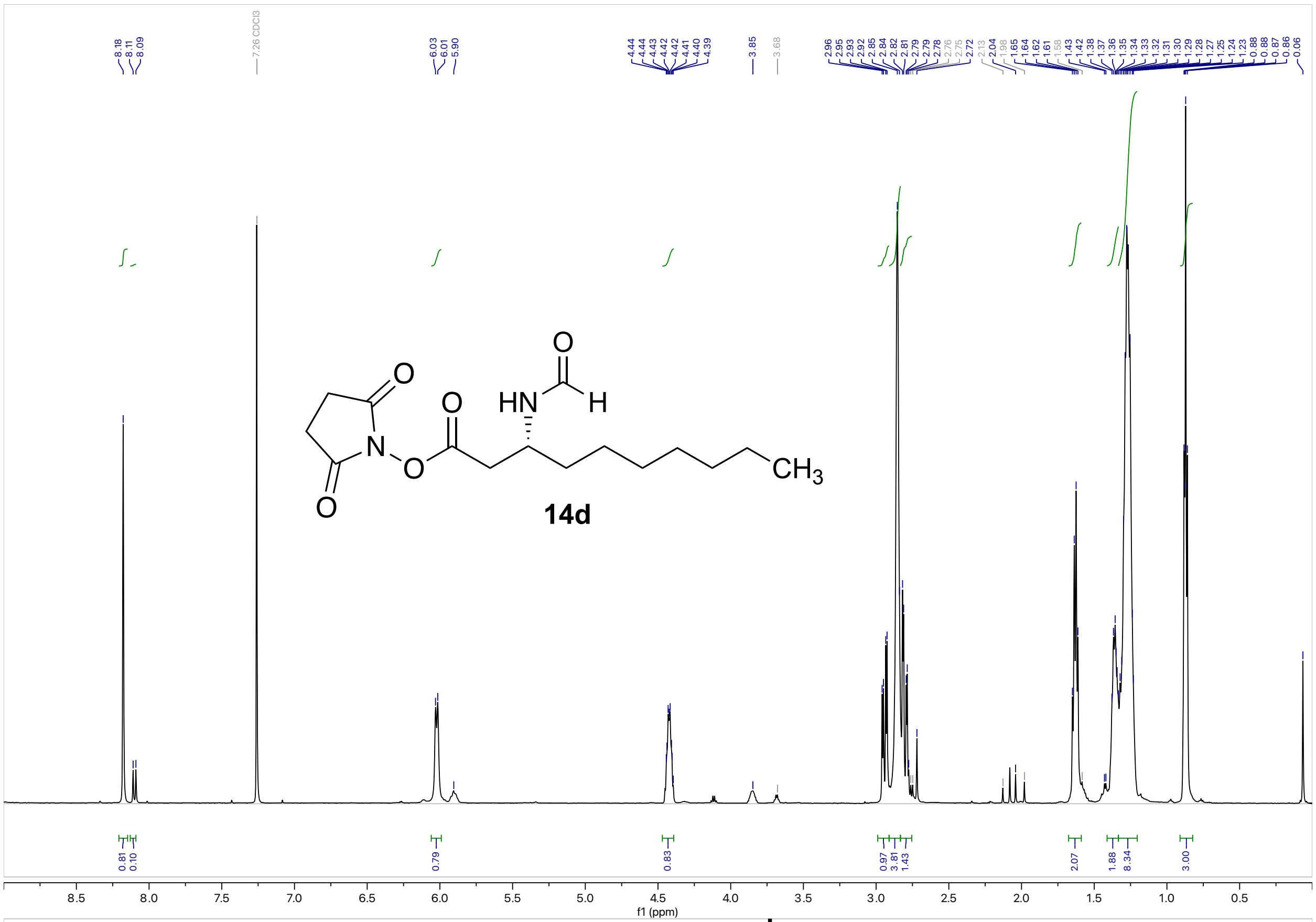
14d
f1 (ppm)

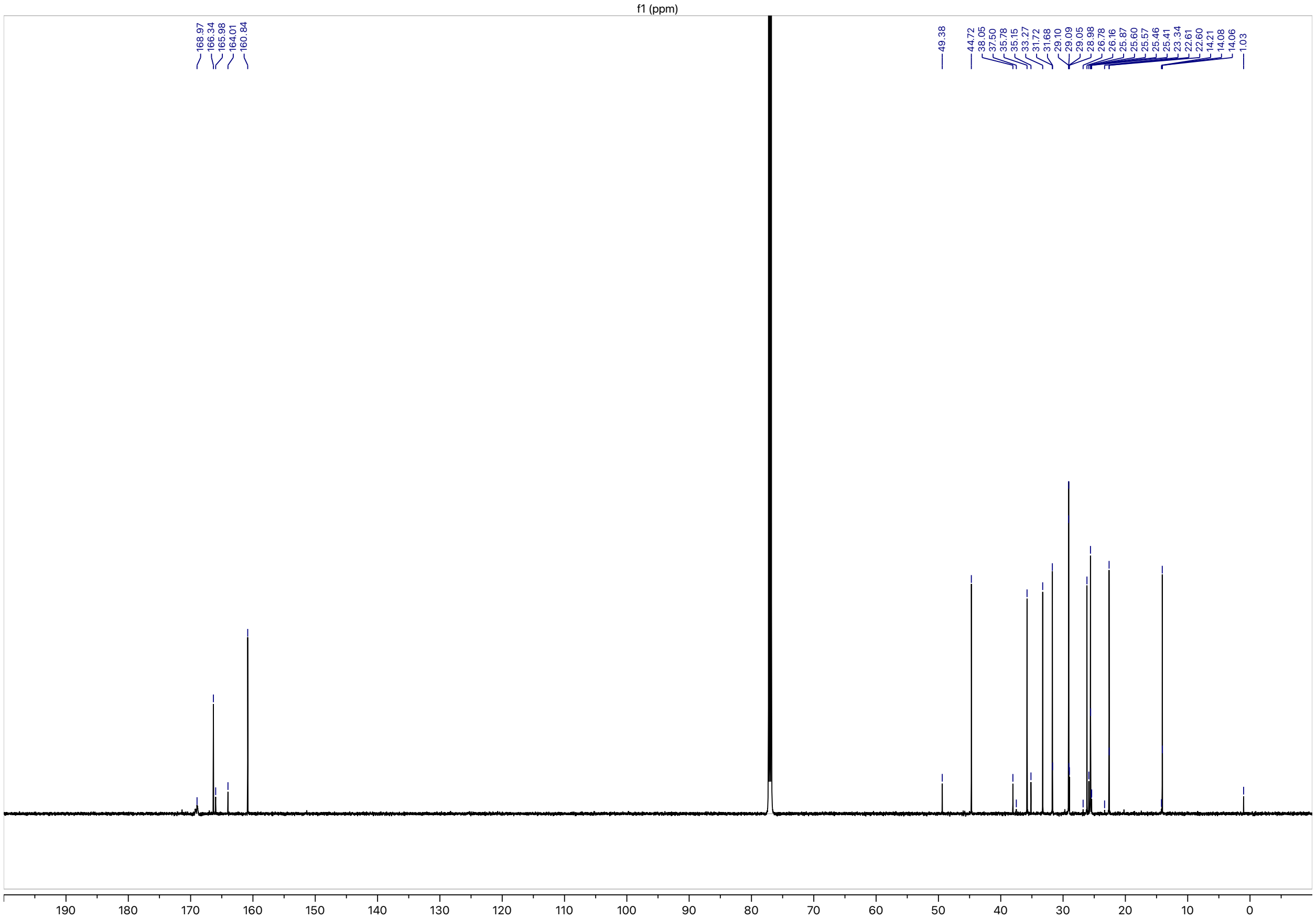
f1 (ppm)

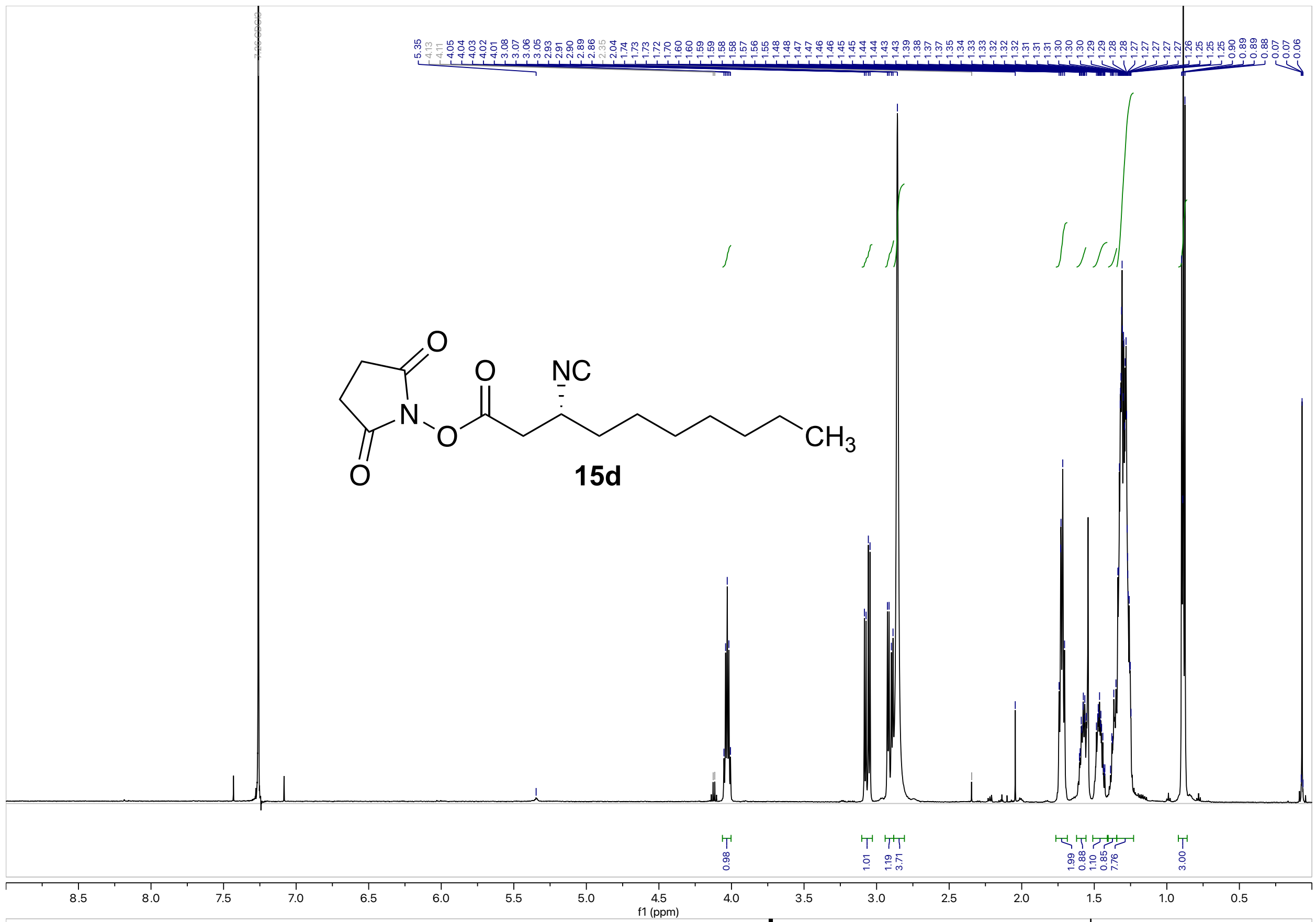
O
O
NC
N
O
CH3
15d
0.98
1.01
1.19
3.71
1.99
0.88
1.10
0.85
7.76
3.00
f1 (ppm)

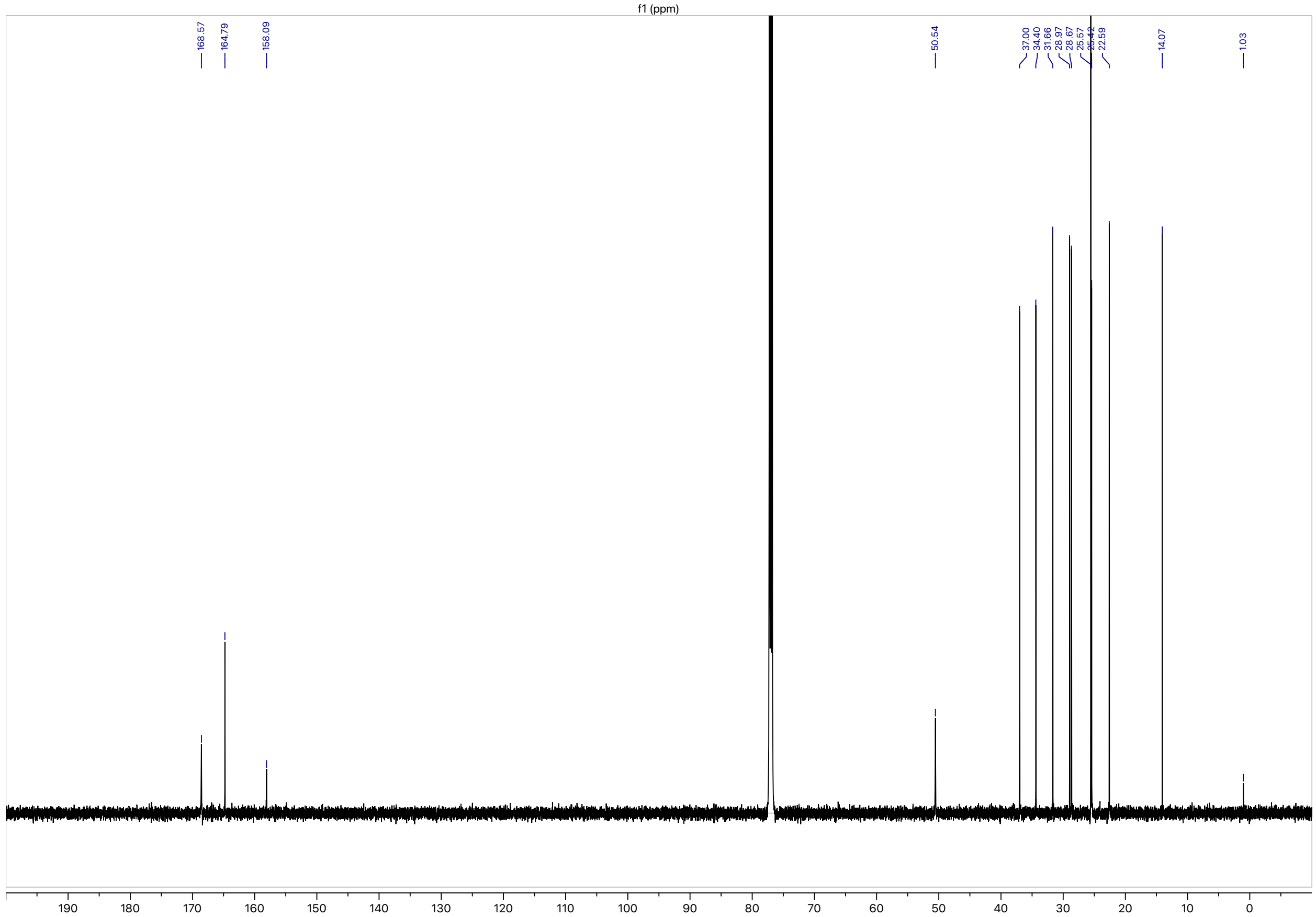
168.57
164.79
158.09
50.54
37.00
34.40
31.66
28.97
28.67
25.57
25.42
22.59
14.07
1.03
f1 (ppm)

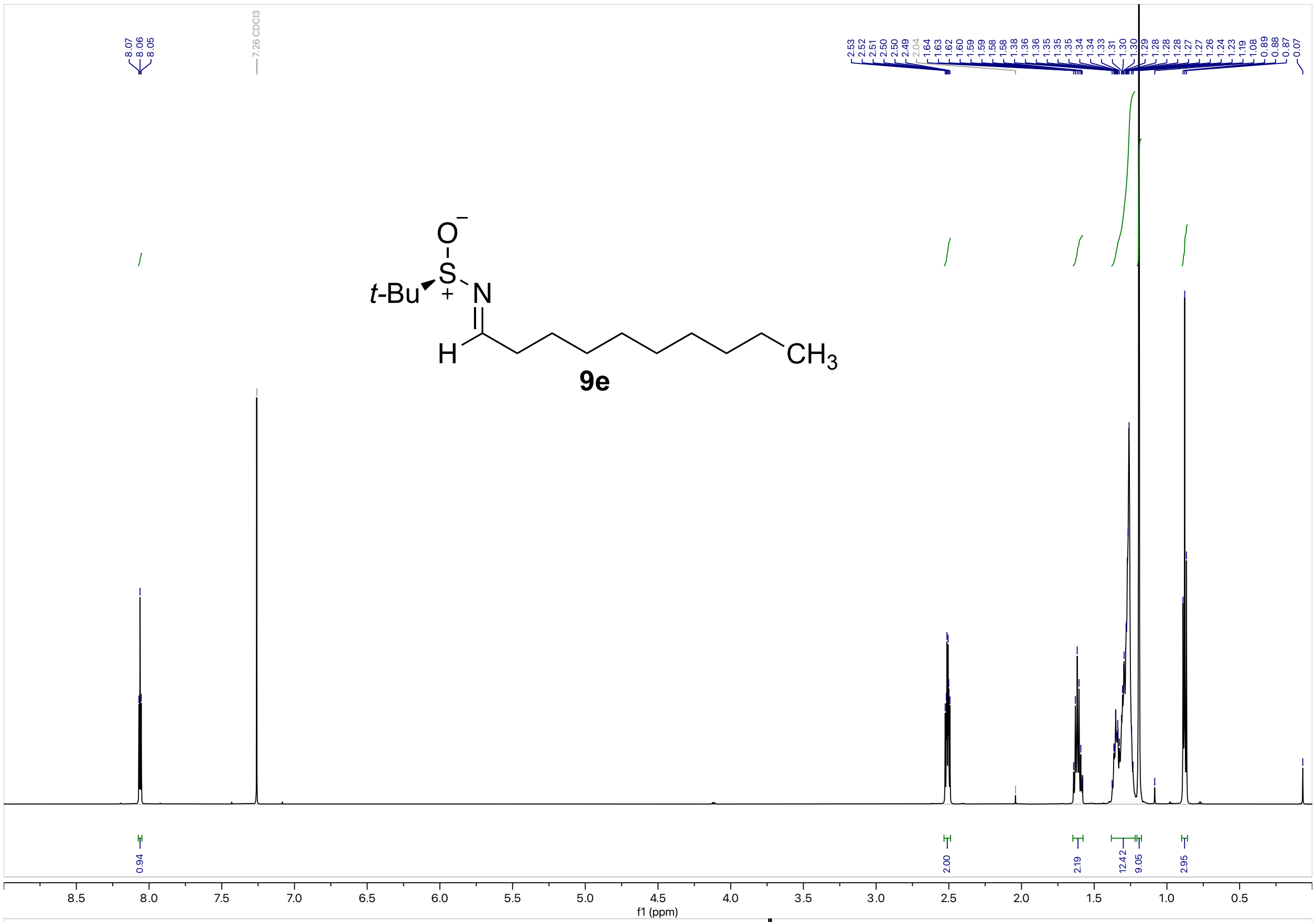

9e
t-Bu
CH3
f1 (ppm)

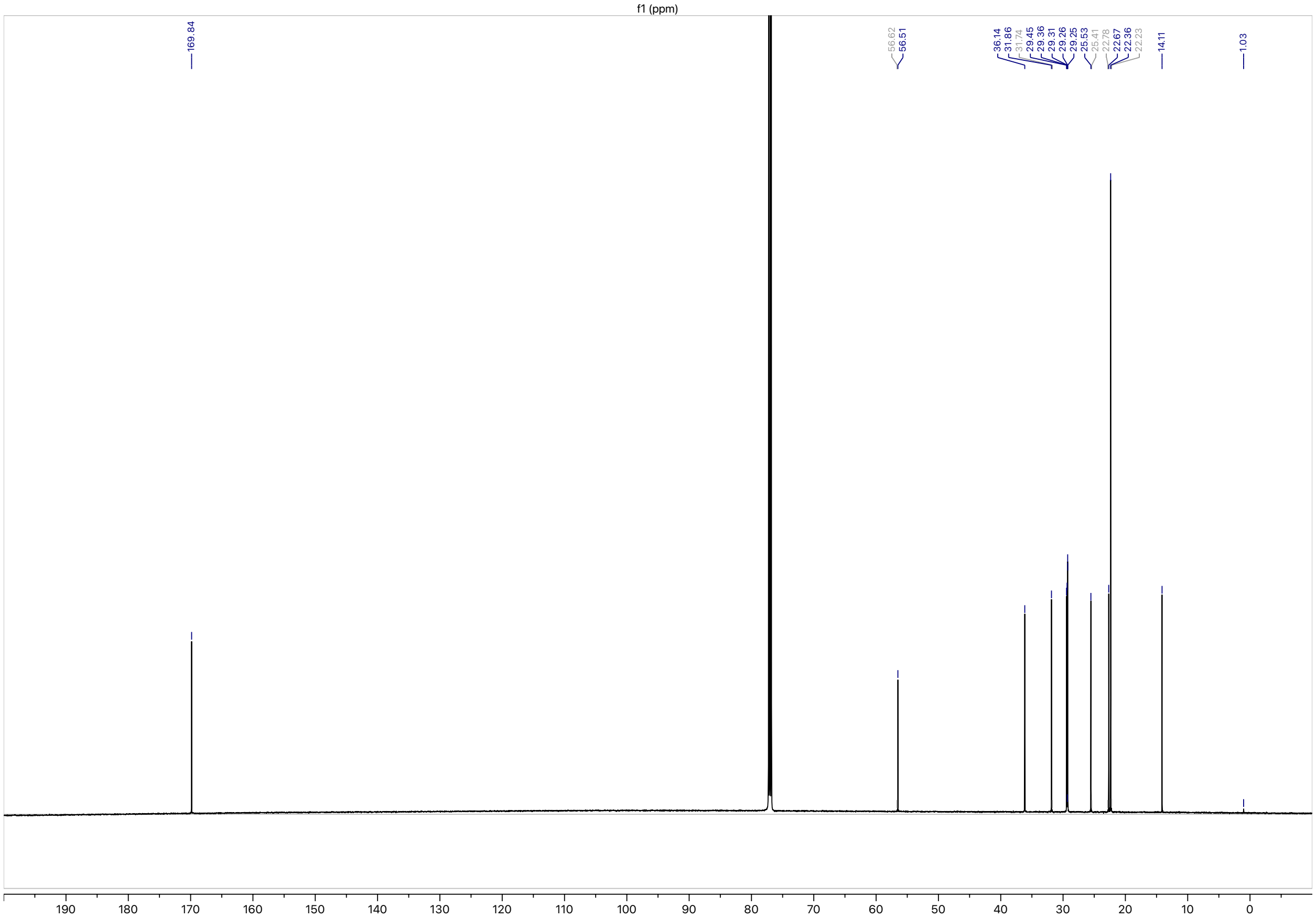

f1 (ppm)

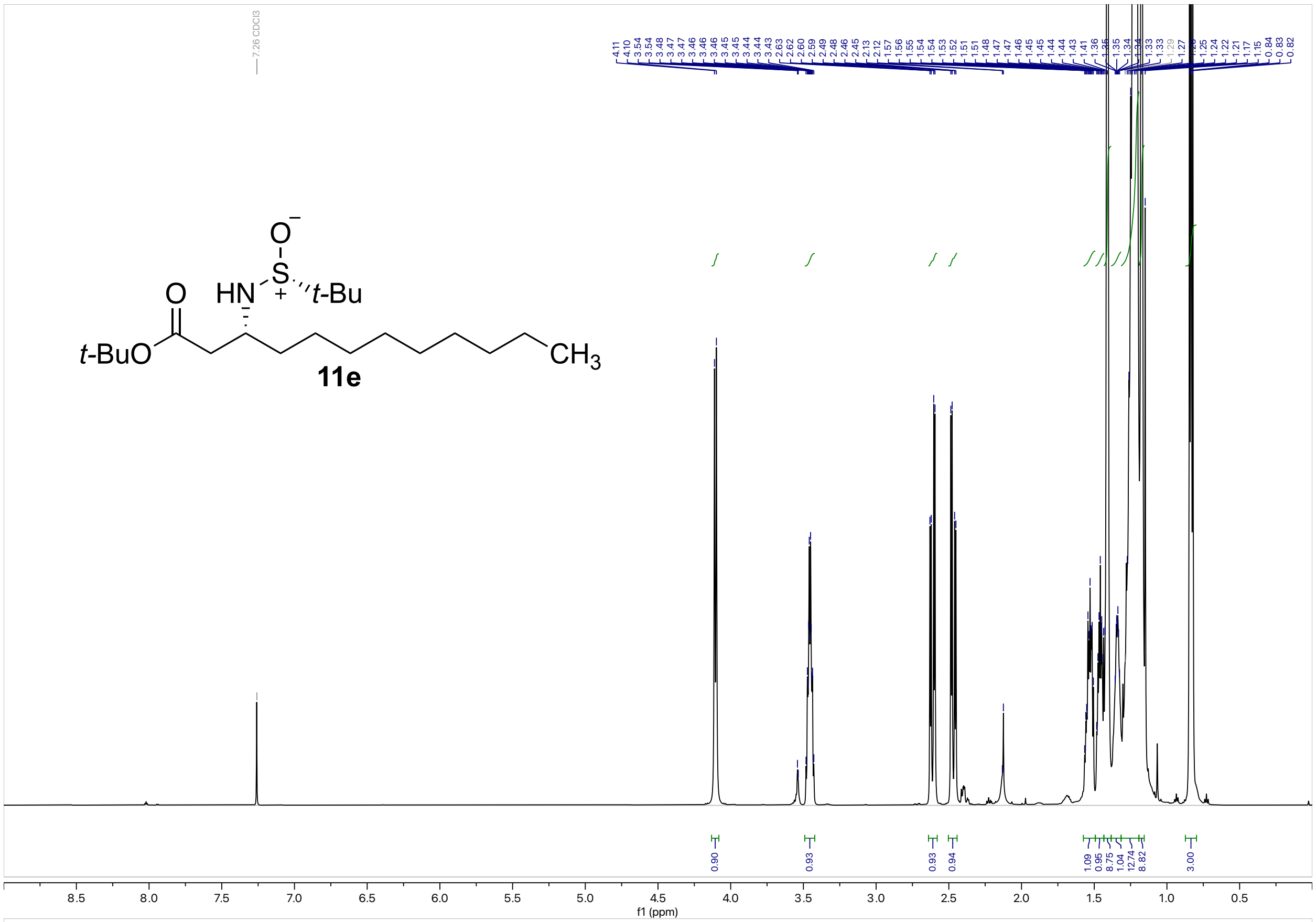
O
HN
S
t-Bu
t-BuO
CH3
11e
f1 (ppm)

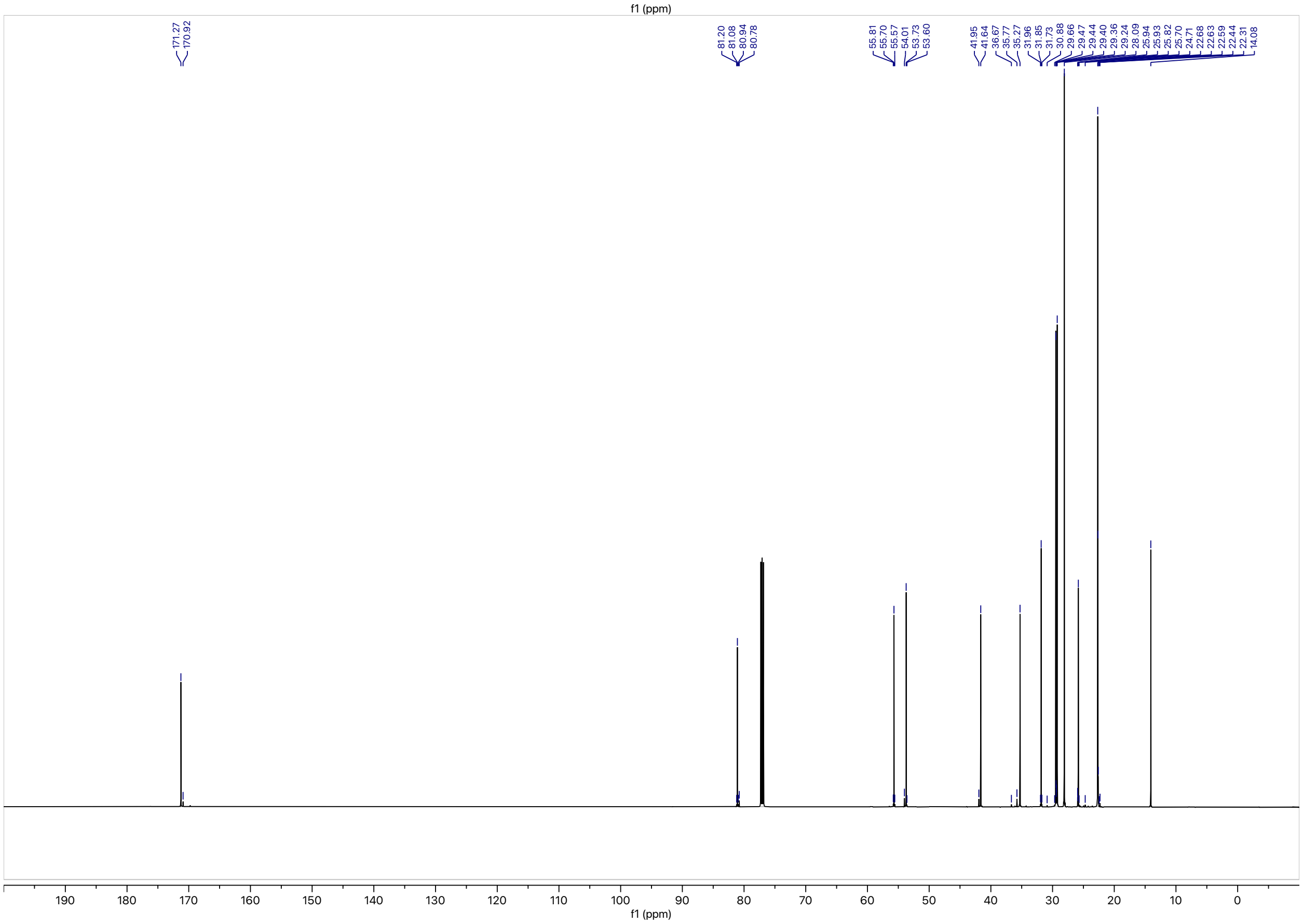
f1 (ppm)

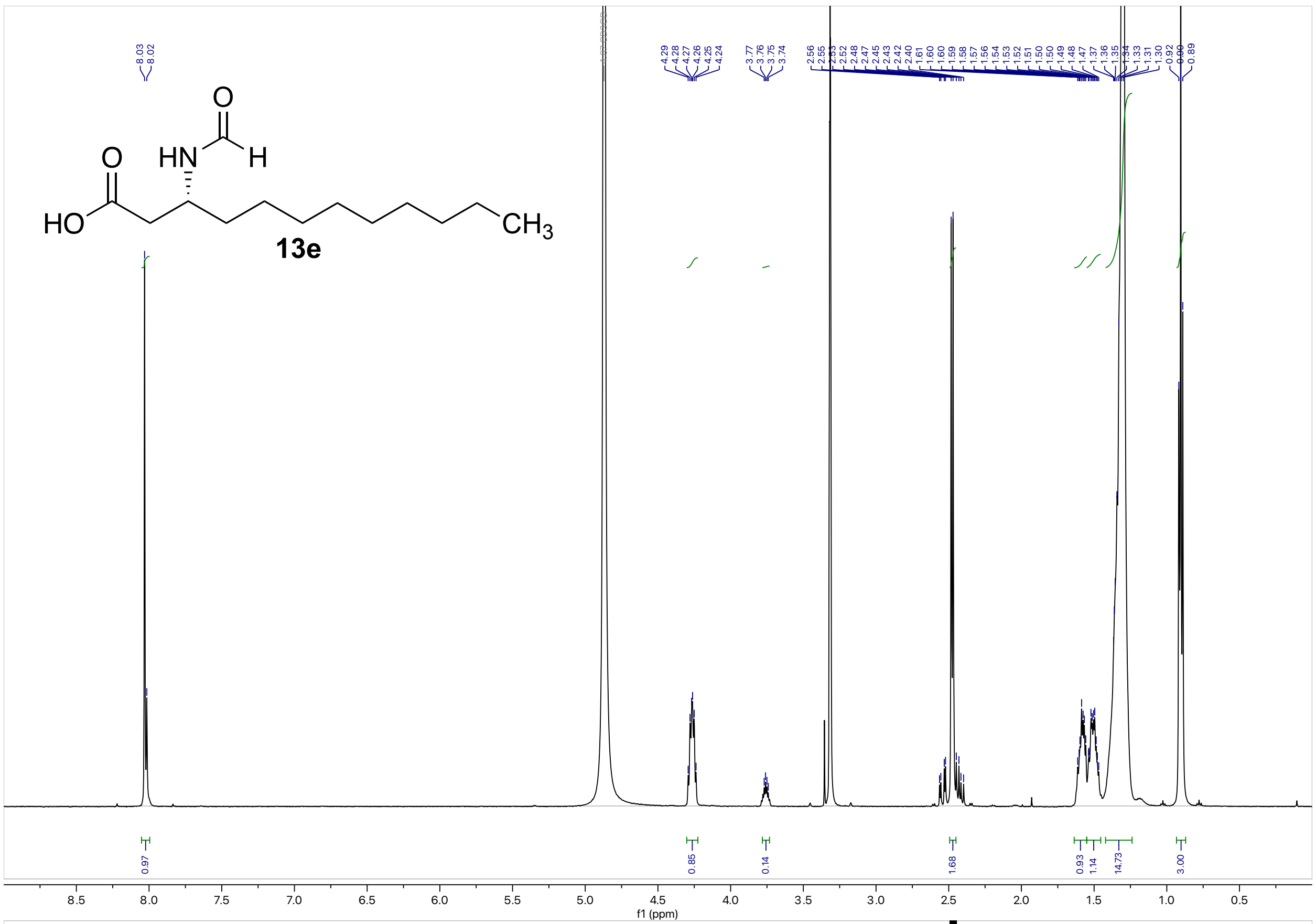
13e
f1 (ppm)

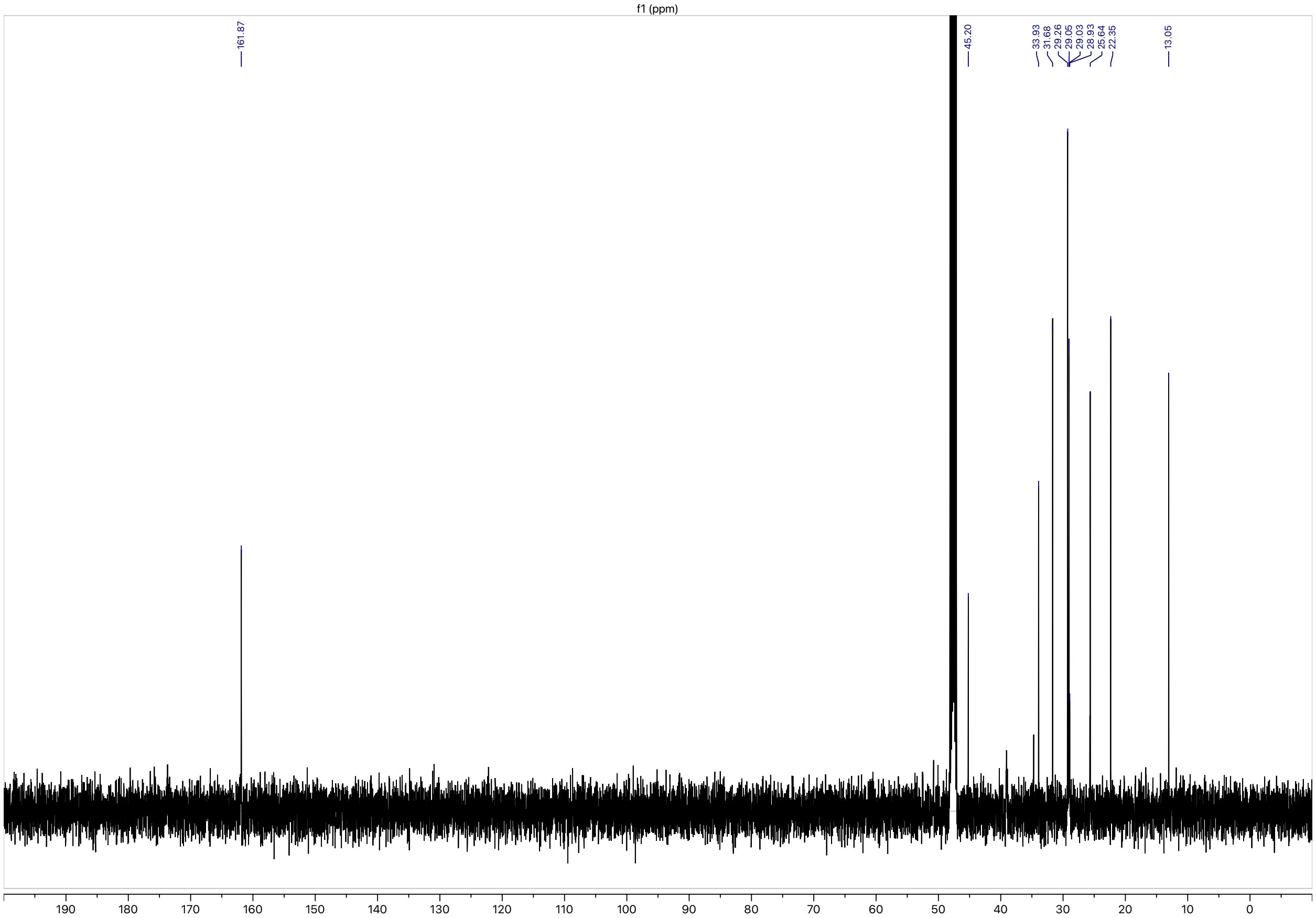
f1 (ppm)

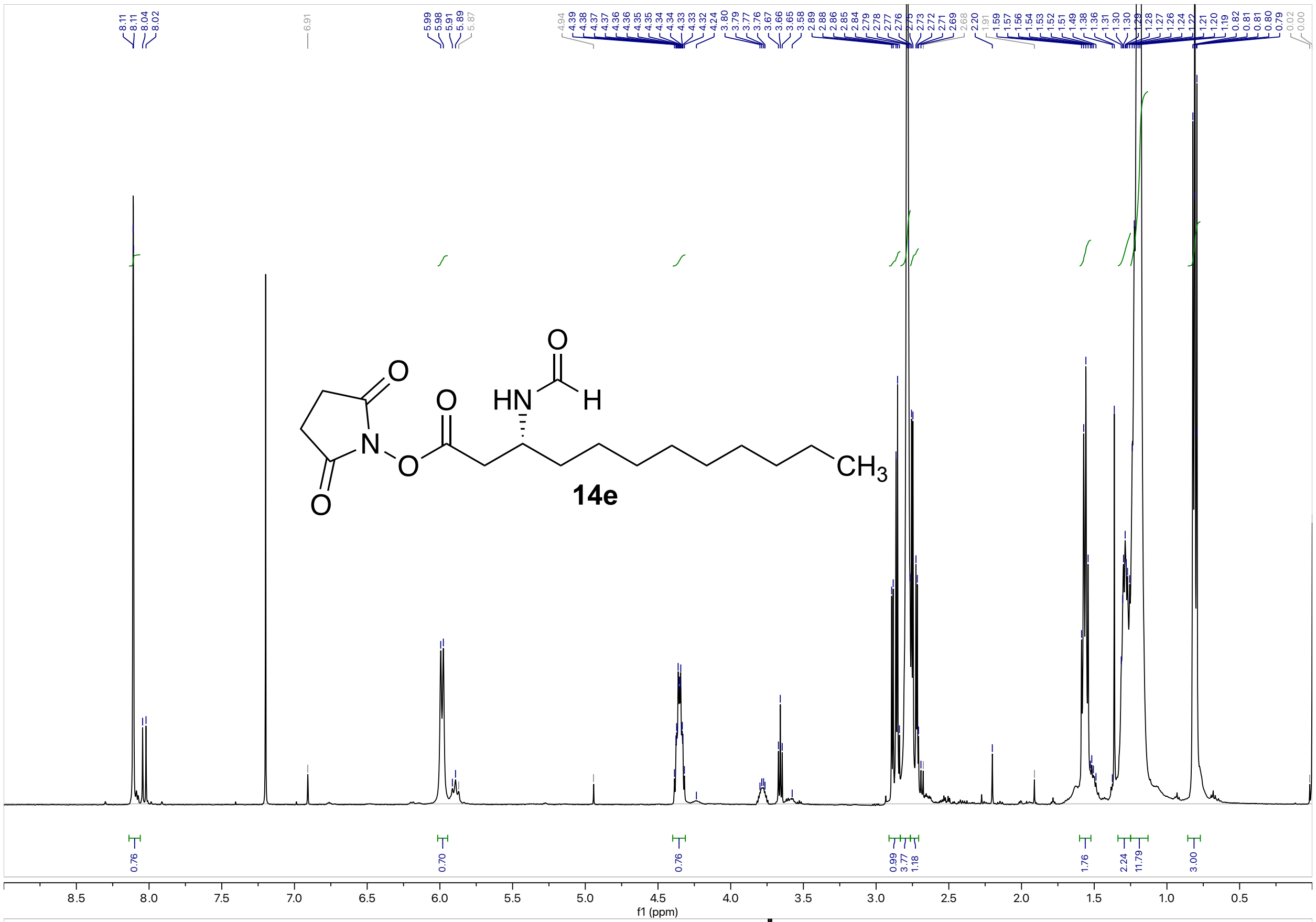
HN
O
H
O
O
N
O
O
CH3
14e
f1 (ppm)

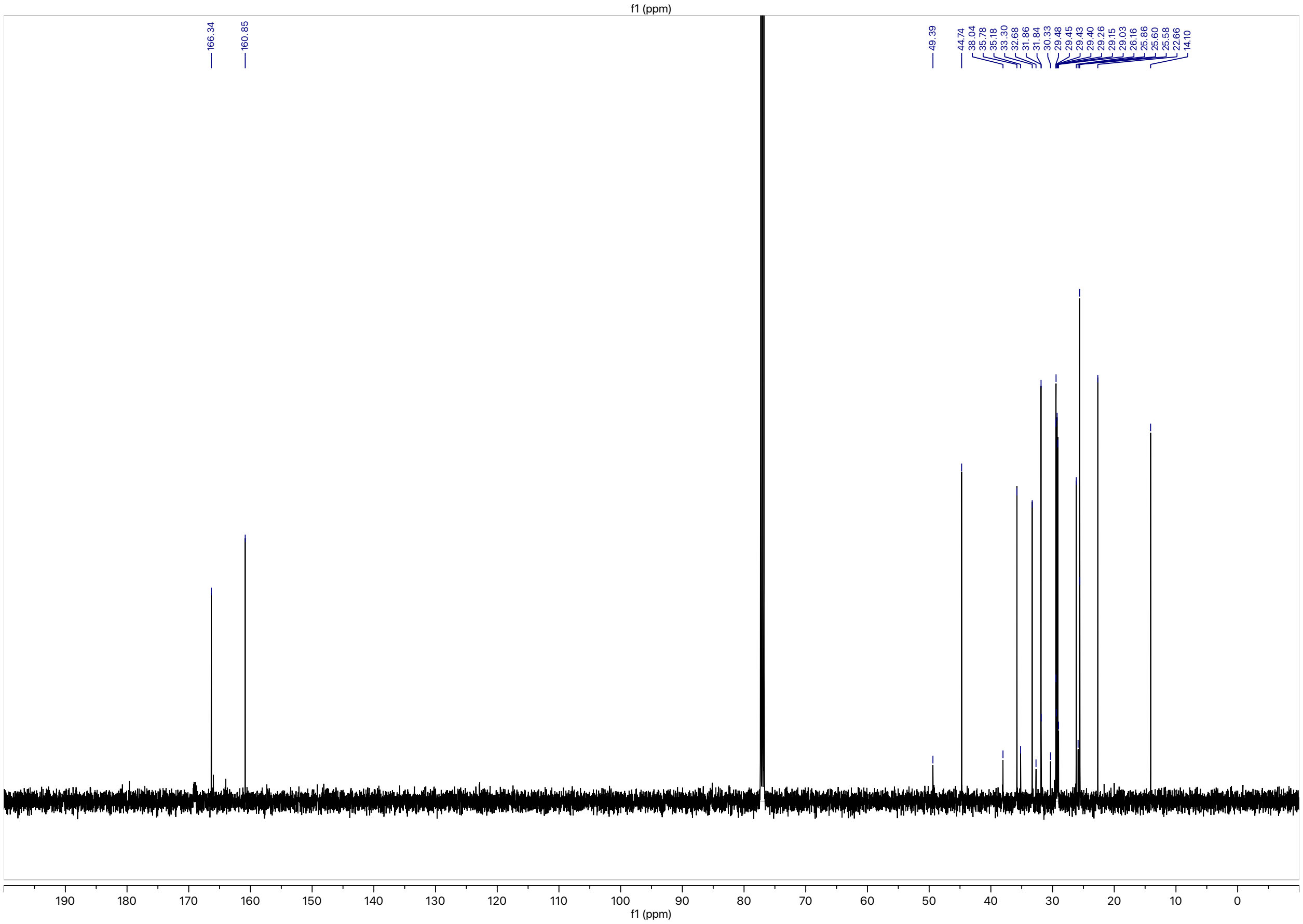
f1 (ppm)

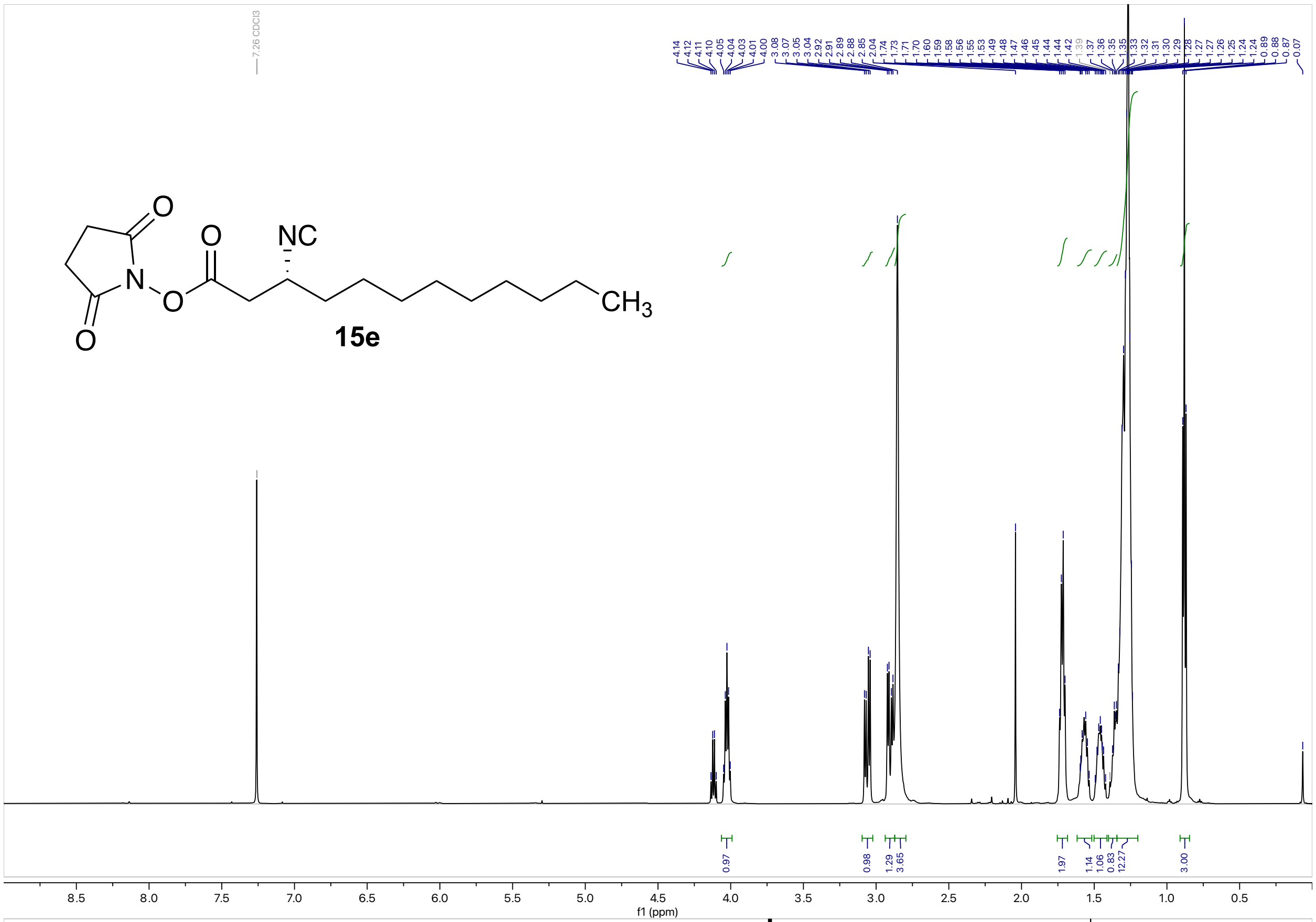

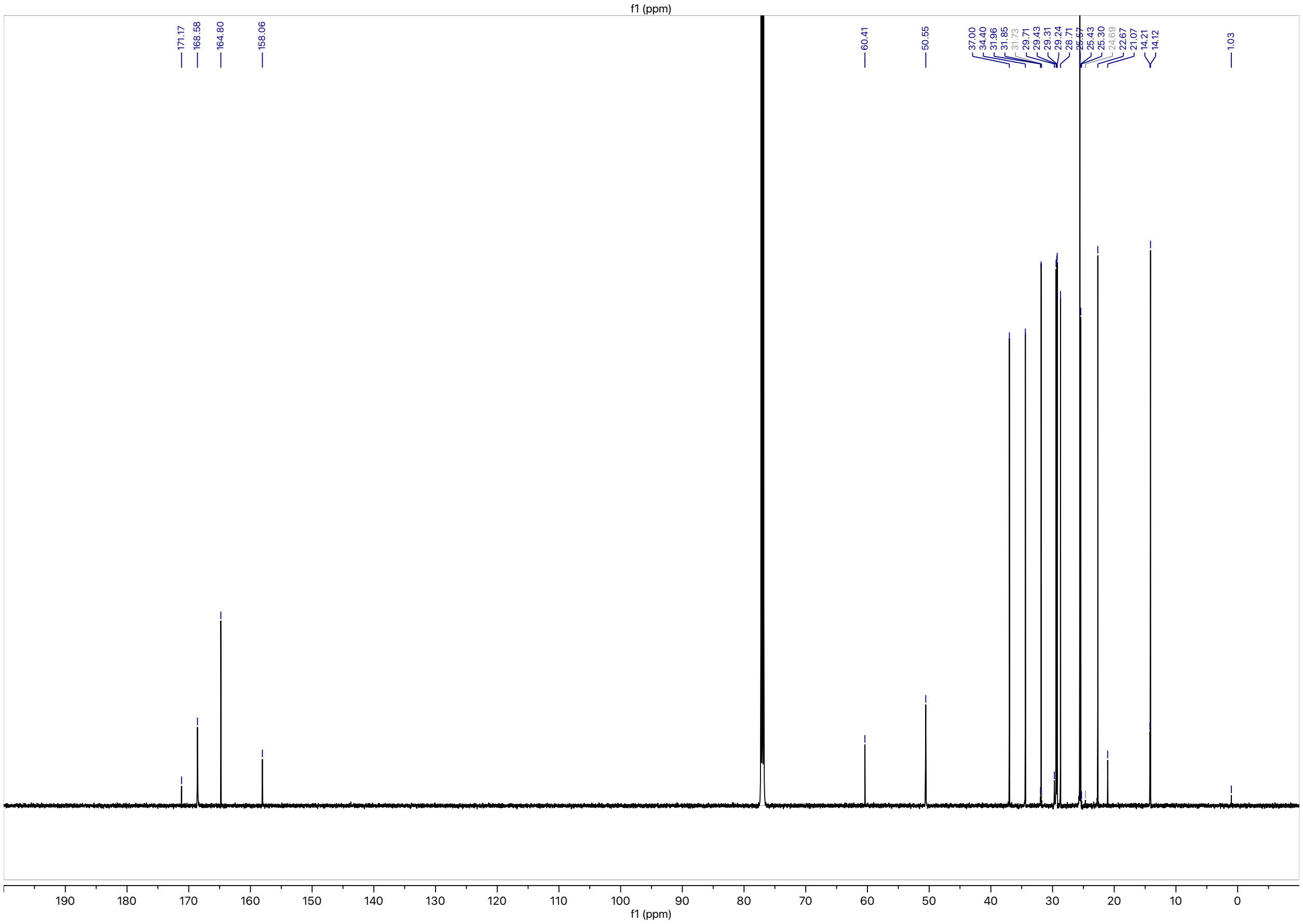

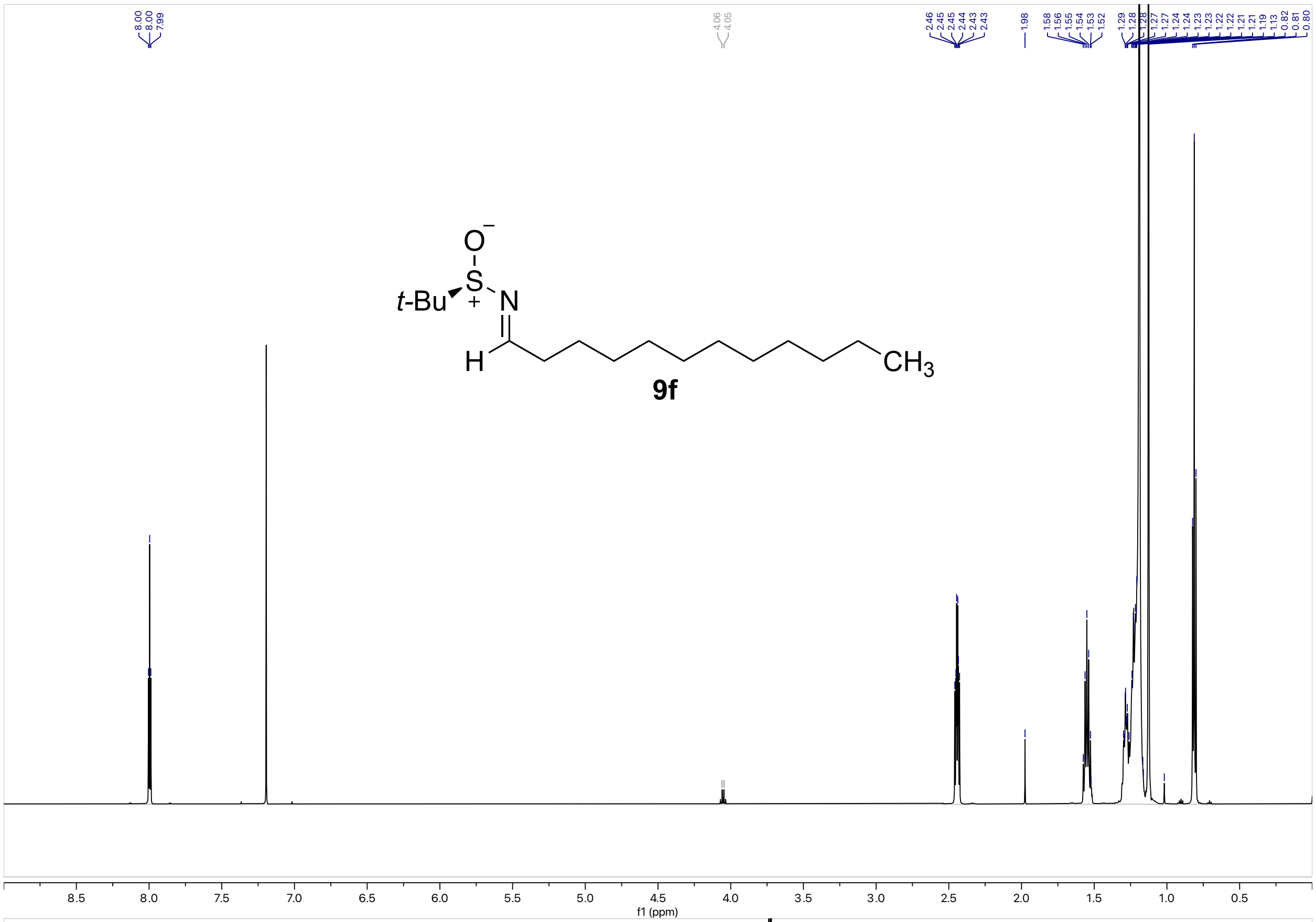
t-Bu
S
O
N
H
CH3
9f
f1 (ppm)

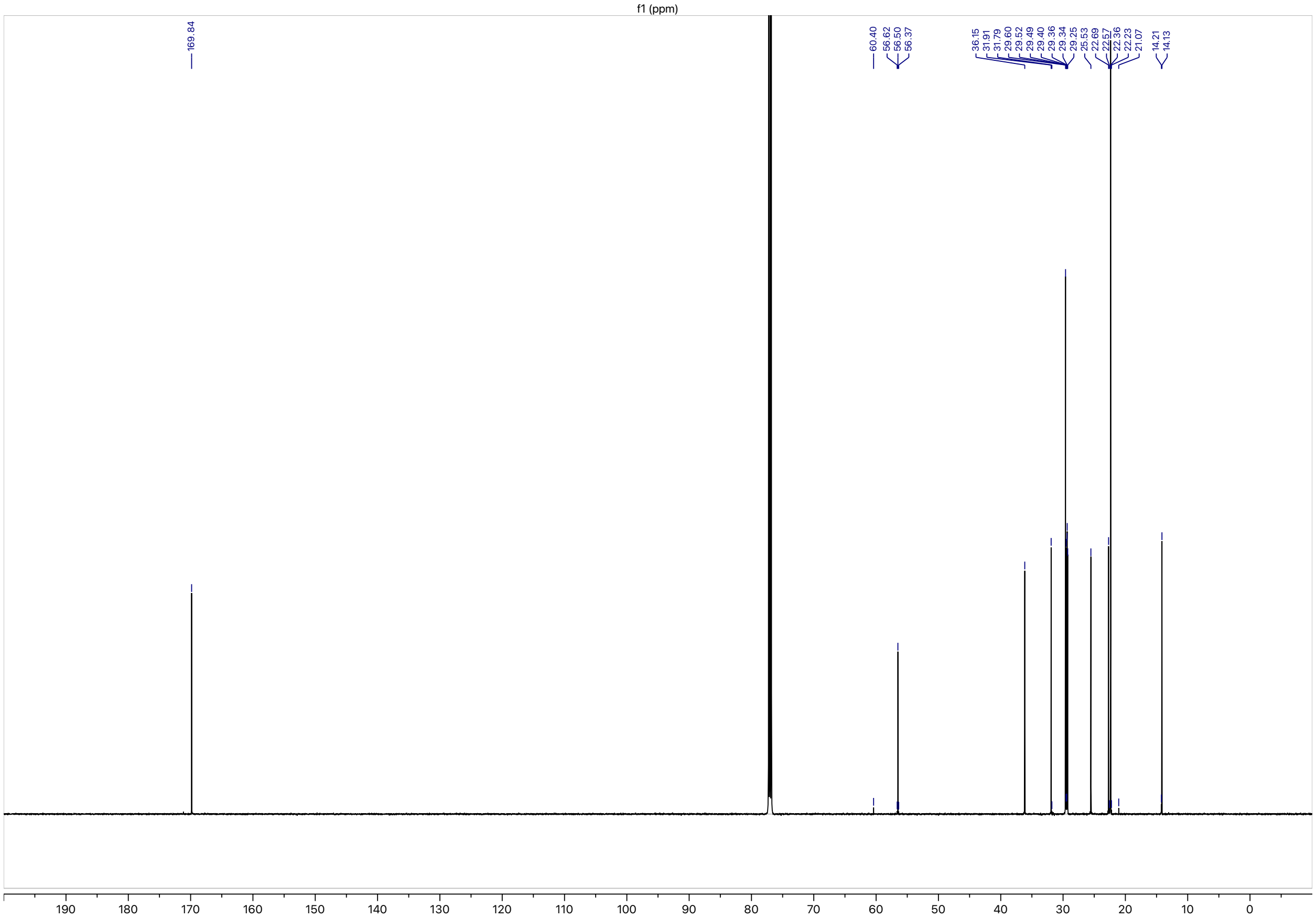
f1 (ppm)

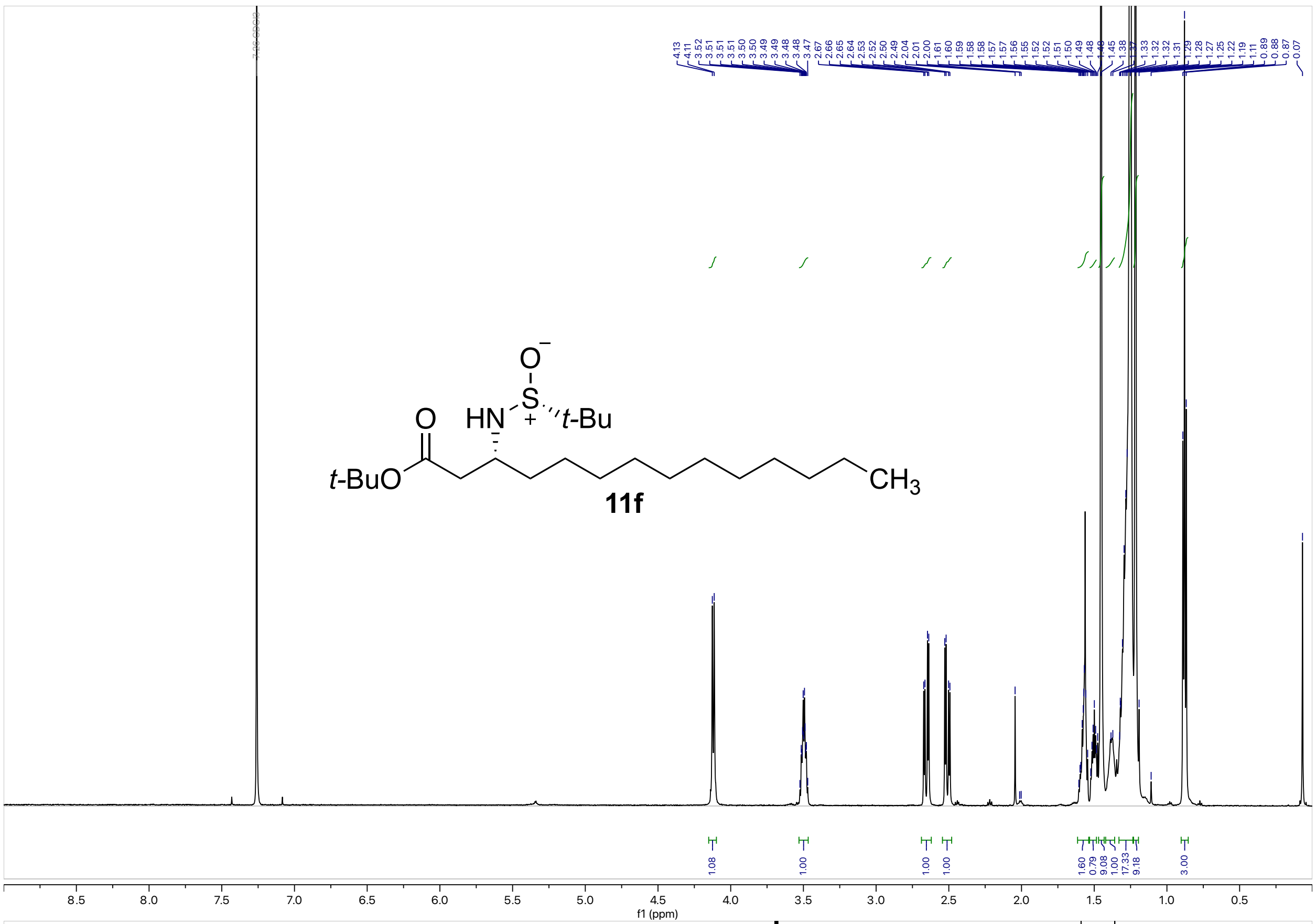
O
HN
S
t-Bu
t-BuO
CH3
11f
f1 (ppm)

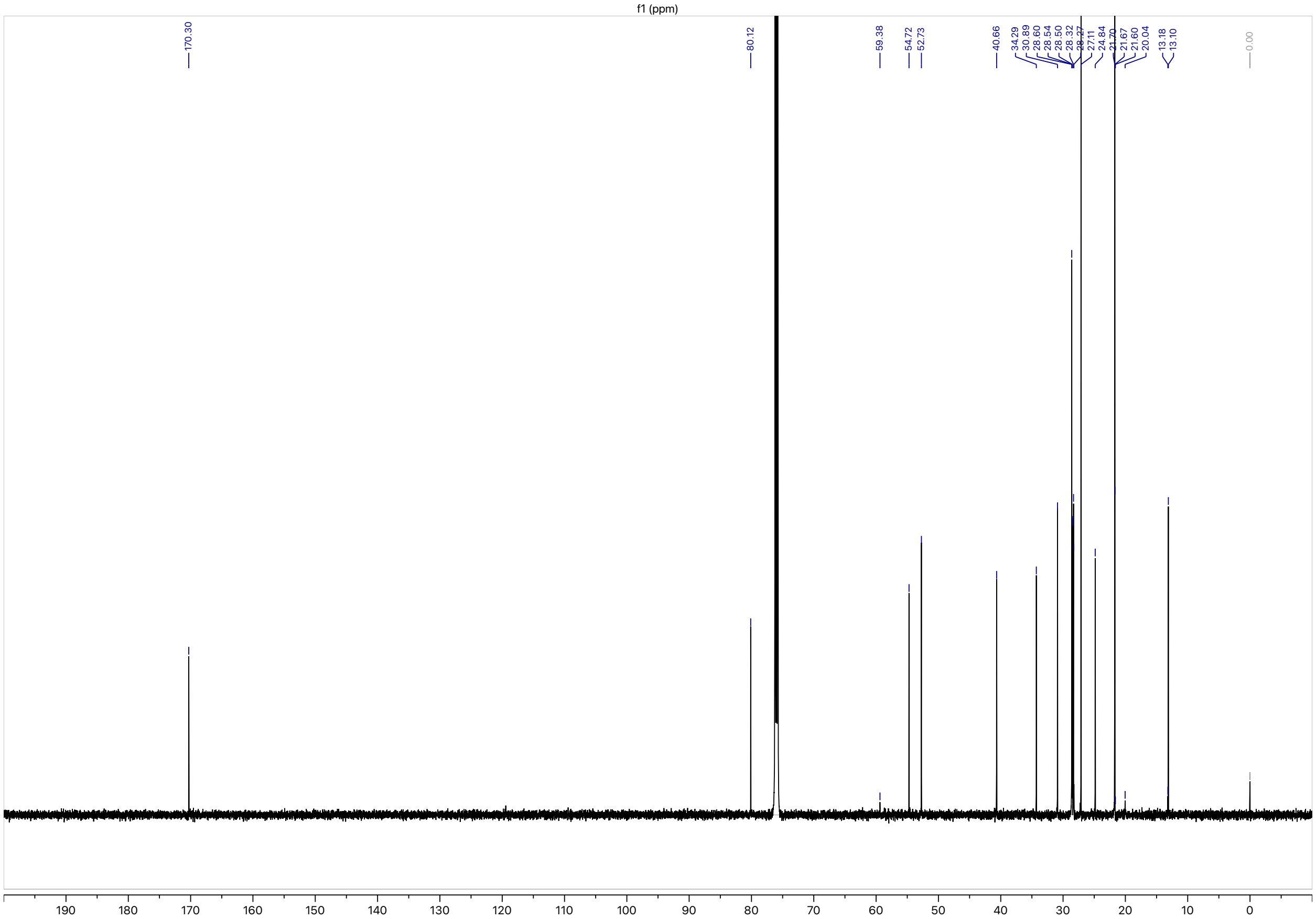
f1 (ppm)

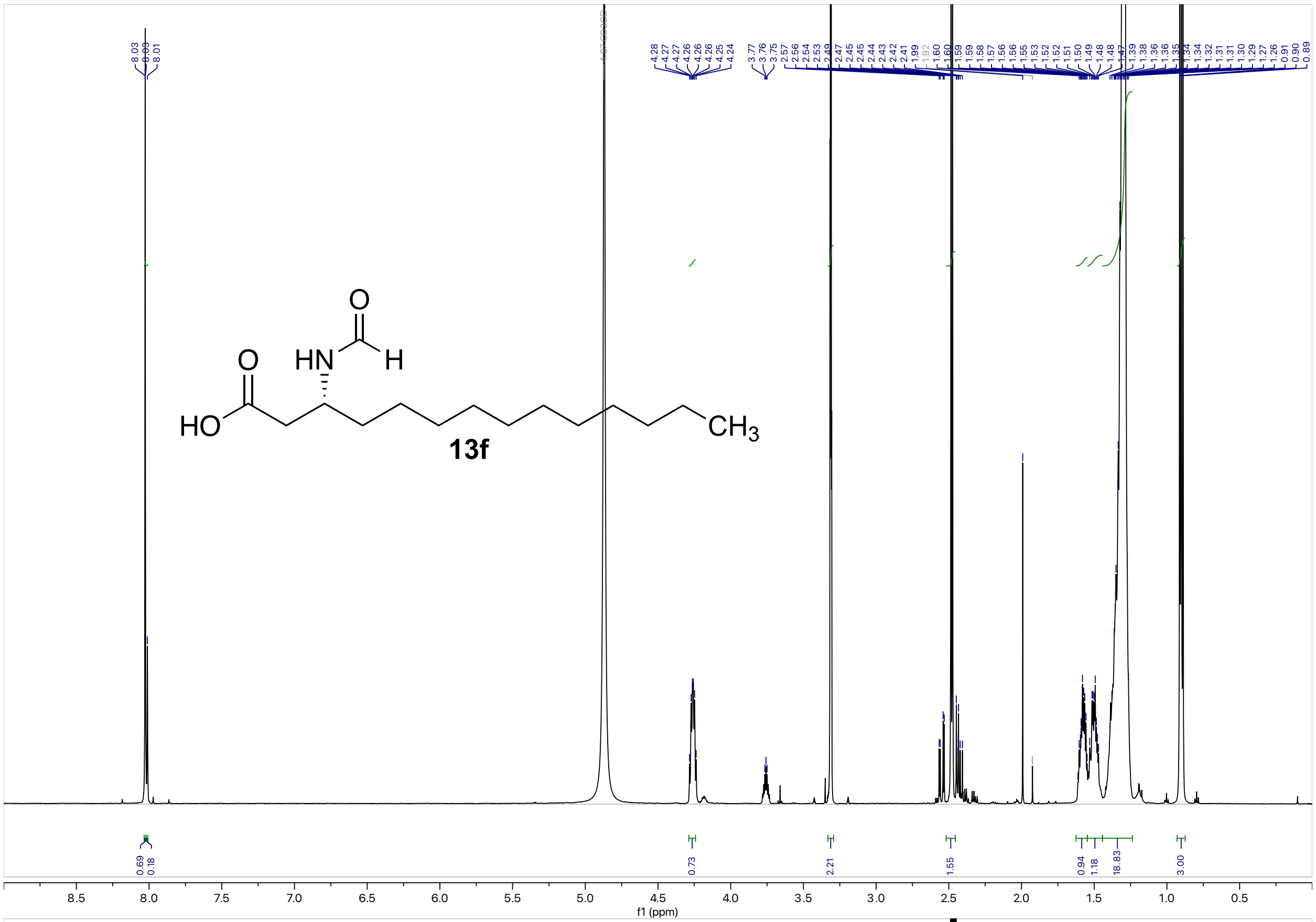
13f
f1 (ppm)

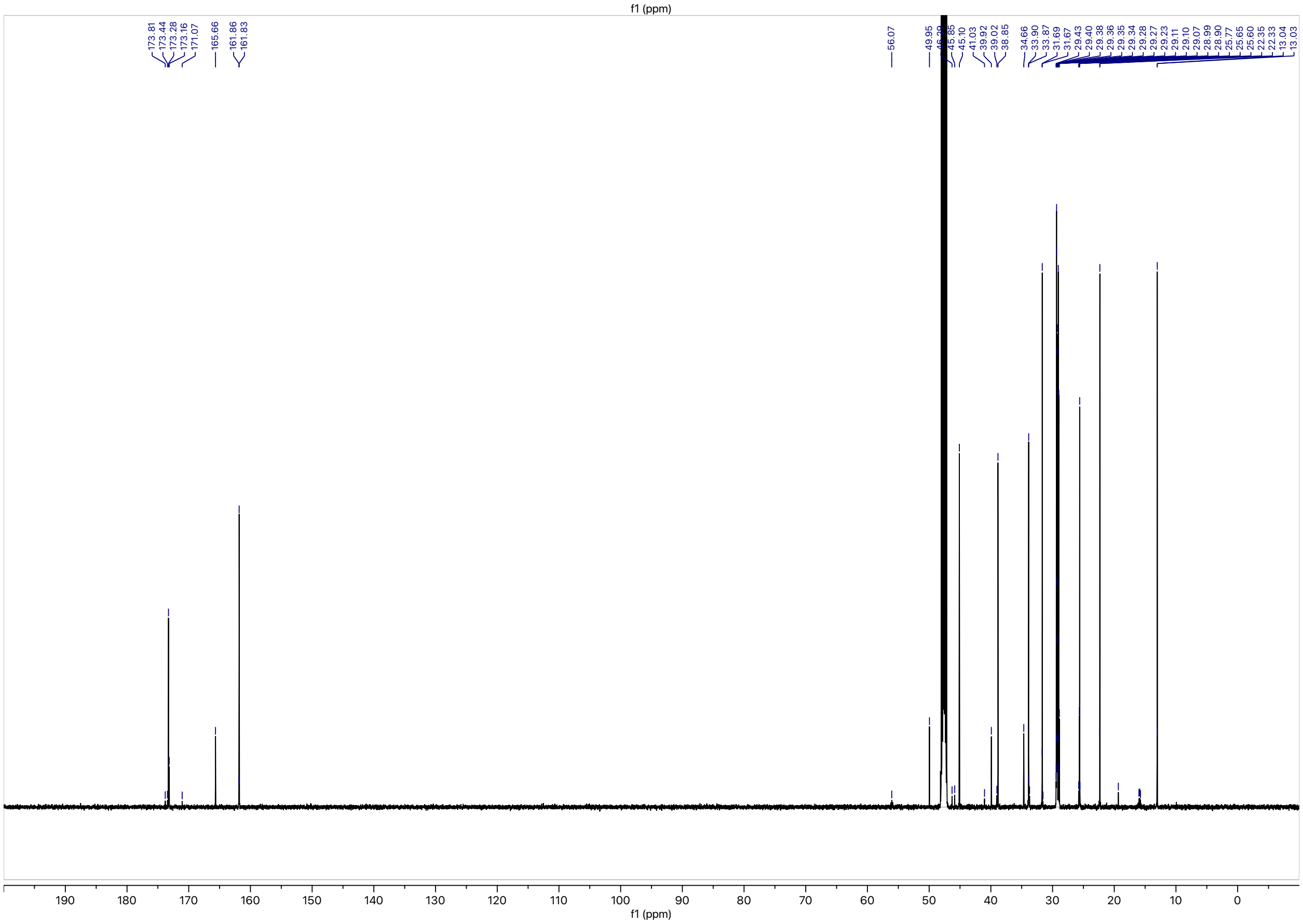
f1 (ppm)

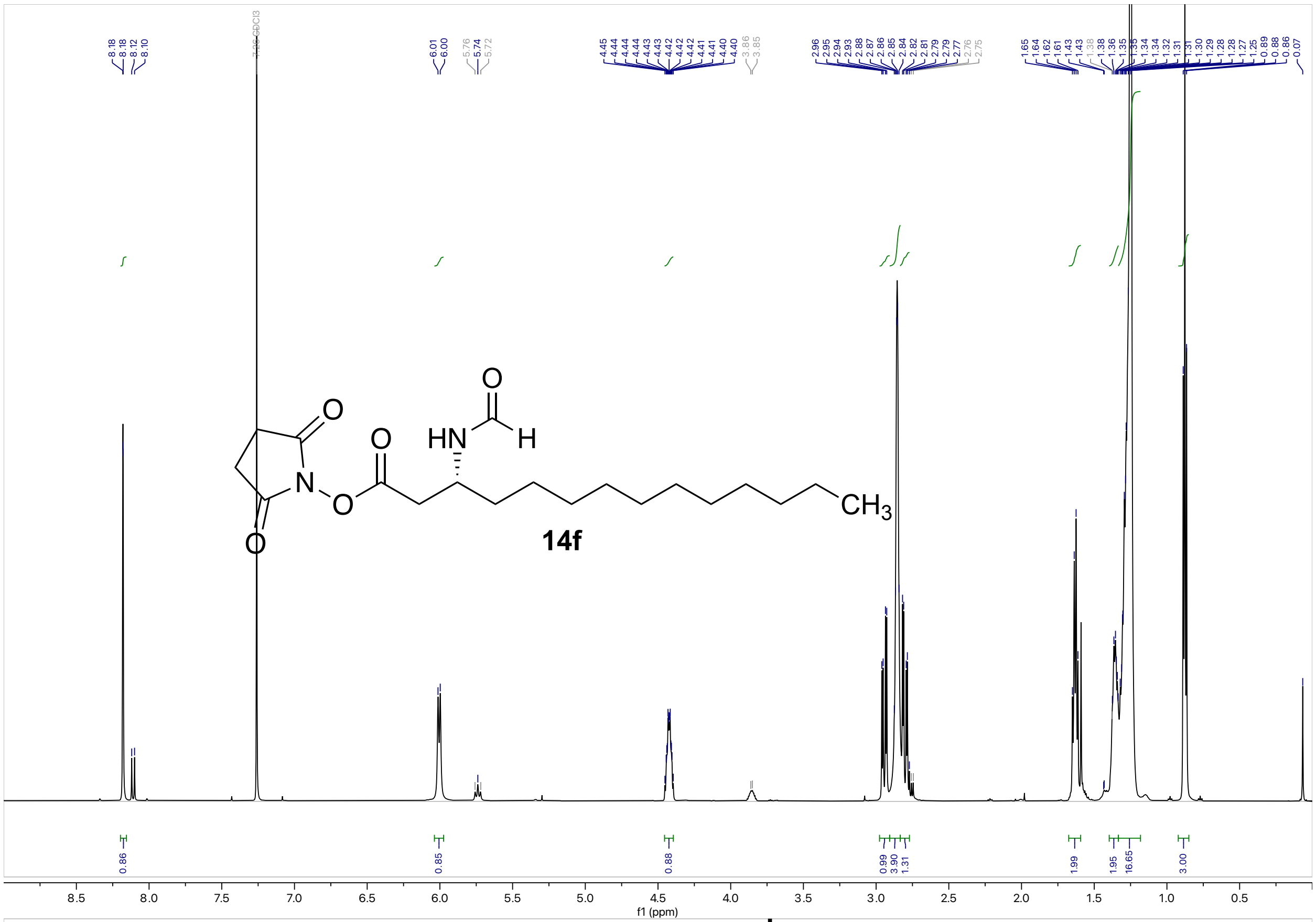
14f
CH3
HN
f1 (ppm)

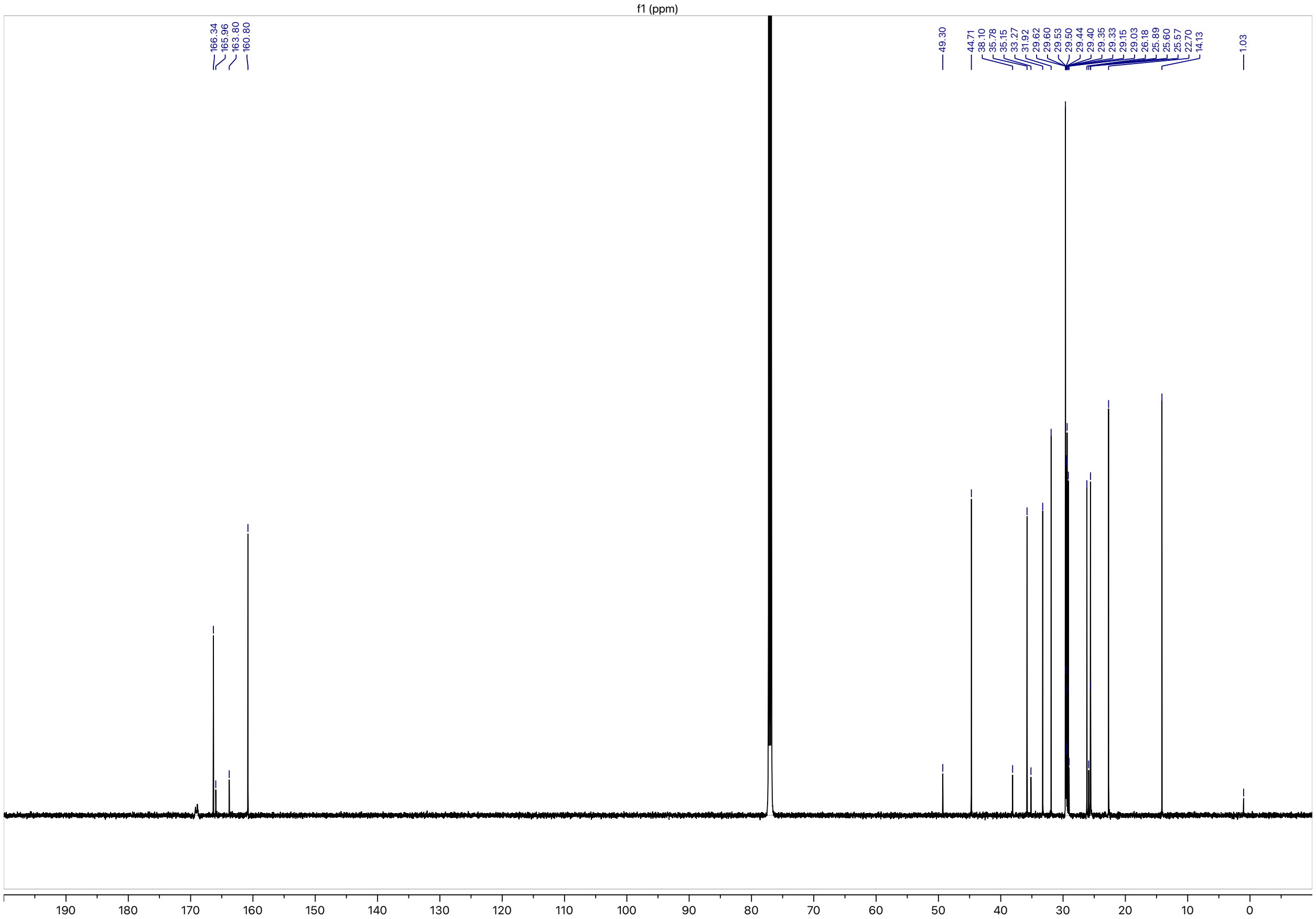
f1 (ppm)

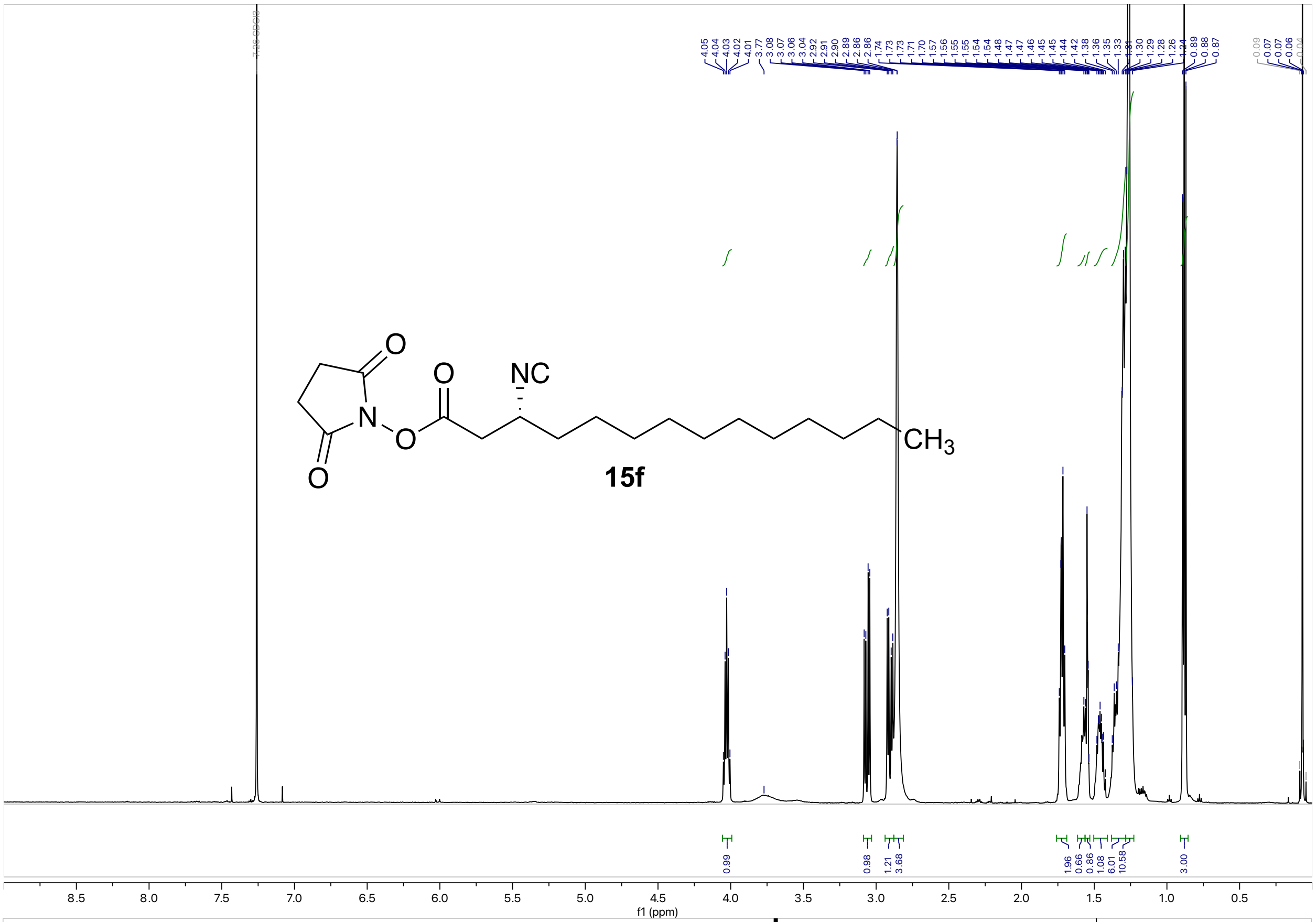
O
O
NC
N
O
CH3
O
15f
f1 (ppm)

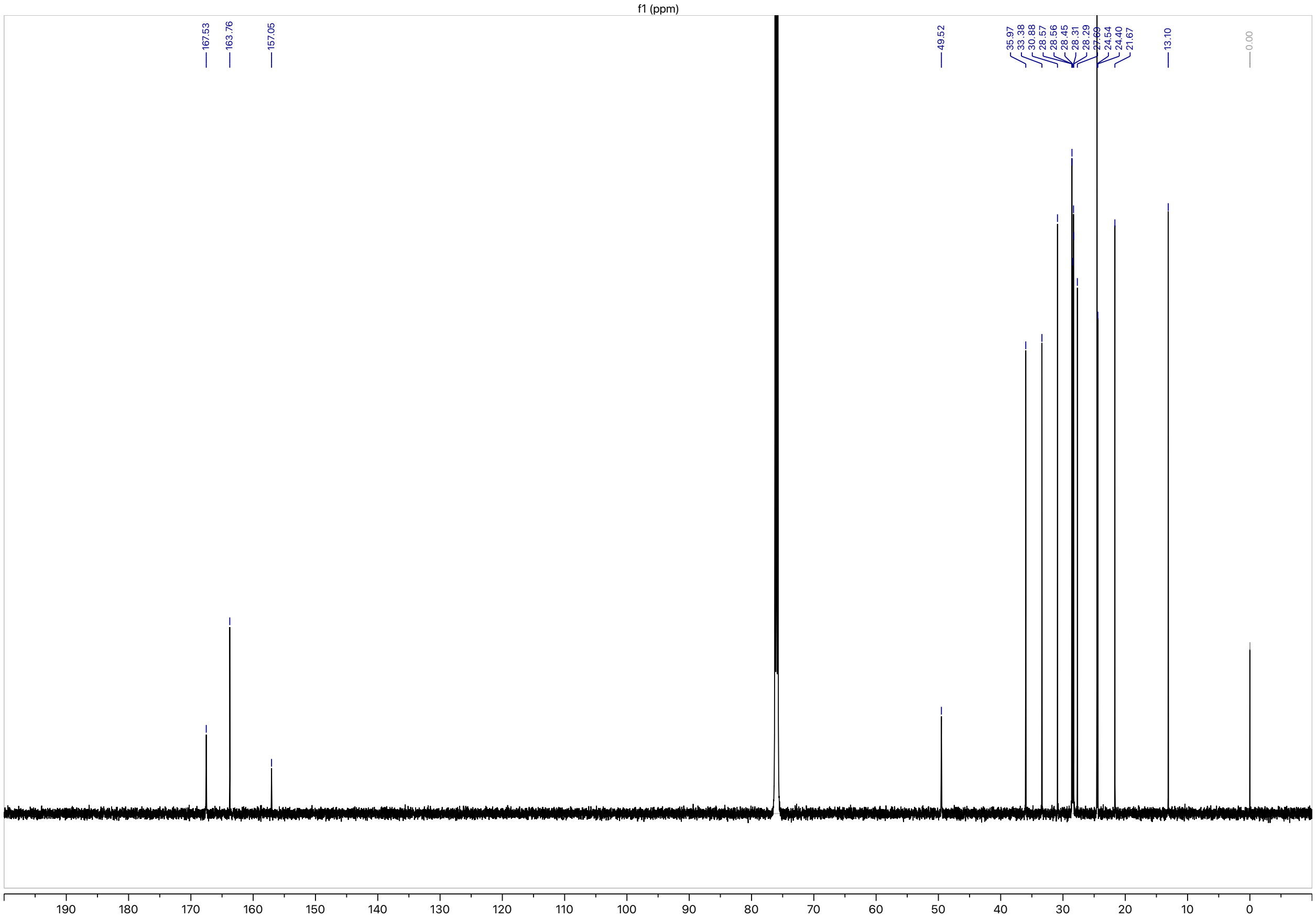
f1 (ppm)

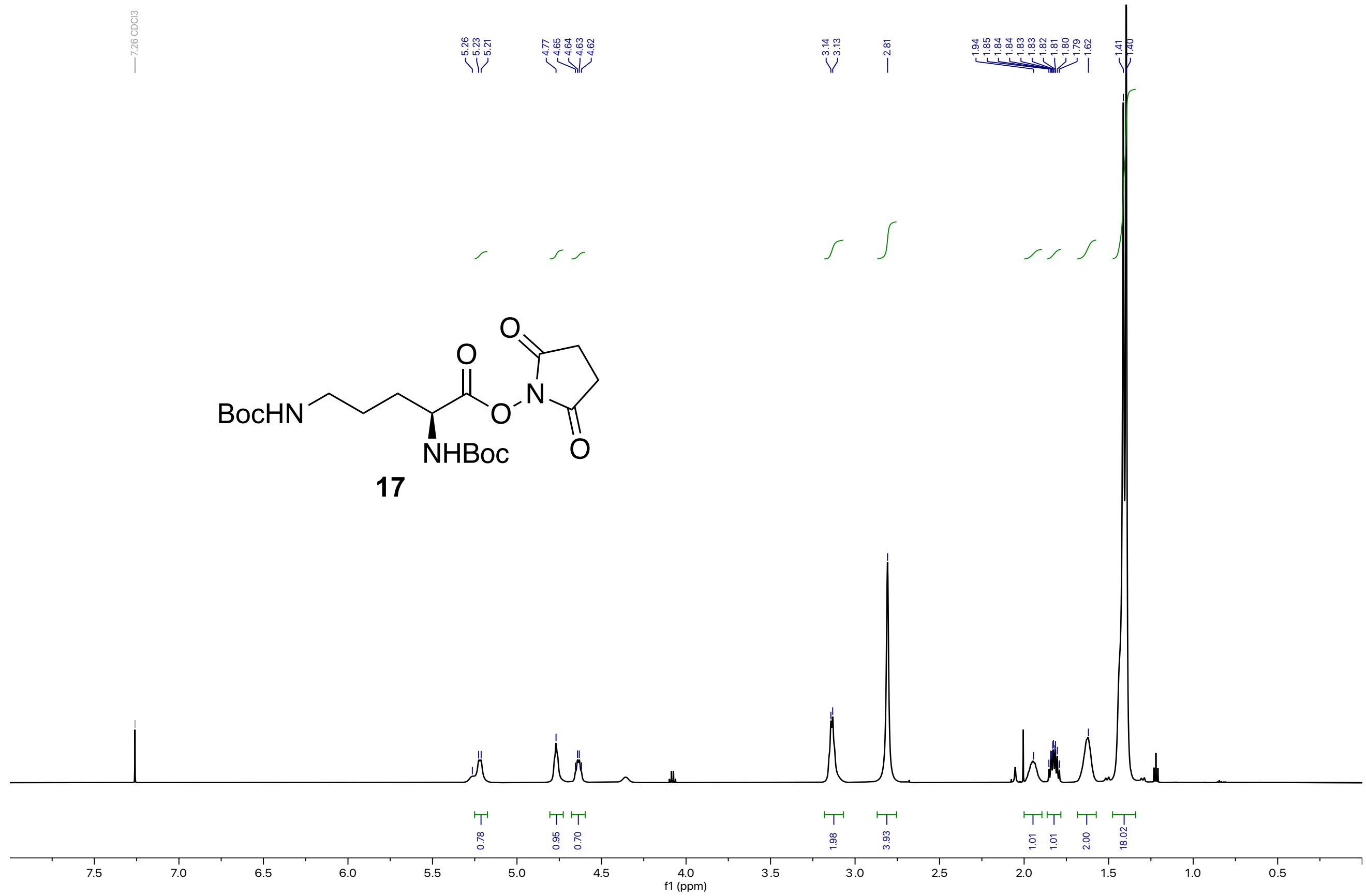
7.26 CDCl3
5.26
5.23
5.21
4.77
4.65
4.64
4.63
4.62
3.14
3.13
2.81
1.94
1.85
1.84
1.84
1.83
1.83
1.82
1.81
1.80
1.79
1.62
1.41
1.40
BocHN
O
O
N
O
O
NHBoc
O
17
0.78
0.95
0.70
1.98
3.93
1.01
1.01
2.00
18.02
7.5 7.0 6.5 6.0 5.5 5.0 4.5 4.0 3.5 3.0 2.5 2.0 1.5 1.0 0.5
f1 (ppm)

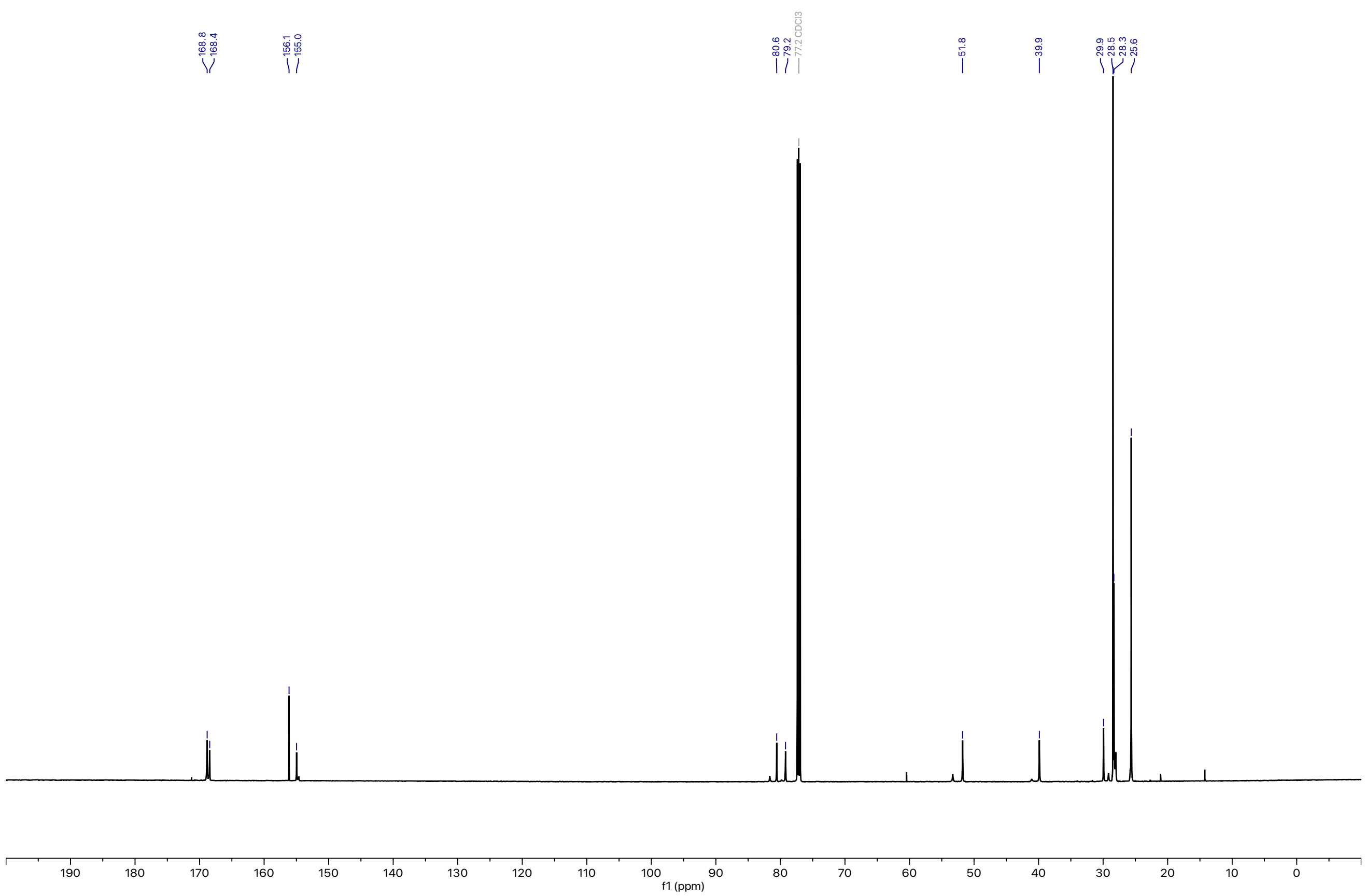
168.8
168.4
156.1
155.0
80.6
79.2
77.2 CDCl3
51.8
39.9
29.9
28.5
28.3
25.6
190 180 170 160 150 140 130 120 110 100 90 80 70 60 50 40 30 20 10 0
f1 (ppm)

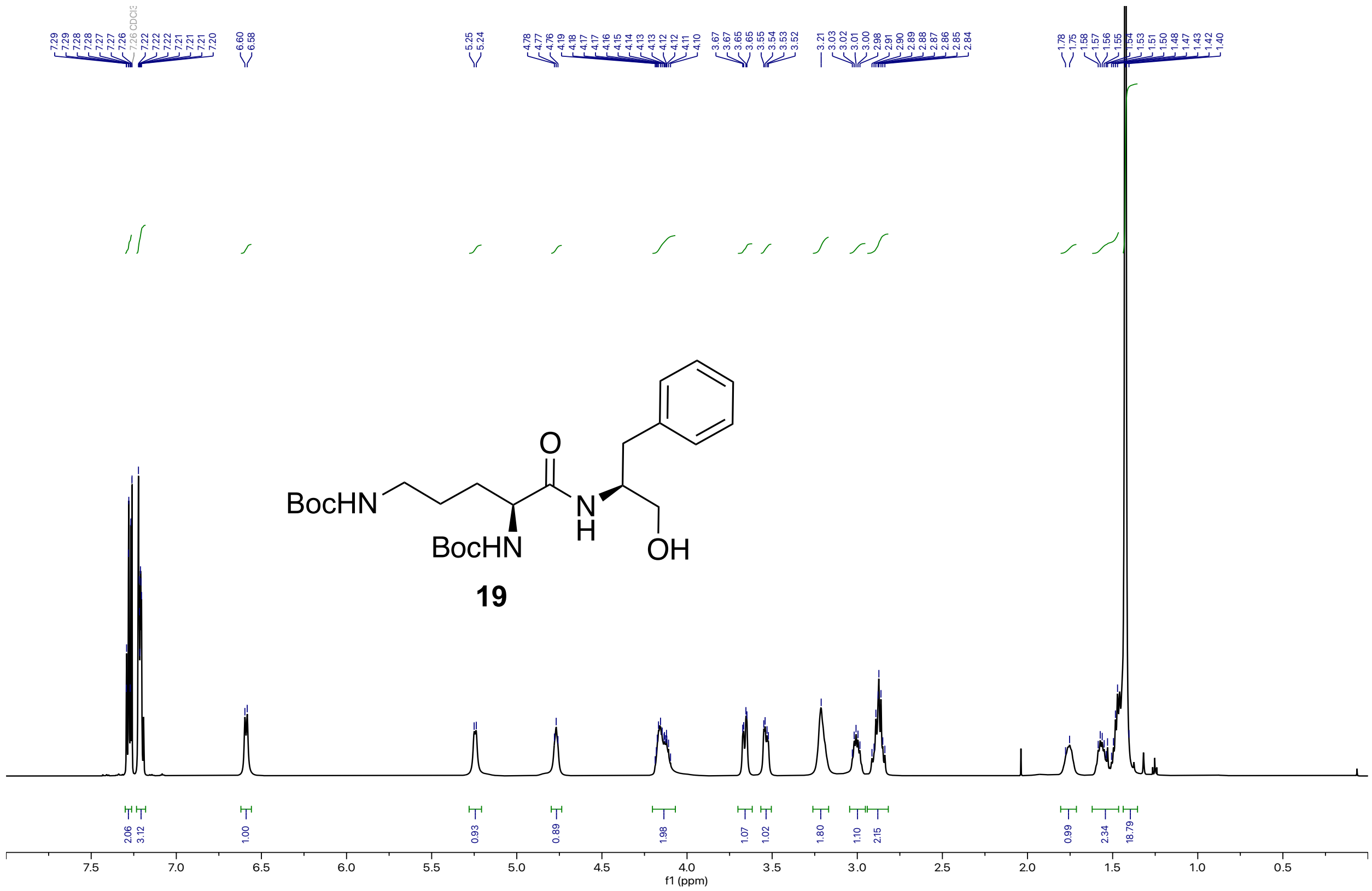
BocHN
O
N
H
BocHN
OH
19
f1 (ppm)

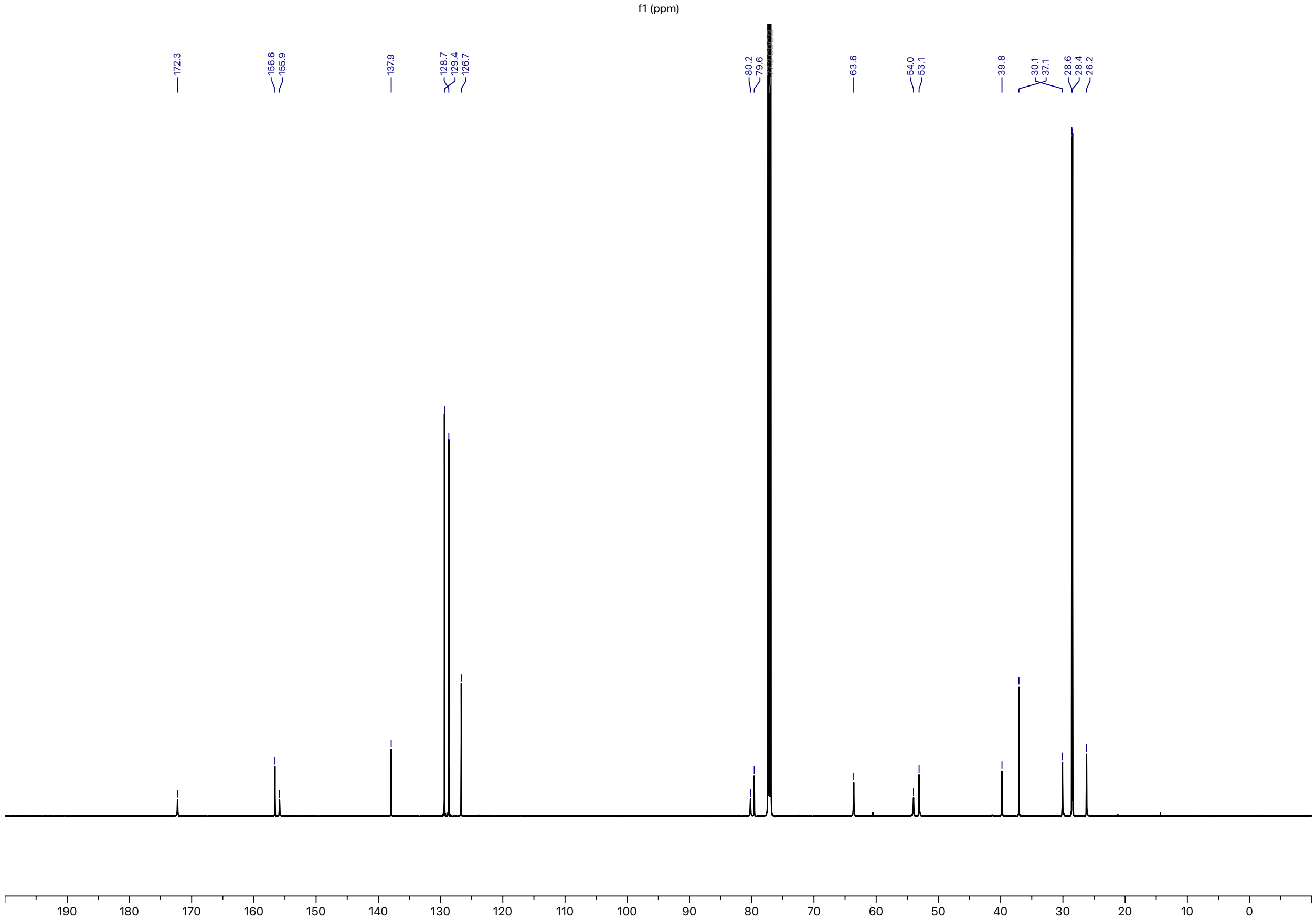
172.3
156.6
155.9
137.9
128.7
129.4
126.7
80.2
79.6
63.6
54.0
53.1
39.8
30.1
37.1
28.6
28.4
26.2
f1 (ppm)

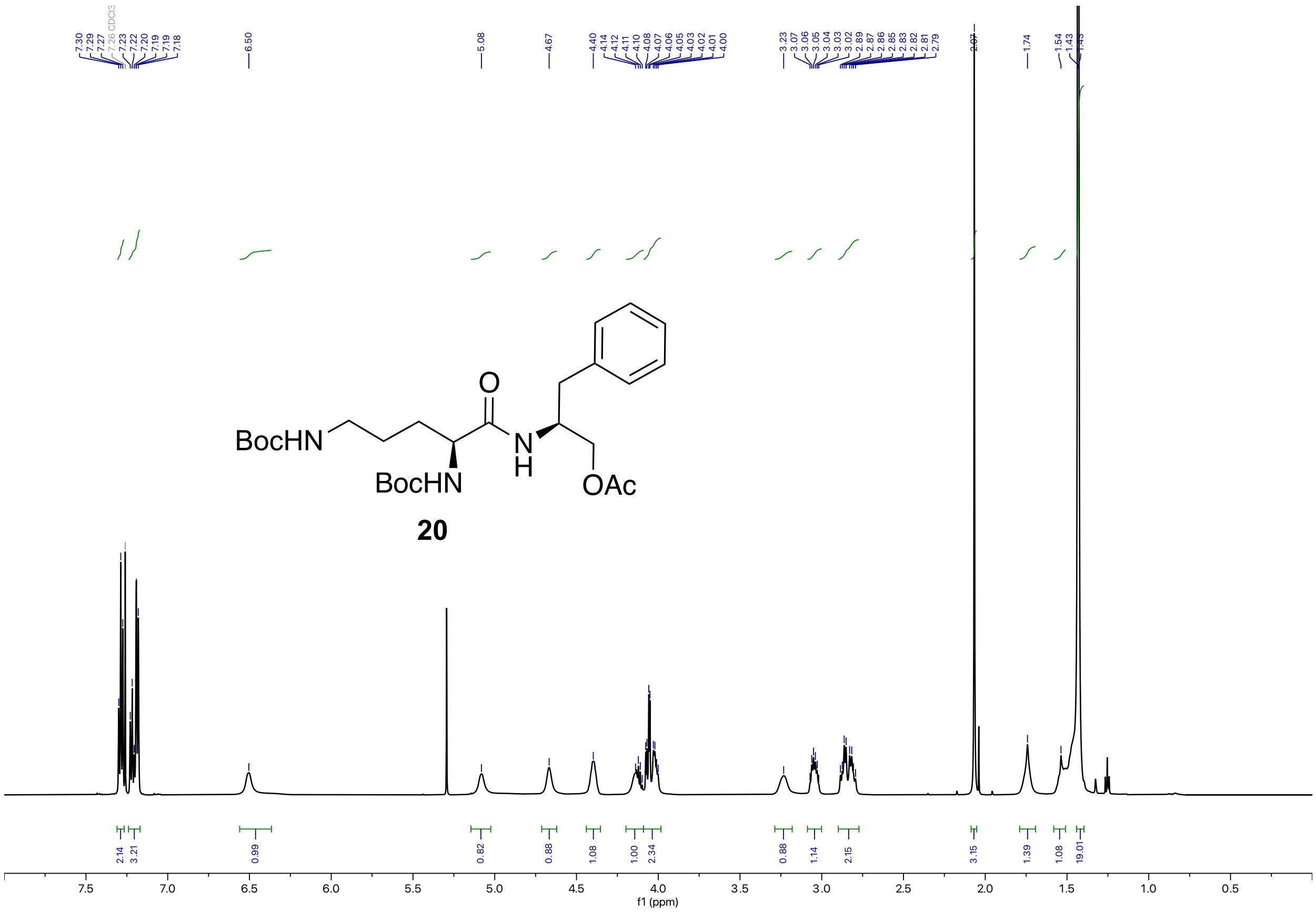
BocHN
BocHN
O
N
H
OAc
20
f1 (ppm)

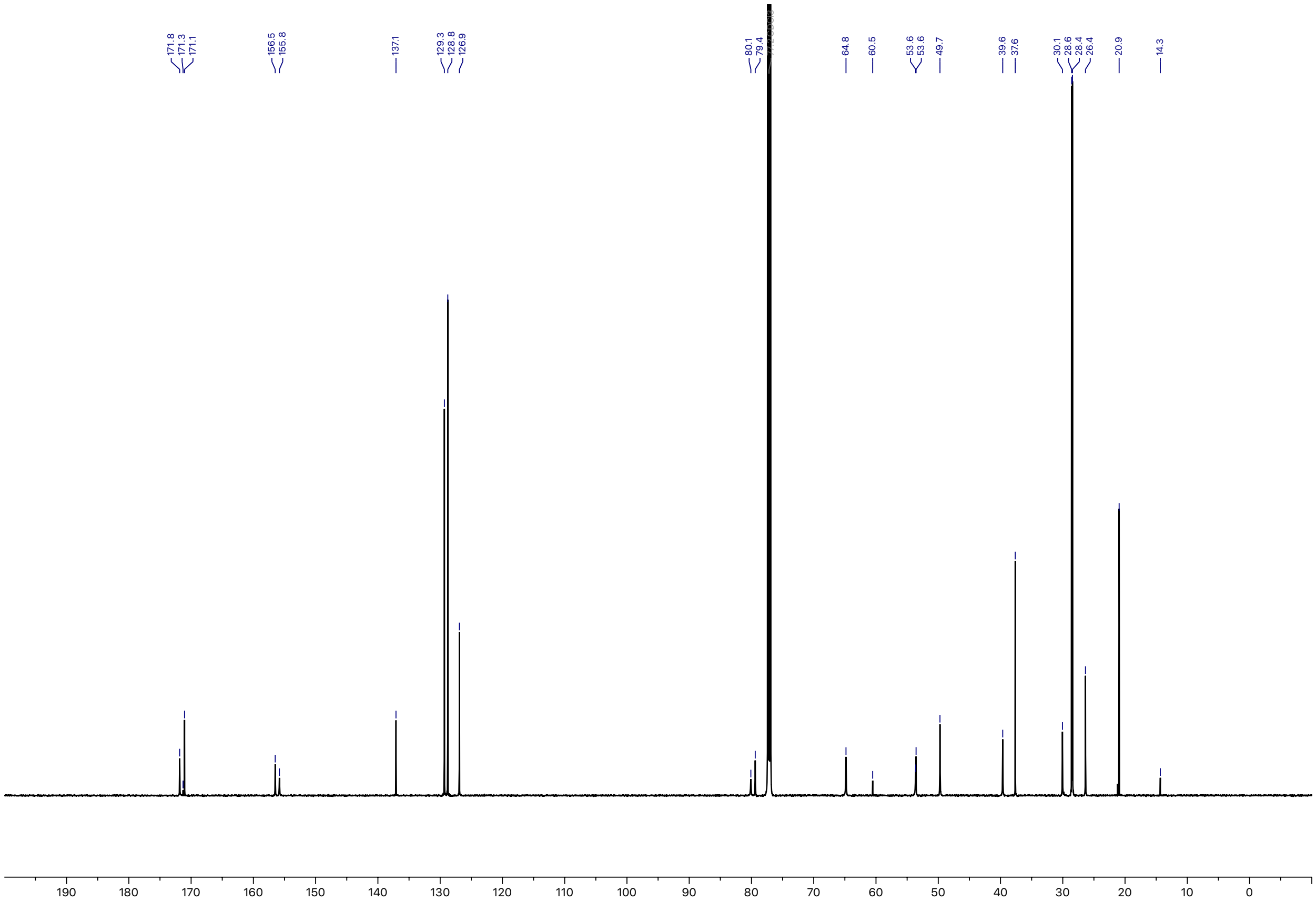
f1 (ppm)

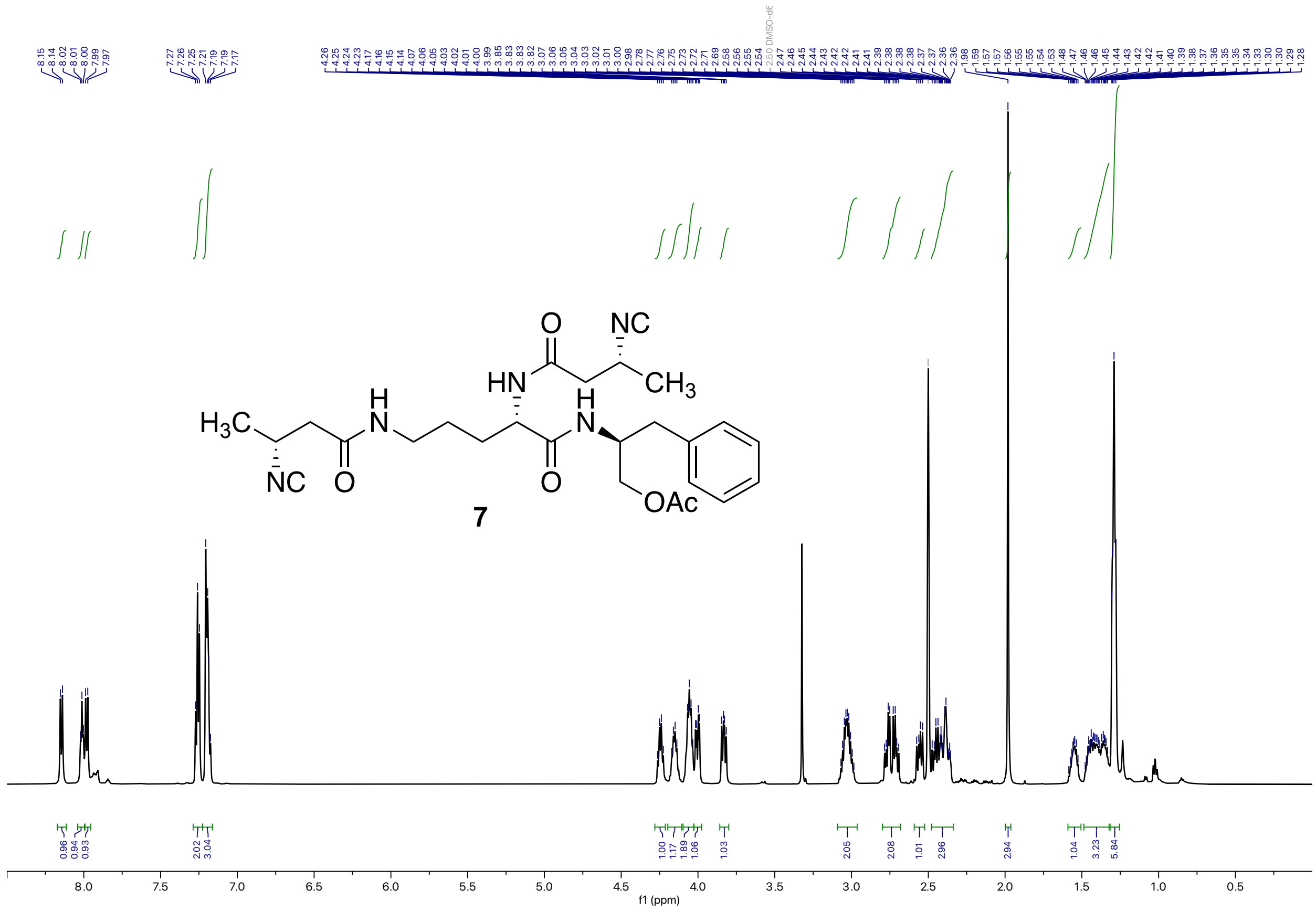
H
N
HN
O
NC
CH3
H3C
NC
O
O
N
H
OAc
7
f1 (ppm)

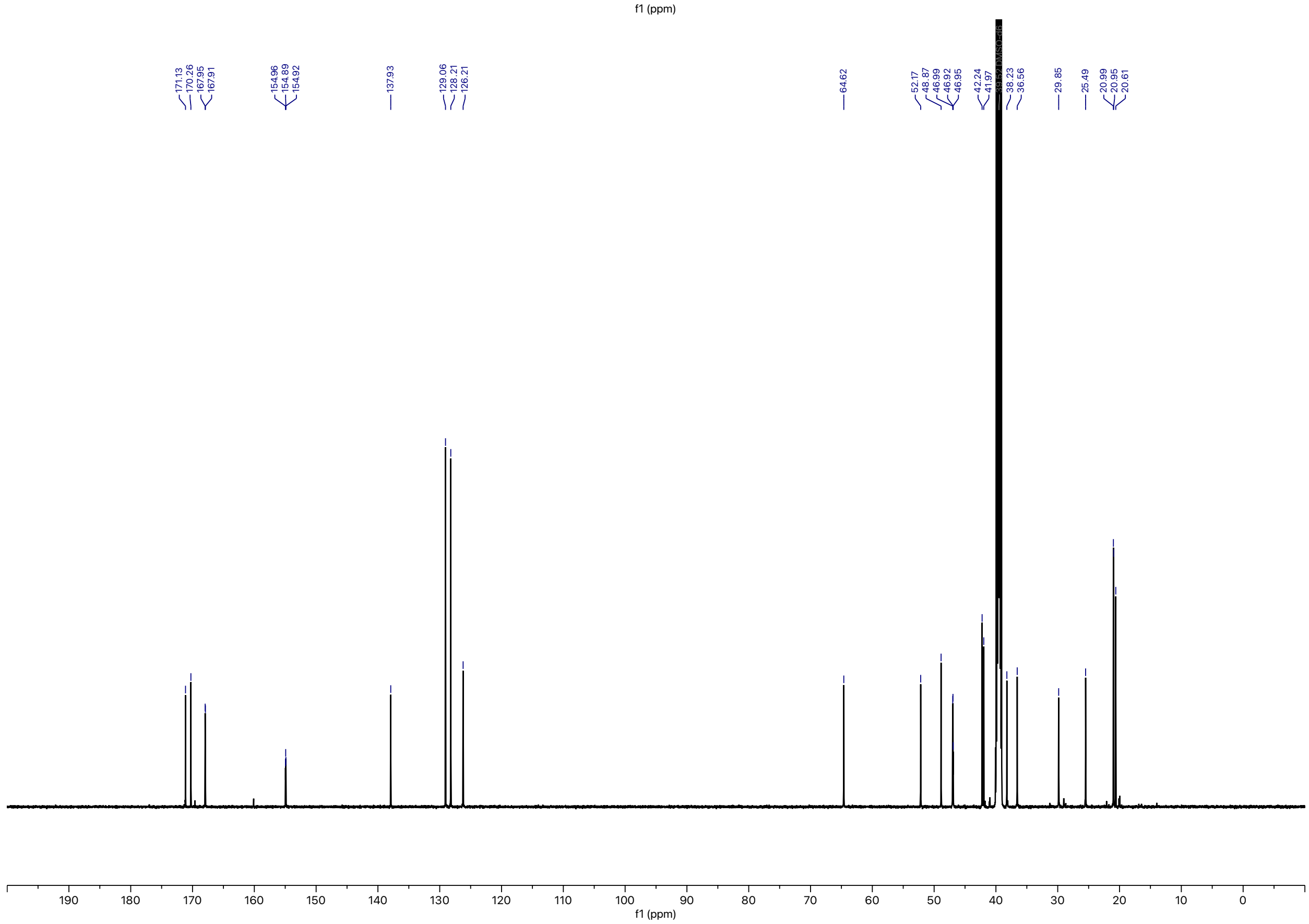
f1 (ppm)

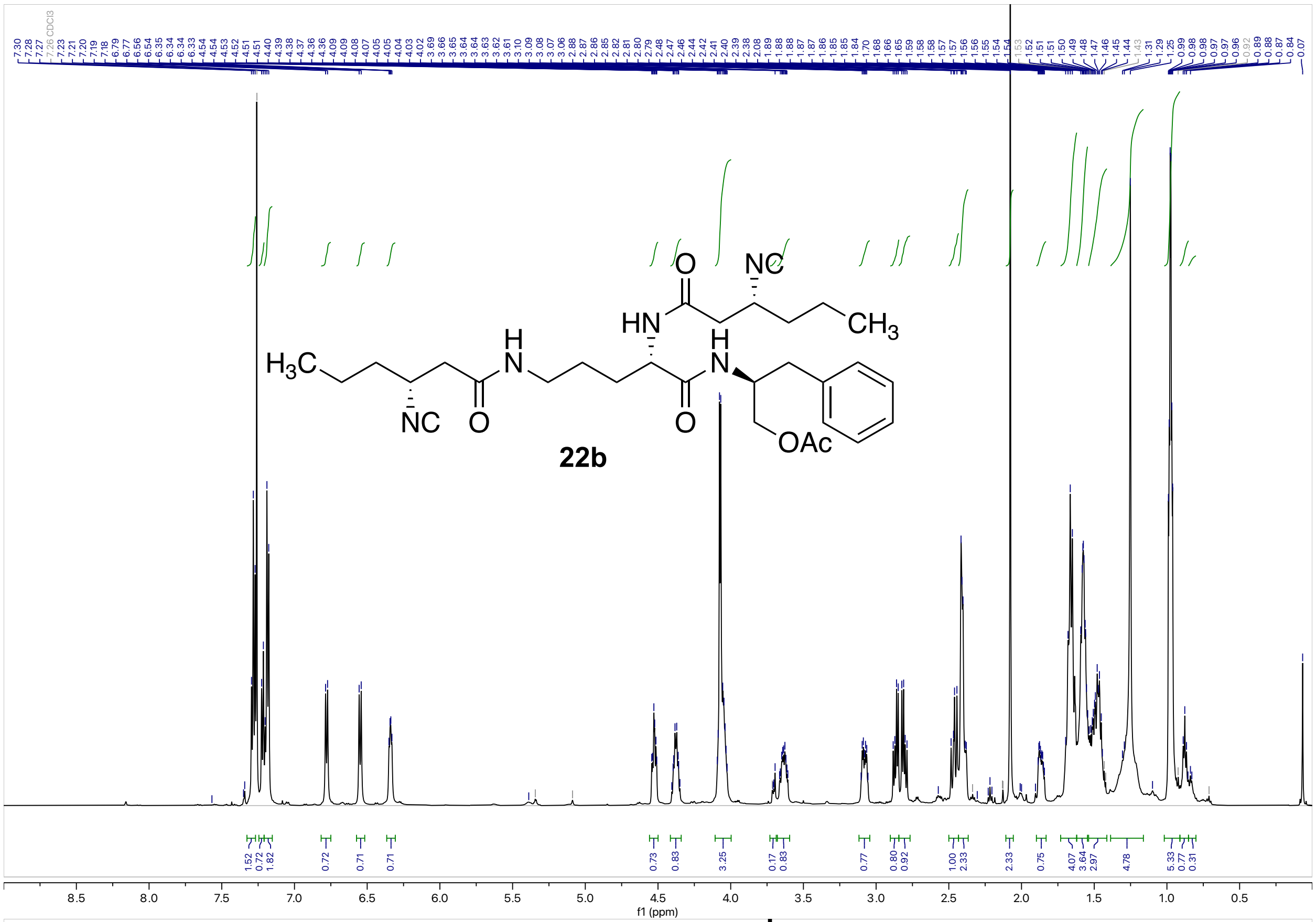
O
NC
HN
CH3
H
H3C
N
N
NC
O
O
OAc
22b
f1 (ppm)

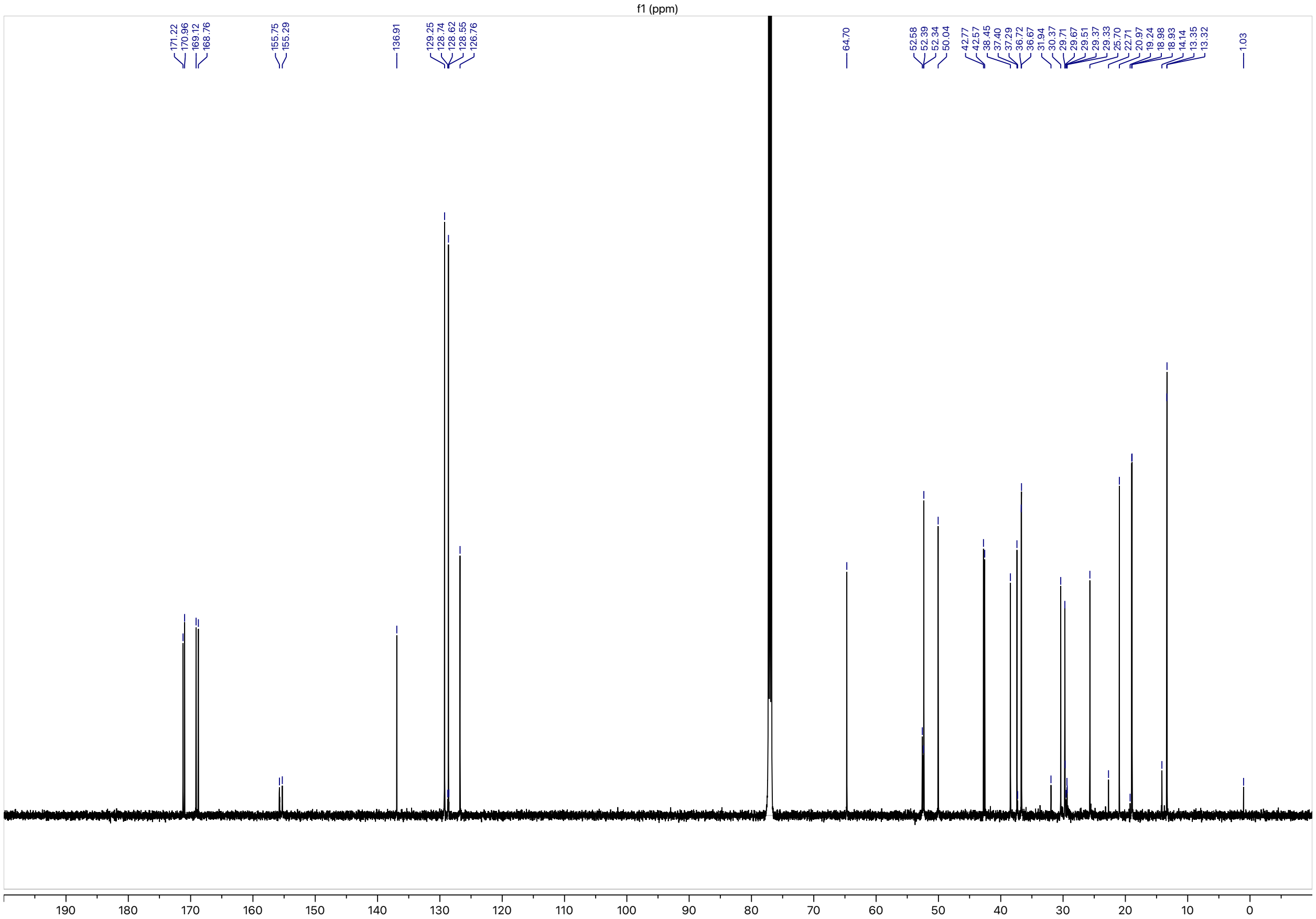
f1 (ppm)

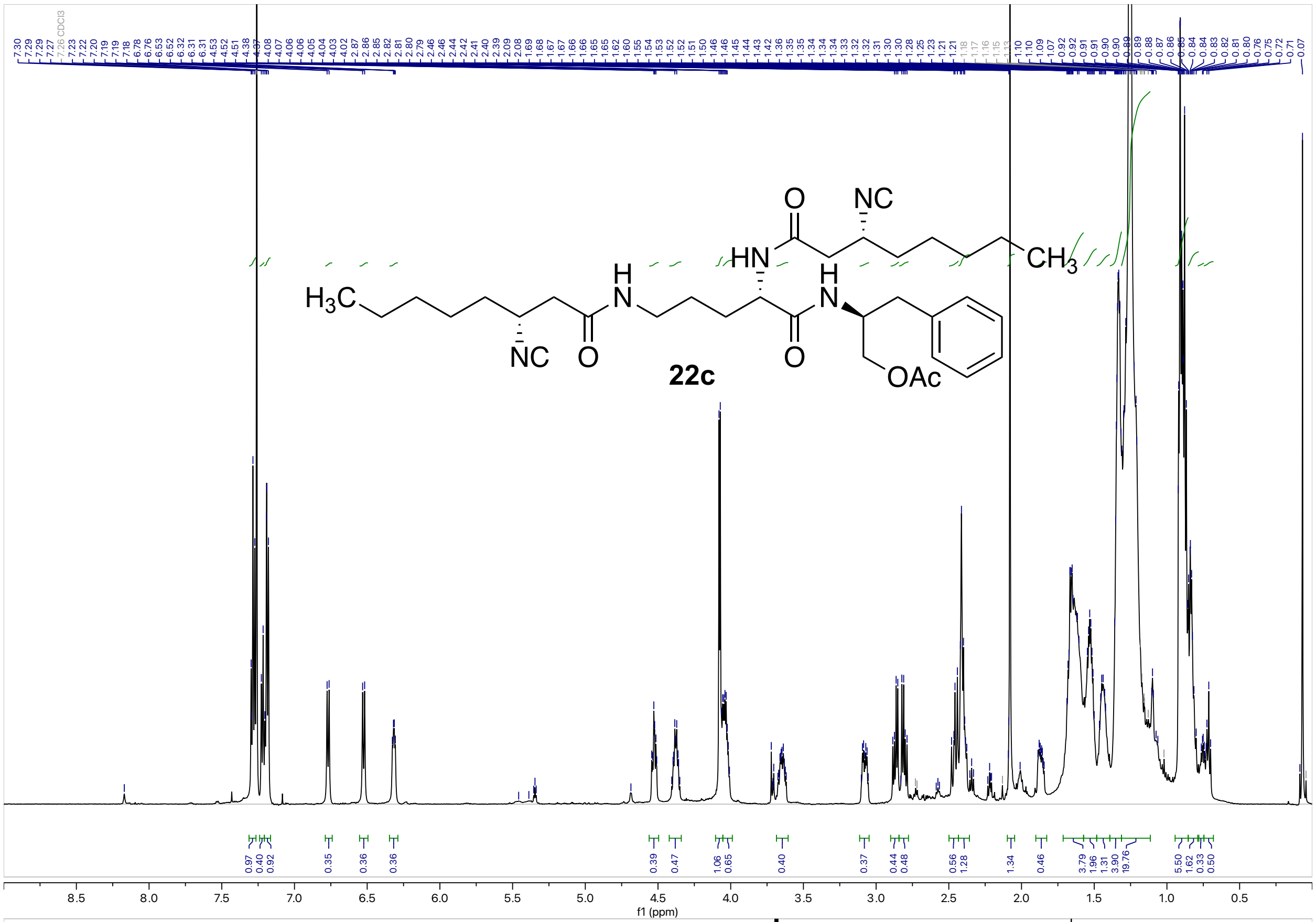
O
NC
HN
H
N
H
N
H_3C
CH_3
NC
O
22c
O
OAc
f1 (ppm)

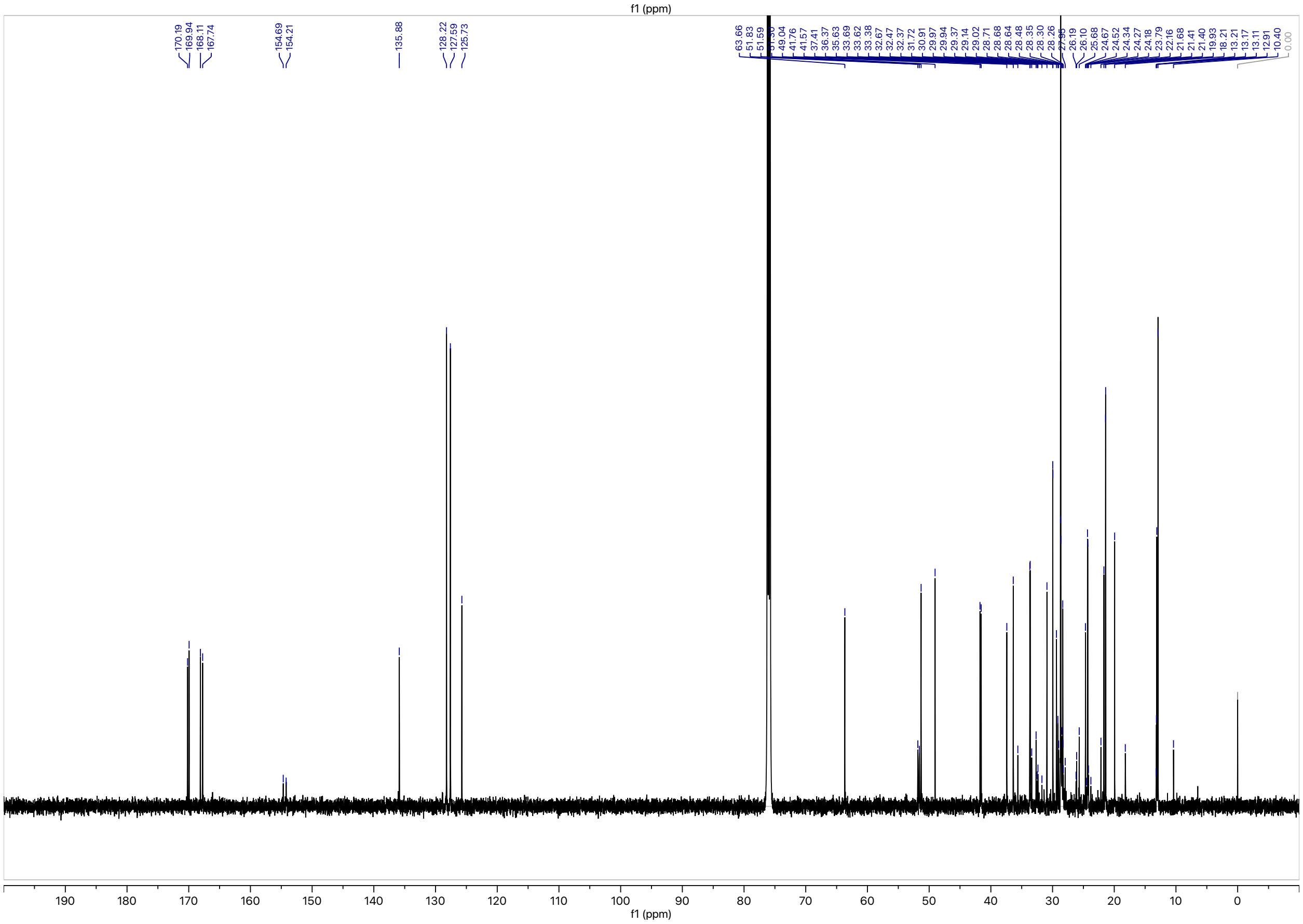
f1 (ppm)

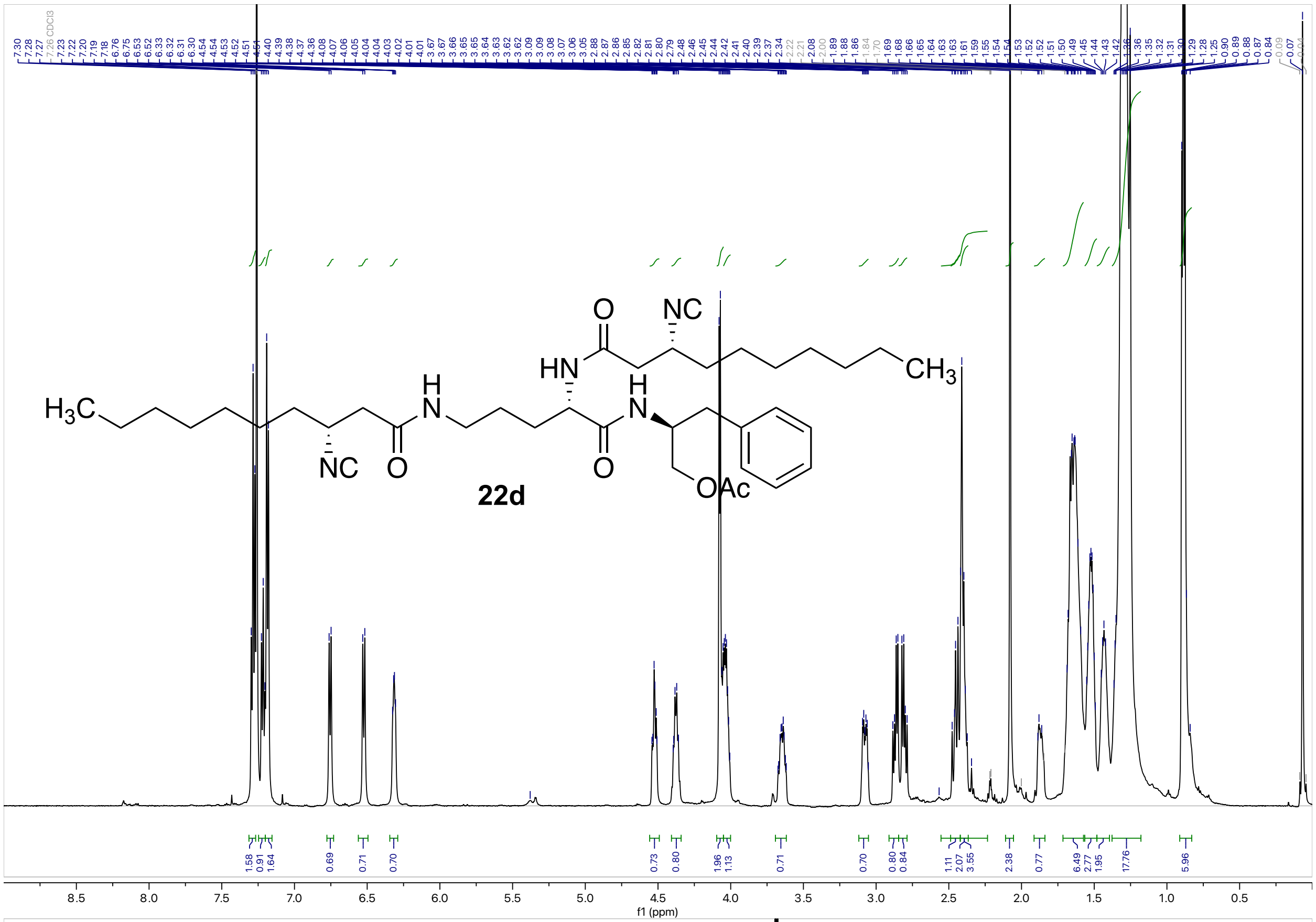
H3C
NC
O
H
N
HN
NC
O
H
N
O
CH3
OAc
O
22d
f1 (ppm)

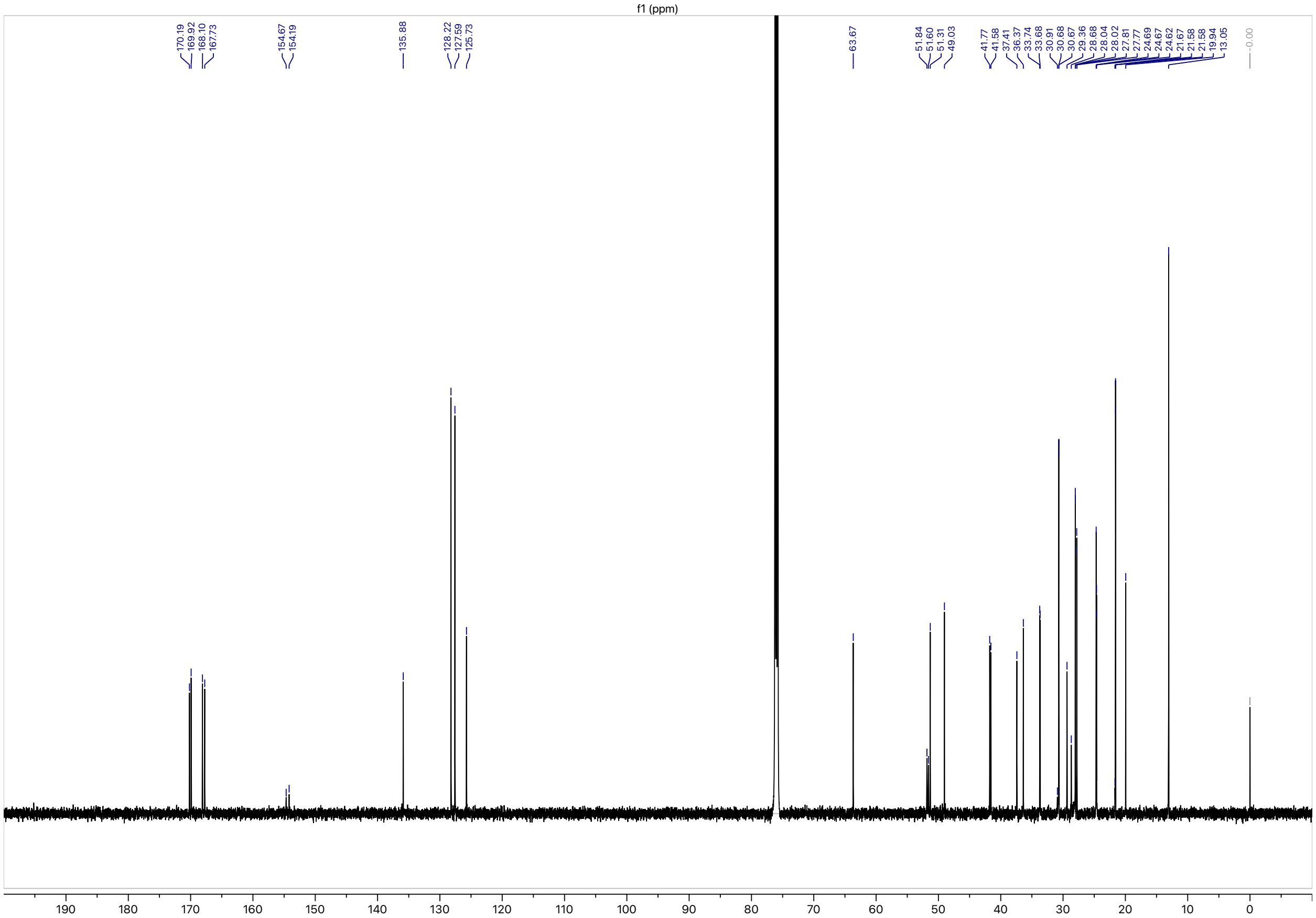
f1 (ppm)

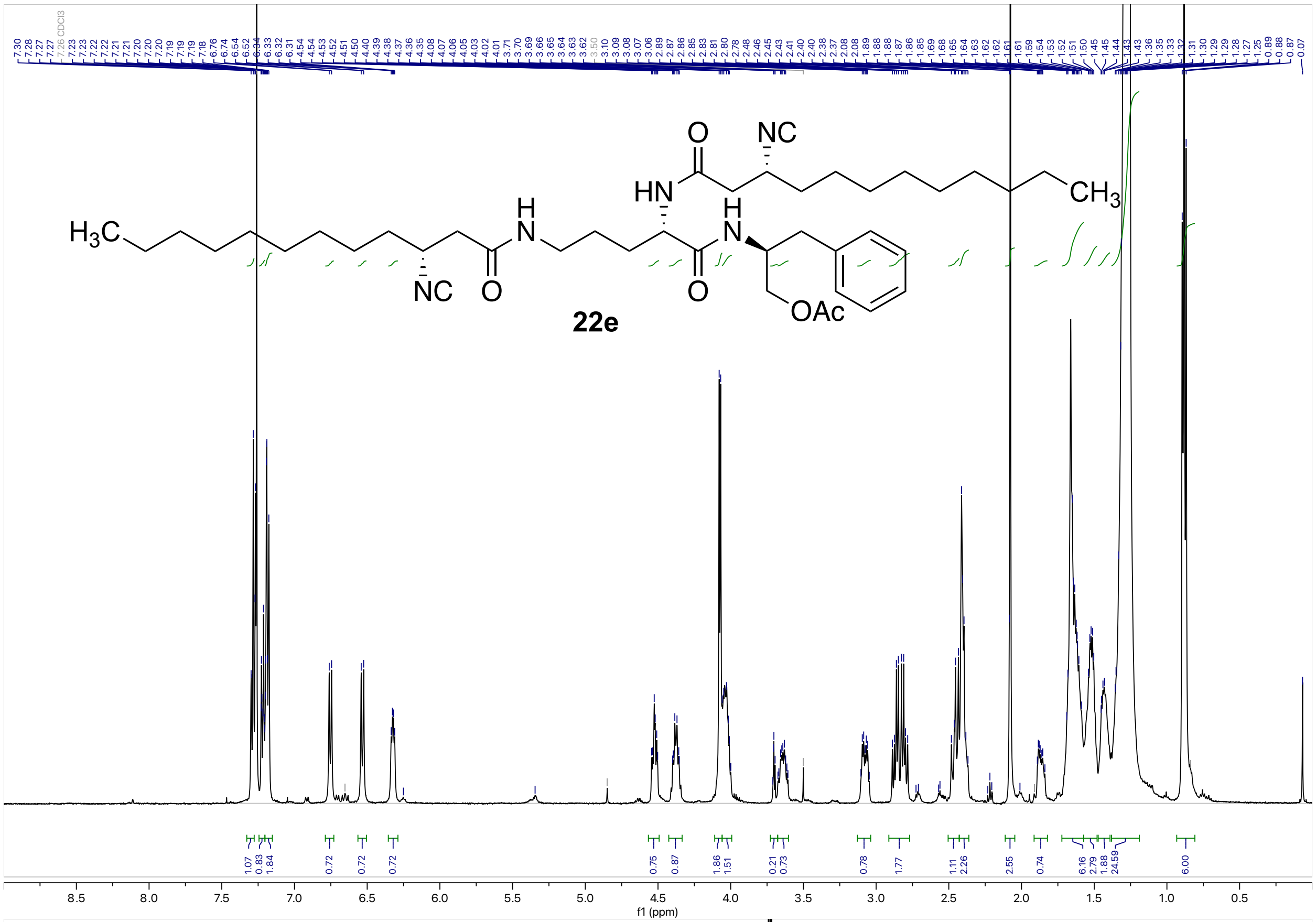
22e
OAc
f1 (ppm)

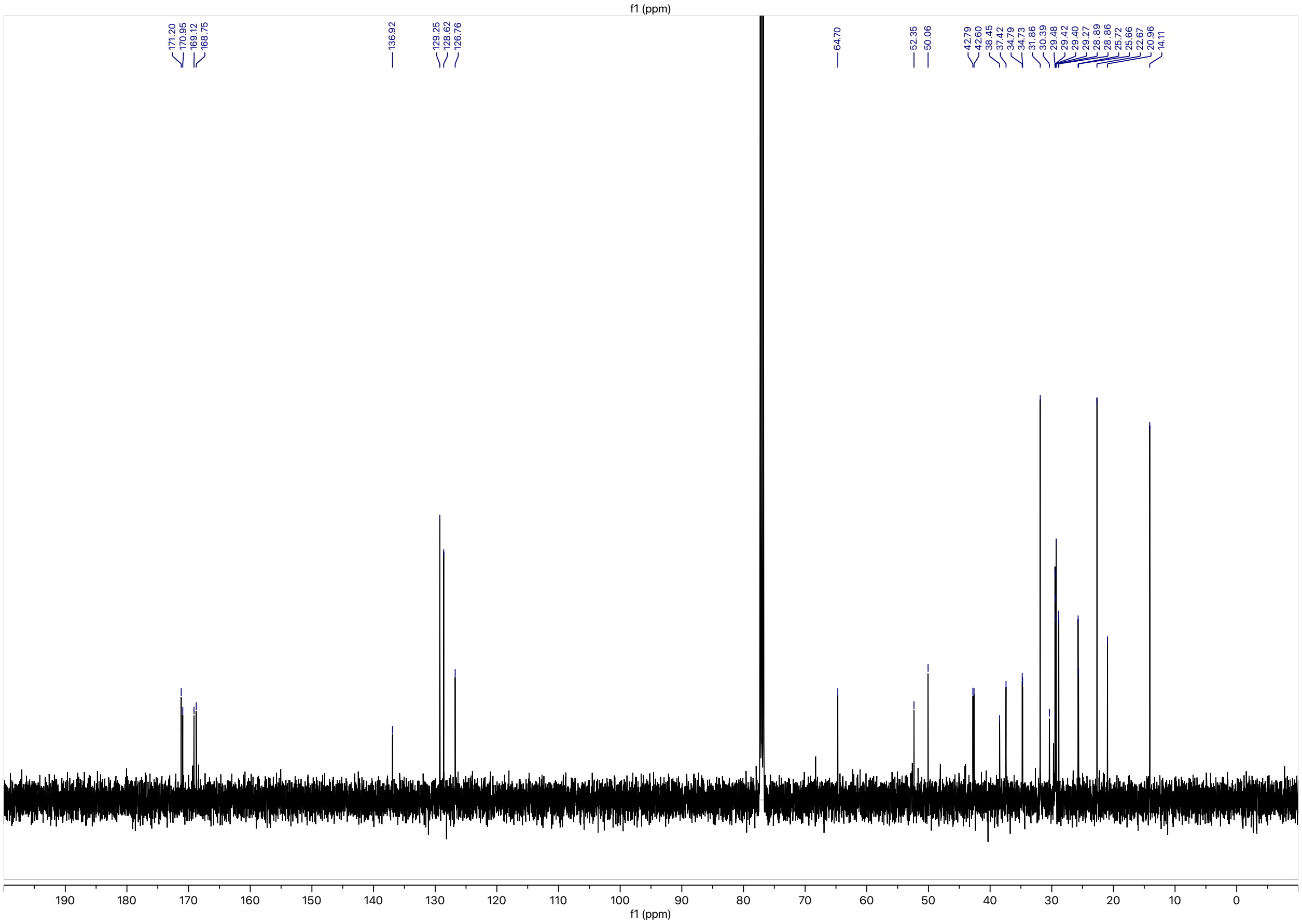
f1 (ppm)

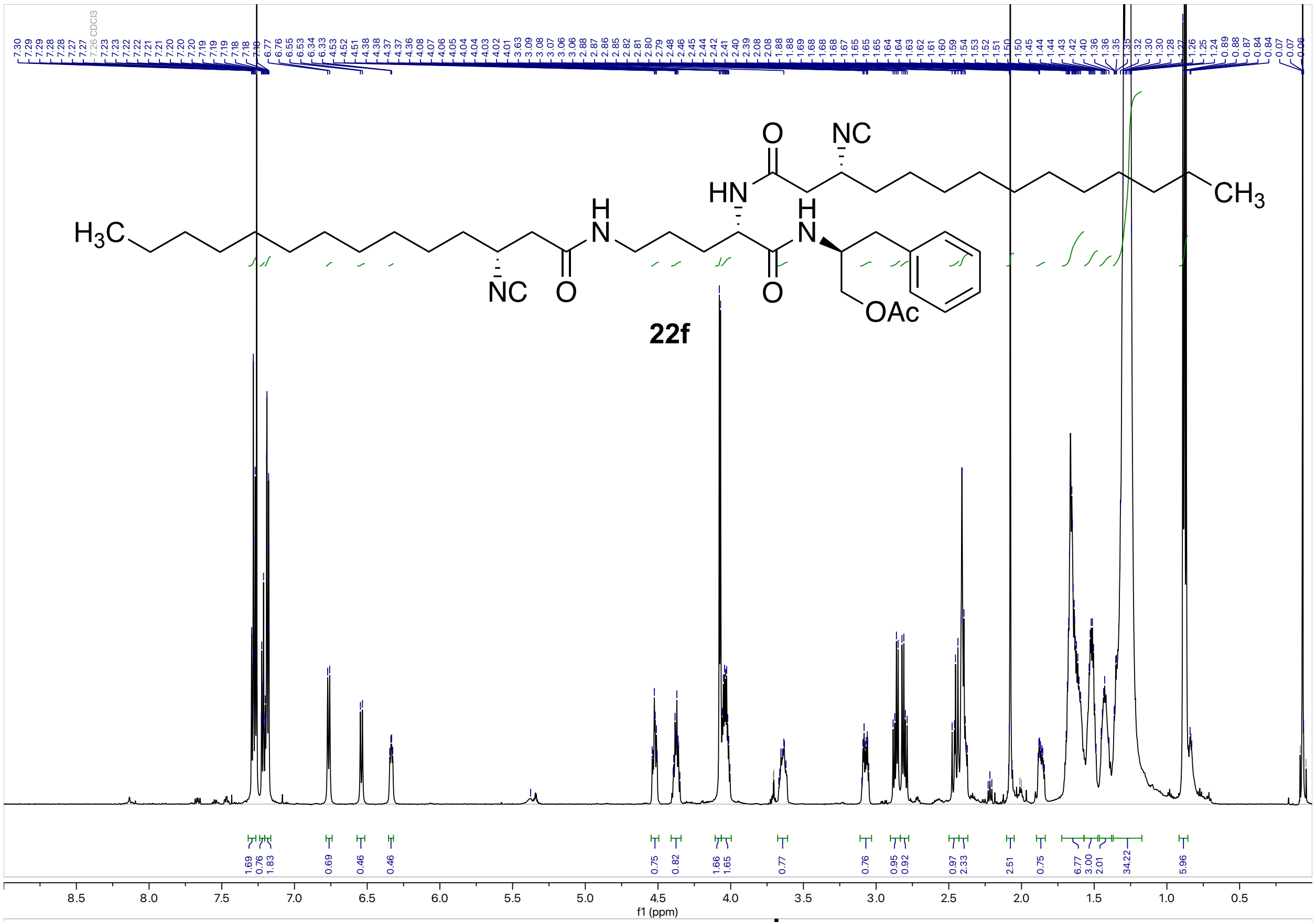

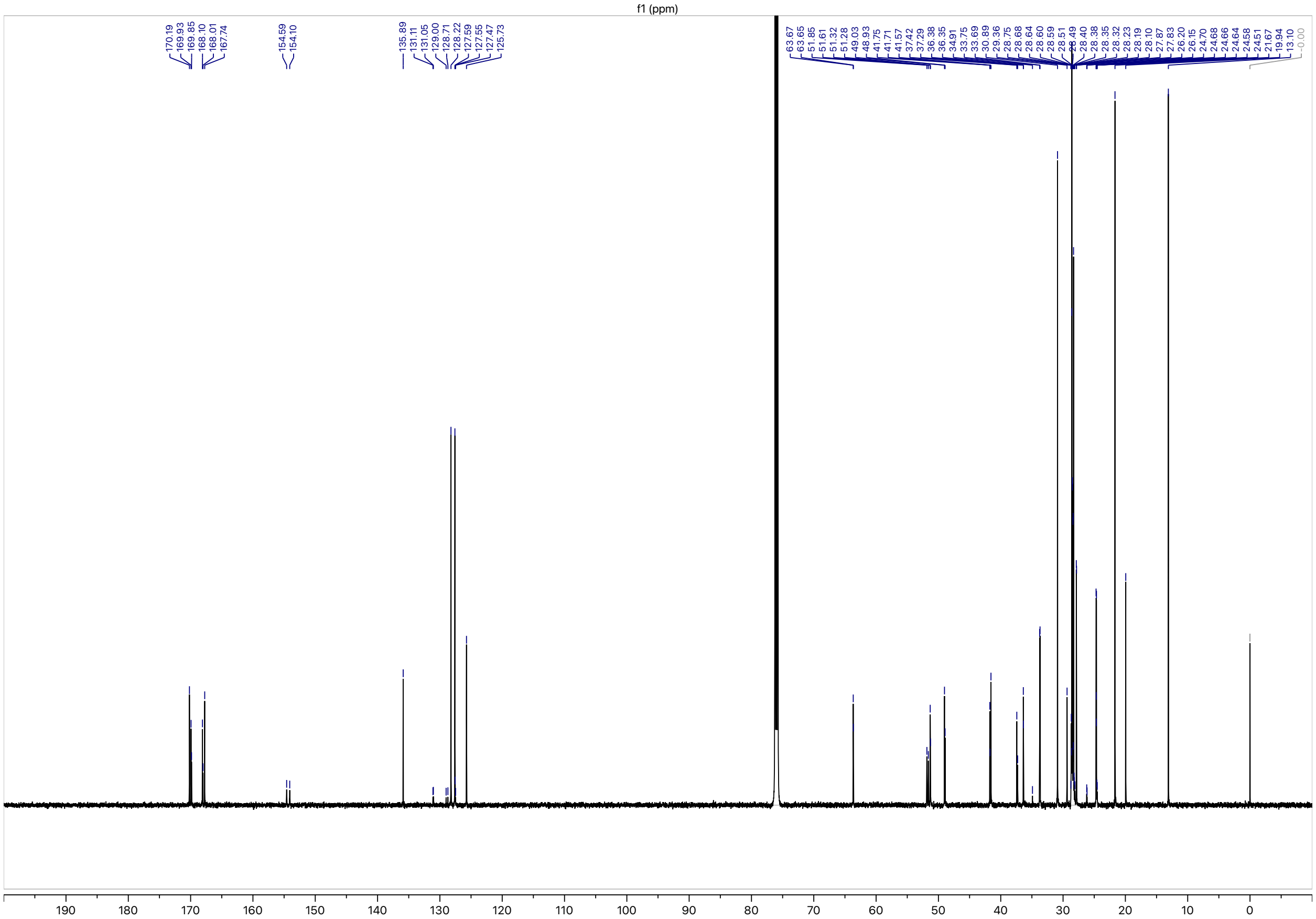

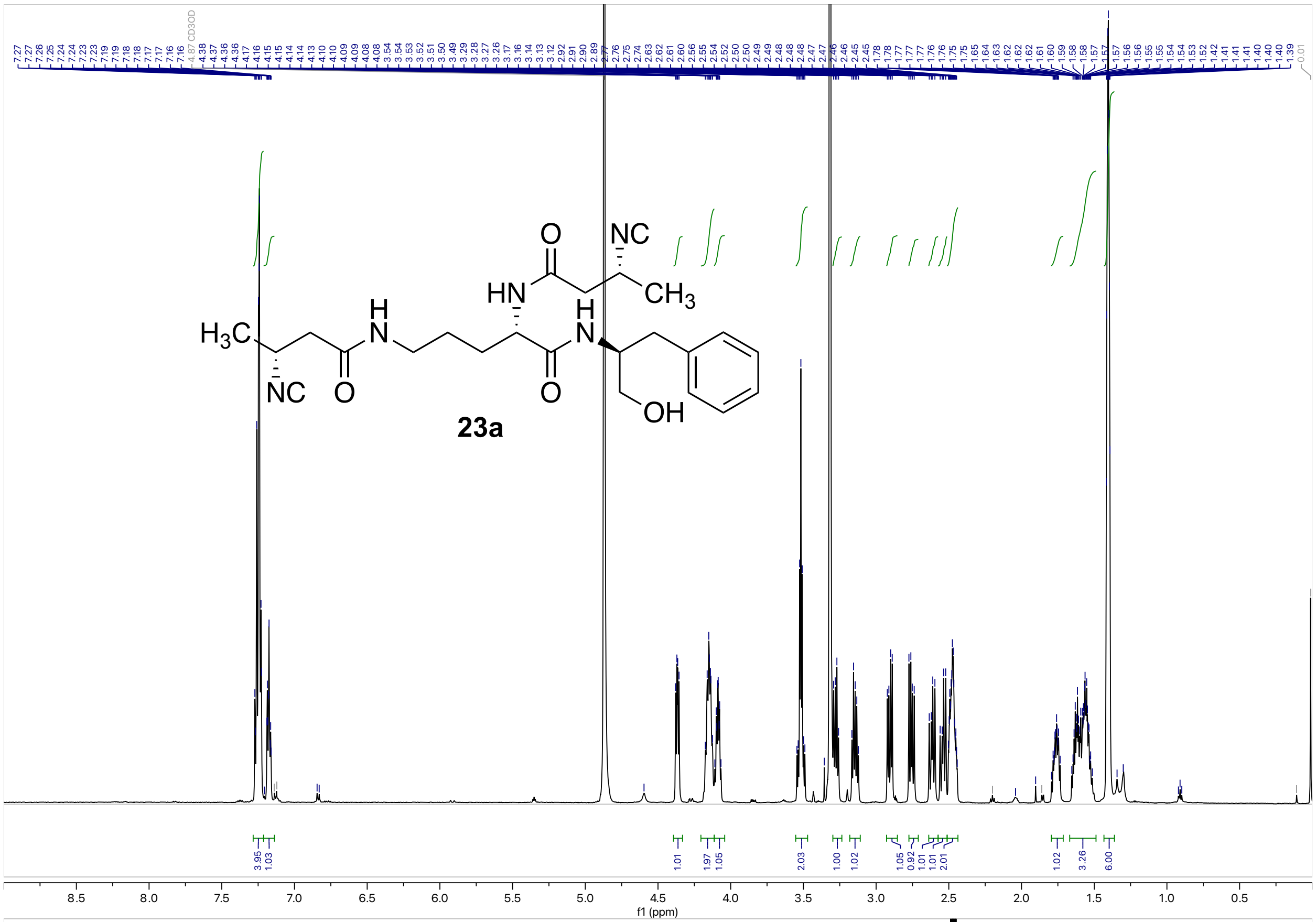
CD3OD
H3C
NC
O
H
N
HN
CH3
OH
23a
f1 (ppm)

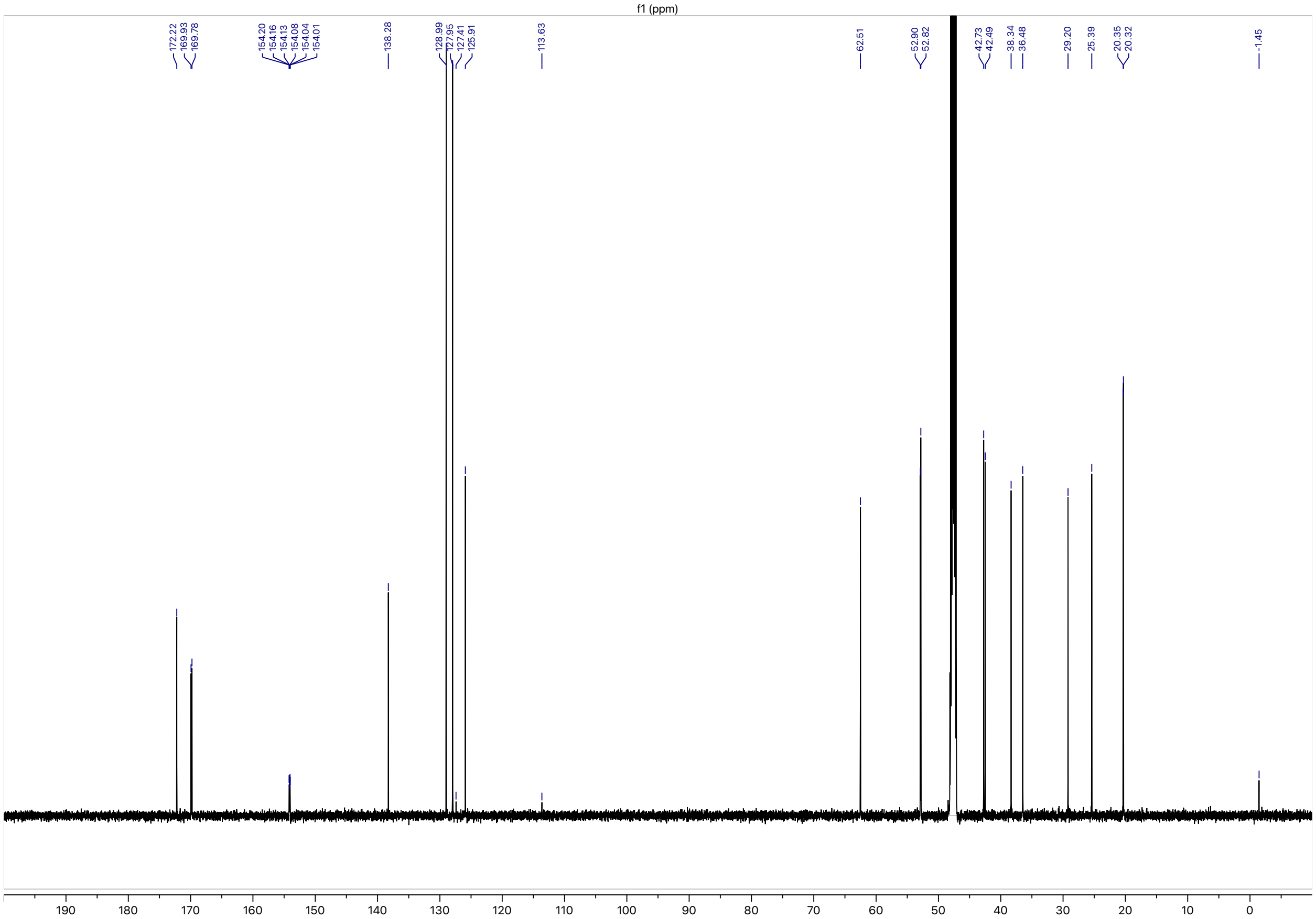
f1 (ppm)

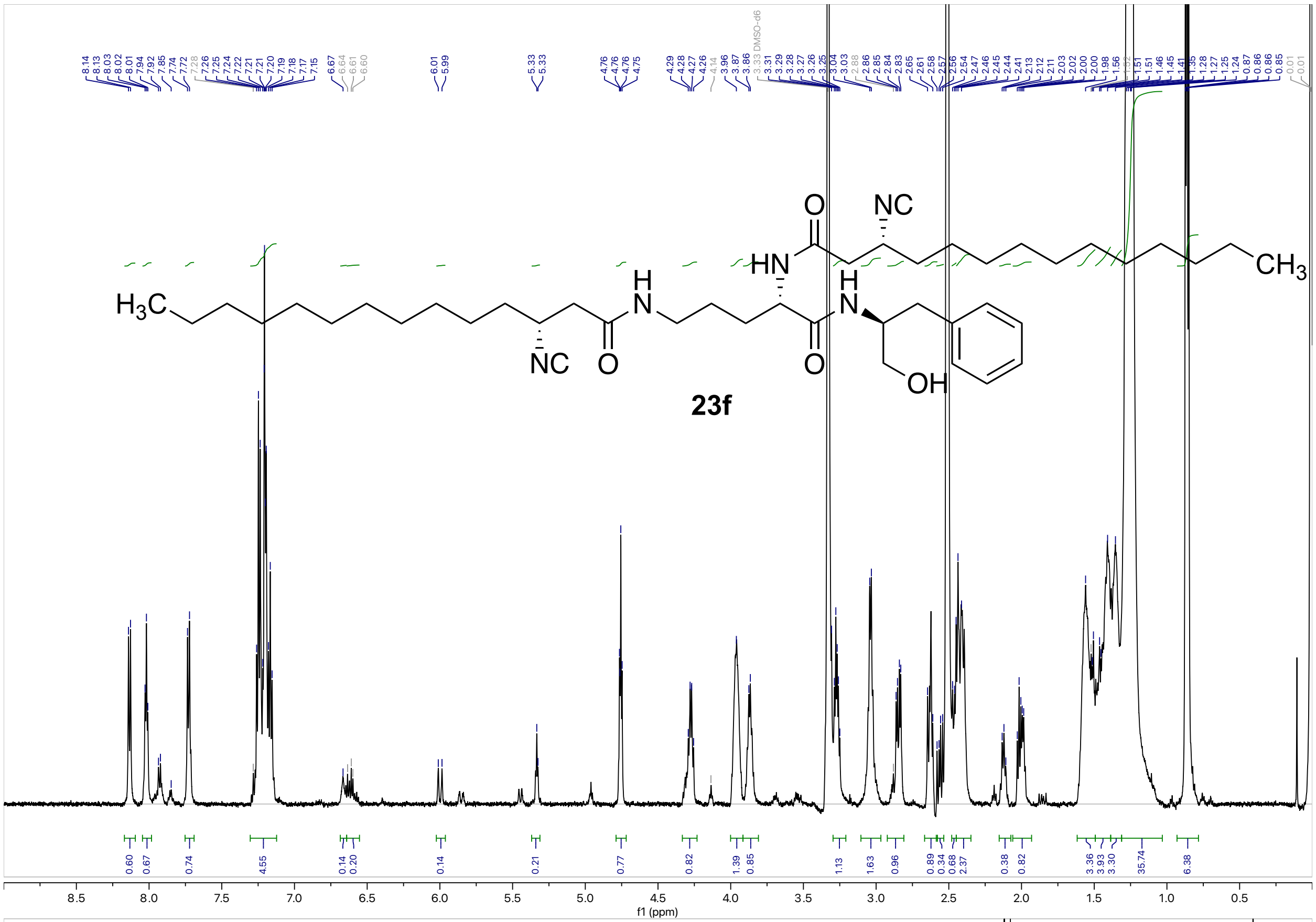

O
NC
HN
H
N
H
N
H3C
NC
O
O
OH
CH3
23f
f1 (ppm)

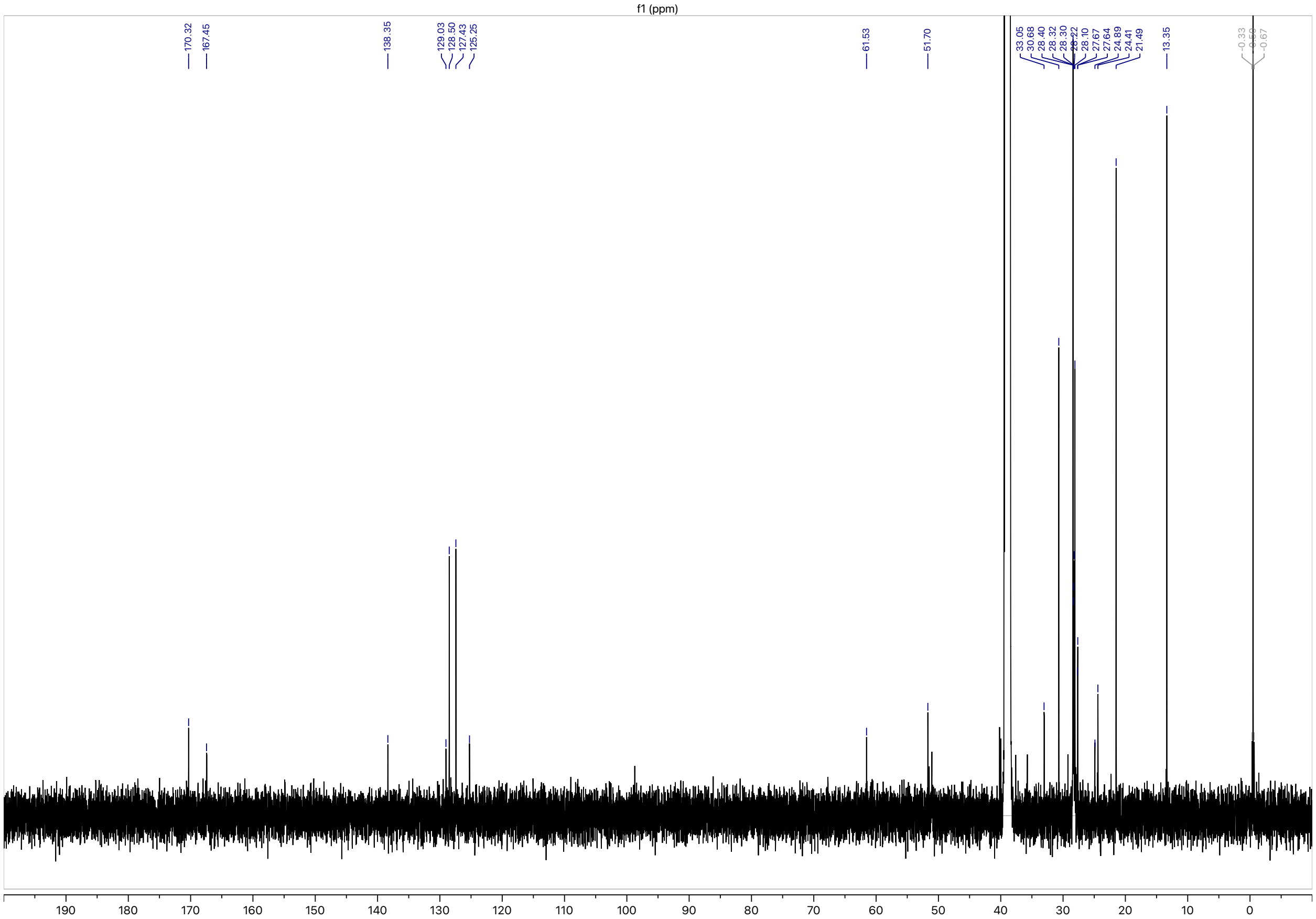

f1 (ppm)

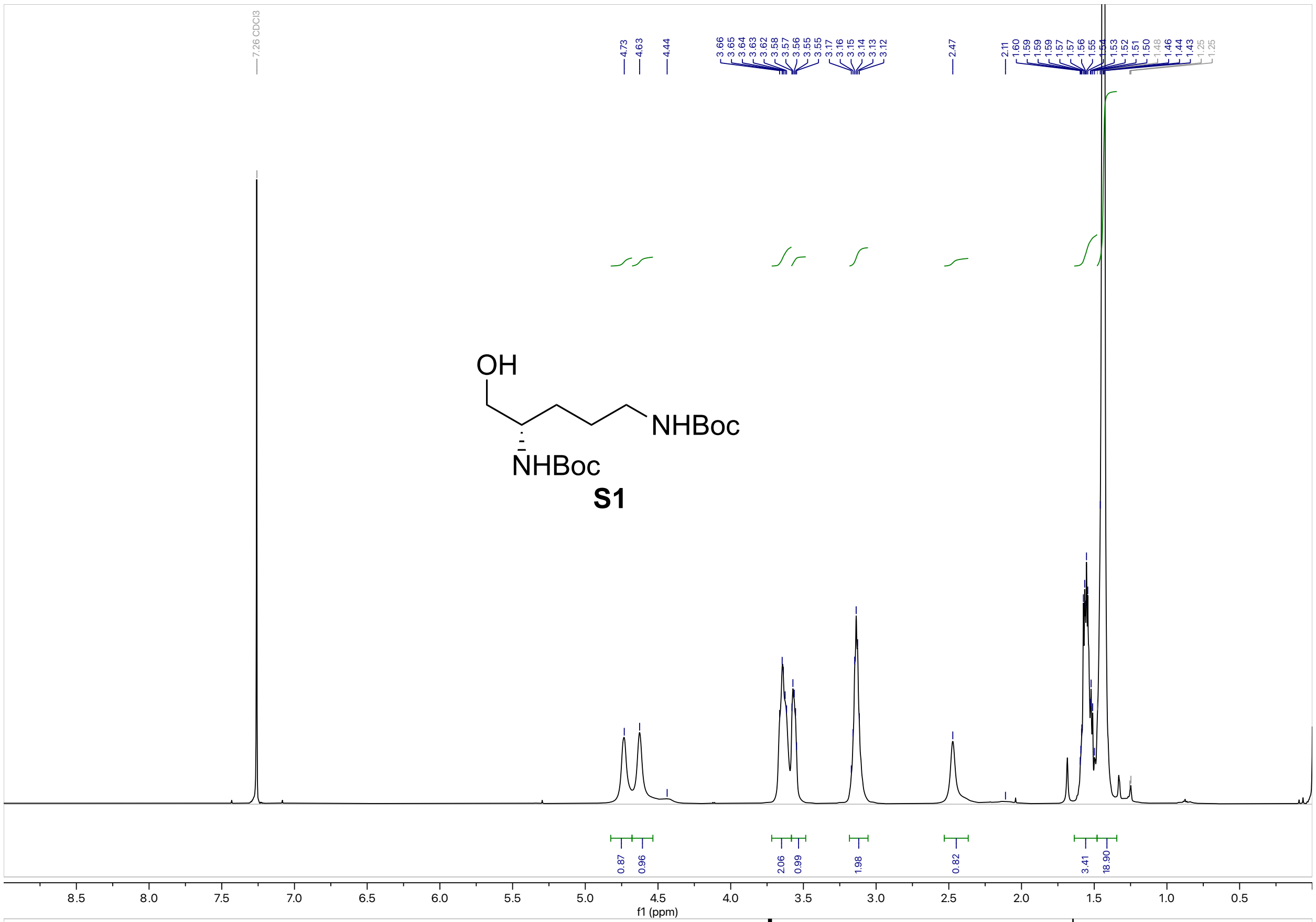
S1

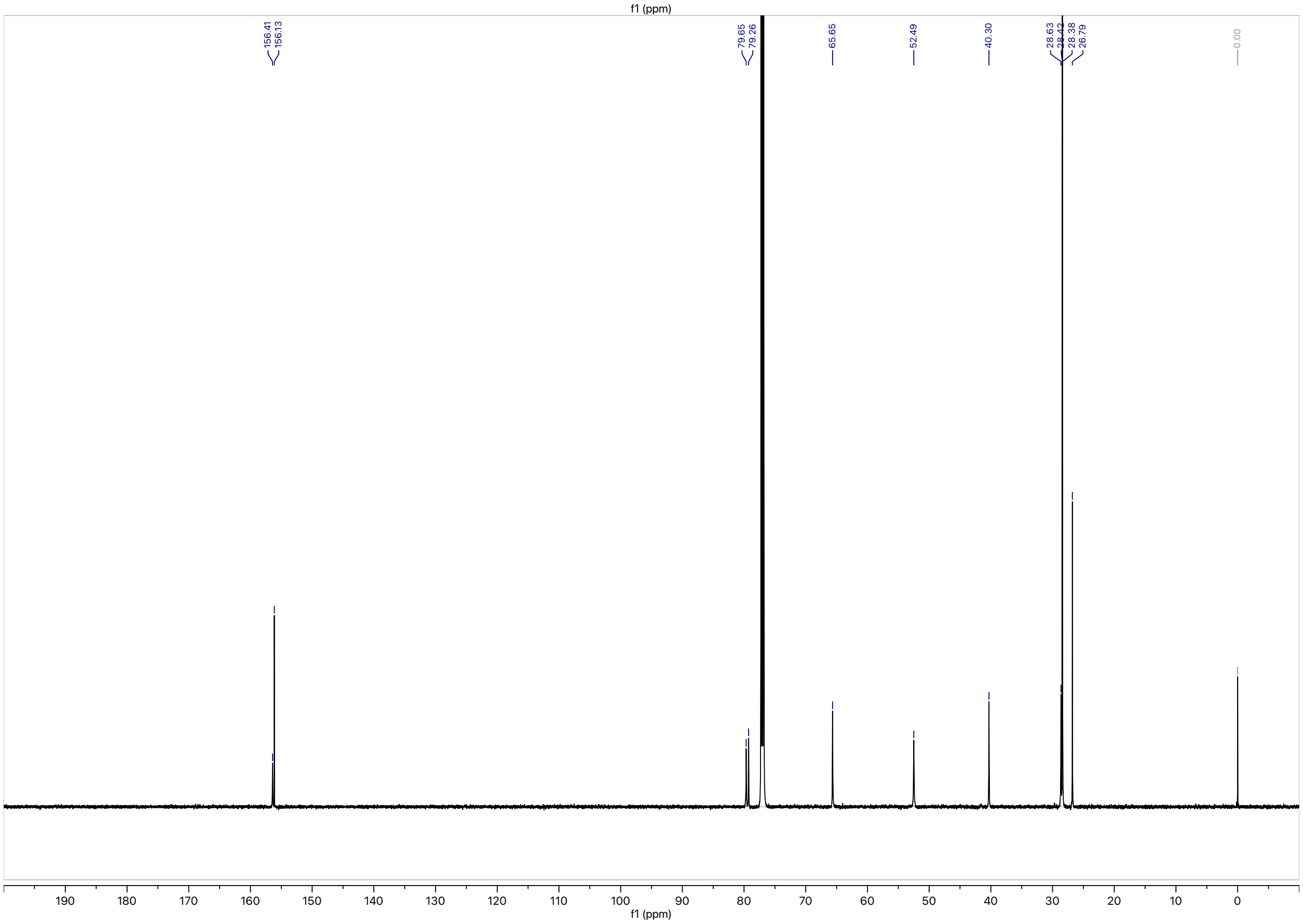

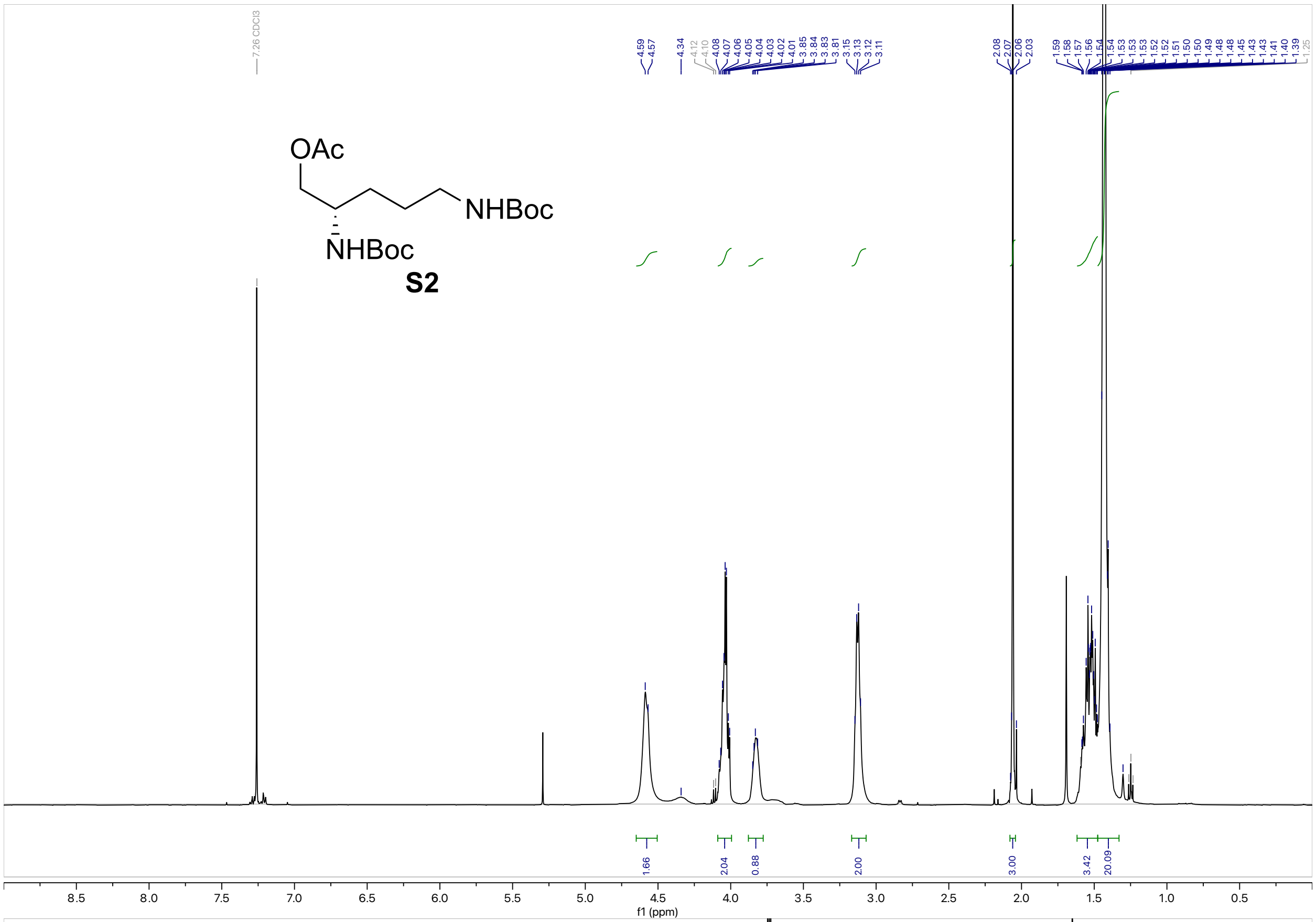

S2

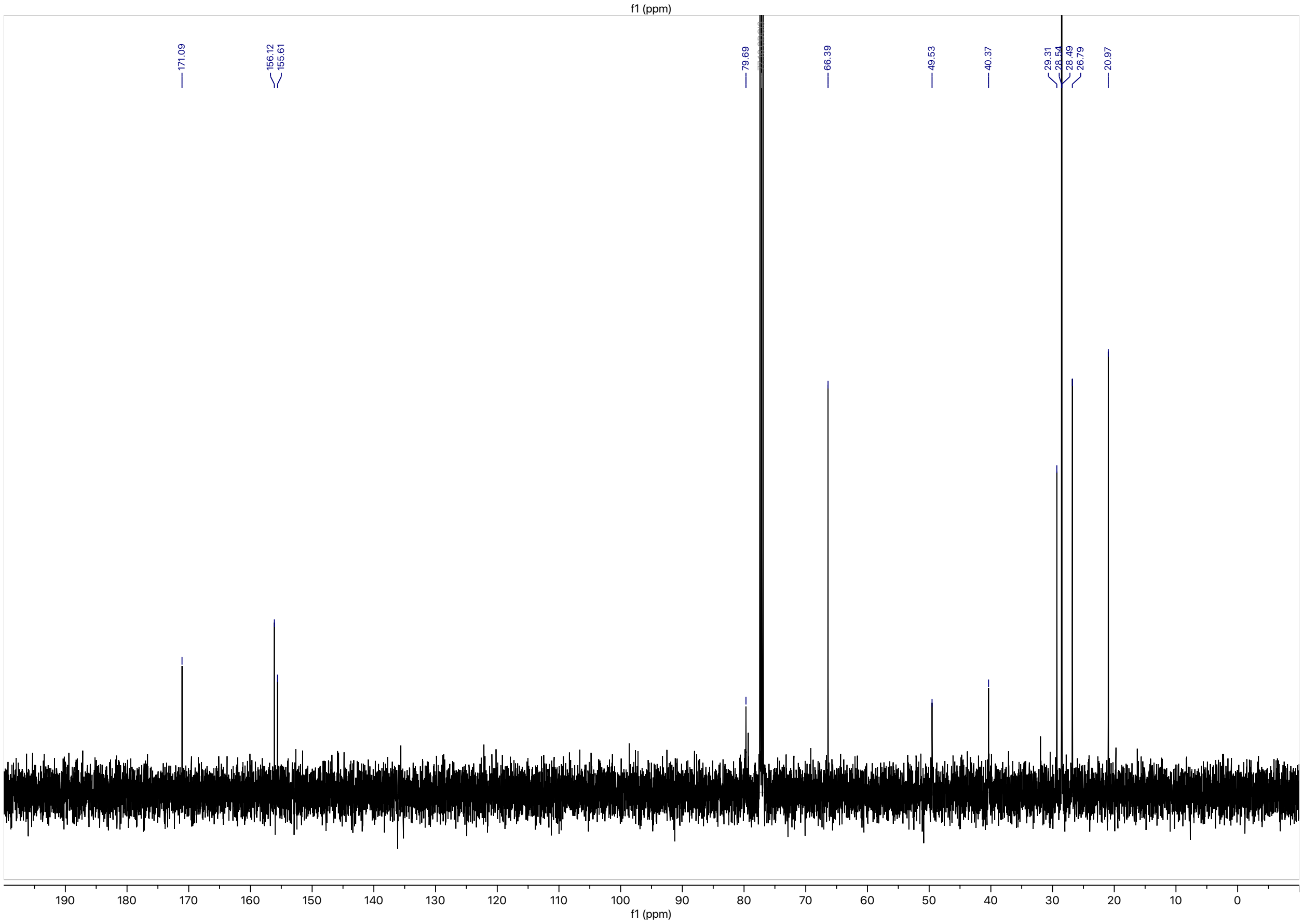

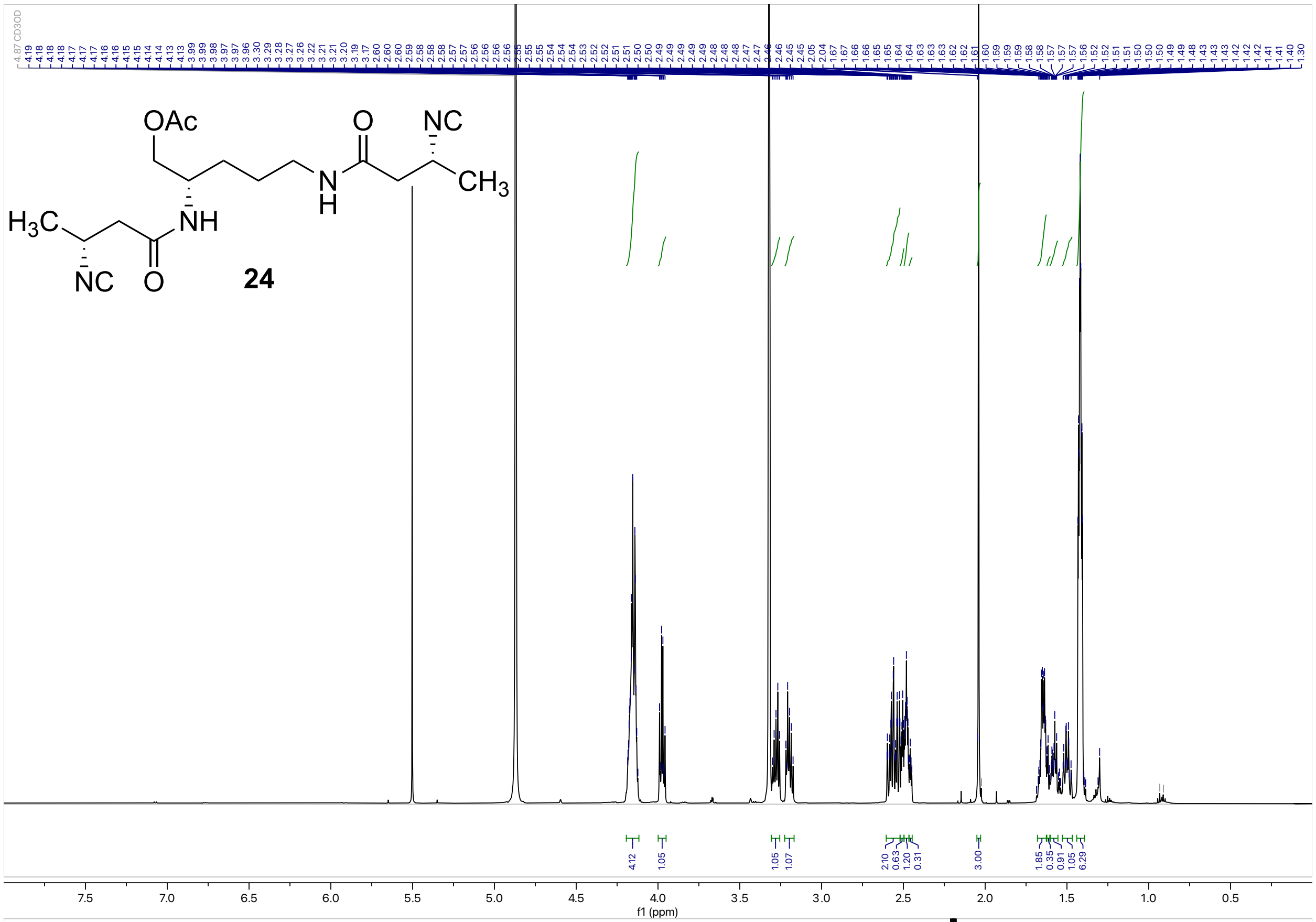
CD3OD
OAc
O
NC
N
H
CH$_3$
H$_3$C
NH
NC
O
24
4.12
1.05
1.05
1.07
2.10
0.63
1.20
0.31
3.00
1.85
0.35
0.91
1.05
6.29
7.5
7.0
6.5
6.0
5.5
5.0
4.5
4.0
3.5
3.0
2.5
2.0
1.5
1.0
0.5
f1 (ppm)

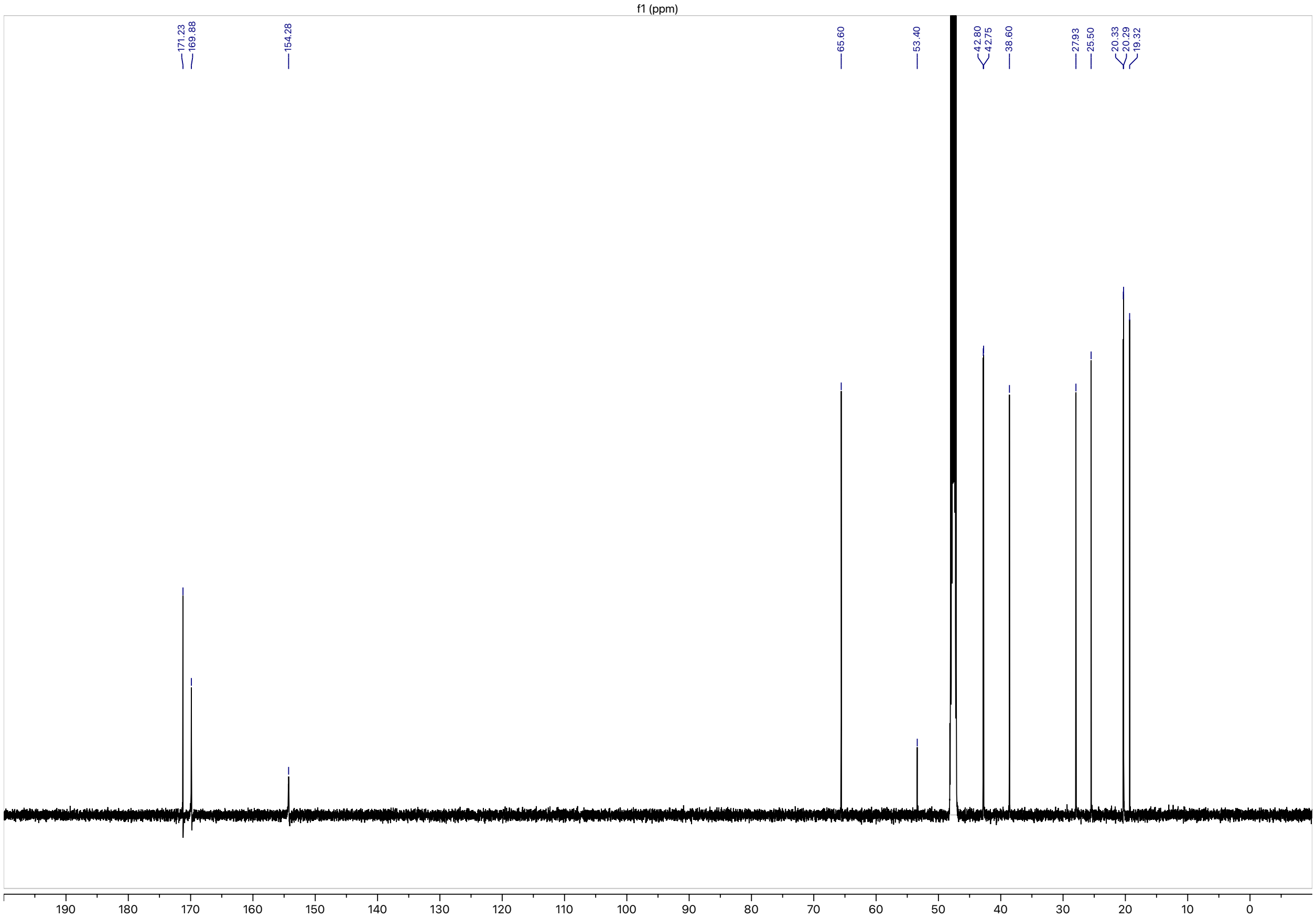
171.23
169.88
154.28
65.60
53.40
42.80
42.75
38.60
27.93
25.50
20.33
20.29
19.32
190
180
170
160
150
140
130
120
110
100
90
80
70
60
50
40
30
20
10
0
f1 (ppm)

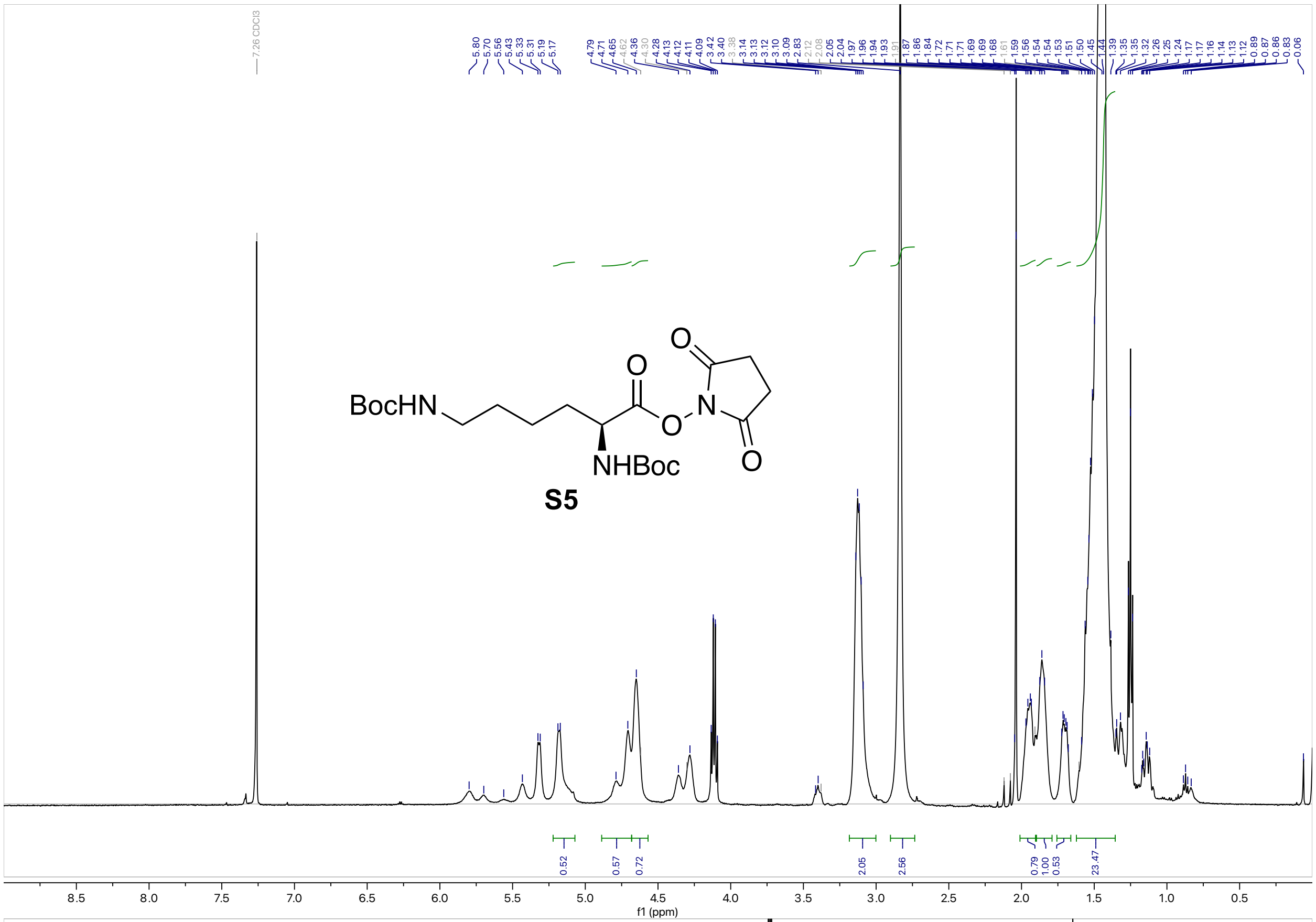
BocHN
NHBoc
O
N
S5
f1 (ppm)

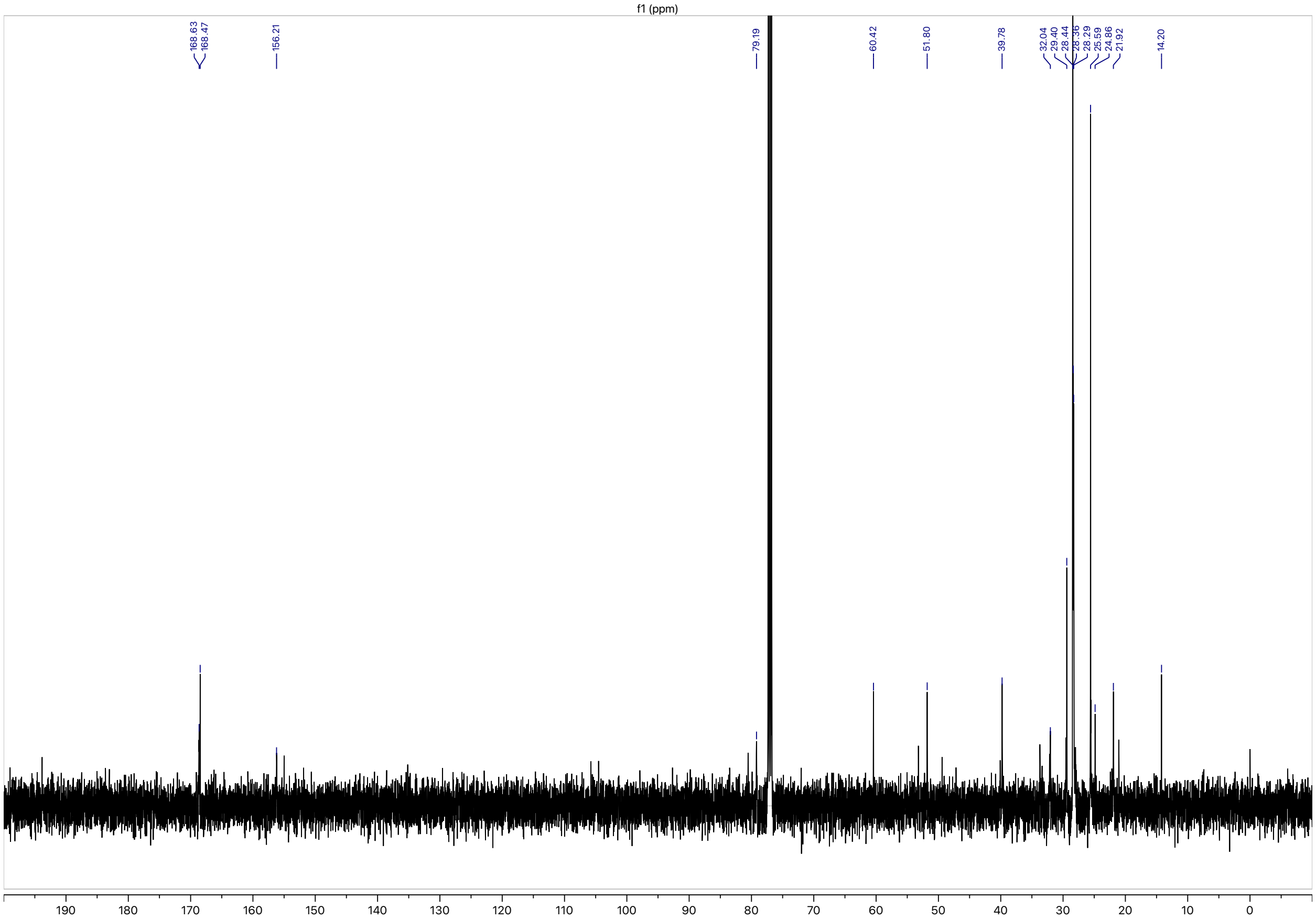
f1 (ppm)

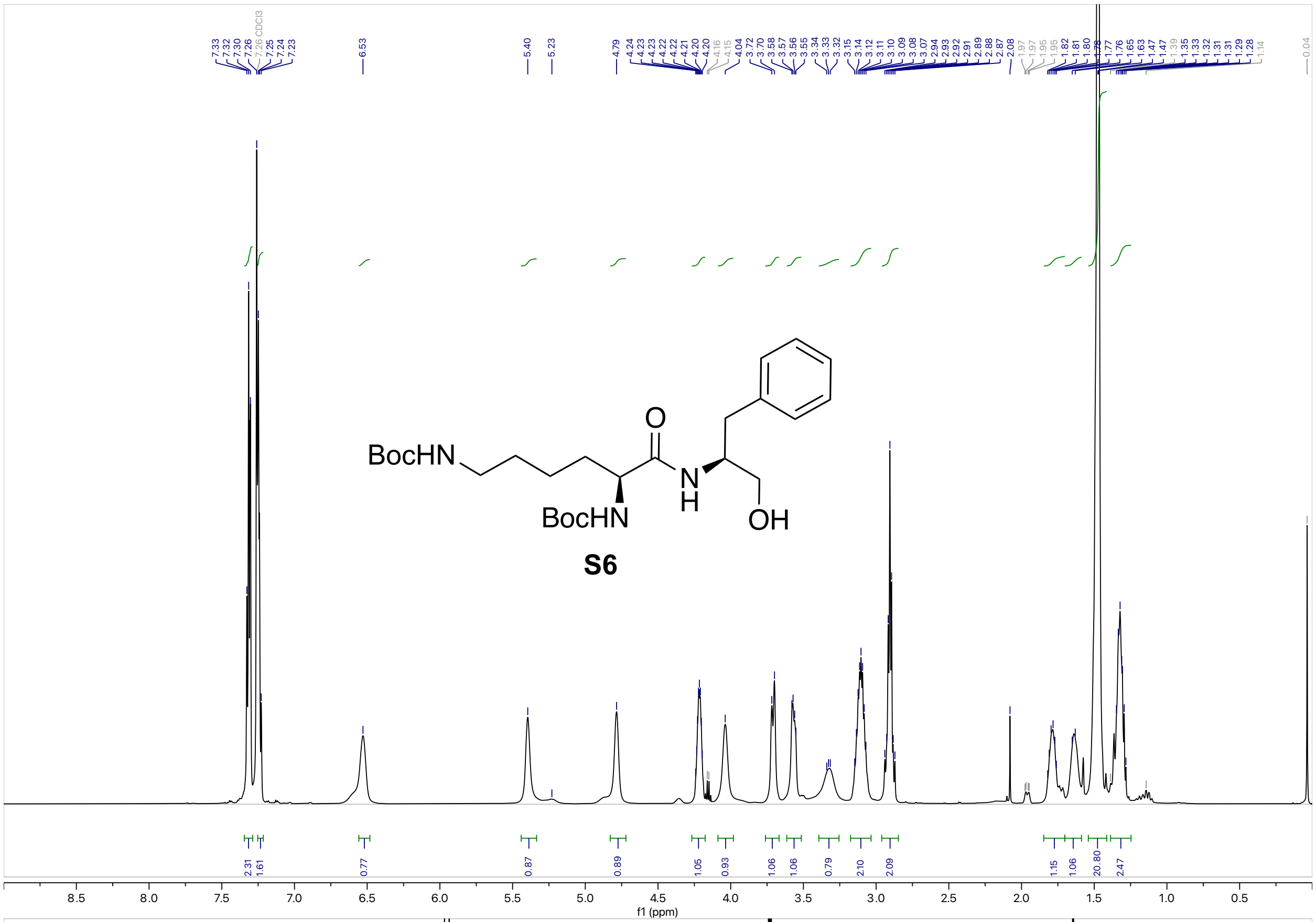
BocHN
BocHN
O
N
H
OH
S6
f1 (ppm)

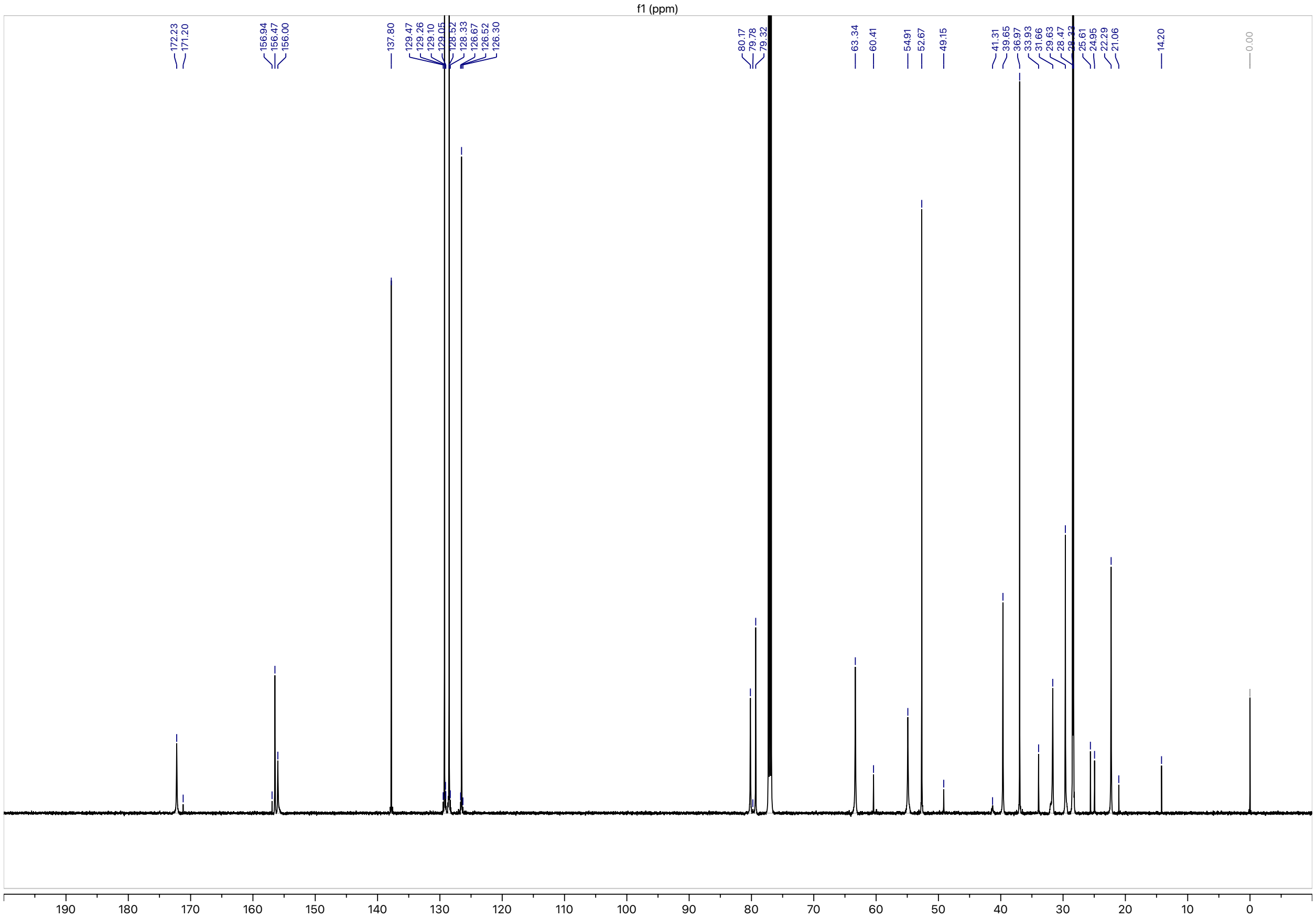
f1 (ppm)

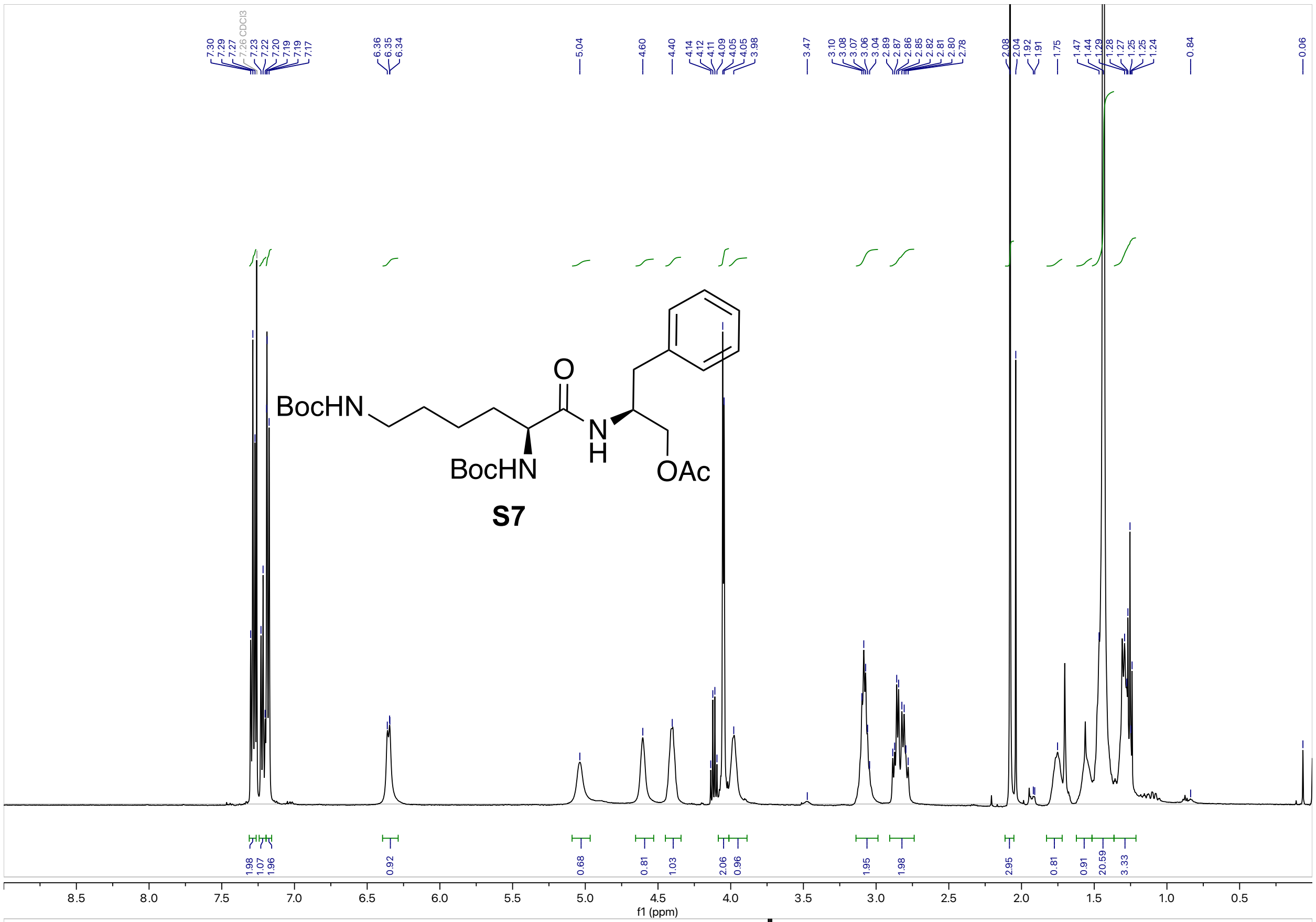
BocHN
O
N
H
BocHN
OAc
S7
f1 (ppm)

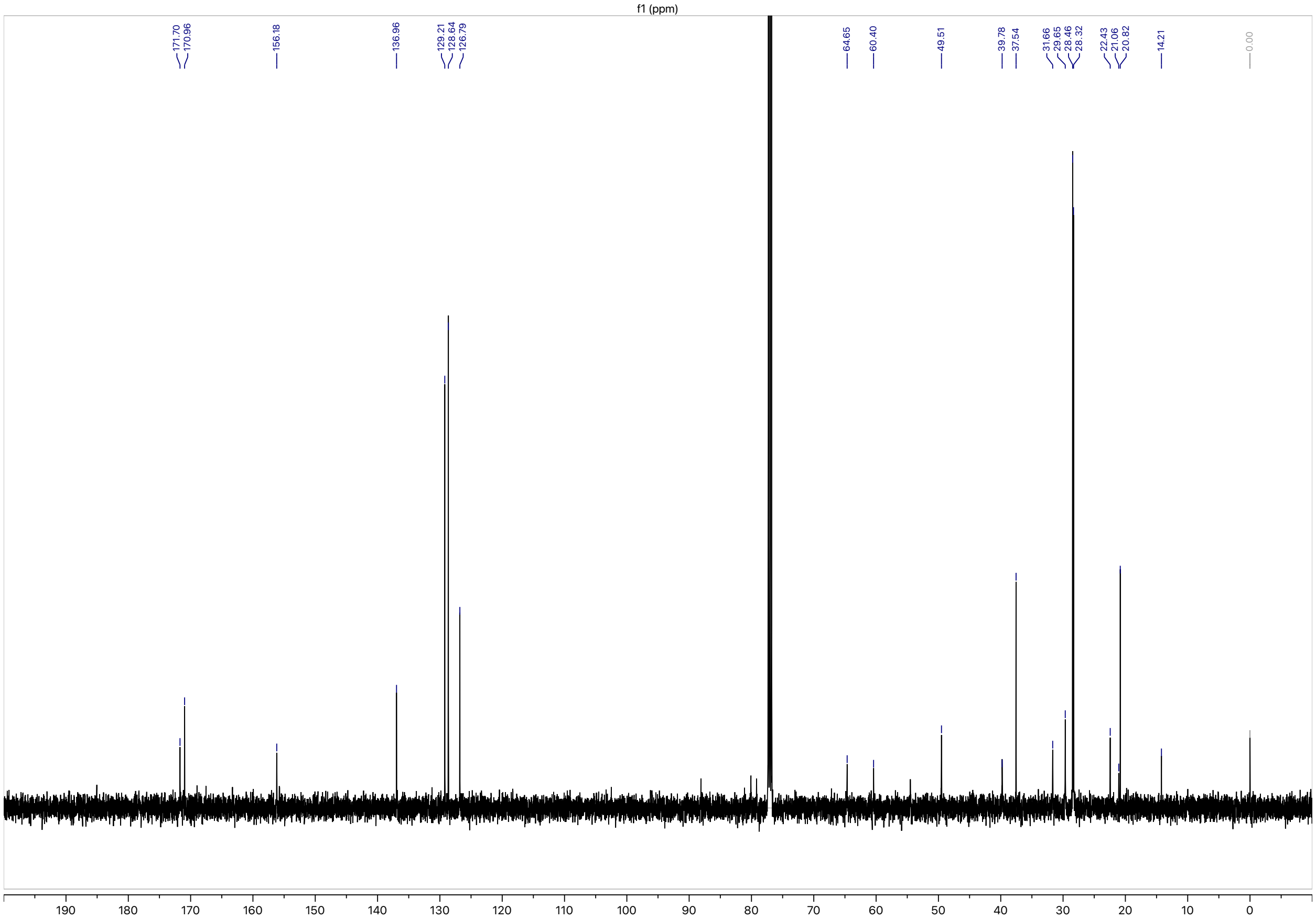
f1 (ppm)

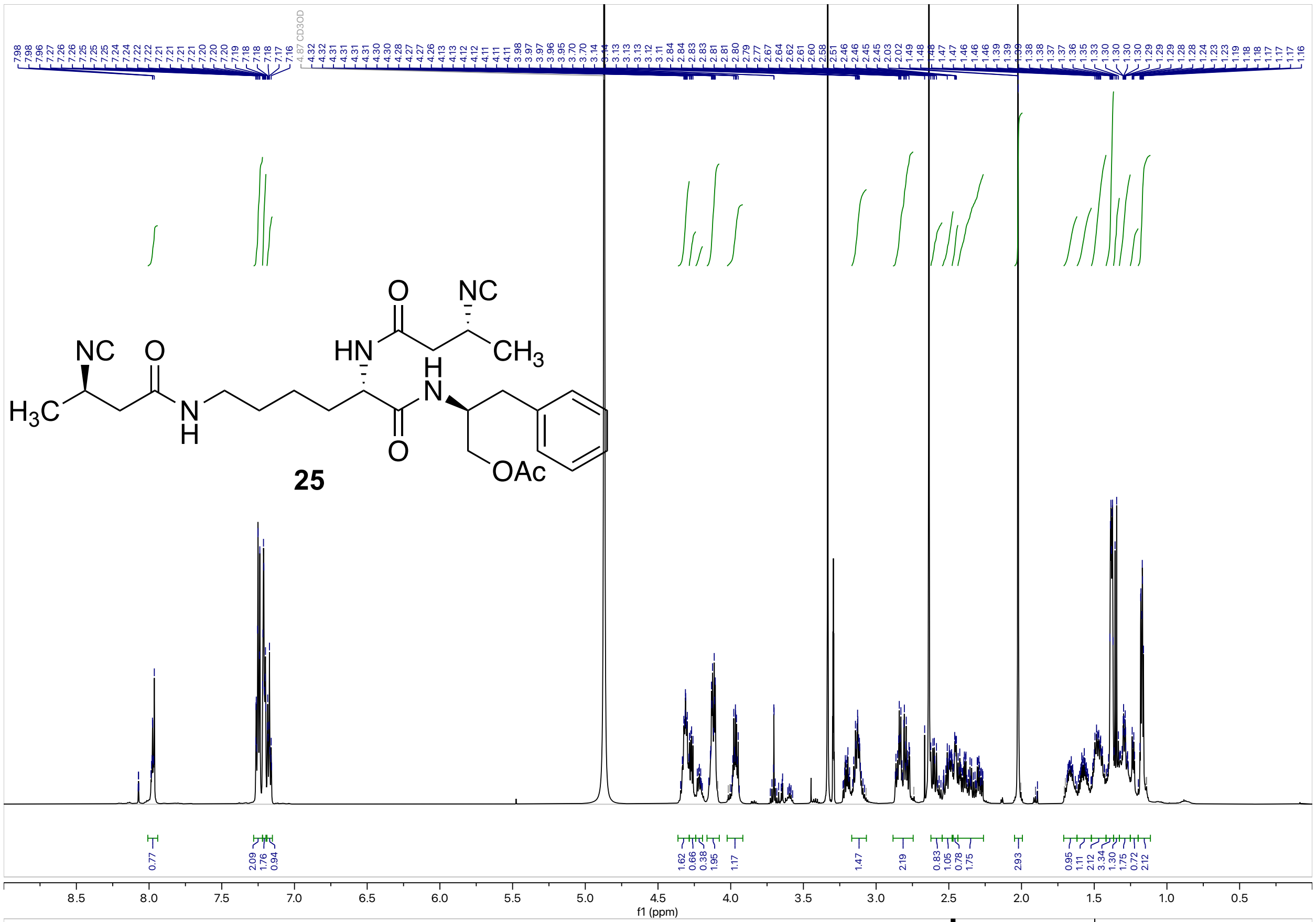
CD3OD
NC
O
HN
CH3
H3C
N
H
OAc
25
f1 (ppm)

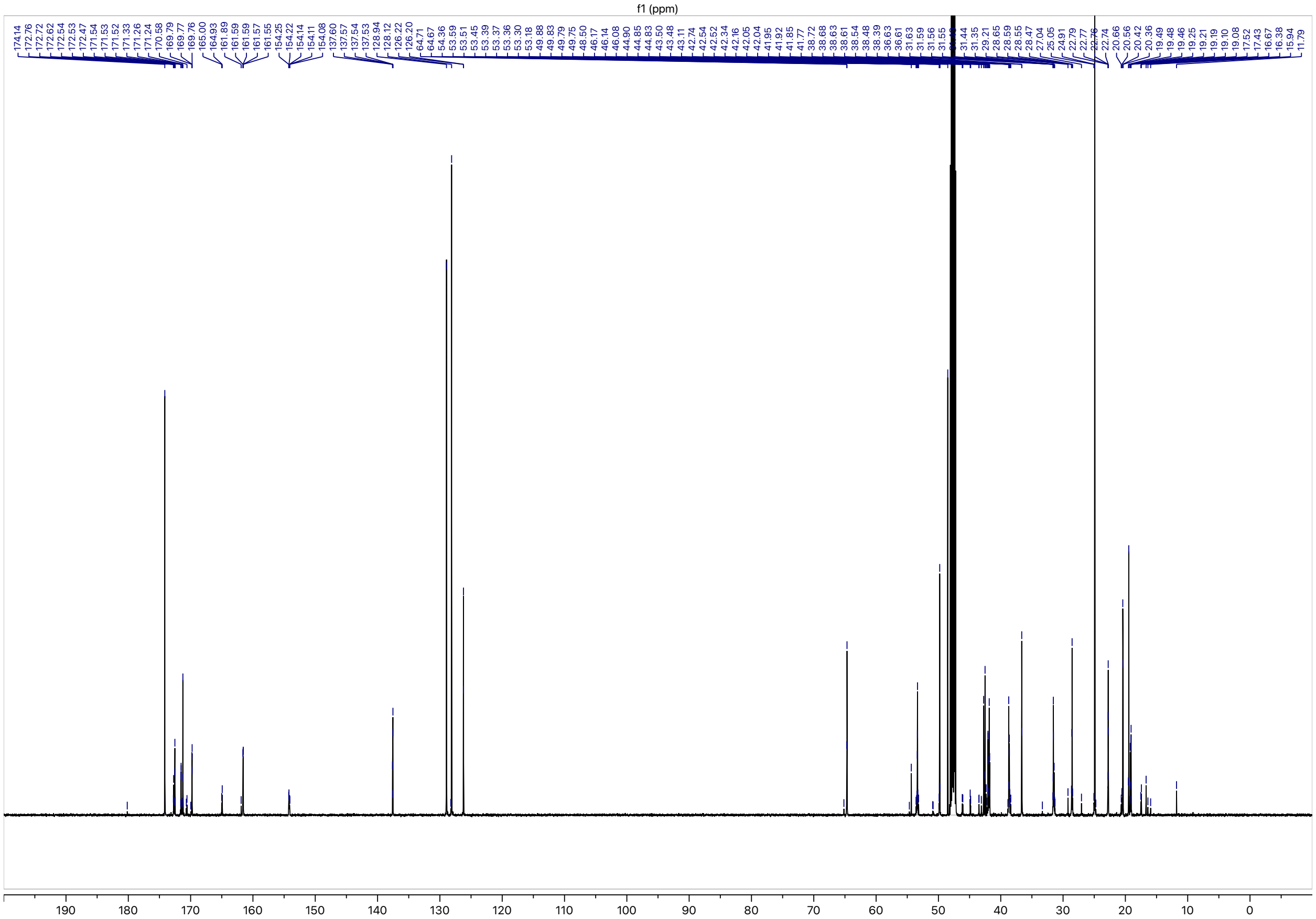
f1 (ppm)

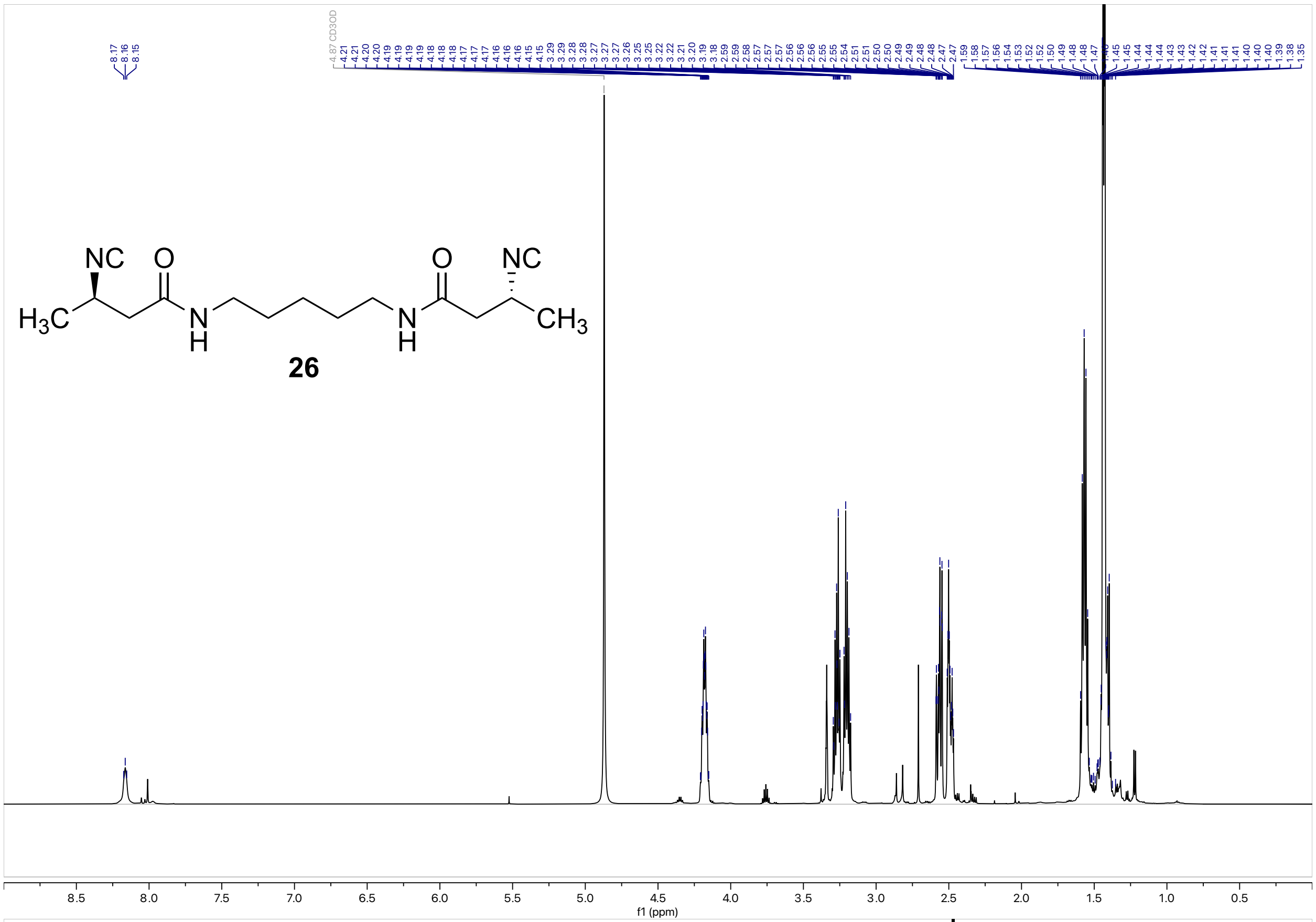
NC
O
O
NC
H_3C
N
H
N
H
CH_3
26
f1 (ppm)

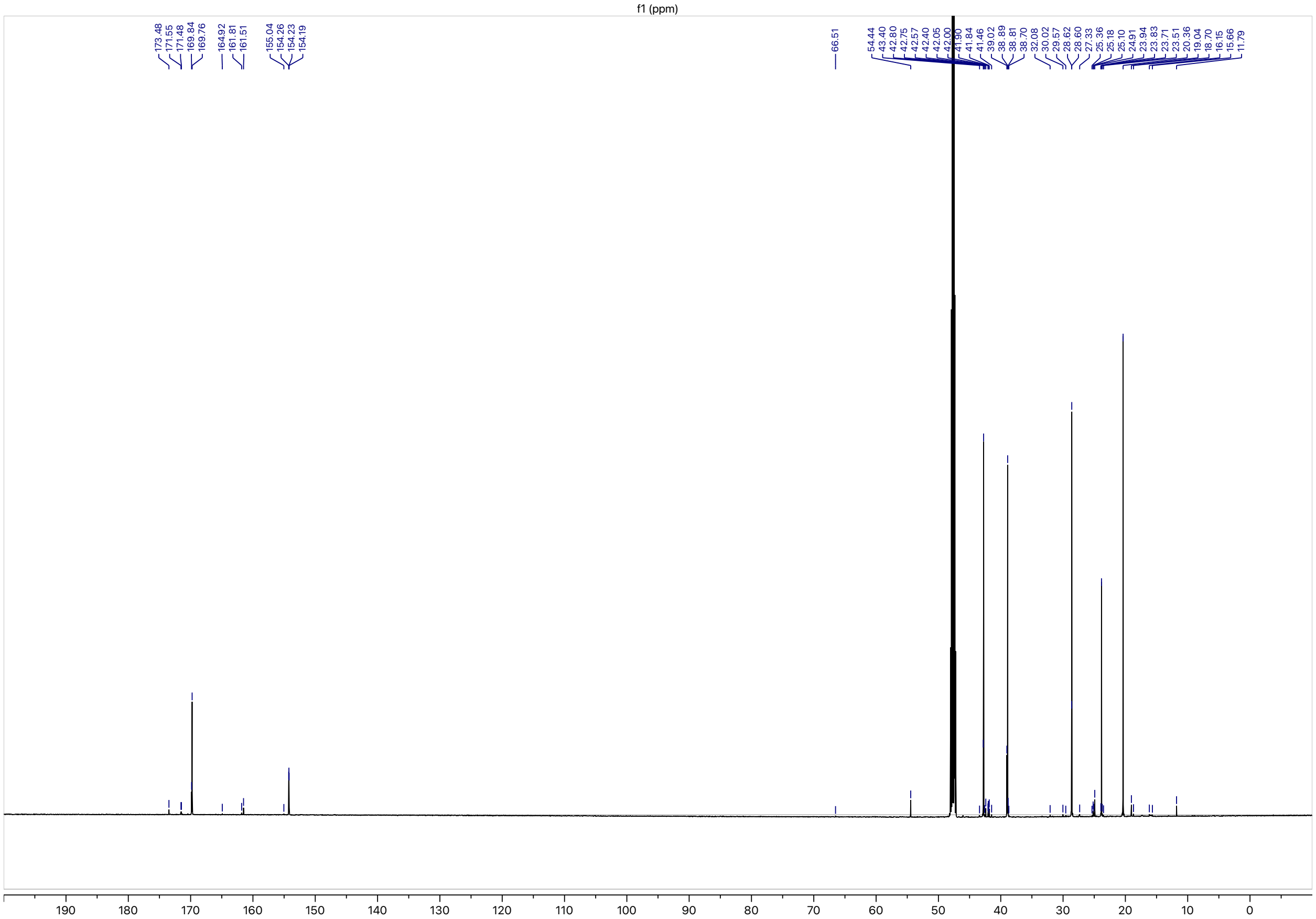
f1 (ppm)

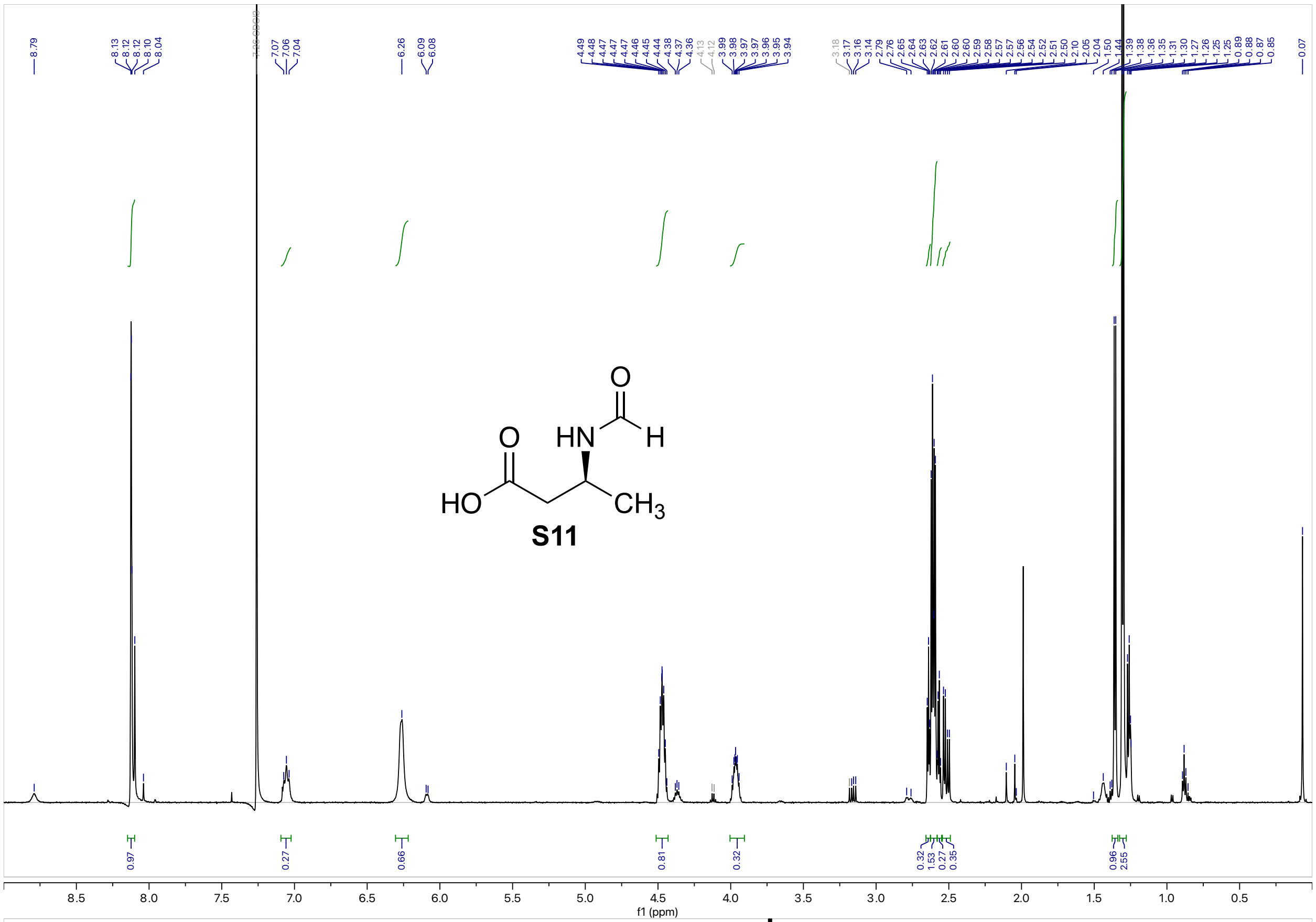
O
HN
H
O
HO
CH3
S11
f1 (ppm)

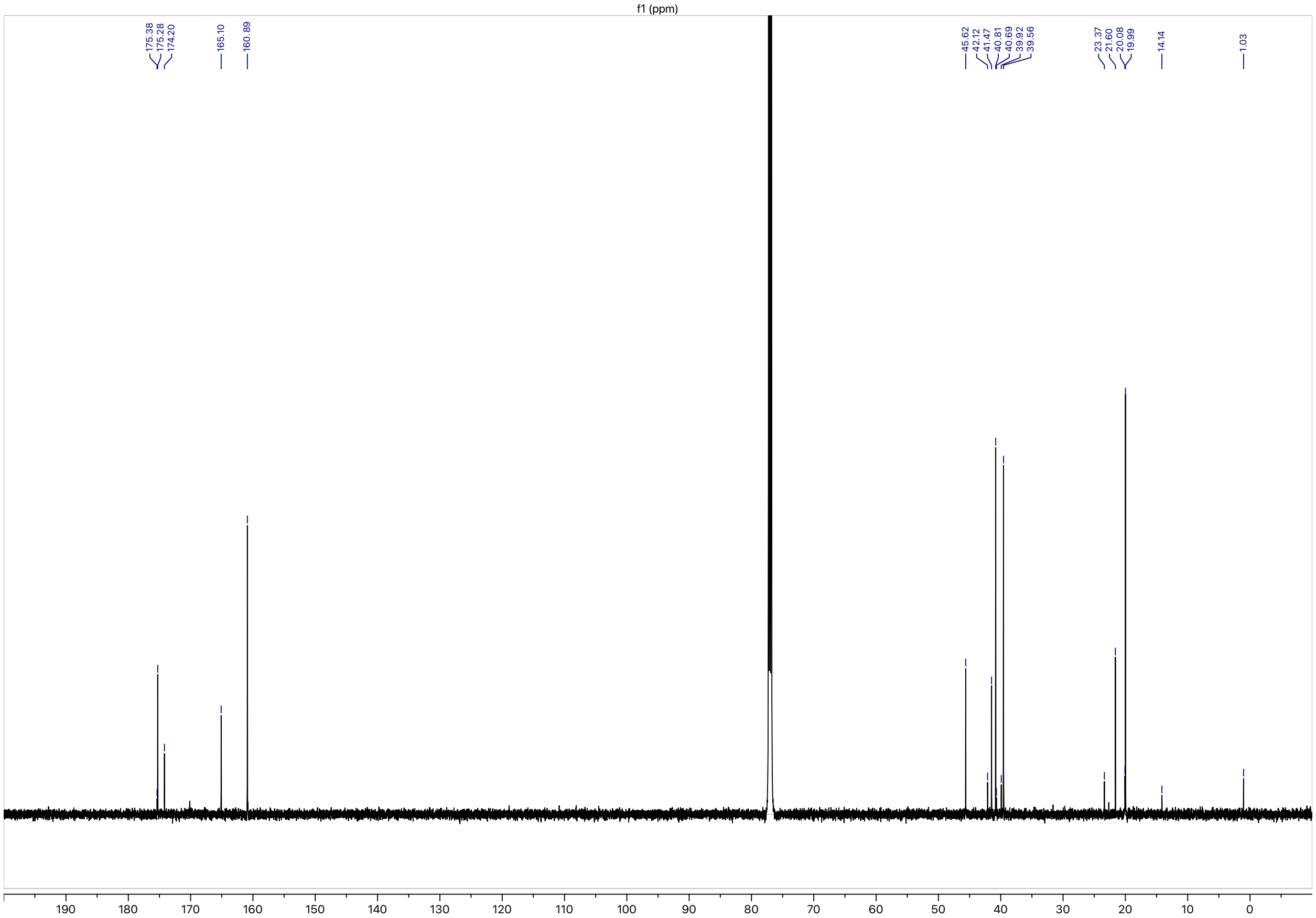
175.38
175.28
174.20
165.10
160.89
45.62
42.12
41.47
40.81
40.69
39.92
39.56
23.37
21.60
20.08
19.99
14.14
1.03
f1 (ppm)

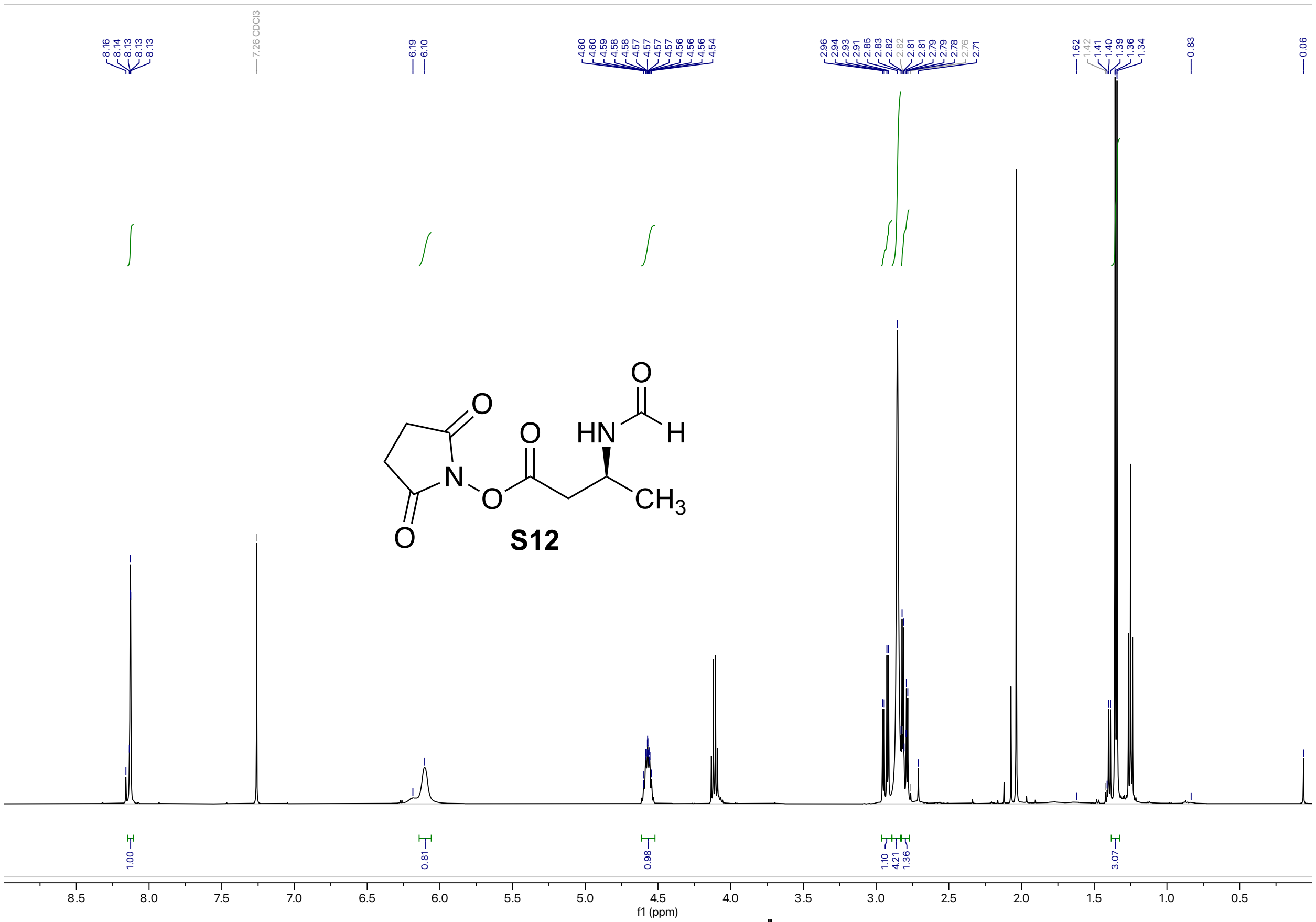
8.16
8.14
8.13
8.13
8.13
7.26 CDCl3
6.19
6.10
4.60
4.60
4.59
4.58
4.58
4.57
4.57
4.57
4.57
4.56
4.56
4.54
2.96
2.94
2.93
2.91
2.85
2.83
2.82
2.82
2.81
2.81
2.79
2.78
2.76
2.71
1.62
1.42
1.41
1.40
1.39
1.36
1.34
0.83
0.06
O
HN
H
O
N
O
O
CH3
O
S12
1.00
0.81
0.98
1.10
4.21
1.36
3.07
f1 (ppm)

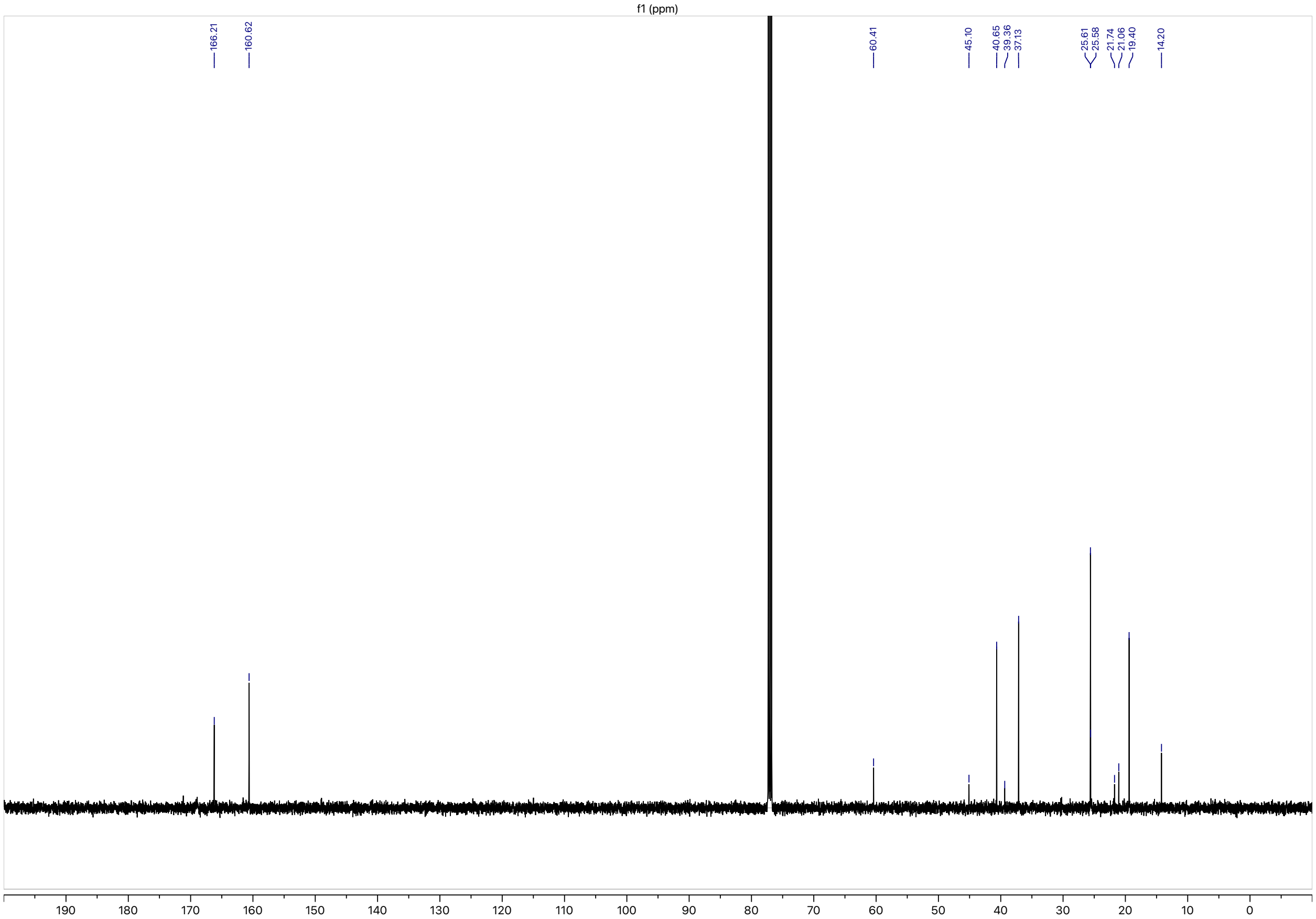
166.21
160.62
60.41
45.10
40.65
39.36
37.13
25.61
25.58
21.74
21.06
19.40
14.20
f1 (ppm)

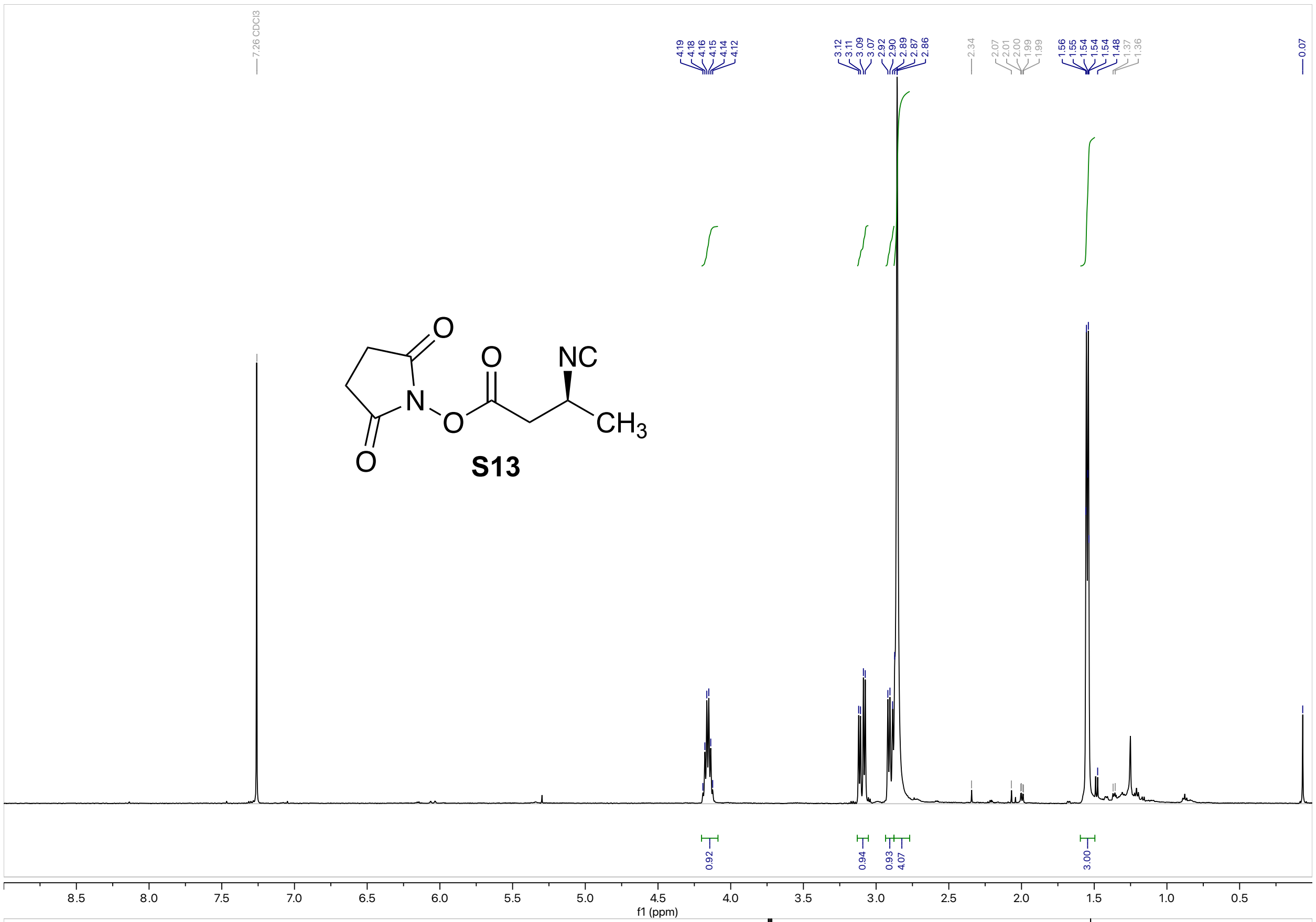

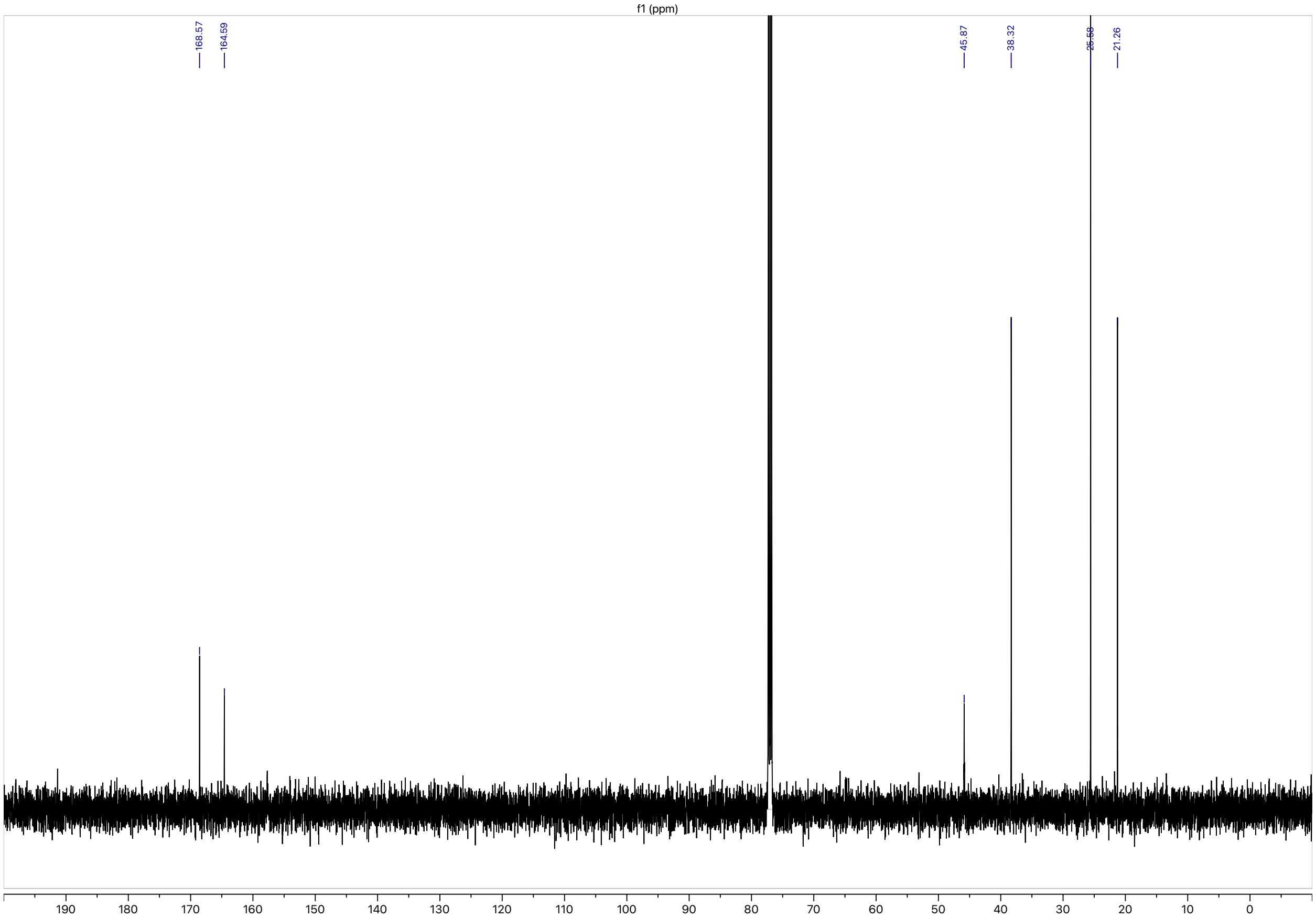

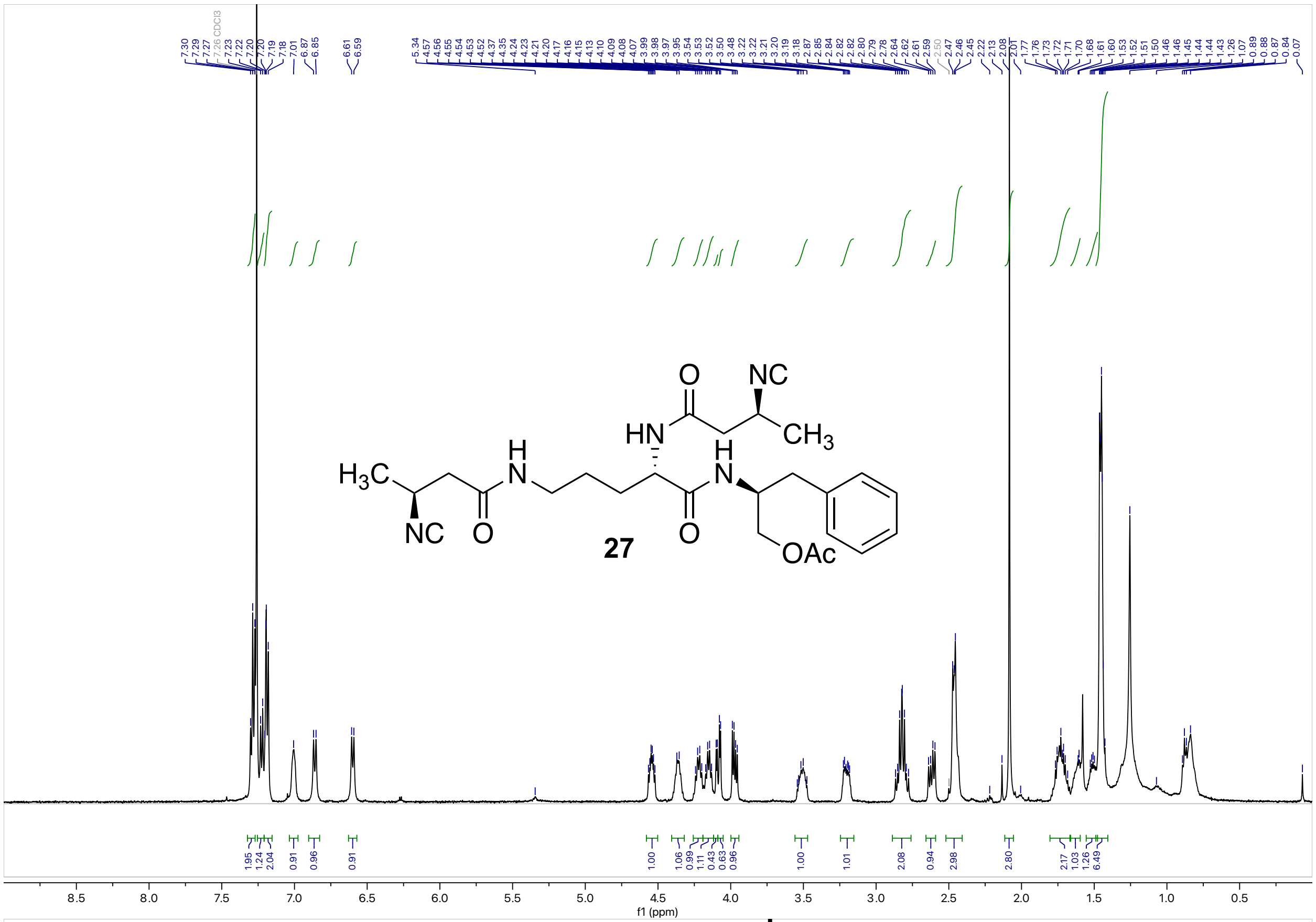
H
H
HN
NC
CH3
H3C
N
N
NC
O
O
O
O
27
OAc
f1 (ppm)

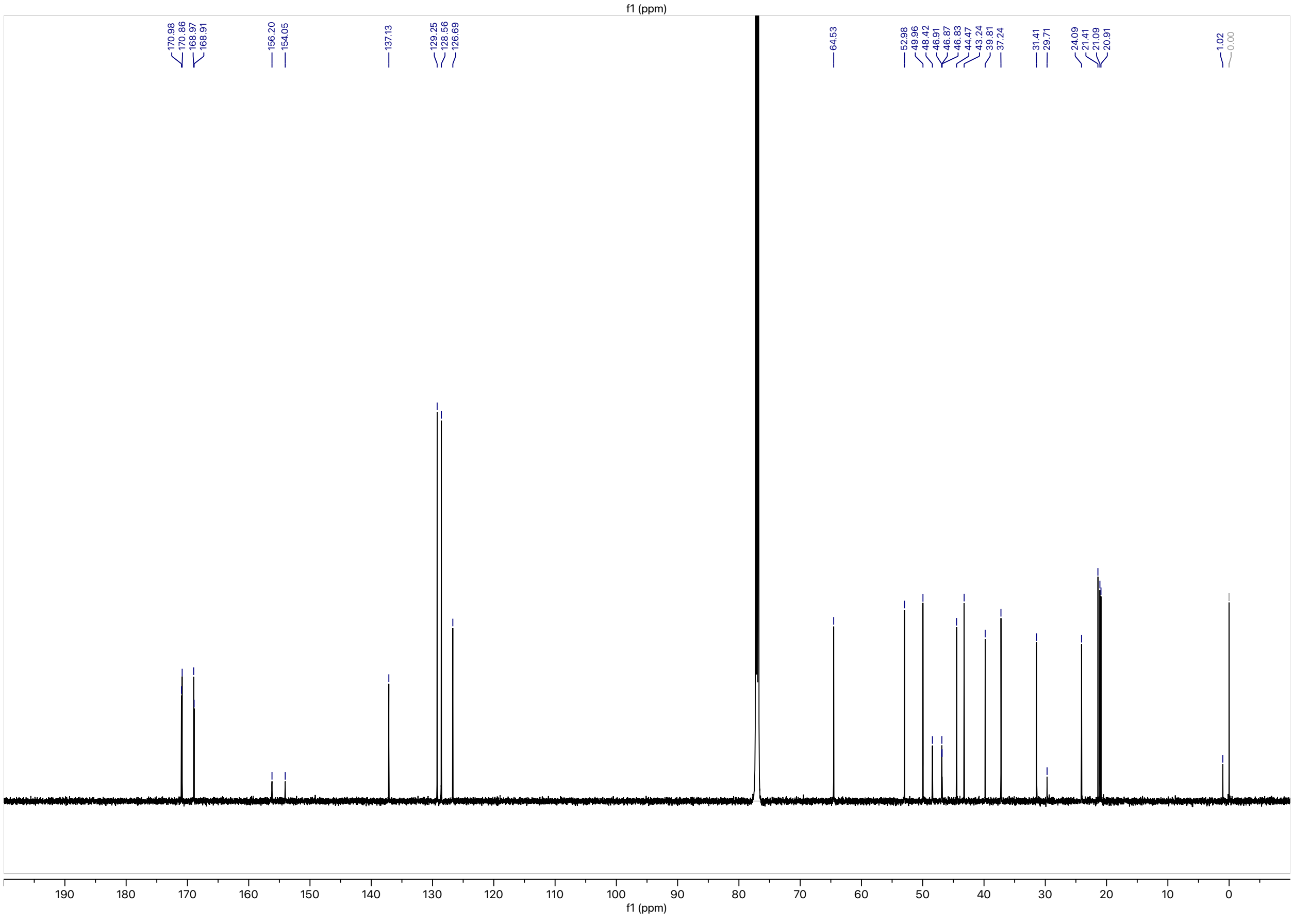
f1 (ppm)

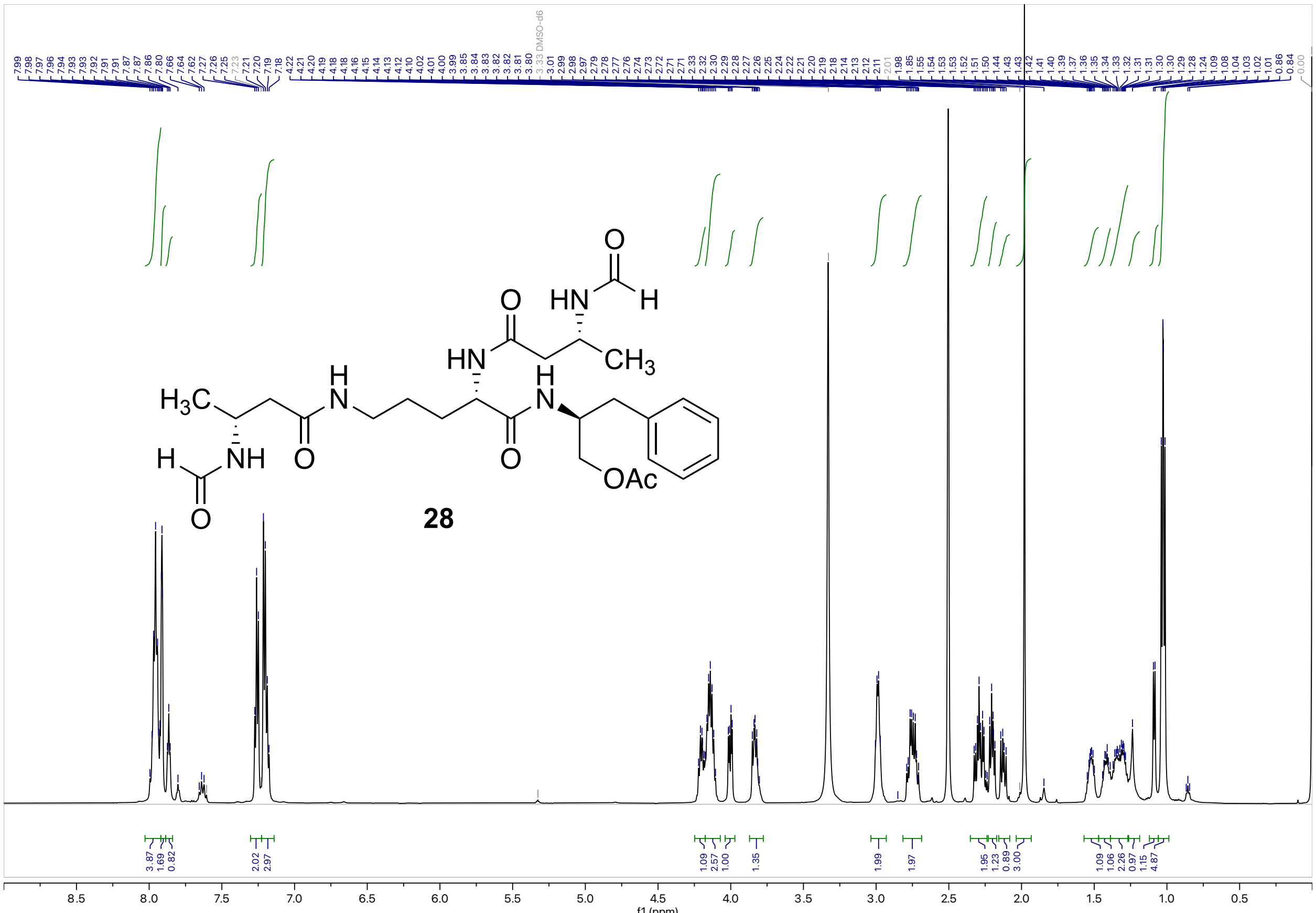

28

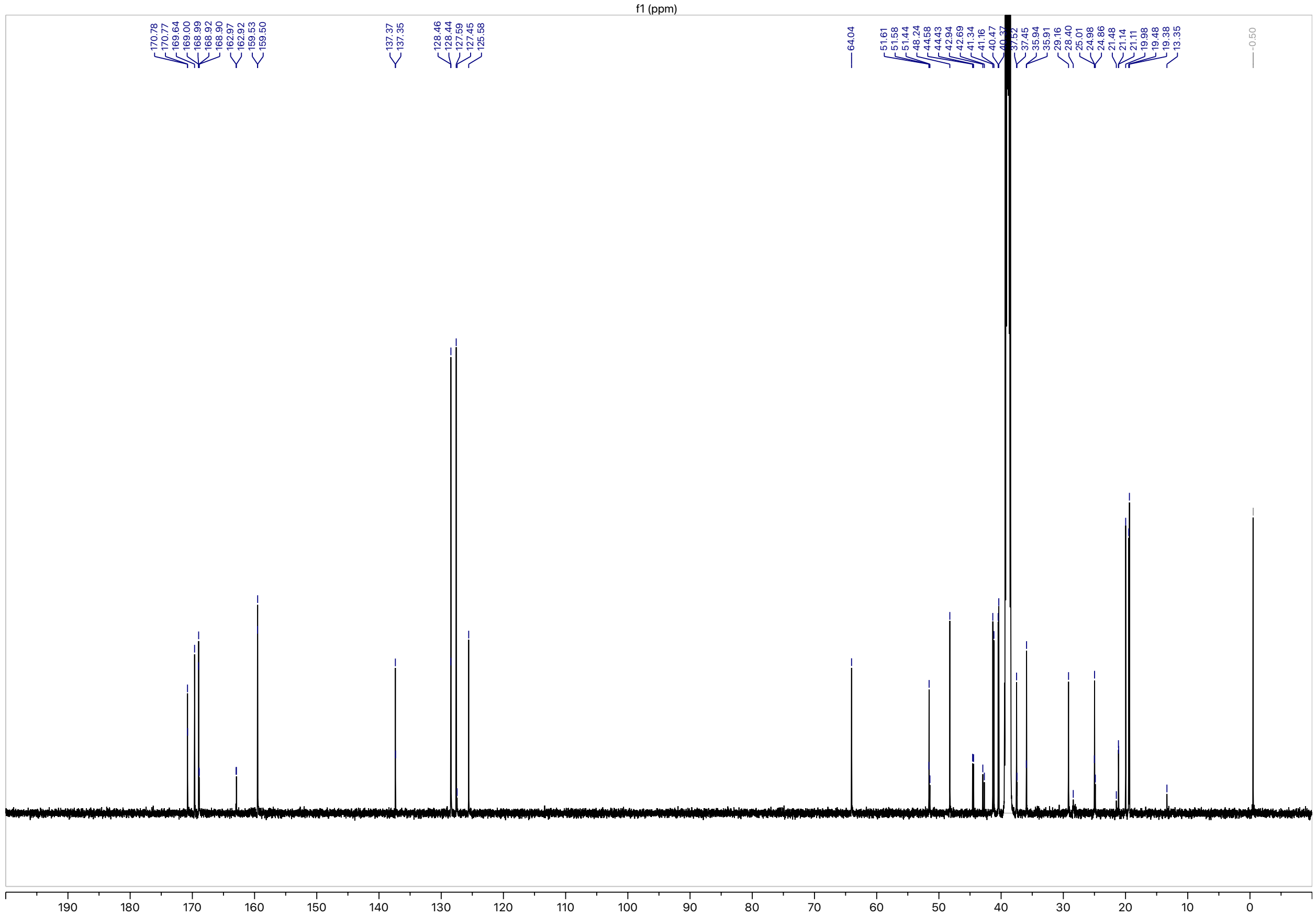

Supplement: Supplementary file 1 [file ja6c09506_si_001.pdf]
